# Comprehensive and accurate tracking of carbon origin of LC-tandem mass spectrometry collisional fragments for ^13^C-MFA

**DOI:** 10.1007/s00216-016-0174-9

**Published:** 2017-01-23

**Authors:** Jannick Kappelmann, Bianca Klein, Petra Geilenkirchen, Stephan Noack

**Affiliations:** Institute of Bio- and Geosciences, IBG-1: Biotechnology, Forschungszentrum Jülich GmbH, Jülich, 52425 Germany

**Keywords:** Carboxylate anions, Collision-induced dissociation, Fragmentation pathways, LC-ESI-MS/MS, ^13^C-Metabolic Flux Analysis, Tandem mass isotopomer

## Abstract

In recent years the benefit of measuring positionally resolved ^13^C-labeling enrichment from tandem mass spectrometry (MS/MS) collisional fragments for improved precision of ^13^C-Metabolic Flux Analysis (^13^C-MFA) has become evident. However, the usage of positional labeling information for ^13^C-MFA faces two challenges: (1) The mass spectrometric acquisition of a large number of potentially interfering mass transitions may hamper accuracy and sensitivity. (2) The positional identity of carbon atoms of product ions needs to be known. The present contribution addresses the latter challenge by deducing the maximal positional labeling information contained in LC-ESI-MS/MS spectra of product anions of central metabolism as well as product cations of amino acids. For this purpose, we draw on accurate mass spectrometry, selectively labeled standards, and published fragmentation pathways to structurally annotate all dominant mass peaks of a large collection of metabolites, some of which with a complete fragmentation pathway. Compiling all available information, we arrive at the most detailed map of carbon atom fate of LC-ESI-MS/MS collisional fragments yet, comprising 170 intense and structurally annotated product ions with unique carbon origin from 76 precursor ions of 72 metabolites. Our ^13^C-data proof that heuristic fragmentation rules often fail to yield correct fragment structures and we expose common pitfalls in the structural annotation of product ions. We show that the positionally resolved ^13^C-label information contained in the product ions that we structurally annotated allows to infer the entire isotopomer distribution of several central metabolism intermediates, which is experimentally demonstrated for malate using quadrupole-time-of-flight MS technology. Finally, the inclusion of the label information from a subset of these fragments improves flux precision in a *Corynebacterium glutamicum* model of the central carbon metabolism.

**Electronic supplementary material:**

The online version of this article (doi:10.1007/s00216-016-0174-9) contains supplementary material, which is available to authorized users.

## Introduction

### Benefit of positional ^13^C-label data for ^13^C-MFA

^13^C-Metabolic Flux Analysis (^13^C-MFA) has developed into a cornerstone technique of modern omics technologies, enabling to infer the so-called fluxome. On the experimental side, it relies on measuring the ^13^C-label distribution in central metabolism intermediates; a task that is most commonly accomplished by mass spectrometry (MS) [1]. This distribution is then evaluated by means of a carbon atom transition model to determine in vivo reaction rates [2–5]. The labeling experiment in combination with the model-based data evaluation is known as ^13^C-MFA [6].

In recent years, efforts have been directed at measuring not only the mass isotopomer (isotopologue) distribution (MID) of intact metabolites but also positional ^13^C-label enrichment in metabolite fragments originating from a carbon backbone cleavage. The arising vector of relative product ion mass isotopomer intensities is referred to as tandem mass isotopomer distribution (TMID) [7]. The advantage of positionally resolved ^13^C-label information for increased precision of flux estimation has been shown experimentally for both gas chromatography-electron ionization-MS/MS (GC-EI-MS/MS) and liquid chromatography-electrospray ionization-MS/MS (LC-ESI-MS/MS) [8–10]. Recent simulation studies demonstrated the gain in statistical quality of flux estimators using positional ^13^C-enrichment of molecules with more than three carbon atoms [3] and even for situations in which the carbon origin is not known [7]. Moreover, some pathway fluxes (like Entner-Doudoroff and Embden-Meyerhof-Parnas) can only structurally be identified from ^13^C-labeling data using positionally resolved ^13^C-enrichment in pyruvate or alanine [11].

### Benefit of tandem MS technology for ^13^C-MFA

Fragmentation patterns of metabolites differ between analytical platforms. GC-EI-MS usually provides rich product ion spectra generated upon EI [1]. Coupled to a triple-quadrupole MS (QqQ), GC-EI offers the possibility to further fragment EI-product ions by collision-induced dissociation (CID), providing an unrivaled degree of carbon backbone fragmentations. Choi and Antoniewicz [12] used this combination of EI and subsequent CID of precursor ions to acquire the TMID of several fragments of aspartate (Asp) concurrently. By combining the obtained TMIDs, the authors succeeded in elucidating the entire isotopomer distribution of Asp, the inclusion of which into the flux estimation improved statistical flux identifiability [3].

In contradistinction to GC-EI-MS, fragmentation on LC-ESI-MS/MS systems relies mainly on CID of intact quasi-molecular ions. Nonetheless, LC-ESI-MS/MS technology is widely used to quantify ^13^C-label enrichment in central metabolism intermediates [9, 13–18] and will continue to be used due to some inherent advantages:

1. Cell extracts do not need to be evaporated to dryness in order to perform methoximation or ethoximation and subsequent trimethylsilyl or *tert*-butyldimethylsilyl derivatization [19–21]. Although the derivatization steps may be automated [22], the total sample processing time in LC-ESI-MS/MS is inherently shorter since cell extracts can be injected directly, at the cost of retention time reproducibility.
2. LC enables to separate a large part of the central carbon metabolism intermediates in a single chromatographic run using hydrophilic interaction chromatography [23–25].
3. Correcting the obtained TMID from LC-ESI-MS/MS or MID from LC-ESI-MS for the occurrence of natural isotopes is inherently more precise and less biased due to the absence of heterogroups from derivatization agents. The correction process of GC-MS/MS mass fractions was shown to inflate the coefficient of variation for low intensity (tandem) mass isotopomer fractions [21].
4. Whereas LC-MS is only slightly more popular than GC-MS in the realm of metabolomics [26], its lead in quantitative proteomics is unrivaled [27, 28]. As such, an LC-ESI-MS/MS device can serve as one-stop platform for proteomics, metabolomics as well as fluxomics, requiring just a change of column between proteomics applications on the one hand and metabolomics and fluxomics experiments on the other hand.

### Challenge of accurate TMID acquisition

Regardless of the chromatographic separation, the generation of TMID data of as many fragments as analytically possible currently faces two challenges, the first of which concerns product ion quantification. Quadrupole technology for product ion detection imposes limits on the maximal number of fragment ion mass traces that can be acquired with a given accuracy (determined by cycle time) and sensitivity (influenced by dwell time) due to the interdependency of dwell time, cycle time, and number of mass transitions. This issue was addressed by Rühl et al. [9] for LC-ESI-QqQ by setting the Q3 transmission windows such that all isotopomers contributing to a given mass isotopomer are transmitted or by setting the Q1 transmission windows to let pass all mass isotopomers contributing a certain tandem mass isotopomer. The inherently lower acquisition rate of a scanning quadrupole device may also be circumvented by restricting the number of acquired product ion spectra to the mass isotopomers surpassing an intensity threshold in an information-dependent acquisition as employed by McCloskey et al. [17]. Alternatively, the entire product ion spectrum of a given mass isotopomer may be acquired at once using a non-scanning mass analyzer like time-of-flight MS (TOF MS) as demonstrated by Mairinger et al. [21] for GC-chemical ionization-quadrupole TOF MS (GC-CI-QTOF).

### Challenge of correct tracking of TMID carbon origin

The second challenge concerns the correct interpretation of the acquired TMID which necessitates knowledge of the positional origin of fragment carbon atoms from the precursor ion. This knowledge may be inferred from annotating the corresponding mass peaks with a structure. For structural elucidation, the above-mentioned studies recurred to fragment spectrum prediction using heuristic fragmentation rules as implemented in MassFrontier or combinatorial fragmentation as implemented in PeakView or FiD [29]. The latter approach exhaustively enumerates all fragment structures as subgraphs of the linear precursor ion matching the observed (accurate) mass [30]. However, these approaches will fail to predict cyclic structures or product ions derived thereof because computational approaches to predict fragment structures do not include possible rearrangement reactions except for hydrogen to keep the dimensionality of the fragment space low enough for the problem to be computationally feasible [29]. The latest development for the computational prediction of MS/MS spectra is termed Competitive Fragmentation Modeling and is based on a machine-learning model trained on mass spectra of known structures [31]. The sophistication notwithstanding, this approach will not be able to capture cyclic fragment structures because the space of admissible structures is as restrictive as in the established computational approaches [32]. Cyclic product ion structures, though, occur frequently for amino acids in positive ionization mode [33]. Furthermore, the studies of Rühl et al. [9] and McCloskey et al. [17] left the product ion structures for many fragment ions of central carbon metabolism open, not least because several alternative fragment structures may explain a given elemental composition. This is especially true for fragments of di- and tricarboxylic acids corresponding to a neutral loss of carbon dioxide. If different carboxygroups are lost simultaneously, the resulting mass peak is composed of a mixed carbon ancestry. This has been shown to affect the fragment of citrate (Cit) used for ^13^C-measurement in the study of Alves et al. [18]: the used fragment is symmetrically derived from two positions within the precursor molecule [18]. Although this study is the first to use LC-ESI-MS/MS data with experimentally validated carbon origins, it falls short of a comprehensive characterization of carbon atom fate over the central carbon metabolism, reporting the carbon ancestry for just one fragment of glutamate (Glu), Cit, and aspartate (Asp) each.

### Aims and structure of this study

This contribution addresses the above-mentioned challenges in the following way:

1. We first take care in choosing the precursor ion species (ionization and adduct) for each metabolite to maximize sensitivity. Contrary to previous studies, we investigate carbon origins for amino acids in positive ionization mode because this metabolite class consistently showed lower limits of detection in positive ionization mode [34]; an important consideration given the loss of sensitivity inherent in tandem MS acquisition [21].
2. We apply accurate MS/MS technology to systematically and comprehensively determine the elemental composition of product ions from 72 metabolites. Importantly, we select to study at least all fragments the intensity of which surpasses a threshold over a broad collision energy ramp. Hence, we analyze the maximal amount of carbon fragmentation information contained in the respective spectrum. Following this approach, we cover all dominant product ion peaks of all major central carbon metabolism intermediates.
3. Using a unique collection of selectively labeled standards for 16 metabolites, which is commercially not available, we comprehensively experimentally validate the carbon identity of CID fragments across the entire product ion spectrum of central carbon metabolism intermediates and amino acids.
4. Information from 2 and 3 was compiled to assemble entire fragmentation pathways for pyruvate (Pyr), oxaloacetate (OAA), α-ketoglutarate (αKG), malate (Mal), Asp, cis-Aconitate (cis-Aco), and Cit in negative ionization mode. These pathways were extensively validated using published fragmentation mechanism and product ion spectra of presumed intermediates occurring during fragmentation.

Following this approach, we could prove that for several fragments of di- and tricarboxylic acids as well as sugar phosphates, competing fragmentation reactions with differing carbon origin contribute to a given accurate mass peak. All available information was used to compile a set of intense fragment ions for positional ^13^C-measurements using LC-ESI-MS/MS covering sugar phosphates, organic and amino acids as well as nucleoside phosphates. For some metabolites, we show that the ^13^C-label information contained in the respective fragmentation patterns enables full isotopomer resolution. We put this claim to the test on an LC-ESI-QTOF MS system using a defined mixture of ^13^C-isotopomers of Mal. Finally, we show that inclusion of the TMID of a subset of the entire collection of fragments into the flux estimation result in an up to 20-fold improvement of flux standard deviations.

## Material and methods

### Chemicals and enzymes

If not otherwise stated, all non-labeled chemicals were acquired from Sigma Aldrich (Schnelldorf, Germany). Acetyl-CoA trilithium salt was acquired from Roche Diagnostics (Mannheim, Germany). Succinic semialdehyde was purchased from Santa Cruz Biotechnology (Santa Cruz, CA, USA). (R)-3-Aminodihydrofuran-2,5-dione hydrochloride was purchased from Interchim (Montlucon, France). Trans-aconitic anhydride was synthesized by FCH Group (Chernigiv, Ukraine) and acquired through AKOS GmbH (Steinen, Germany). All stable ^13^C-isotopes had an atom purity of 99%. H_2_[^18^O] was acquired from Sigma Aldrich and had an ^18^O-enrichment of 10%. NaH[^13^C]O_3_ was acquired from MSD Isotopes (Montreal, Canada). Sodium [2-^13^C]pyruvate and sodium [3-^13^C]pyruvate were purchased at Isotech (Miamisburg, OH, USA). Sodium [1-^13^C]pyruvate, [1-^13^C]L-glutamic acid, and sodium [1,2,3,4-^13^C]α-ketoglutarate were acquired from Cambridge Isotope Laboratories (Andover, MA, USA). [4-^13^C]L-aspartic acid, [1-^13^C]L-aspartic acid, [5-^13^C]L-methionine, and [6-^13^C]L-lysine dihydrochloride were purchased from Sigma Aldrich (Schnelldorf, Germany). [2-^13^C]Glucose, [1,2-^13^C]Glucose, and [6-^13^C]Glucose were acquired from Omicron Biochemicals (South Bend, IN, USA) and [1-^13^C]Glucose from Campro Scientific (Berlin, Germany). Citric anhydride was synthesized according to Repta and Higushi [35]. Acetic anhydride and acetic acid for the synthesis of citric anhydride were acquired from Roth (Karlsruhe, Germany).

The assay enzymes pyruvate carboxylase (bovine liver), L-glutamic dehydrogenase (bovine liver), citrate synthase (porcine heart), aconitase (porcine heart), phosphoglucose isomerase (*Saccharomyces cerevisiae*), fructose-6-phosphate kinase (*Bacillus stearothermophilus*), and glucose-6-phosphate dehydrogenase (*Leuconostoc mesenteroides*) were acquired from Sigma Aldrich (Schnelldorf, Germany); malate dehydrogenase (porcine heart) and hexokinase (*Saccharomyces cerevisiae*) were purchased at Roche Diagnostics GmbH (Mannheim, Germany).

### In vitro enzyme assays to generate selectively labeled ^13^C-species and ^18^O-species

To generate selectively labeled Mal and OAA species, a modified version of the pyruvate carboxylase assay of the manufacturer was employed [36]. Sodium [1-^13^C]pyruvate, sodium [3-^13^C]pyruvate, or sodium pyruvate was incubated with either NaHCO_3_ or NaH[^13^C]O_3_ to generate [1-^13^C]Mal, [3-^13^C]Mal, and [U-^12^C]Mal or [1,4-^13^C]Mal, [3,4-^13^C]Mal, and [4-^13^C]Mal, respectively. The assay volume was 1 ml and contained 98.5 mM triethanolamine-HCL (pH 8), 4.93 mM MgSO_4_, 6.45 mM sodium pyruvate, 0.026 mM acetyl-CoA, 0.99 mM Na_2_ATP, 14.85 mM sodium bicarbonate, 0.228 mM Na_2_NADH, and 2.61 units/ml of malate dehydrogenase. For the generation of selectively labeled OAA species, malate dehydrogenase and NADH were omitted. After the assay solution was allowed to thermally equilibrate at 30 °C in a Thermomixer, the reaction was started by pipetting 3 μl of pyruvate carboxylase into the reaction tube, resulting in an enzyme concentration of 0.18 units/ml. After gentle agitation, the assay mixture was incubated for 30 min without agitation. After the successful completion was ascertained by measuring the absorbance at 320 nm, the assay solution was 0.2 μm-filtrated and immediately stored at −20 °C. For LC-ESI-MS/MS analysis, the assay samples were diluted with methanol to a concentration of 45% (*v*/*v*) methanol and stored at −80 °C till analysis.

To generate selectively labeled αKG, a modified version of glutamate dehydrogenase assay of the manufacturer was employed [37]. The assay volume was 1 ml and contained 7.62 mM of either Glu or [1-^13^C]Glu and 0.015 units glutamate dehydrogenase per ml. The reaction was carried out at room temperature. After 60 min of incubation, successful completion was checked photometrically at 320 nm. All subsequent steps were carried out as described above.

Selectively labeled Cit was generated by using a modified version of the pyruvate carboxylase assay from above. Malate dehydrogenase and NADH were omitted from the assay solution. Substrates were sodium [1-^13^C]pyruvate, sodium [2-^13^C]pyruvate, and sodium [3-^13^C]pyruvate in combination with either NaHCO_3_ or NaH[^13^C]O_3_ and unlabeled acetyl-CoA. Additionally, the assay solution contained 0.1 mM 5,5′-dithiobis(2-nitrobenzoic acid) (dissolved in absolute ethanol at a concentration of 10 mM) to check the success of the enzymatic reaction photometrically. Before use in the assay, citrate synthase was diluted 1:10 in 3.2 M (NH_4_)_2_SO_4_ at pH 7. From this solution 3 μl was added to the assay, yielding a final enzyme concentration of 1 unit/ml. The reaction was again started by adding 3 μl pyruvate carboxylase, followed by gentle agitation. After 15 min of incubation at 30 °C, the samples were measured at 412 nm, processed, and stored as described above. For LC-ESI-MS/MS analysis, the assay samples were diluted with acetonitrile to a concentration of 60% (*v*/*v*) and stored at −80 °C till analysis. The same protocol was used for the production of ^13^C-isotopic standards of isocitrate (Icit) and cis-Aco, except that the assay solution was at pH 7.4. Additionally, 0.05 units/ml of aconitase, which was activated according to the manufacturer protocol [38], was added to the assay. For the production of [*hydroxy*-^18^O]Cit and [*hydroxy*-^18^O]Icit, 250 μl of a 25 mM solution of cis-Aco in 100 mM Tris–HCl at pH 7.4 was evaporated to dryness in a vacuum centrifuge. After the residue was dissolved in the same volume of H_2_[^18^O], 0.05 units/ml aconitase was added. All subsequent steps were carried out as described above.

Selectively labeled species of glucose 6-phosphate (G6P), fructose 6-phosphate (F6P), fructose 1,6-bisphosphate (FBP), and 6-phosphogluconate (6PG) were produced by incubating 200 μM of either [1-^13^C]Glc, [2-^13^C]Glc, [1,2-^13^C]Glc, or [6-^13^C]Glc with one or a combination of the enzymes hexokinase (at 5 units/ml), phosphoglucose isomerase (at 2 units/ml), fructose 6-phosphate kinase (at 3 units/ml), and glucose 6-phosphate dehydrogenase (at 1 unit/ml) in 100 mM triethanolamine-HCL, 6.5 mM MgCl_2_, 2.7 mM Na_2_ATP, and 0.83 mM NaNAD at pH 8 at 30 °C. All following steps were as above.

### LC-ESI-QqQ and MRM assays

The enzymatic assay mixtures for the generation of Mal, OAA, αKG, G6P, F6P, 6PG, and FBP were separated on an Agilent 1200 chromatography system (Agilent Technologies, Waldbronn, Germany) using a C18 synergy hydro reverse-phased column (150 × 2 mm, 4 μm, Phenomenex) coupled to a QqQ device (API 4000, ABSciex, Darmstadt, Germany) with an TurboSpray ion source in negative ionization mode. The separation of Mal-, OAA-, αKG-assays was performed isocratically with 84% buffer A and 16% buffer B at 0.45 ml/min and 40 °C. Sugar phosphate assays were separated using the gradient described in Paczia et al. [39]. The buffer compositions were as follows: buffer A—10 mM tributylamine, 15 mM acetic acid (pH 4.95); buffer B—methanol. The measured mass transitions of each metabolite were acquired with settings of collision energy (CE), declustering potential (DP), and cell exit potential (CXP) identified in a tuning with unlabeled analytical standard of the metabolite in question if not otherwise stated. Both mass transitions and MS parameter settings can be found in Electronic Supplementary Material S3. Acquisition was performed at a mass resolution of ±0.5 amu. Source temperature was 650 °C, GS1 and GS2 were both 70. Collision gas (CAD) was nitrogen at 5 psi. Further hardware parameters were as in the study of Luo et al. [40].

The product ion spectra on the QqQ device were generated upon direct infusion of metabolite standards dissolved in 1:1 MilliQ water/methanol except for sugars of which a solution in ammonium acetate buffer at pH 9.2 was infused. Citric anhydride was injected as solution in 11% acetic anhydride in acetic acid (*v*/*v*). Each spectrum was the combination of 26 MCA scans recorded over a collision energy ramp from −130 to −5 eV for negative ionization and +5 to +130 eV for positive ionization at an optimized DP value. We provide each spectrum in Electronic Supplementary Material S7.

### Accurate mass spectrometry and LC-ESI-QTOF assays

The accurate mass of metabolite fragments was obtained by recording a high resolution product ion spectrum of the corresponding metabolites using an ESI-QTOF (TripleTOF 6600, ABSciex, Darmstadt, Germany) equipped with DuoSpray ion source. Direct infusion was performed as for the QqQ spectra. The data acquisition and peak annotation were performed using manufacturer software (Peakview 2.1, ABSciex). We acquired QTOF mass spectra for all considered precursor ions. CAD was nitrogen at 4 psi and the CE-ramp was recorded with optimal DP-setting from section “LC-ESI-QqQ and MRM assays”.

To acquire the product ion spectra of mass isotopomers of enzymatically generated Mal, the QTOF MS was coupled to an Agilent 1260 Infinity chromatography system (Agilent Technologies, Waldbronn, Germany). Samples were diluted with acetonitrile to a concentration of 60% acetonitrile (*v*/*v*) and separated using the chromatographic method described in Teleki et al. [24]. During the elution, the product ion spectra of all mass isotopomers of the [M-H]^−^ ions Mal were recorded at a fixed CE of −16 eV.

The enzymatic assays to generate selectively labeled Cit, Icit, and cis-Aco were separated using the HPLC column described in the study of Teleki et al. [24] with the following eluent profile at a flowrate of 100 μl/min: 0 min, 10% A; 2 min, 10% A; 26 min, 38% A; 50 min, 58% A; 50.05 min, 90% A; 60 min, 90% A; 80 min, 10% A, followed by 15 min equilibration at 10% A at a flow of 200 μl/min. Buffers were A:10 mM (NH_4_)CH_3_COO, pH 9.2; B:acetonitrile. During the elution, the Q1 windows corresponding to mass isotopomers of Cit (CE −20 eV, CE spread 10 eV, and optimal DP of −15 eV) and Aco (CE −15 eV, CE spread 10 eV, and DP −60 eV) were monitored. The same HPLC method was used to separate the assays for production of [*hydroxy*-^18^O]-isotopes of Cit and Icit.

## Results and discussion

### Mixed elemental composition of nominal LC-ESI-MS/MS mass peaks

In our study, we focused on collisional fragments of central carbon metabolism intermediates, nucleoside phosphates, and amino acids which exceeded a threshold of 3% intensity relative to the most intense product ion (including the quasi-molecular ion) in a product ion spectrum recorded as described above. We provide each spectrum in Electronic Supplementary Material S7. The intensity criterion notwithstanding, we included all fragments of the study of Rühl et al. [9], for which we could find an accurate mass. We furthermore included fragments that do not satisfy the intensity criterion, but closely neighbor an intense fragment on the *m/z* axis which may generate overlapping mass traces in an MRM assay depending on the elemental composition of the neighboring fragments. All considered fragments are listed in Electronic Supplementary Material S1. We used accurate mass entries from the MassBank server [41], complemented with own experimental accurate mass measurements (see above) to determine the elemental composition of each product ion from all admissible elemental combinations. For details, the reader is referred to Electronic Supplementary Material S9.

**Fig. 1:**
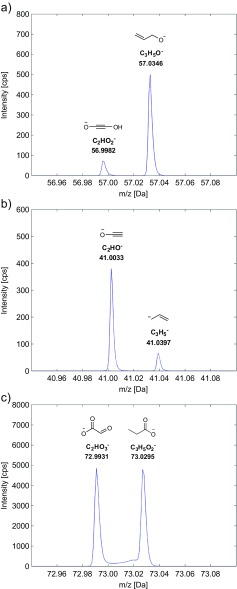
Accurate mass peaks of the *m/z* 57 of 6PG (**a**), *m/z* 41 of Glu (**b**), and *m/z* 73 of Icit (**c**) in negative ionization mode. The product ion spectra were recorded in a CE range from −50 to −5 eV

Overall, the elemental composition determined by this approach matched the determined elemental composition proposed in Rühl et al. [9] for the vast majority of fragments. We found a unique elemental composition for all fragments of which the number of carbon atoms could not be determined in Rühl et al. Noteworthy, we uncovered discrepancies to the proposed number of carbon for several QqQ mass peaks, which are highlighted in gray in Electronic Supplementary Material S1 along with those discrepancies concerning carbon origin (see below). We discovered that four of these QqQ peaks with discordant elemental composition are indeed composed of two distinct accurate mass peaks, which can only be differentiated by accurate mass spectrometry. We reviewed all studied fragments for multiple accurate mass peaks and highlighted each such instance in orange in Electronic Supplementary Material S1. Figure 1 exemplarily depicts the corresponding peaks from the QTOF mass spectra of 6PG, Glu, and Icit along with proposed structures for each pair of fragments. It can be clearly seen that no signal is negligible in comparison to the other, such that a QqQ MRM protocol measuring the mass transitions of one of these fragments would actually measure a superposition of two different carbon backbones. The study of Alves et al. [18] used the *m/z* 41 ion the [M-H]^−^ ion of Glu, shown in Fig. 1b, for ^13^C-isotopomer profiling. The carbon identity the authors refer to is the one of the *m/z* = 41.0033 fragment. The effect on the accuracy of measured fractional abundance cannot be assessed since no direct comparison of NMR multiplet fractions of Glu and measured LC-ESI-MS/MS enrichments in Glu is shown.

### Systematic identification of isotopic interferences in metabolite spectra

We used the elemental composition of fragments determined in Section “Mixed elemental composition of nominal LC-ESI-MS/MS mass peaks” to algorithmically compute the mass traces corresponding to carbon labeling states of each fragment with monoisotopic masses of the elements and carbon-13 [42], accounting for the electron mass. With product ion detection at unit mass resolution, the labeling states of neighboring fragments of one metabolite will or will not, depending on the elemental composition, overlap. Such isotopic interferences between labeling states of neighboring fragments can hardly be avoided since the CE parameter can almost never be tuned to solely generate the fragment in question. Electronic Supplementary Material S2 lists all fragments which suffer from a nominal overlap between tandem mass isotopomers of neighboring fragments and reveals that almost 50% of all studied fragments (intense and non intense fragments) are affected. Only tandem mass isotopomer detection using high resolution MS instrumentation provides the necessary selectivity to discern tandem mass isotopomers of neighboring fragments of equal nominal mass. The needed mass accuracy to achieve this is determined by the smallest mass difference between any two exact mass traces of equal nominal Q1 and Q3 mass. Therefore, for every such nominal overlap on Q1 and Q3, we computed the exact mass difference between the corresponding mass peaks. The resulting distribution of the mass difference over all such pairs has a median mass difference of 0.0089 Da, whereas its minimum of 0.000264 Da would allow for a maximal mass extraction window width of ±0.0001. Since an optimal calibration for a state-of-the-art QTOF device would result in 0.0001 mass difference between computed and measured masses, this overlap cannot be realistically expected to be resolved. Assuming an error of 0.0004 Da, a total extraction width of 0.001 Da is warranted. Using this best-case mass extraction width, only 2% of the isotopic interferences would not be resolved. Therefore, high resolution mass spectrometry for product ion detection will prove indispensable to harness the maximal amount of positional ^13^C-labeling information from CID collisional fragments. Apart from QTOF MS technology, QOrbitrap MS platforms may serve the same purpose. The latter instrument platform was shown to be comparable in terms of quantification accuracy to conventional QqQ technology [43, 44], whereas the former one is routinely used in quantitative proteomics [45].

### Structural assignment of product ion peaks

In many cases, the structure of the product ion alone suffices to deduce its carbon ancestry when orientation-providing heteroatoms like nitrogen and phosphorous or functional groups are present in the fragment ion. Therefore, the structural annotation of product ions is a pivotal step in deducing the positional origin of the product ions carbon atoms. For the structural annotation of product ion peaks, our sources of evidence were threefold:

1. We performed a structural assignment to mass peaks of known elemental composition by assuming a linear substructure of the precursor ion consistent with the elemental composition (we report all structures in Electronic Supplementary Material S7). Where possible, we included already published fragment structures from the studies of Dookeran et al., Rogalewicz et al., and El Aribi et al. [33, 46, 47]. For many fragments, alternative carbon bond fissures of the precursor ion yield the same fragment structure, resulting in several admissible substructures for one elemental composition. In this case, we exhaustively enumerated all alternative structures if they resulted in alternate carbon origins (differentiated by the keyword “OR” in Electronic Supplementary Material S7). Alternative carbon origins may also arise from intramolecular cyclization reactions since these reactions establish a new bond not present in the precursor ion. When chemically plausible, we allowed fragment ion structures to be derived from intermediates which correspond to intramolecular rearrangement products of the precursor ion. As possible rearrangement reactions, we considered the intramolecular formation of (1) anhydrides, (2) esters (lactones), (3) imides, and (4) amides. These cyclic intermediates establish a carbon origin not apparent from the precursor ion. In many cases, the elemental annotation of product ion spectra yielded direct experimental evidence of the presence of intermediates stemming from a rearrangement reaction. One striking example of this class represents the *m/z* 41.9985 (CON^−^) ion of Asp in negative ionization mode (see Scheme S-4 in Electronic Supplementary Material S11). From the mass spectrum of [1-^13^C]Asp and [4-^13^C]Asp, it becomes apparent that the carbon atom originates from positions 1 and 4 concurrently (see Electronic Supplementary Material S7). It appears that this ion is derived from a cyclic intermediate structure, the maleimide anion, apparent at *m/z* = 96.0091 peak in the mass spectrum (C_4_H_2_NO_2_^−^) of Asp, establishing a link between the α-nitrogen atom and C4 or C1 of Asp. Indeed, the mass spectrum of the [M-H]^−^ ion of maleimide exhibits the *m/z* 41.9985 ion as sole intense peak. Analogously, the elemental composition of C_2_H_3_OS^+^ for the *m/z* = 74.9899 fragment of the [M + H]^+^ ion of methionine clearly exposes this fragment as product of a rearrangement since oxygen and sulfur are connected through two carbon atoms. The mass spectrum of [5-^13^C]methionine indicates the retention of the S-methyl group, such that it appears likely to be derived of a cyclic intermediate at *m/z* = 105.0369 through elimination of ethane (see Electronic Supplementary Material S7). This intermediate, in turn, can be derived through carbon monoxide elimination from the cyclic intermediate at *m/z* = 133.0318 proposed by Rogalewicz et al. [33]. We used these clues as guidance to the structural annotation of higher mass fragments.
2. In some cases, insight into the fragmentation mechanism enabled the elucidation of the carbon origin. For instance, Eichinger et al. [48] reported on the fragmentation mechanism of pyruvate derivatives involving a CO-elimination from the C1 of the pyruvate backbone. As it turns out, this mechanism allows to assign a structure to all fragment ions derived from α-ketoacids by CO-elimination (see fragmentation scheme of αKG in Scheme S-5 in Electronic Supplementary Material S11). We furthermore recurred to known mechanisms for dicarbonic acids [49] and α-hydroxyl acids [50] to aid the structural annotation of OAA, Asp, and Mal.
3. The third source of evidence for the structural interpretation of product ions consisted in the use of selectively labeled ^13^C-standards which helped to delineate isobaric fragment structures for one elemental composition.

### Mixed carbon origin for fragments of sugar phosphates

To elucidate the carbon origin of selected fragment ions whose carbon origin was not apparent from the structural annotation, we used selectively labeled standards. From the group of sugar phosphates we investigated fragments from F6P, G6P, 6PG, and FBP in negative ionization mode. Such isotopic standards are commercially not available or prohibitively expensive. Therefore, we generated such standards from less expensive ^13^C-labeled standards by means of in vitro enzymatic assays, which we separated using LC. During the elution, the mass transitions corresponding to labeling states of certain fragment ions were acquired by means of an MRM assay according to section “LC-ESI-QqQ and MRM assays.” If just one fragmentation reaction was to contribute to the intensity of a given fragment peak, only a single mass trace should bear the total signal intensity (up to isotopic purity) when clearly defined isotopic standards are used. The identity of the mass trace for different isotopic standards on which the total intensity appears allows to deduce the identity of the fragment. When the isotopic purity of such standards is known, the expected intensity distribution across the mass traces can be computed, analogously to the computation of the natural isotopic abundance. This computation assumes knowledge of the neutral loss such that a carbon identity (reflecting itself in the retention or loss of the ^13^C-label) has to be assumed for each fragment. Comparison of the theoretical distribution to the experimental data allows to assess the correctness of the assumed carbon origin. We algorithmically computed the expected signal intensity for selectively labeled standards using 0.99 as atomic enrichment for the ^13^C-label and the natural isotopic abundances given in van Winden et al. [51]. The implementation is provided in the form of a zipped MATLAB m-File as Electronic Supplementary Material S8. For naturally labeled species, this implementation yields the same result as the software from Ramaley et al. [52] for the computation of natural isotopic abundance for MS/MS data.

Table 1 lists the result for investigated fragments of sugar phosphates. All signals were acquired with a setting for DP, CE, and CXP optimizing the signal intensity for the product ion in question. We use notation from Choi and Antoniewicz [3] to denote mass isotopomers and tandem mass isotopomers. For each labeled species of the investigated metabolites, the experimentally measured fractional abundance (FA) in the studied fragment is given in boldface type. Each value is accompanied by the theoretical isotopic distribution in italic font. This represents the expected FA which would arise if the assumed carbon origin indicated after the fragment mass was true. The mean standard deviation in FA over replicate injections was 0.01, with a maximum of 0.03. Most importantly, replication is afforded by using different isotopomers of each metabolite. For the *m/z* 129 (C_5_H_5_O_4_^−^) fragment of 6PG, almost all signal intensity appears on M1 > m1 for [1-^13^C]6PG as well as [2-^13^C]6PG and on M1 > m0 for [6-^13^C]6PG. Since the measured fractional abundance of each mass trace for all isotopic species agrees well with the expected distribution if this fragment was derived from carbon atoms [1–5], the carbon origin of this fragment can be clearly established. The deduced carbon origin suggests a product ion arising from a single carbon bond break. Any alternative carbon origin would necessitate a product ion structure that requires two bond breaks, therefore being energetically unfavorable. A quite different picture emerges for the *m/z* 101 ion of F6P (C_4_H_5_O_3_^−^): All isotopomers consistently show that the fragment is derived from positions [1–4] and [3–6] concurrently, although the latter product ion involves two bond breaks assuming a linear precursor ion structure. Heuristic fragmentation rules, favoring parsimonious reactions, and considerations based on standard bond energies would predict the carbon origin [1–4] to be unique. These results show that energetically unfavorable fragmentation routes may still be kinetically competitive. For the *m/z* 169 (C_3_H_6_O_6_P^−^) of F6P, the well-established and unique carbon origin from carbon atoms [4–6] is confirmed by the labeling pattern of Table 1. Since F6P and G6P always fragmented identically on the investigated product ions, only F6P is shown such that these results hold equally true for G6P. A unique origin also holds true for the *m/z* 139 and *m/z* 169 ions of FBP, although two potential fragmentation routes lead to these product ions due to two distal phosphate groups. Using our isotopic standards, we also deduced a mixed carbon ancestry for the *m/z* = 151 and *m/z* = 249 peaks of FBP and the *m/z* = 85 fragment of G6P (data not shown).

**Table 1:** Relative peak areas of individual mass traces for selectively ^13^C-labeled 6PG, F6P, and FBP

	M0	M1		M2		
	m0	m0	m1	m0	m1	m2
Labeled species	Relative signal intensity					
6PG	*m*/z = 129 (assumed origin: [1–5])					
^12^C	**0.90** (*0.91*)	**0.02** (*0.01*)	**0.05** (*0.05*)	**0.01** (*0.01*)	**0.00** (*0.00*)	**0.01** (*0.01*)
1-^13^C	**0.00** (*0.01*)	**0.01** (*0.00*)	**0.93** (*0.91*)	**0.00** (*0.00*)	**0.01** (*0.01*)	**0.05** (*0.04*)
2-^13^C	**0.01** (*0.01*)	**0.01** (*0.00*)	**0.93** (*0.91*)	**0.00** (*0.00*)	**0.01** (*0.01*)	**0.04** (*0.04*)
1,2-^13^C	**0.00** (*0.00*)	**0.00** (*0.00*)	**0.02** (*0.02*)	**0.00** (*0.00*)	**0.01** (*0.00*)	**0.97** (*0.92*)
6-^13^C	**0.06** (*0.01*)	**0.88** (*0.91*)	**0.00** (*0.00*)	**0.00** (*0.00*)	**0.05** (*0.05*)	**0.00** (*0.00*)
F6P	*m*/*z* = 101 (assumed origin: [3–6])					
^12^C	**0.93** (*0.92*)	**0.02** (*0.02*)	**0.04** (*0.04*)	**0.01** (*0.01*)	**0.00** (*0.00*)	**0.00** (*0.01*)
1-^13^C	**0.04** (*0.01*)	**0.67** (*0.92*)	**0.24** (*0.00*)	**0.01** (*0.01*)	**0.04** (*0.04*)	**0.01** (*0.00*)
2-^13^C	**0.05** (*0.01*)	**0.62** (*0.92*)	**0.28** (*0.00*)	**0.01** (*0.01*)	**0.03** (*0.04*)	**0.01** (*0.00*)
1,2-^13^C	**0.01** (*0.00*)	**0.04** (*0.02*)	**0.01** (*0.00*)	**0.65** (*0.92*)	**0.05** (*0.00*)	**0.24** (*0.00*)
6-^13^C	**0.03** (*0.01*)	**0.29** (*0.00*)	**0.62** (*0.92*)	**0.00** (*0.00*)	**0.03** (*0.02*)	**0.02** (*0.03*)
F6P	*m*/*z* = 169 (assumed origin: [4–6])					
^12^C	**0.92** (*0.92*)	**0.03** (*0.03*)	**0.03** (*0.03*)	**0.01** (*0.01*)	**0.00** (*0.00*)	**0.01** (*0.01*)
1-^13^C	**0.05** (*0.01*)	**0.89** (*0.92*)	**0.00** (*0.00*)	**0.02** (*0.02*)	**0.03** (*0.03*)	**0.00** (*0.00*)
2-^13^C	**0.07** (*0.01*)	**0.87** (*0.92*)	**0.00** (*0.00*)	**0.02** (*0.02*)	**0.03** (*0.03*)	**0.00** (*0.00*)
1,2-^13^C	**0.01** (*0.00*)	**0.07** (*0.02*)	**0.00** (*0.00*)	**0.92** (*0.92*)	**0.00** (*0.00*)	**0.00** (*0.00*)
6-^13^C	**0.01** (*0.01*)	**0.01** (*0.00*)	**0.92** (*0.92*)	**0.00** (*0.00*)	**0.03** (*0.03*)	**0.02** (*0.02*)
FBP	*m*/*z* = 139 (assumed origin: [5, 6])					
^12^C	**0.91** (*0.91*)	**0.05** (*0.04*)	**0.02** (*0.02*)	**0.01** (*0.01*)	**0.00** (*0.00*)	**0.01** (*0.01*)
1-^13^C	**0.06** (*0.01*)	**0.87** (*0.91*)	**0.01** (*0.00*)	**0.03** (*0.03*)	**0.02** (*0.02*)	**0.00** (*0.00*)
2-^13^C	**0.07** (*0.01*)	**0.86** (*0.91*)	**0.01** (*0.00*)	**0.03** (*0.03*)	**0.02** (*0.02*)	**0.00** (*0.00*)
6-^13^C	**0.01** (*0.01*)	**0.03** (*0.00*)	**0.90** (*0.91*)	**0.00** (*0.00*)	**0.04** (*0.04*)	**0.01** (*0.01*)
FBP	*m*/*z* = 169 (assumed origin: [4–6])					
^12^C	**0.91** (*0.91*)	**0.04** (*0.03*)	**0.04** (*0.03*)	**0.01** (*0.01*)	**0.00** (*0.00*)	**0.00** (*0.01*)
1-^13^C	**0.06** (*0.01*)	**0.89** (*0.91*)	**0.04** (*0.00*)	**0.00** (*0.02*)	**0.00** (*0.03*)	**0.00** (*0.00*)
2-^13^C	**0.07** (*0.01*)	**0.89** (*0.91*)	**0.04** (*0.00*)	**0.00** (*0.02*)	**0.00** (*0.03*)	**0.00** (*0.00*)
6-^13^C	**0.02** (*0.01*)	**0.05** (*0.00*)	**0.92** (*0.91*)	**0.02** (*0.00*)	**0.00** (*0.03*)	**0.00** *(0.02*)

M0, M1, and M2 signify that the quadrupole 1 was set to the mass of the M0, M1, and M2 mass isotopomer of the metabolite in question. m1, m2, and m3 denote that the quadrupole 3 was set to the mass of the m0, m1 and m2 tandem mass isotopomer of the fragment in question. The arising precursor-daughter mass transitions can be found in Electronic Supplementary Material S3. Bold face: measured experimental relative mass trace intensity, italic face: theoretical fractional abundance arising from the assumed carbon origin of the fragment indicated after the fragment mass. The theoretical abundance was calculated according to the method provided in Electronic Supplementary Material S8

### Mixed carbon origin for fragments of dicarbonic acids

Fragments of di-and tricarboxylic acids corresponding to a neutral loss of CO_2_ are another representative of the class of fragments structures for which alternative carbon origins exists. Neither fragmentation rules nor combinatorial fragmentation based on standard bond energies allow to infer the carbon ancestry from the mother molecule since for each such a fragment a carbon-carbon bond is broken leading to equal scores for alternative structures [29].

The relative peak areas of different mass traces of the MRM assay for OAA, Mal, αKG, Glu, and Asp are given in Table 2. Again, all signals were acquired with a setting for DP, CE, and CXP optimizing the signal intensity for the product ion in question. The mean standard deviation over replicate injections was 0.01 and is therefore omitted. For the *m/z* 87 fragment of OAA, the total signal intensity of [1-^13^C]OAA appears on M1 > m1 which suggests that C4-decarboxylation is the only CID fragmentation taking place under the parameters tested. This is not a surprising result, given the well-known susceptibility of β-keto acids in liquid solution to decarboxylation which proceeds through a six-membered transition state. Obviously, the same mechanism appears to favor C4-decarboxylation in the gas phase, although the C1-C2 carbon bond is more labile. Thus, the favorability of the transition state makes a rearrangement reaction dominant in comparison to the single cleavage of the more labile bond. The break of the most labile bond is one of many heuristic fragmentation rules commonly employed for structural annotation, which led McCloskey et al. [17] to erroneously assume C1-decarboxylation of oxaloacetate, resulting in an inaccurate carbon origin.

**Table 2:** Relative peak areas of selected mass traces for selectively ^13^C-labeled OAA, Mal, αKG, Glu, and Asp

	M0	M1		M2	
	m0	m0	m1	m1	m2
Labeled species	Relative signal intensity				
OAA	*m*/*z* = 87 (assumed origin: [1–3])				
^12^C	**0.94** (*0.95*)	**0.01** (*0.01*)	**0.03** (*0.03*)	**0.00** (*0.00*)	**0.01** (*0.01*)
1-^13^C	**0.01** (*0.01*)	**0.01** (*0.00*)	**0.94** (*0.95*)	**0.01** *(0.01*)	**0.03** (*0.02*)
4-^13^C	**0.03** (*0.01*)	**0.93** (*0.95*)	**0.00** (*0.00*)	**0.03** (*0.03*)	**0.00** (*0.00*)
1,4-^13^C	**0.00** (*0.00*)	**0.01** (*0.01*)	**0.04** (*0.01*)	**0.93** (*0.95*)	**0.00** (*0.00*)
Mal	*m*/*z* = 71 (assumed origin: [1–3])				
^12^C	**0.97** (*0.95*)	**0.01** (*0.01*)	**0.02** (*0.03*)	**0.00** (*0.00*)	**0.00** (*0.00*)
1-^13^C	**0.01** (*0.01*)	**0.32** (*0.00*)	**0.62** (*0.95*)	**0.02** (*0.01*)	**0.01** (*0.02*)
4-^13^C	**0.07** (*0.01*)	**0.57** (*0.95*)	**0.32** (*0.00*)	**0.02** (*0.03*)	**0.01** (*0.00*)
1,4-^13^C	**0.00** (*0.00*)	**0.02** (*0.01*)	**0.03** (*0.01*)	**0.92** (*0.95*)	**0.00** (*0.00*)
3-^13^C	**0.02** (*0.01*)	**0.01** (*0.00*)	**0.94** (*0.95*)	**0.01** (*0.01*)	**0.02** (*0.02*)
Mal	*m*/*z* = 73 (assumed origin: [1, 2])				
^12^C	**0.94** (*0.95*)	**0.02** (*0.02*)	**0.02** (*0.02*)	**0.00** (*0.00*)	**0.01** (*0.01*)
1-^13^C	**0.01** (*0.01*)	**0.02** (*0.00*)	**0.92** (*0.95*)	**0.02** (*0.02*)	**0.02** (*0.01*)
4-^13^C	**0.05** (*0.01*)	**0.92** (*0.95*)	**0.00** (*0.00*)	**0.02** (*0.02*)	**0.00** (*0.00*)
1,4-^13^C	**0.00** (*0.00*)	**0.01** (*0.01*)	**0.05** (*0.01*)	**0.92** (*0.95*)	**0.00** (*0.00*)
3-^13^C	**0.02** (*0.01*)	**0.95** (*0.95*)	**0.00** (*0.00*)	**0.02** (*0.02*)	**0.00** (*0.00*)
αKG	*m*/*z* = 101 (assumed origin: [1–4])				
^12^C	**0.99** (*0.94*)	**0.01** (*0.01*)	**0.00** (*0.04*)	**0.00** (*0.00*)	**0.00** (*0.00*)
1-^13^C	**0.00** (*0.01*)	**0.18** (*0.00*)	**0.82** (*0.94*)	**0.00** (*0.01*)	**0.00** (*0.03*)
Glu	*m*/*z* = 74 (assumed origin: [1, 2])				
^12^C	**0.94** (*0.93*)	**0.03** (*0.03*)	**0.03** (*0.02*)	**0.00** (*0.00*)	**0.00** (*0.00*)
1-^13^C	**0.00** (*0.01*)	**0.01** (*0.00*)	**0.94** (*0.93*)	**0.03** (*0.03*)	**0.02** (*0.01*)
Glu	*m*/*z* = 102 (assumed origin: [1–4])				
^12^C	**0.92** (*0.93*)	**0.01** (*0.01*)	**0.05** (*0.05*)	**0.00** (*0.00*)	**0.00** (*0.00*)
1-^13^C	**0.03** (*0.01*)	**0.09** (*0.00*)	**0.82** (*0.94*)	**0.02** (*0.01*)	**0.04** (*0.04*)
Asp	*m*/*z* = 88 (assumed origin: [1–3])				
^12^C	**0.94** (*0.94*)	**0.01** (*0.01*)	**0.04** (*0.04*)	**0.00** (*0.00*)	**0.00** (*0.00*)
1-^13^C	**0.01** (*0.01*)	**0.07** (*0.00*)	**0.88** (*0.95*)	**0.01** (*0.01*)	**0.02** (*0.03*)

M0, M1, and M2 signify that the quadrupole 1 was set to the mass of the M0, M1, and M2 mass isotopomer of the metabolite in question. m1, m2, and m3 denote that the quadrupole 3 was set to the mass of the m0, m1 and m2 tandem mass isotopomer of the fragment in question. The arising precursor-daughter mass transitions can be found in Electronic Supplementary Material S3. Bold face: measured experimental relative mass trace intensity, italic face: theoretical FA arising from the assumed carbon origin of the fragment indicated after the fragment mass. The theoretical abundance was calculated according to the method provided in Electronic Supplementary Material S8

In the case of the *m/z* 71 fragment of Mal, the data suggest that two fragmentation reactions take place simultaneously, one generating a 2,3,4-carbon-fragment and the other a 1,2,3-carbon-fragment. The *m/z* 73 fragment of Mal is composed of two carbon atoms, as revealed by the elemental annotation (see Electronic Supplementary Material S1), which originate from carbon atom positions [1, 2] since [3-^13^C]Mal appears on the M1 > m0 trace. Although chemically similar to OAA, αKG appears to undergo a C1- and C5-decarboxylation simultaneously. The CE energy setting generating this result is the one maximizing the signal intensity of the *m/z* 101 fragment. We investigated whether this result holds over the entire domain of CE settings. Figure S-1 in Electronic Supplementary Material S10 shows that each of the fragmentation reactions corresponding to a C1- or C5-decarboxylation has a different energy threshold and a different CE dependence of its signal intensity, suggesting that both reactions indeed yield chemically different product ions. Over the entire domain of CE, a mixture of both species contribute to the *m/z* 101 peak.

Experimentally, the production of isotopic pure [4-^13^C]Mal and [4-^13^C]OAA proved difficult, evidenced by the higher relative peak areas on mass traces corresponding to M + 1 for doubly labeled species and M + 0 for singly labeled species comprising a ^13^C-label in C4. This effect is most likely due to the isotopic dilution of H[^13^C]O_3_^−^ in the enzymatic assay resulting from the solution of atmospheric carbon dioxide in the distinct assay components. However, at no point in the running discussion does this effect compromise the conclusions drawn from the data.

For Glu as well as Asp, the main signal intensity is recorded on the M1 > m1 trace for the [M-H-44] ^−^ fragments. This observation is in conflict with the heuristically determined carbon origin in the studies of Rühl et al. [9], but agrees with experimental study of Grossert et al. [53], who reported the retention of 1-^13^C-label in all product ions of Glu and Asp in negative ionization mode. However, the contribution to the M1 > m0 trace is higher than the expected intensity due to natural isotopic abundance and isotopic impurity such that both C1 and C4 or C5 neutral losses appear to occur simultaneously. This result inherently depends on the ionization mode because Glu and Asp in positive ionization mode undergo exclusively a C1-decarboxylation due to the high stability of the immonium ion generated upon CO and H_2_O loss [54].

Through the use of ^13^C-isotopic standards, we furthermore deciphered a mixed carbon ancestry for *m/z* = 41, *m/z* = 71, and *m/z* = 86 peaks of Asp, the *m/z* = 84 and *m/z* = 85 ions of Glu, and for the *m/z* = 54, 56, 68, 69, 70 ions of lysine. For Cit, we established that each fragment ion has a symmetrical mixed carbon origin except for product ions which are symmetric around the prochiral C3 carbon atom (see below). For Icit, it holds that the vast majority of fragments is of mixed carbon ancestry as are the *m/z* = 85 and *m/z* = 129 fragments of cis-Aco. We conclude that the competitive fragmentation reactions generating isobaric product ions of alternate carbon origin are a rather common phenomenon in the product ion spectra of central carbon metabolism. Therefore, we caution the use of parsimonious and heuristic rules for carbon identity annotation. We recommend taking into account all chemically admissible fragmentation routes to avoid misassigning carbon identity.

### Fragmentation pathway of malate

We complemented the experimental approaches hitherto applied by examining the product ion spectra of selected metabolites for generic fragmentation patterns published so far. One such pattern was deduced by Greene et al. [50] for *α*-hydroxyacids: The negative ions of these metabolites concomitantly yield a *m/z* 45 ion and a [M-H-46] ^−^ fragment, which the authors showed to correspond to complementary pairs of product ion and neutral loss upon loss of formic acid.

**Scheme 1:**
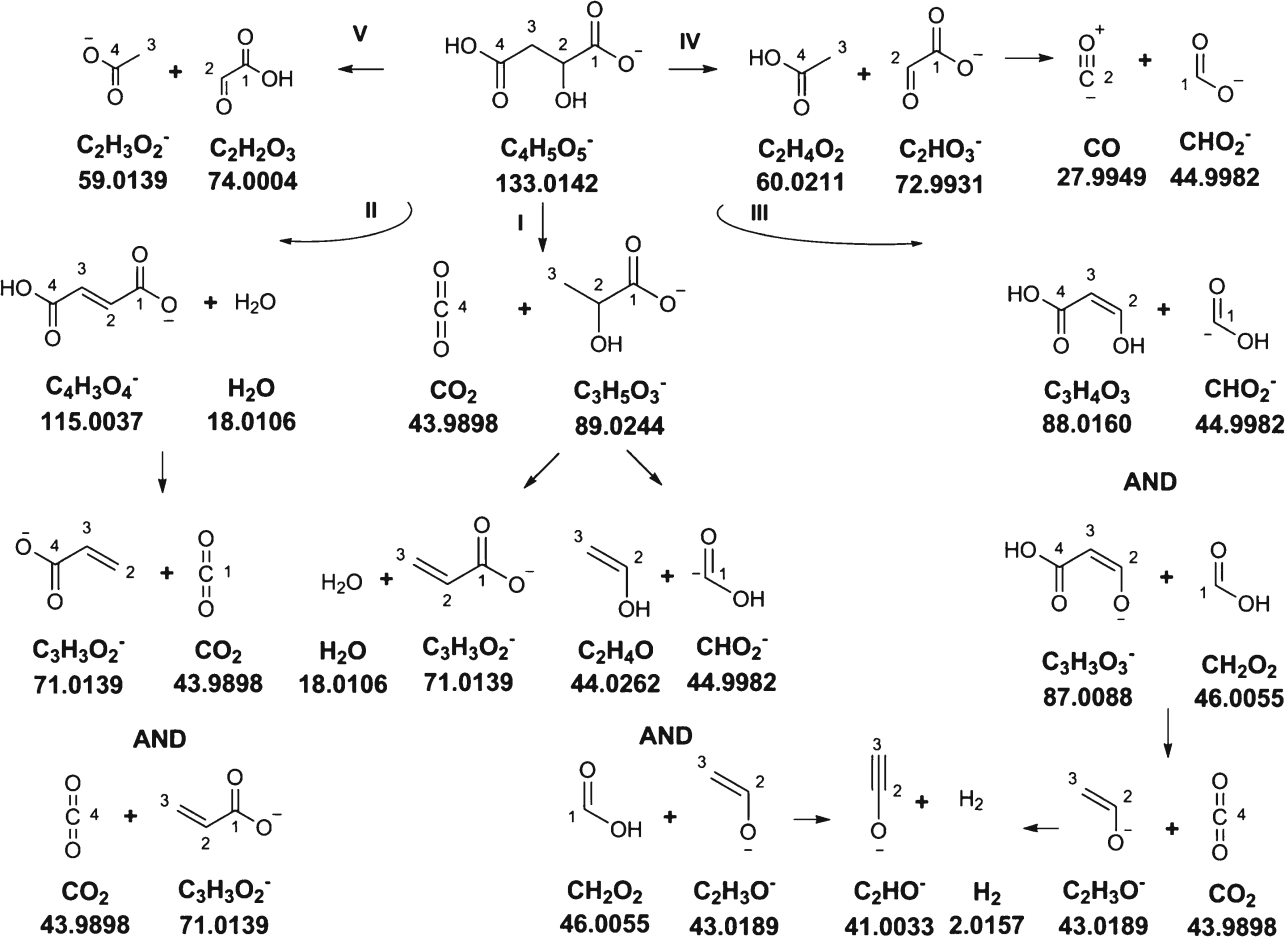
Fragmentation pathway of Mal explaining the apparent product ion spectrum in negative ionization mode. The product ion spectrum can be found in Electronic Supplementary Material S7. For each product ion and neutral loss, the chemical formula and exact mass are indicated. “AND” signifies that the presence of complementary product ion species of one fragmentation reaction has been confirmed either by them generating a peak in the product ion spectrum or by selectively labeled species

The product ion spectrum of Mal exhibits not only those two fragments, i.e. *m/z* 45 and *m/z* 87 (see pathway **III** in Scheme 1), but also those of lactic acid (*m/z* 43 and *m/z* 45). It appears that the lactic acid anion is generated from Mal through C4-decarboxylation, giving rise to fragmentation pathway **I** in Scheme 1. The *m/z* 43 fragment may further eliminate hydrogen at higher collision energies, yielding the *m/z* 41 ion. Further fragmentation routes comprise a McLafferty-type rearrangement with its archetypal product ion at *m/z* 59 (pathway **V**) [49] and the elimination of acetic acid (pathway **IV**). The latter one generates the glyoxylic acid anion. The spectrum of this ion, in turn, exhibits just one peak at *m/z* 45, corresponding to the formic acid anion. Since this anion is again derived from C1, the unique carbon origin of the *m/z* = 45 ion is preserved, although various reactions contribute to this peak. Pathway **II** proceeds via the elimination of water generating the symmetric fumaric acid anion. This symmetry explains the mixture of carbon ancestry of the *m/z* 71 fragment derived from this intermediate by decarboxylation (Table 2). However, since the fumarate anion is symmetric, an equal partition of the signal between M1 *>* m0 and M1 *>* m1 would be expected, which is apparently not the case. Due to the higher signal intensity on the M1 *>* m1 mass trace for [1-^13^C]Mal, there needs to be an additional labeled contribution to the *m/z* 71 peak. Elimination of water from the lactic acid anion, containing the [1-^13^C]-label, would account for this observation. Indeed, the *m/z* 71 ion appears in the product ion spectrum of lactate, the accurate mass of which matches C_3_H_3_O_2_^−^. This pathway is in agreement with the elemental composition determined above and the data presented in Tables 2 and 3 (see below). It explains the apparent product ion spectrum of Mal, which is given in the Electronic Supplementary Material S7.

**Table 3:**
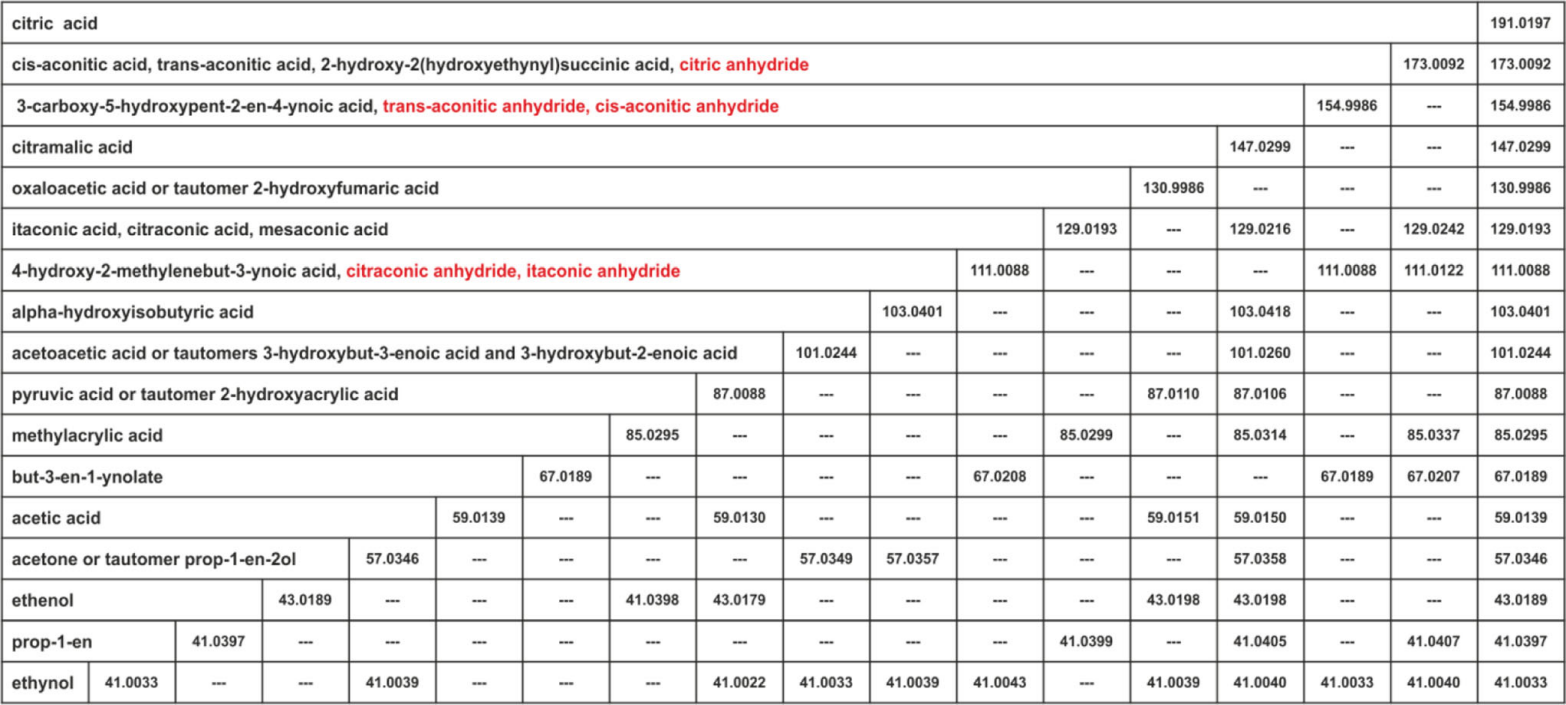
Schematic of the fragmentation pathway of Cit using structure names. In each column the fragment ions of the corresponding metabolite appear, ordered by mass. A presumed intermediate structure was assumed to be correct if it only generated product ions also present in the product ion spectrum of Cit itself. The fragments apparent in the product ion spectrum of Cit are listed in the rightmost column. In the leftmost column chemically plausible structures for each product ion Cit are given which are consistent with the labeling data of Electronic Supplementary Material S6. The red colored ones are excluded based on other evidence (see main text). The shown masses are experimental accurate masses from the respective injection.

In the case of Mal, it turns out that its product ion spectrum can be interpreted as a superposition of the fragmentation reactions of smaller molecules which, in turn, are derived from the [M-H]^−^ ion of Mal by simple one-step elimination reactions, i.e., decarboxylation, elimination of water, acetic acid, and formic acid. This observation prompted us to ask whether necessarily more complex spectra of larger molecules can also be understood in terms of more basic fragmentation reactions. For this purpose, we choose Cit as central molecule of the tricarboxylic acid cycle which is amenable to a multitude of fragmentation channels.

### Fragmentation pathway of citrate

As a first step, we used the assay of section “In vitro enzyme assays to generate selectively labeled ^13^C-species and ^18^O-species” to generate [1-^13^C]Cit, [2-^13^C]Cit, [3-^13^C]Cit, and [6-^13^C]Cit corresponding to unlabeled acetyl-CoA reacting with [4-^13^C]OAA and [3-^13^C]OAA, [2-^13^C]OAA, and [1-^13^C]OAA, respectively. We furthermore included [1,6-^13^C]Cit, [1,2-^13^C]Cit, and [1,3-^13^C]Cit to afford replication. Using the acquisition method described in section “Accurate mass spectrometry and LC-ESI-QTOF assays,” we acquired the labeling enrichment in each of the product ions of Cit for the above-mentioned selectively labeled species in technical triplicate. To aid our interpretation, we acquired the analogous data set for Icit as well as cis-Aco. The ^13^C-enrichment across all fragments of Cit, Icit, and cis-Aco for the above-mentioned isotopomers is shown in Electronic Supplementary Material S6 together with the standard deviation and expected signal intensity assuming a unique carbon origin. Tracking which carbon atom is retained and which carbon atom is lost, one is able to exclude many structures explaining a given elemental composition. For instance, the *m/z* = 87.0088 fragment of Cit (C_3_H_3_O_3_^−^) admits two chemically different fragments, one corresponding to 2-oxopropanoic acid (pyruvate) from carbon atoms [2, 3, 6] or [3, 4, 6], the other being 3-oxopropanoic acid (malonate semialdehyde) from carbon position [1-3] or [3-5]. Each structure admits two alternative carbon origins, necessitating the differentiation of four different carbon origins. The data in Electronic Supplementary Material S6 reveal that this fragment is derived symmetrically from carbon atoms [2, 3, 6] and [3, 4, 6]. Thus, its structure can be assumed to be the pyruvate anion. Analogously, given the labeling data, the space of possible structures for the remaining fragment ions 'can be restricted to the ones given in Table 3. For all compounds for which we could obtain authentic standards down to the mass of 85.0295, we recorded a QTOF product ion spectrum as described in section “Accurate mass spectrometry and LC-ESI-QTOF assays,” the mass peaks of which are listed in the column corresponding to the compound in question. Smaller molecules are unlikely to fragment at all or their fragmentation is inconsequential given that we restrict our analysis to the mass range from 40 to 191. The mass spectrum of Cit exhibits two product ions at *m/z* = 173.0092 and *m/z* = 154.9986, corresponding to the loss of one and two water molecules, respectively. For aliphatic hydroxylated compounds, the loss of water is conventionally assumed to originate from the hydroxyl group [50]. By this token, a racemic mixture of cis- and trans-Aco would contribute to the *m/z* = 173.0092 peak. For the second loss of water, only two processes could account: Either it is eliminated from a carboxyl group or an intracellular anhydride is formed. The latter process would generate cis-aconitic anhydride and trans-aconitic anhydride. However, the product ion spectrum of neither was concordant with that of Cit: Both compounds yield one fragment not apparent in the mass spectrum of Cit (*m/z* = 83.0139). We took this observation as evidence against their presence in the fragmentation sequence. Thus, the assumption that the second water molecule is eliminated from a carboxyl group seems compelling. Surprisingly, the ^18^O-enrichment in both fragments for [*hydroxy*-^18^O]Cit (shown in Electronic Supplementary Material S6) reveals that this reaction sequence accounts for a small share of signal intensity. Instead, the larger part of signal intensity is accounted for by the reverse sequence of initial water elimination from a carboxyl group with subsequent water loss arising from the hydroxyl moiety. The structure contributing to the *m/z* 173.0092 peak of Icit even fully retains the hydroxyl group. For the water elimination from carboxyl groups, a mechanism was proposed by Grossert et al. [53] in which a ynolate-ion is generated following the elimination of water. However, for these ions no standards are commercially available which is why a fragmentation pattern was assumed for the corresponding ions (see Scheme S-12, S-16 and S-19 in Electronic Supplementary Material S11).

Since no anhydride is formed from cis-Aco and trans-Aco in the fragmentation sequence of Cit, we judged anhydride formation in general to be unlikely to account for an additional water loss. By this token, citric anhydride, citraconic anhydride, and itaconic anhydride can be excluded as possible structures for the *m/z* = 173.0092 and *m/z* = 111.0088 peaks, respectively, although all of their product ions also appear in the mass spectrum of Cit. The *m/z* = 111.0088 ion does not appear in the mass spectrum of citraconate nor mesaconate (*m/z* = 129.0193), but first appears in the spectrum of cis-Aco, from which the neutral loss is -H_2_O and -CO_2_. Since a sequential loss of CO_2_ and H_2_O would proceed through citraconate, from which water is obviously not lost, the reverse mechanism of initial water loss with subsequent CO_2_ loss must hold true. This mechanism was shown to proceed through a combined loss of H_2_O and CO_2_ as carbonic acid for glutaric acid [55]. This explains why the *m*/*z* = 111.0088 ion is not generated from citraconate anion (*m/z* = 129), but rather from cis-Aco. The mechanism of glutaric acid is directly transferable to cis-Aco since it can be understood as 2-dehydrogenated 3-carboxy-glutaric acid.

Because the *m/z* 41.0033 peak appears in the mass spectra of many presumed intermediates, it is generated by a multitude of fragmentation channels. One such channel is the elimination of CO_2_ and H_2_ from Pyr (*m/z* = 87.0088). Pyr for its part is derived from the fragmentation of OAA which arises from Cit through elimination of CH_3_COOH. Analogously, beginning with the lowest mass fragments, one is able to recursively construct other fragmentation channels of Cit. The fragments appearing in no mass spectrum other than Cit itself are those corresponding to OAA (*m/z* 130.9986), citramalate (*m/z* 147.0299), and Aco and 2-hydroxy-2-(hydroxyethynyl)succinic acid (*m/z* 173.0092). These compounds arise from Cit through the elimination of acetic acid, CO_2_, and water. Thus, as in the case of Mal, the spectrum of Cit appears as superposition of the fragmentation routes of intermediates arising from these basic eliminations. The fragmentation sequence of the presumed intermediates of Cit is depicted in Electronic Supplementary Material S11. These fragmentation sequences also establish the reason as to the symmetrically mixed carbon origin of the majority of Cit fragments: The initial fragmentation reactions yield a symmetric mixture of citramalate (through CO_2_ loss from C1 and C5), OAA (through CH_3_COOH elimination from the pro-S arm and pro-R arm, respectively), and a racemic mixture of cis/trans aconitate as well as 1 and 5-dehydrated Cit (through water loss) since fragmentation reactions for each pair of fragments are chemically identical. In the following reactions, this symmetry is propagated to smaller fragments. Some fragmentation reactions let each of the two carbon isomers collapse onto one structure with unique carbon origin, centered on the prochiral C3 of Cit such that the only fragments with unique carbon origin are *m/z* 103, *m/z* 85, *m/z* 57, and *m/z* 41.0397. However, McCloskey et al. [17] assumed a unique carbon origin for fragments *m/z* 87, *m/z* 111, and *m/z* 147 which is apparently not the case given the data in Electronic Supplementary Material S6.

We argue that every mass peak of which the carbon ancestry is established by two fragmentation pathways proceeding through chemically identical intermediates will be of symmetrically mixed carbon ancestry.

Reactions proceeding through symmetric fragmentation intermediates are representative for this class of reactions. One such fragment is present in the mass spectrum of Icit: Upon elimination of glyoxylic acid, Icit generates the succinate anion apparent at *m/z* = 117.0193, analogously to the mechanism of glyoxylic acid elimination from Mal. Conspicuously, all ions from the product ion spectrum of succinate also appear in the spectrum of Icit, distinguishing it from Cit. Since decarboxylation from both terminal carboxyl groups of the succinate ion (originating from C5 and C6 of Icit) are chemically identical, the carbon origin of the corresponding peak (*m/z* = 73.0295) is indeed symmetrically mixed (see Electronic Supplementary Material S6).

In terms of biology, the diastereotopic hydrogen atoms cause the prochiral Cit molecule to react such that a unique carbon atom mapping exists from OAA and acetyl-CoA through Cit to isocitric acid. For this reason, each carbon atom has a unique positional assignment within the Cit molecule. In terms of mass spectrometry, the prochiral side chains of Cit fragment symmetrically, yielding a relative contribution of 0.5 for each of the two alternative carbon origins. Interestingly, as we show in the Electronic Supplementary Material S12, this symmetric mixture still contains as much information with regard to positional labeling enrichment as a unique carbon ancestry. This symmetric mixture enabled Alves et al. [18] to derive equations relating the mass fractions from the *m/z* 67 fragment of Cit to the flux ratio of pyruvate carboxylase and pyruvate dehydrogenase. This ratio could be determined from the Cit mass fractions alone, showing the still informative labeling pattern of symmetric mixed carbon ancestry.

We furthermore derived and validated fragmentation pathways of Pyr (Scheme S-2), Mal (Scheme S-3), Asp (Scheme S-4), *α*KG (Scheme S-5), OAA (Scheme S-6), and cis-Aco (Scheme S-7) in negative ionization mode, which are provided in S9 together with additional discussion. For fragments of Pyr and *α*KG corresponding to CO-elimination, our data are fully consistent with the mechanism of CO-elimination proposed by Eichinger et al. [48] for pyruvate derivatives. The mechanism proposed for Asp is consistent with the MS^3^ data from the study of Eckersley et al. [56]. The identity of each product ion in the fragmentation pathway was confirmed by recording a product ion spectrum of [1,2,3,4-^13^C]*α*KG, [1-^13^C]Pyr, [2-^13^C]Pyr, [3-^13^C]Pyr, [1-^13^C]Asp, and [4-^13^C]Asp, respectively, or by using our in-house produced ^13^C-isotopes. The product ion spectra of *α*KG, Pyr, Asp, Mal, ^13^C-labeled analogues, and pathway intermediates are shown in Electronic Supplementary Material S7.

### Complete isotopomer information for several metabolites

Choi and Antoniewicz [12] showed that by combining the TMID of several fragments the entire isotopomer distribution of the corresponding metabolite may be inferred. For this inference to be feasible, the matrix mapping the isotopomer vector onto the combined tandem mass isotopomer vectors of several fragments has to have full rank. A mathematical synopsis detailing this fact can be found in Electronic Supplementary Material S12. We algorithmically computed the rank of the mapping matrix for all metabolites assuming the TMID of all studied fragments with unique carbon ancestry to be known. For this purpose, we used algorithms inspired by Weitzel et al. [57].

The resulting rank is shown in Electronic Supplementary Material S2. Since all studied fragments with unique carbon origin are included, regardless of their intensity, this rank captures the maximal degree of information with regard to isotopomer resolution afforded by the carbon backbone fragmentations apparent in the respective MS/MS spectrum. The higher the rank, the more informative is the product ion spectrum with respect to full isotopomer resolution; a rank equal to 2^*n*^ where *n* is the number of carbon atoms in the metabolite signifies that full isotopomer resolution can be achieved.

For the running example of Mal, it turns out that the corresponding matrix has full rank. The same holds true for metabolites OAA, Asp, and glyoxylate in negative ionization mode and glycine, cysteine, and Asp in positive ionization mode. In case of Mal, however, Electronic Supplementary Material S2 also reveals that many mass transitions overlap. Therefore, only accurate mass spectrometry will enable to harvest the full information content contained in the CID product ion spectrum. To demonstrate this contention, we followed an LC-ESI-QTOF-based approach to acquire the entire product ion spectrum of mass isotopomers of Mal. For this purpose, we produced an equimolar mixture of isotopomers MAL_1000_, MAL_0100_, and MAL_0010_ using the pyruvate carboxylase assay described in the Experimental section. The resulting relative signal intensities are given in Table 4, whereas the M1 product ion spectrum of Mal and the ion chromatogram are given in Electronic Supplementary Material S13. Obviously, the fractions represent the expected signal partition according to how many of the labeled atoms are present in the corresponding fragment, yielding either 0.33, 0.66, or 1 (up to isotopic purity). The found accurate mass allows an unambiguous identification of different fragment mass isotopomers in all cases, demonstrating that both good quantification and mass accuracy can be achieved with QTOF MS technology.

**Table 4:** Relative signal intensities of tandem mass isotopomers of the M0, M1, and M2 mass isotopomers of Mal for an equimolar mixture of isotopomers Mal_1000_, Mal_0100_, and Mal_0010_

M0^a^		M1				M2					
m0^b^		m0		m1		m0		m1		m2	
*m*/*z* found^c^	Rel. int.^d^	*m*/*z* found	Rel. int.	*m*/*z* found	Rel. int.	*m*/*z* found	Rel. int.	*m*/*z* found	Rel. int.	*m*/*z* found	Rel. int.
41.0035	0.01	41.0034	0.36	42.0067	0.62	<LOD	–	42.0072	0.02	<LOD	–
43.0193	0.01	43.0192	0.33	44.0227	0.64	43.0192	0.00	44.0226	0.02	45.0262	0.00
44.9988	0.01	44.9984	0.63	46.0019	0.36	44.9987	0.00	46.0019	0.00	–	–
59.0146	0.03	59.0143	0.63	60.0178	0.30	59.0146	0.02	60.0180	0.02	61.0200	0.00
72.9941	0.01	72.9939	0.31	73.9973	0.65	72.9939	0.00	73.9972	0.02	75.0008	0.01
87.0099	0.01	87.0097	0.28	88.0129	0.68	–	–	88.0131	0.01	89.0167	0.01
89.0254	0.01	89.0254	0.01	90.0289	0.96	–	–	90.0288	0.01	91.0322	0.02
115.0049	0.01	–	–	116.0091	0.96	–	–	–	–	117.0119	0.03

^a^M0, M1, and M2 denote the mass isotopomers of Mal which correspond to the quadrupole mass filters Q1 = 133, Q2 = 134, and Q3 = 135, respectively

^b^m0, m1, and m2 denote the unlabeled, singly labeled and doubly labeled product ions, respectively

^c^The accurate masses are the measured accurate masses in this specific experiment

^d^The relative intensities were not corrected for natural isotopic abundance

### Statistical information gain of positionally resolved labeling information

Clearly, in the absence of systematic errors a greater number of measured quantities directly translates into improved statistical quality of derived quantities. The number of measured mass traces for an arbitrary TMID is given as (*n* − *m* + 1)(*m* + 1), where *n* is the number of carbon atoms in the precursor and *m* is the number of carbon atoms in the fragment [3]. Thus, for 0 < *m* < *n*, this quantity is always greater than *n* + 1, the number of measurements contained in a MID. On the other hand, it may be noted that the additional measurements in a TMID necessarily arise from a different mass spectrometric acquisition mode, i.e., MS/MS acquisition as opposed to MS acquisition sufficient for MID acquisition. Due to the loss of signal intensity inherent in MS/MS acquisition, the TMID may be less precise than the corresponding MID of the precursor ion in question. Therefore, a gain in statistical quality from positionally resolved labeling information is not guaranteed.

**Fig. 2:**
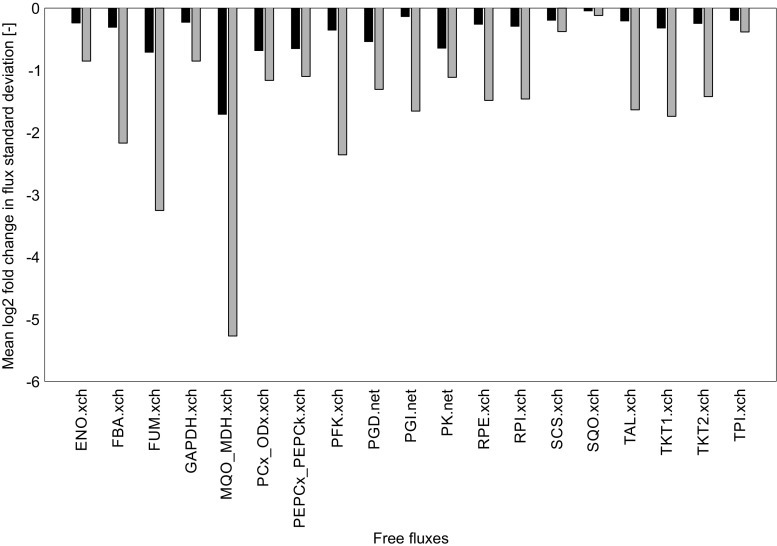
Relative flux standard deviations for all free fluxes in comparison to the measurement scenario described in Kappelmann et al. [58]. *Black bars* represent the measurement scenario of higher acquisition precision for MID than for TMID, whereas *gray bars* represent the scenario of equal measurement precision for MID and TMID. The flux nomenclature can be found in Kappelmann et al. [58]

To analyze the extent to which flux confidence may increase, we used the central metabolism network of *Corynebacterium glutamicum* Δ*malE* and the measurement scenario described by Kappelmann et al. [58]. This measurement configuration is taken as base scenario and encompasses mass isotopomer information for sugar phosphates and organic acids and the positional resolution afforded by the immonium ion of most amino acids in positive ionization mode and is given in Electronic Supplementary Material S4. For comparison, we extended this measurement model by positional labeling information from fragment ions of which the carbon identity was identified or validated in this study. The complete list of added labeling information is listed in Electronic Supplementary Material S5. For all simulations, we used 13CFLUX2 software suite [5]. Essentially, 1000 random label distributions were generated from the network, for each of which the associated flux standard deviations were evaluated for the two measurement scenarios. Figure 2 shows the mean log2 of the relative flux standard deviations computed from the extended measurement model in comparison to the one computed from the base scenario. This computation was once performed using an error of 0.01 for the MID and 0.05 for the TMID (black bars) and once assuming an equal measurement error of 0.01 for the MID and TMID (gray bars). For the latter scenario, it can be seen that an up to 20-fold increase in flux precision can be achieved for selected fluxes and that on average the standard deviation is half as high as in the base measurement scenario. Even for the worst case scenario of 0.05 as TMID precision, a significant information gain can be accomplished.

## Conclusions

We studied in detail the elemental composition as well as carbon origin of CID fragments from intermediates of primary metabolism, of which the isotopomer distribution is routinely experimentally quantified for use in flux estimation by means of ^13^C-MFA. The elucidated carbon fate in this study represents the most accurate and comprehensive account yet on this matter. We showed that nominal QqQ mass peaks can be composed of fragments of different elemental composition, compromising the measurement of the TMID in any of these fragments using QqQ technology with unit mass resolution. On the other hand, the signal intensity of some fragments with a uniquely defined elemental composition may draw from the contribution of two product ions with differing carbon origins, as shown by our specifically synthesized ^13^C-isotopic standards. These tracers also helped us expose that heuristic fragmentation rules are often inept to predict the true fragment structure. We conclude that the factors compromising the measurement of the TMID of a given fragment, namely non-unique carbon origin, non-unique elemental composition, and overlapping mass traces are rather pervasive within the CID spectra of central carbon metabolism intermediates. However, the latter two intricacies are certainly platform-dependent. With the help of a defined isotopomer mixture of Mal, we show that the problem of overlapping mass traces can be circumvented by using an ESI-QTOF device. The same approach will enable to differentiate different accurate mass peaks contributing to one integral mass peak. Therefore, QTOF MS technology will prove indispensable, if improved positionally resolved ^13^C-enrichment information from LC-ESI-CID product ions is desired.

## Electronic supplementary material

Below is the link to the electronic supplementary material.

ESM 1 (PDF 437 kb)

ESM 2 (PDF 5.43 mb)

ESM 3 (TAR 5.69 mb)
