# Supplementary material for: Comprehensive and accurate tracking of carbon origin of LC-tandem mass spectrometry collisional fragments for 13C-MFA
Source: Anal Bioanal Chem. 2017 Jan 23;409(9):2309–26. doi: 10.1007/s00216-016-0174-9 (PMC5477699; doi:10.1007/s00216-016-0174-9)
Supplement: Supplementary file 1 — (PDF 437 kb) [file 216_2016_174_MOESM1_ESM.pdf]

Analytical and Bioanalytical Chemistry

Electronic Supplementary Material

Comprehensive and accurate tracking of carbon origin of LC-tandem mass spectrometry collisional fragments for ^{13}C-MFA

Jannick Kappelmann, Bianca Klein, Petra Geilenkirchen, Stephan Noack

216_2016_174_MOESM2_ESM.pdf

216_2016_174_MOESM3_ESM.tar
