# Supplementary material for: Comprehensive and accurate tracking of carbon origin of LC-tandem mass spectrometry collisional fragments for 13C-MFA
Source: Anal Bioanal Chem. 2017 Jan 23;409(9):2309–26. doi: 10.1007/s00216-016-0174-9 (PMC5477699; doi:10.1007/s00216-016-0174-9)
Supplement: Supplementary file 2 — (PDF 5.43 mb) [file 216_2016_174_MOESM2_ESM.pdf]

Supplementary Information for

Comprehensive and Accurate Tracking of Carbon Origin of LC-Tandem Mass Spectrometry Collisional Fragments for ^{13}C-MFA

Jannick Kappelmann, Bianca Klein, Petra Geilenkirchen, and Stephan Noack

We provide Supplementary Information S1, S2, S3, S4 and S5 as one Excel file. S1 details the considered set of metabolites and the studied collisional fragments thereof. It also contains the exact mass annotation and determined elemental composition for each fragment. S2 lists the positional origin of fragment carbon atoms for product ions from a carbon backbone break if it is known and unique. It furthermore contains the rank of the tandem mass isotopomer measurement matrix and the mass transitions for each fragment. The measured mass transitions of the MRM assays are given in Supplementary Information S3. S4 details the base measurement scenario whereas S5 details the extended ^{13}C-labeling measurement system. S6 contains the citrate, isocitrate and aconitate labeling data and is a separate Excel-file. The metabolite mass spectra alongside fragment structures are provided as S7 in form of a separate pdf file. S8 is a zipped-MATLAB file for the computation of the isotopic distribution of selectively labeled standards.

Contents

S 9 Determination of the Elemental Composition of Product Ions S-2
S 10 Collision Energy Dependence of Mixed Carbon Ancestry S-4
S 11 Fragmentation Pathways of Selected Metabolites S-6
S 12 Exemplary Measurement Matrix for Tandem Mass Isotopomer Distributions S-27
S 13 Total Ion Chromatogram and Product Ion Spectrum of the M1 Mass Isotopomer of Malate S-32

Bibliography **S-33**

S 9 Determination of the Elemental Composition of Product Ions

Each fragment ion was annotated with an exact mass obtained either from the uploads of Keio University, RIKEN and Halle University to the MassBank database (Horai et al. (2010)) or from an experimentally generated ESI-QTOF product ion spectrum of the corresponding metabolite upon direct infusion or from both. All available data points are listed in S1. For each considered fragment all theoretically possible elemental compositions were computed by solving equation (1) over the space of integers using Mathematica (Wolfram-Research (2014)).

$$12x_1(C) + 1x_2(H) + 14x_3(N) + 16x_4(O) + 31x_5(P) + 32x_6(S) = \lfloor Q3 \rceil \quad (1)$$

Q3 in equation (1) denotes the fragment mass-to-charge ratio as integer number. As constraints the known elemental composition of the quasi-molecular ion $[M+H]^+$ for positive ionization or $[M-H]^-$ for negative ionization mode were applied to each variable. For sugar phosphates additionally $x_1 + x5 \geq 1$ was added as constraint, requiring the fragment to have a carbon or phosphorus backbone. For each solution to equation (1) the arising monoisotopic mass was calculated according to standard monoisotopic masses of the elements (Wang et al. (2012)) and compared with the exact mass annotation. In cases where several atomic compositions matched the exact mass chemically non-probable atomic combinations were excluded. Due to the large number of elemental combinations explaining the fragment masses of nucleoside mono-

and triphosphates (163 combinations for the *m/z* 159 fragment of guanosine 5-triphosphate alone) not all combinations according to equation (1) were computed for this class of metabolites. Instead, we logically deduced a possible structure for each intense fragment ion from the pseudo-molecular ion, computed its exact mass, and compared it to the exact mass annotation (see Supplementary Information S1). We provide structures for at least all intense product ions with additional discussion in Supplementary Information S7 and S8.

S 10 Collision Energy Dependence of Mixed Carbon Ancestry

Apparently, the CE energy setting optimizing total signal intensity of the m/z 101 fragment of αKG and the m/z 88 fragment of Asp induces two fragmentation reactions to occur simultaneously. To investigate the CE dependence of this phenomenon, we performed a collision energy ramp on a direct infusion of selectively labeled [1,2,3,4-^{13}C]αKG and [1-^{13}C]Asp and recorded the signal intensity on the mass transitions M4>m3/M4>m4 and M1>m1/M1>m0 for αKG and Asp, respectively. The resulting signal intensities and calculated relative share of C1-decarboxylation are depicted in Figure S-1. Apparently, with increasing CE the C1-decarboxylation in αKG is favored, the contribution of which actually never fades to zero, but is still present at -6 eV, the lowest setting in the CE ramp. With higher translational energy proportioned by the increased accelerating potential more energy is available for conversion into internal energy. Higher internal, in turn, increases the likelihood of more expensive bond breakages (Shukla and Futrell (2000)).

The increased C1-decarboxylation for αKG with increasing collision energy cannot be observed for the m/z 88 fragment of Asp in negative ionization mode. As seen in Figure S-1B a C4-decarboxylation independent of the collision-energy takes primarily place, yielding a C123-fragment. This is in contradiction to the assumed carbon identity in Rühl et al. (2012).

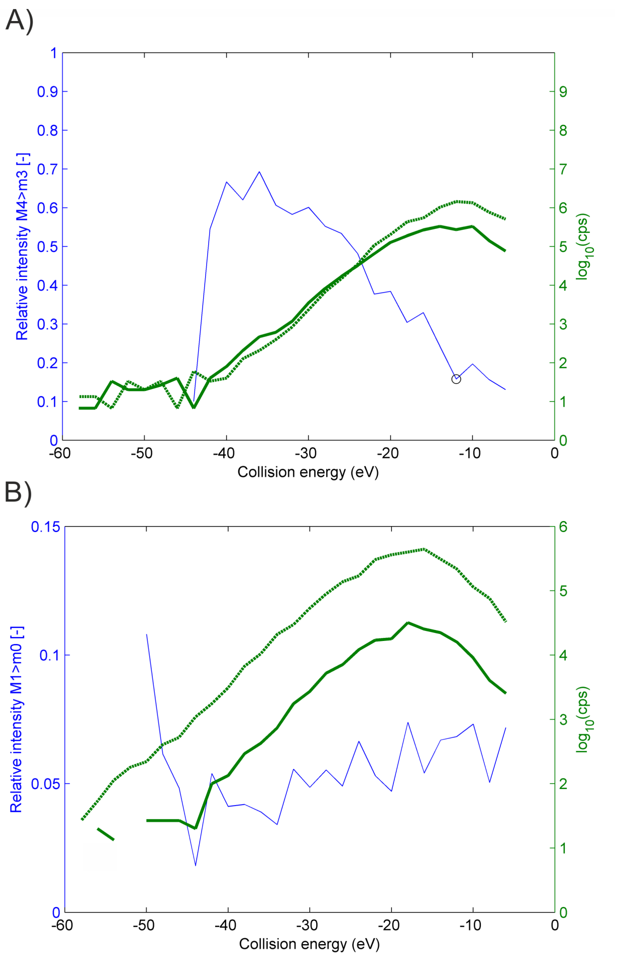

Figure S-1: Collision energy dependence of the share of C1-decarboxylation for αKG and Asp. A) Collision energy dependence of the relative intensity of mass transition M4>m3 for the m/z 101 fragment of αKG upon direct infusion of [1,2,3,4-^{13}C]αKG. Blue solid line: relative signal intensity of M4>m3 to M4>m4; green solid line: logarithmic signal intensity on the mass transition M4>m3 (149.017/104.025); green dashed line: logarithmic signal intensity on the mass transition M4>m4 (149.017/105.054). The black open circle marks the CE-optimized signal intensity derived from tuning an unlabeled standard. B) Collision energy dependence of the relative intensity of mass transition M1>m0 for the m/z 88 fragment of Asp in negative ionization mode upon direct infusion of [1-^{13}C]Asp. Blue solid line: relative signal intensity of M1>m0 to M1>m1; green solid line: logarithmic signal intensity on the mass transition M1>m0 (133.128/87.802); green dashed line: logarithmic signal intensity on the mass transition M1>m1 (133.128/88.784).

S 11 Fragmentation Pathways of Selected Metabolites

Pyruvate Fragmentation Scheme

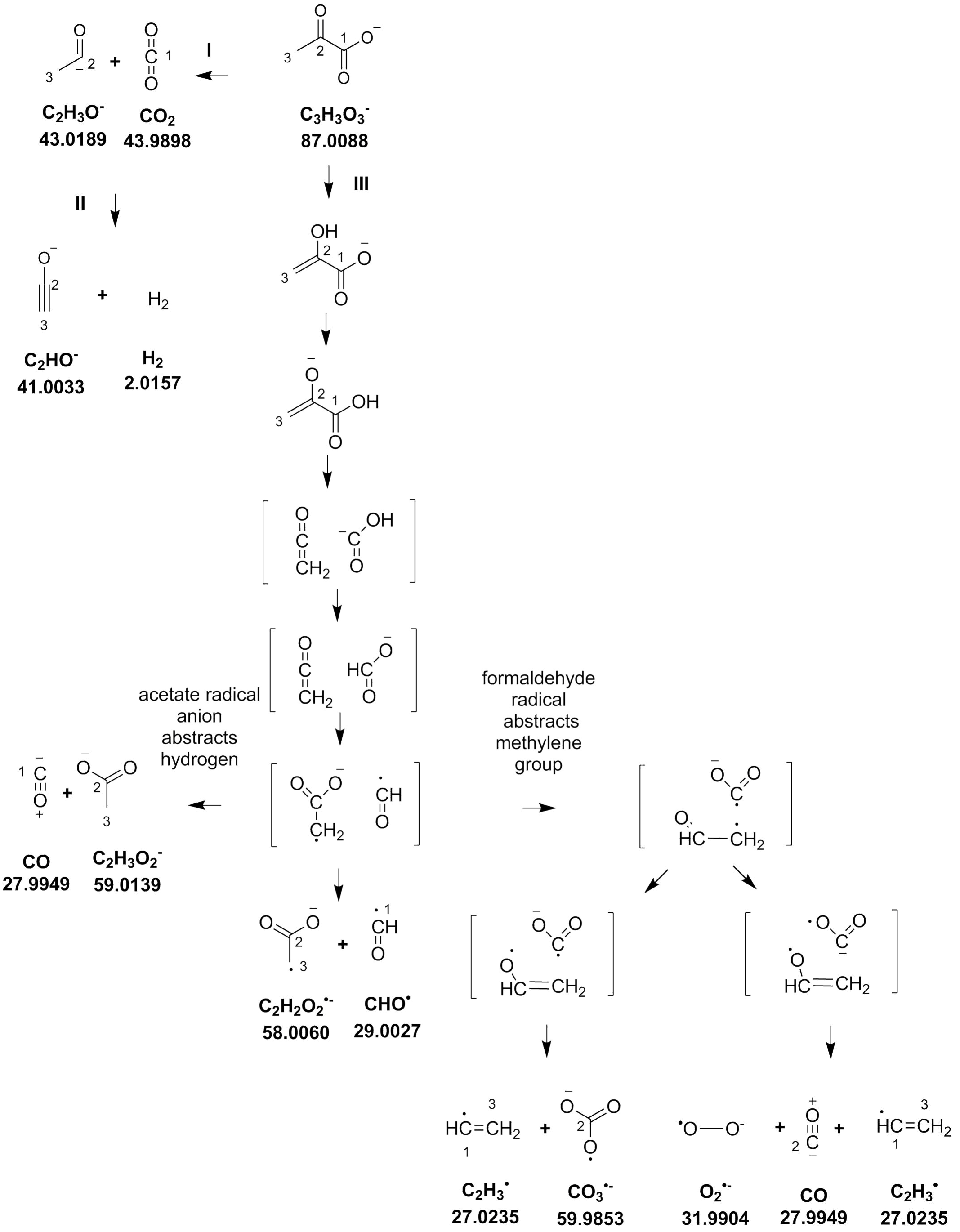

Scheme S-2: The fragmentation pathways of the [M-H]$^-$ ion of pyruvate upon collision induced dissociation. Each ionized fragment can be found in the TQMS-product ion spectrum recorded in the range of -130 to -5 eV (laboratory frame). This pathway is in agreement with the accurate mass annotation and the observed mass shift in the mass spectra of [1-^{13}C]pyruvate, [2-^{13}C]pyruvate and [3-^{13}C]pyruvate (see Supplementary Information S7).

The mechanism for the CO-elimination from C1 through pathway **III**, which proceeds through an ion-molecule complex, was proposed by Eichinger et al. (1990) for deprotonated pyruvate derivatives. We propose the formation of the acetic acid anion at m/z 59.0139 through hydrogen abstraction from the formyl radical. The complex may also dissociate and generate the m/z 58 mass peak corresponding to the acetic acid radical ion. A series of radical atom abstractions explains the appearance of the m/z 59.9853 and m/z 31.9904 peak. Fragment ion m/z 59.9853 does not appear in the QTOF-spectrum recorded over an collision energy ramp from -90 to -5 eV and can therefore not be annotated with an exact mass. However, for the nominal mass of m/z 60 only one possible elemental composition exists, which is derived from the $[M\text{-}H]^-$ ion of pyruvate. The QTOF-Spectrum of pyruvate exhibits a mass peak at 41.9976 (see Supplementary Information S5), which allows only a fragment with two carbon atoms. However, this fragment does not show a mass shift for [2-^{13}C]pyruvate and [3-^{13}C]pyruvate and can therefore not be derived from these atoms. We judge this fragment to be a contamination.

Malate Fragmentation Scheme

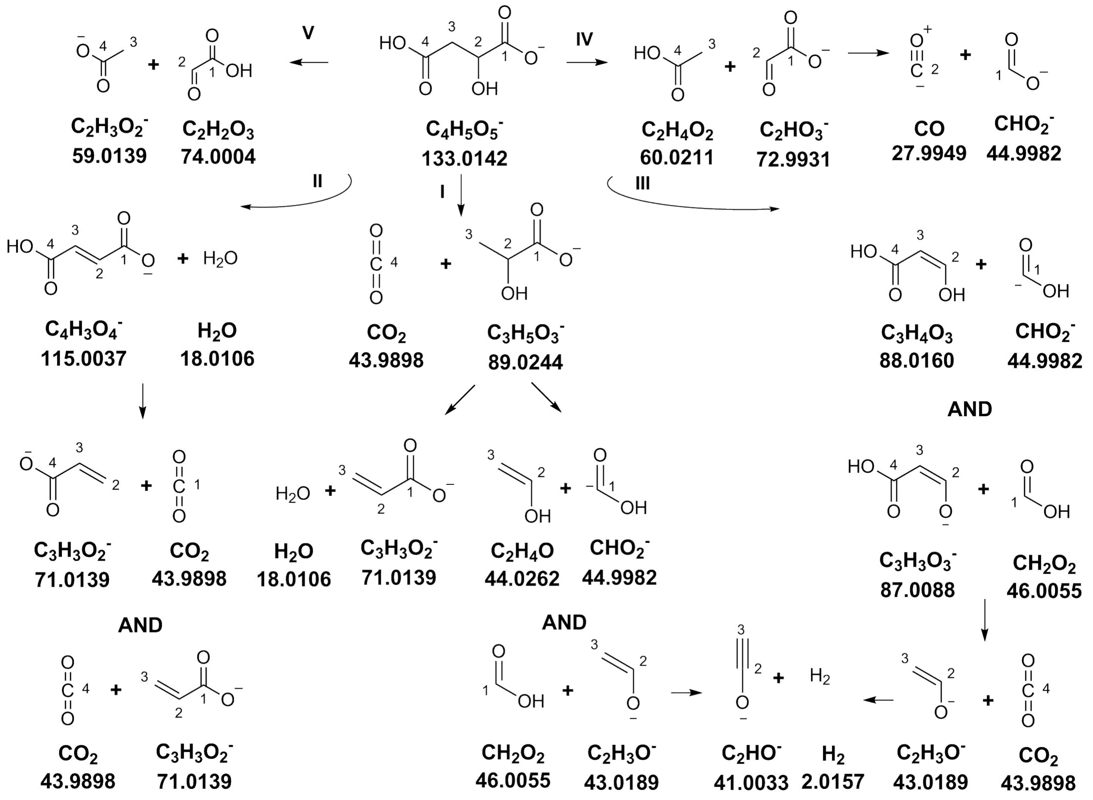

Scheme S-3: The fragmentation pathways of the [M-H]$^-$ ion of malate upon collision induced dissociation. Each ionized fragment can be found in the QqQ product ion spectrum recorded in the range of -130 to -5 eV (laboratory frame). The word "AND" signifies that one fragmentation reaction generates a complementary pair of product ions. Apart from the fragments discussed in the main text, we furthermore validated the uniqueness of carbon origin for fragments m/z 43, 59 and 89 by means of the MRM assay and selectively-labeled species discussed in the main text (data not shown). To account for the asymmetric carbon origin of the m/z 71 fragment of malate, we propose a reaction from the lactate anion through water elimination leading to the m/z 71 fragment.

Aspartate Fragmentation Scheme

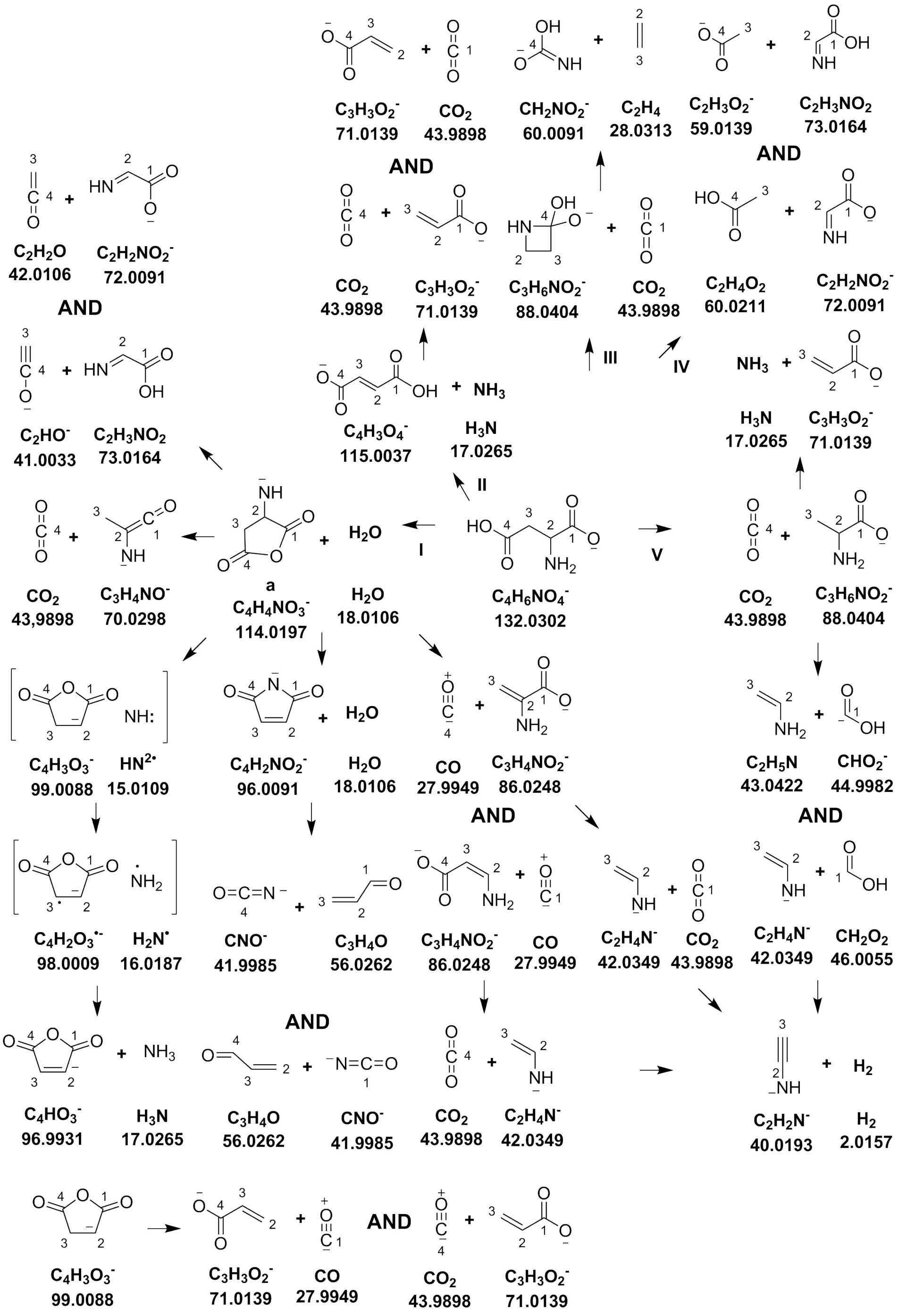

Scheme S-4: The fragmentation pathways of the [M-H]$^-$ ion of aspartate upon collision induced dissociation. Each ionized fragment can be found in the product ion spectrum recorded in the range of -130 to -5 eV (laboratory frame) or in the QTOF-spectrum over an CE-ramp from -90 eV to -5 eV.

Pathways **II**, **IV**, **V** are mechanistically identical to those of Mal. This pathway is consistent with the observed exact mass shift in the mass spectra of [1-^{13}C]Asp and [4-^{13}C]Asp

(see Supplementary Information S7). Both spectra suggest a mixed carbon ancestry for the m/z 41.9985, 71.0139, 86.0248 and 88.0404 fragment ions. We recorded the QTOF mass spectrum of (R)-3-Aminodehydrofuran-2,5-dione hydrochloride (corresponding to structure **a**, exact mass: 114.0197) and observed product ions from the spectrum of Asp at m/z=40.0201, 41.0041, 41.9994, 42.0395, 70.0319, 71.0161, 72.0112, 86.0273, 96.0122, 96.9963 and 98.0042. The presence of ions m/z 70, 71, 72, 86, 97 is consistent with the MS^3 scan of the m/z 114 ion in the study of Eckersley et al. (1989). Therefore, we propose the shown reaction sequence in Scheme S-4 starting with the m/z 114.0197 ion. Importantly, m/z 97, 98 and 99 peaks are not visible in the spectrum of fumarate (see Supplementary Information S7) but only in the one of 3-Aminodehydrofuran-2,5-dione. Therefore, we propose that succinic anhydride (m/z=99.9931) and maleic anhydride (m/z=96.9931) are generated from the m/z=114 fragment and not from the fumaric acid anion at m/z=115. The m/z 41.9985 ion is also of mixed carbon ancestry. Given its formula CON^-, its mixed carbon ancestry can only be rationalized by assuming it to be derived from product ion m/z 96.0091, which appears in the QTOF MS spectrum of Asp as low intensity mass peak. Since (R)-3-Aminodehydrofuran-2,5-dione also generates a m/z 71.0161 ion, we propose the bottommost fragmentation pathway to proceed from the succinic anhydride anion.

We propose pathway **III** to account for the formation of the m/z 60.0091 ion since its elemental composition requires a rearrangement. This pathway would at the same time account for the mixed carbon ancestry of the m/z 88.0404 ion.

α-Ketoglutarate Fragmentation Scheme

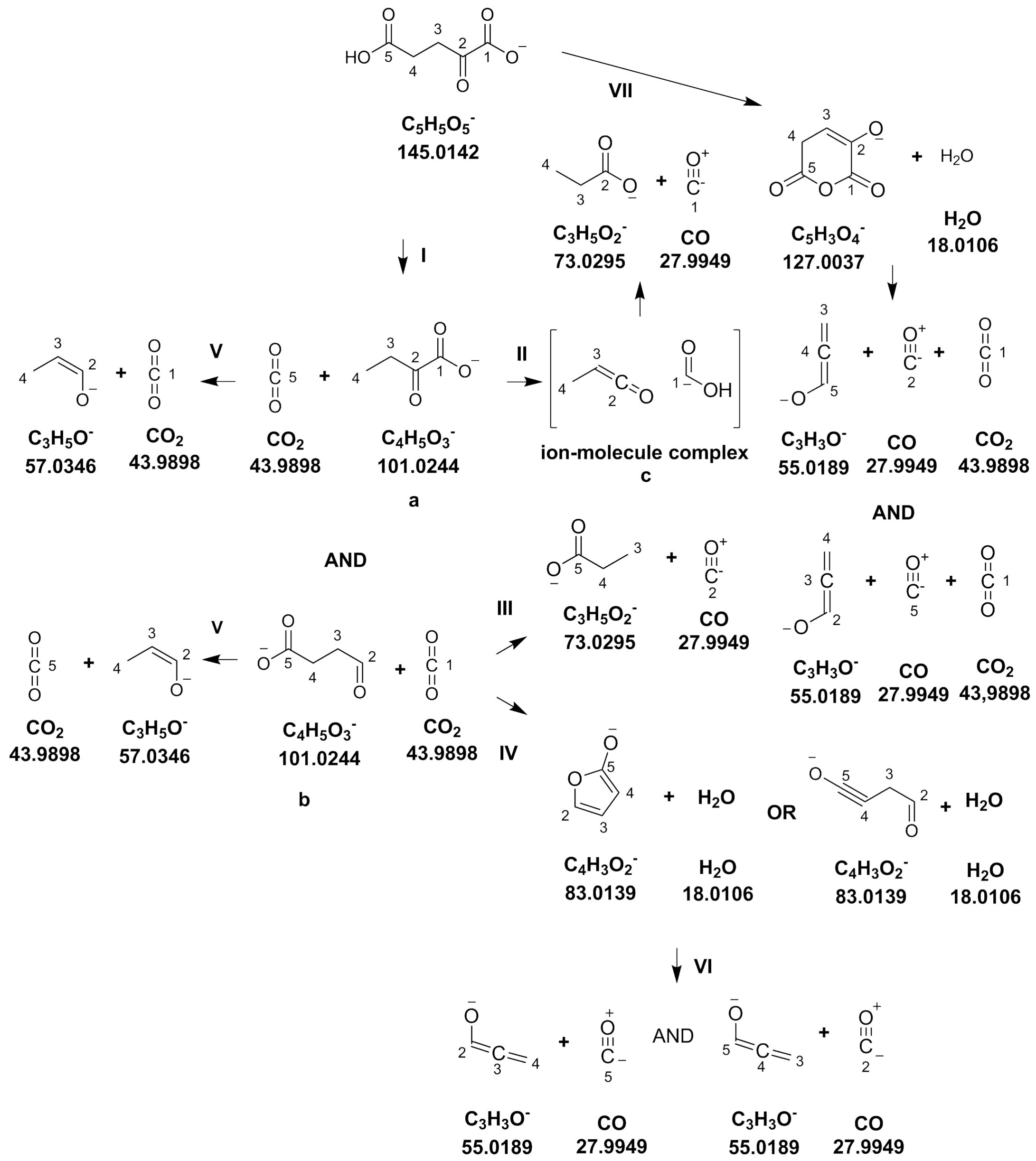

Scheme S-5: The Fragmentation pathways of the [M-H]$^-$ ion of α-ketoglutarate upon collision induced dissociation. Each ionized fragment can be found in the product ion TQMS-spectrum recorded in the range of -130 to -5 eV (laboratory frame).

The product ion spectrum of [1,2,3,4-^{13}C]αKG exhibits one peak at m/z=75 as well as one at m/z=76 (see Supplementary Information S5), both corresponding to the m/z 73 fragment of unlabeled αKG. We confirmed this observation by recording the QTOFMS spectrum of [1,2,3,4-^{13}C]αKG. This spectrum contains two peaks at exact masses of m/z=75.0327 and m/z=76.0360, corresponding to $C_3H_5O_2{}^-$ with two and three ^{13}C-labeled carbon atoms, respectively. Therefore, we propose that fragment m/z 73 is produced by two pathways, i.e. pathway

II and **III**. The former one involves an oxygen rearrangement. On the other hand, the *m/z* 83 fragment ion is solely generated from structure **b**. We verified this sequence by recording the mass spectrum of 2-ketobutyric acid (**a**) and succinic semialdehyde (**b**), which are provided in Supplementary Information S7. Expectedly, both spectra contain the *m/z* 73 ion but only the one of succinic semialdehyde exhibits the *m/z* 83 ion. Furthermore, the *m/z* 55 ion is also unique to succinic semialdehyde. We therefore propose pathway **VI** to yield the *m/z* 55 ion from ring cleavage. This mechanism explains at the same time the signal partition of *m/z* 55 ion into 57.0229 and 58.0260 for [1,2,3,4-^{13}C]αKG in the QTOF mass spectrum, corresponding to C_3H_3O with two and three ^{13}C-labeled carbon atoms. A low intensity peak is visible at *m/z* 127 in the spectrum of αKG which we assume to be the anhydride which fragments to also yield the *m/z* ion of mixed carbon ancestry. The *m/z* 57 ion is generated by both 2-ketobutyric acid and succinic semialdehyde by CO_2 elimination (pathway **V**). We verified that the 1-^{13}C-label is not retained in the *m/z* 73 fragment using the MRM assay described in the main text (data not shown). The mechanism for the CO-elimination from C1 from structure **a** which proceed through an ion-molecule complex **c** was proposed by Eichinger et al. (1990) for deprotonated pyruvate derivatives.

Oxaloacetate Fragmentation Scheme

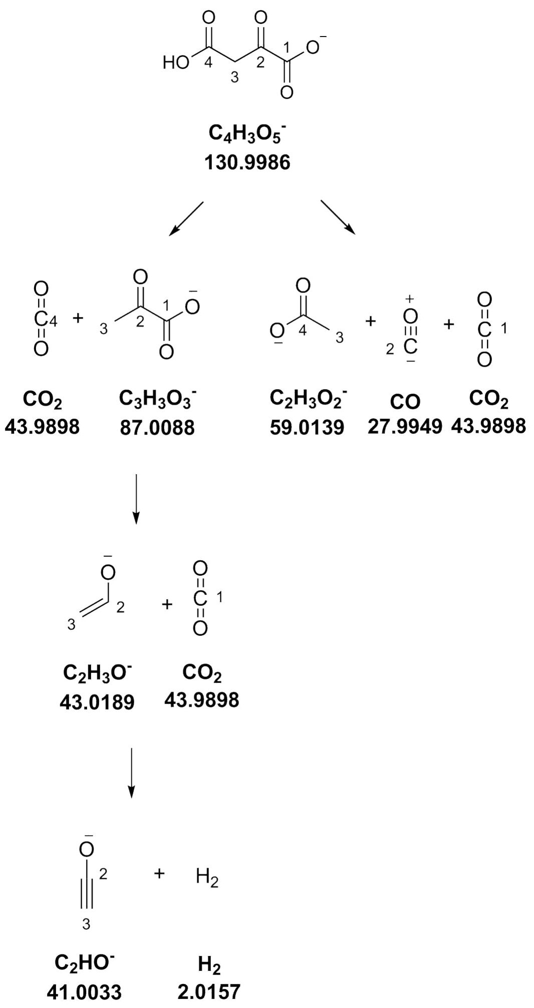

Scheme S-6: The Fragmentation pathways of the [M-H]$^-$ ion of oxaloacetate upon collision induced dissociation. Each ionized fragment can be found in the product ion QqQ mass spectrum recorded in the range of -130 to -5 eV (laboratory frame).

cis-Aconitate Fragmentation Scheme

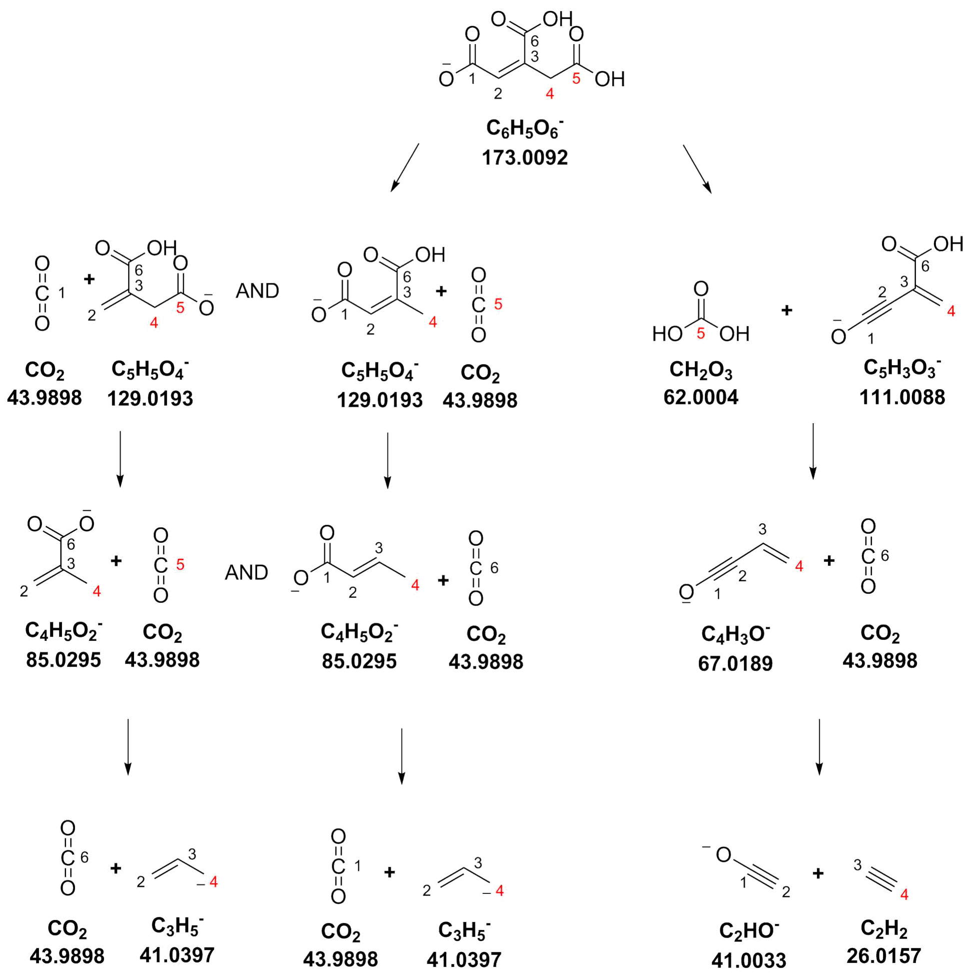

Scheme S-7: The Fragmentation pathways of the $[M\text{-}H]^-$ ion of cis-aconitate upon collision induced dissociation. Each ionized fragment can be found in the QTOF product ion spectrum -50 to -5 eV (laboratory frame).

Citrate Fragmentation Scheme

The fragmentation Scheme of Citrate appears as the superposition of the fragmentation of more basic metabolite fragmentation routes. In the following these individual fragmentations are depicted. Each compound bears the carbon atom labels from the Citrate precursor ion. If a compound corresponding to one mass peak is a mixed carbon origin, both variants are depicted to indicate the flow of carbon during the fragmentation.

Methacrylate Fragmentation Scheme

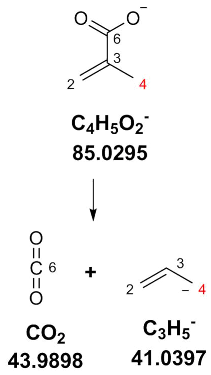

Scheme S-8: The fragmentation pathways of the $[M\text{-}H]^-$ ion of methacrylate upon collision induced dissociation. Each ionized fragment can be found in the product ion QTOF product ion spectrum recorded in the range of -90 to -5 eV (laboratory frame).

Pyruvate Fragmentation Scheme

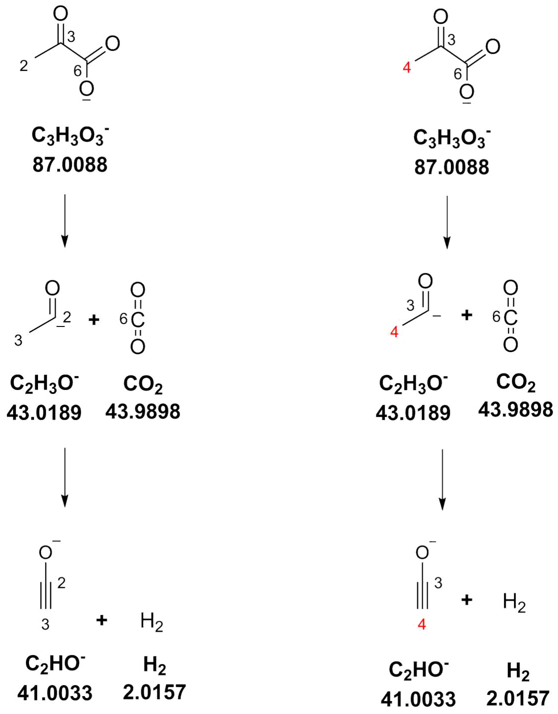

Scheme S-9: The fragmentation pathways of the [M-H]$^-$ ion of pyruvate upon collision induced dissociation. EEach ionized fragment can be found in the product ion QTOF product ion spectrum recorded in the range of -90 to -5 eV (laboratory frame).

Acetoacetate Fragmentation Scheme

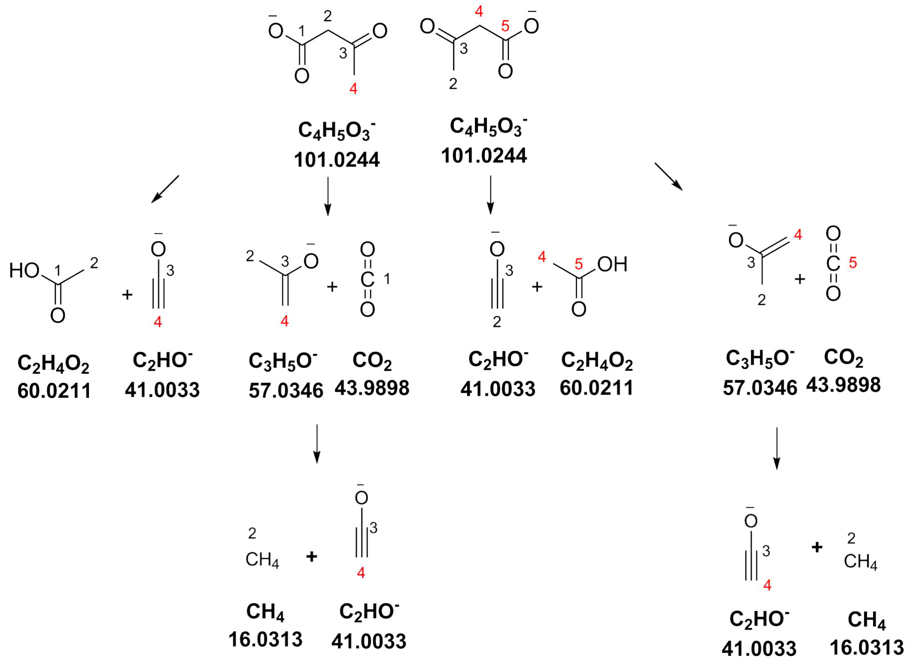

Scheme S-10: The fragmentation pathways of the [M-H]$^{-}$ ion of acetoacetate upon collision induced dissociation. Each ionized fragment can be found in the product ion QTOF product ion spectrum recorded in the range of -90 to -5 eV (laboratory frame).

α-Hydroxyisobutyrate Fragmentation Scheme

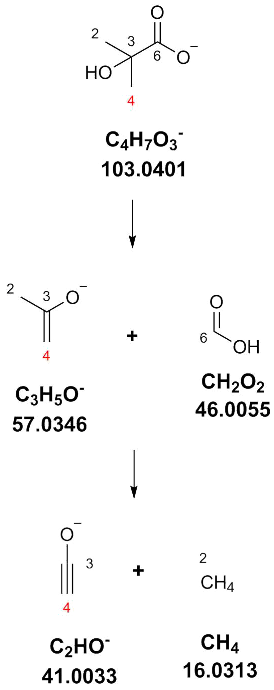

Scheme S-11: The fragmentation pathways of the [M-H]$^-$ ion of alpha-hydroxyisobutyrate upon collision induced dissociation. Each ionized fragment can be found in the product ion QTOF product ion spectrum recorded in the range of -90 to -5 eV (laboratory frame).

4-hydroxy-2-methylenebut-3-ynoate Fragmentation Scheme

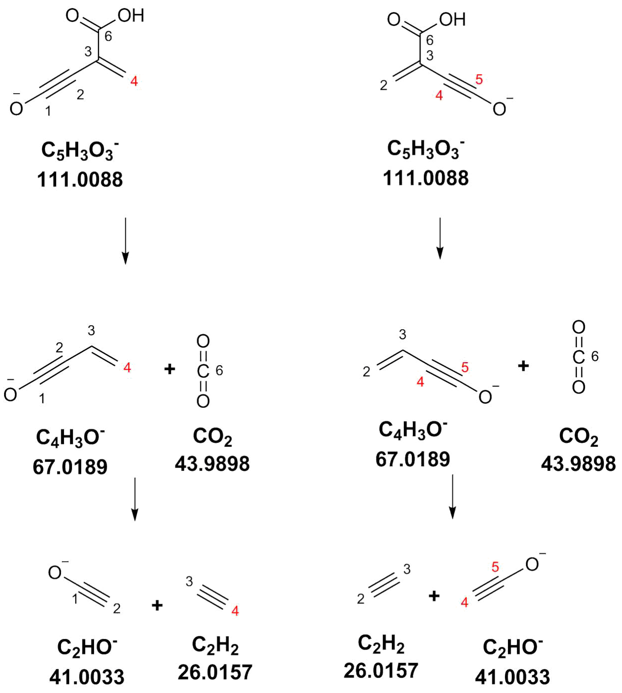

Scheme S-12: The fragmentation pathways of the $[M\text{-}H]^-$ ion of 4-hydroxy-2-methylenebut-3-ynoate acid upon collision induced dissociation. Due to a lack of commercial standards a fragmentation sequence was assumed.

Itaconate Fragmentation Scheme

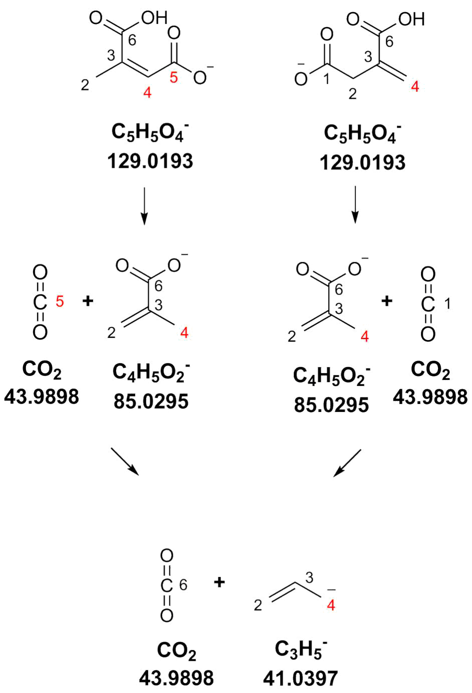

Scheme S-13: The fragmentation pathways of the [M-H]$^-$ ion of itaconate upon collision induced dissociation. Each ionized fragment can be found in the product ion QTOF product ion spectrum recorded in the range of -90 to -5 eV (laboratory frame).

Oxaloacetate Fragmentation Scheme

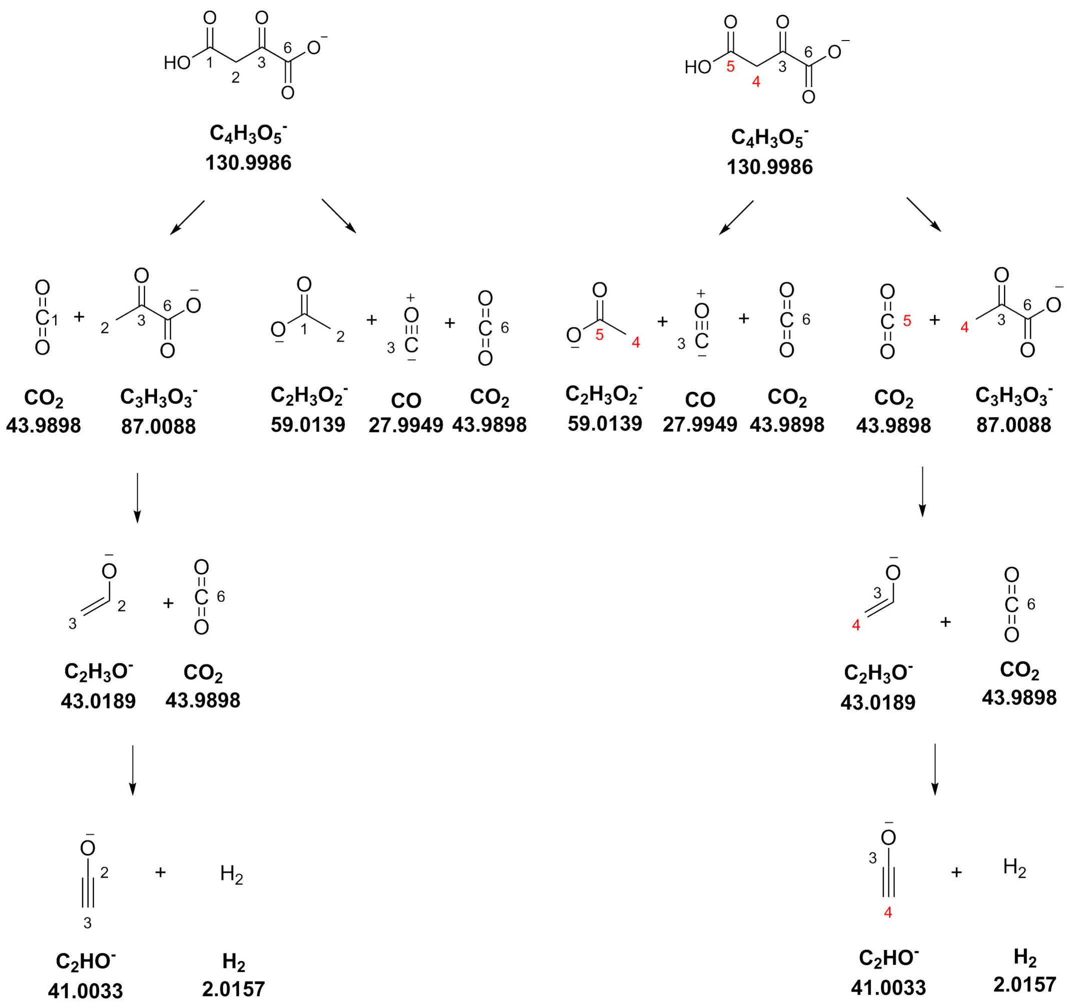

Scheme S-14: The fragmentation pathways of the $[M\text{-}H]^-$ ion of oxaloacetate upon collision induced dissociation. Each ionized fragment can be found in the product ion QTOF product ion spectrum recorded in the range of -90 to -5 eV (laboratory frame).

Citramalate Fragmentation Scheme

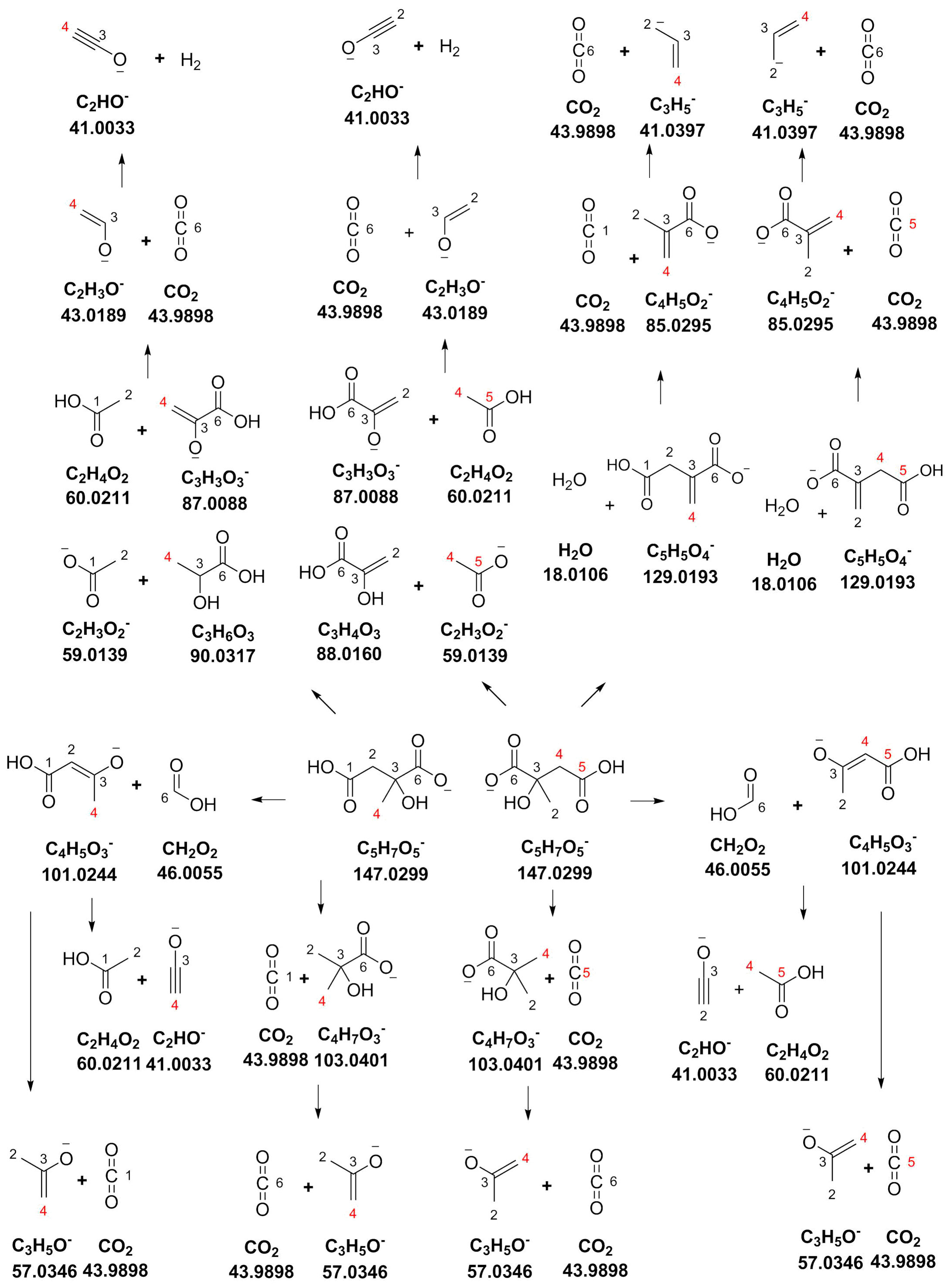

Scheme S-15: The fragmentation pathways of the [M-H]$^{-}$ ion of citramalate upon collision induced dissociation. Each ionized fragment can be found in the product ion QTOF product ion spectrum recorded in the range of -90 to -5 eV (laboratory frame).

3-carboxy-5-hydroxypent-2-en-4-ynoate Fragmentation Scheme

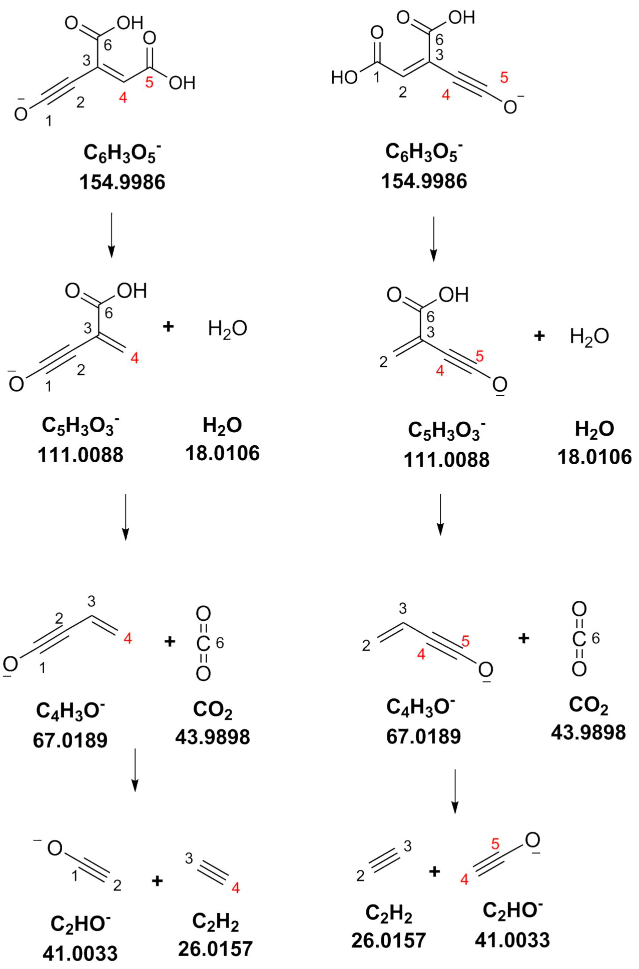

Scheme S-16: The fragmentation pathway of the $[M\text{-}H]^-$ ion of 3-carboxy-5-hydroxypent-2-en-4-ynoate upon collision induced dissociation. Due to a lack of commercial standards a fragmentation sequence was assumed.

trans-aconitate Fragmentation Scheme

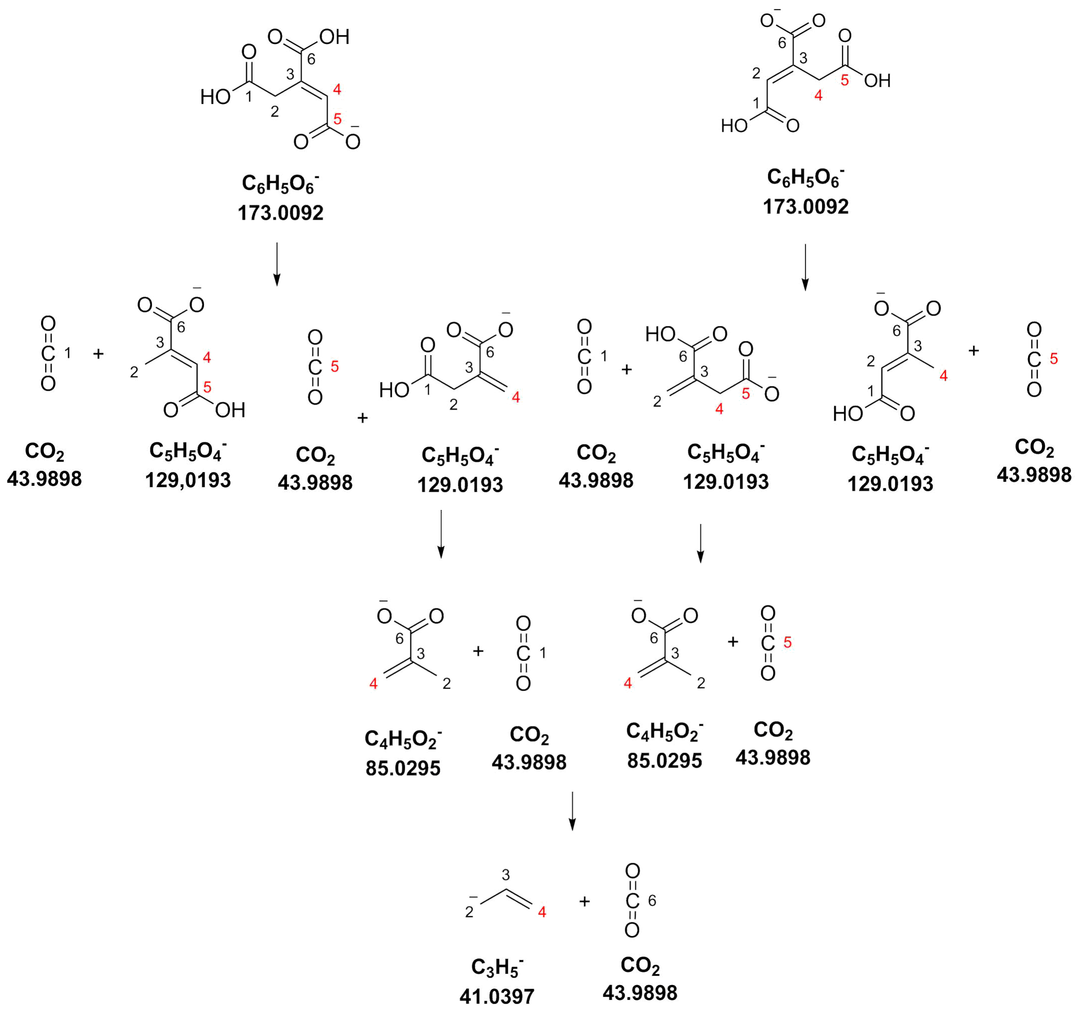

Scheme S-17: The Fragmentation pathways of the [M-H]$^-$ ion of trans-aconitate upon collision induced dissociation. Each ionized fragment can be found in the product ion QTOF product ion spectrum recorded in the range of -90 to -5 eV (laboratory frame).

cis-Aconitate Fragmentation Scheme

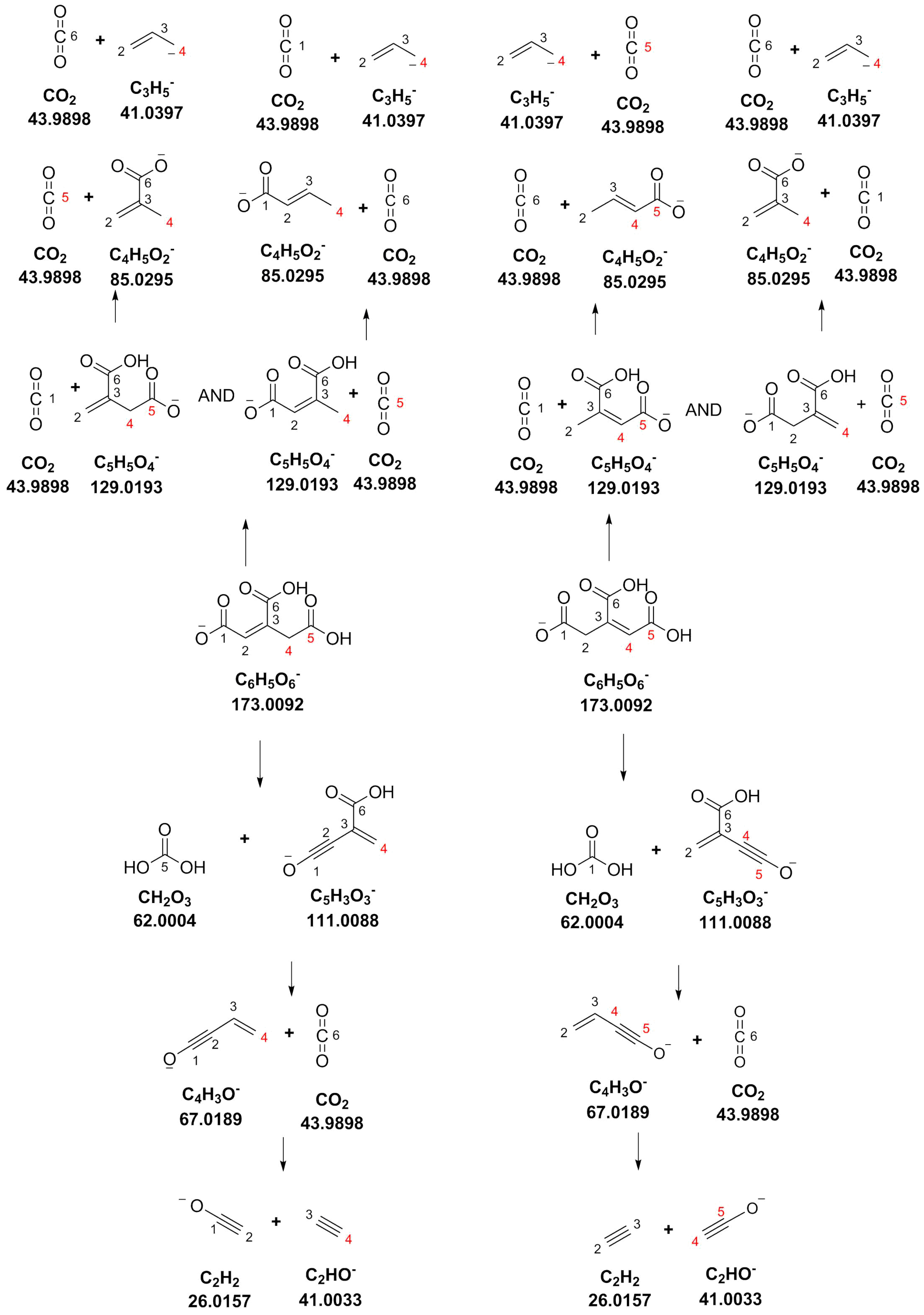

Scheme S-18: The Fragmentation pathways of the [M-H]$^-$ ion of cis-aconitate upon collision induced dissociation. Each ionized fragment can be found in the product ion QTOF product ion spectrum recorded in the range of -90 to -5 eV (laboratory frame).

2-Hydroxy-2-(hydroxyethynyl)succinic acid Fragmentation Scheme

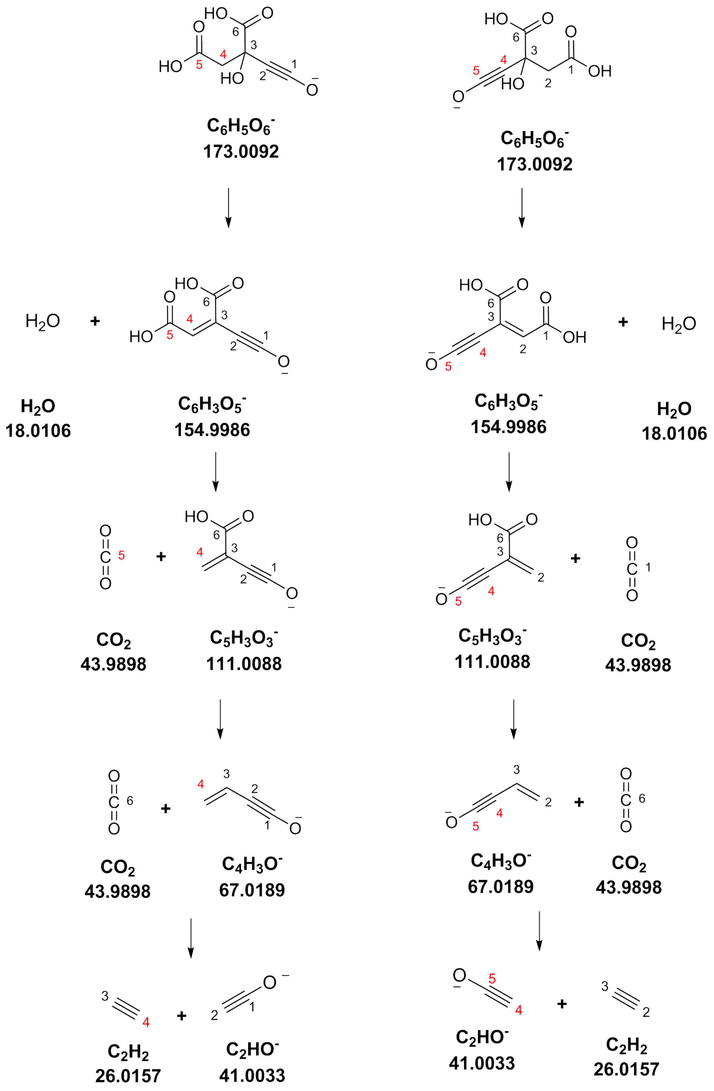

Scheme S-19: The Fragmentation pathways of the $[M\text{-}H]^-$ ion of 2-hydroxy-2-(hydroxyethynyl)succinic acid upon collision induced dissociation. Due to a lack of commercial standards a fragmentation sequence was assumed.

Citrate Fragmentation Scheme

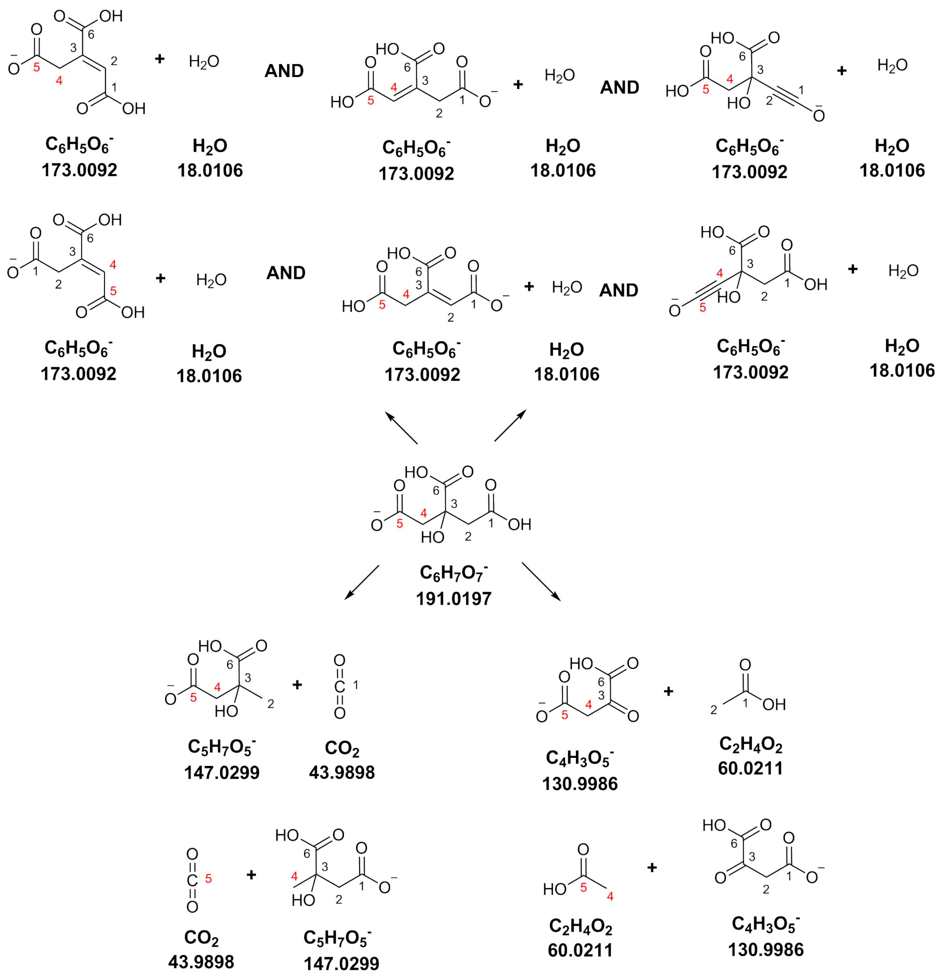

Scheme S-20: The Fragmentation pathways of the [M-H]$^-$ ion of citrate upon collision induced dissociation. Each ionized fragment can be found in the product ion QTOF product ion spectrum recorded in the range of -90 to -5 eV (laboratory frame).

S 12 Exemplary Measurement Matrix for Tandem Mass Isotopomer Distributions

To map the isotopomer vector of a metabolite onto the vector representing the tandem mass isotopomer distribution in a single matrix operation, a unique carbon origin has to be assumed. For the m/z 87 fragment of malic acid, stemming from C2, C3 and C4, the following matrix

operation computes the tandem MS vector for an assumed isotopomer vector:

$$
\begin{pmatrix} M0 > m0 \\ M1 > m0 \\ M1 > m1 \\ M2 > m1 \\ M2 > m2 \\ M3 > m2 \\ M3 > m3 \\ M4 > m3 \end{pmatrix} = \begin{pmatrix}
1 & 0 & 0 & 0 & 0 & 0 & 0 & 0 & 0 & 0 & 0 & 0 & 0 & 0 & 0 & 0 \\
0 & 0 & 0 & 0 & 0 & 0 & 0 & 0 & 1 & 0 & 0 & 0 & 0 & 0 & 0 & 0 \\
0 & 1 & 1 & 0 & 1 & 0 & 0 & 0 & 0 & 0 & 0 & 0 & 0 & 0 & 0 & 0 \\
0 & 0 & 0 & 0 & 0 & 0 & 0 & 0 & 0 & 1 & 1 & 0 & 1 & 0 & 0 & 0 \\
0 & 0 & 0 & 1 & 0 & 1 & 1 & 0 & 0 & 0 & 0 & 0 & 0 & 0 & 0 & 0 \\
0 & 0 & 0 & 0 & 0 & 0 & 0 & 0 & 0 & 0 & 0 & 1 & 0 & 1 & 1 & 0 \\
0 & 0 & 0 & 0 & 0 & 0 & 0 & 1 & 0 & 0 & 0 & 0 & 0 & 0 & 0 & 0 \\
0 & 0 & 0 & 0 & 0 & 0 & 0 & 0 & 0 & 0 & 0 & 0 & 0 & 0 & 0 & 1
\end{pmatrix} \cdot \begin{pmatrix} x_{\mathsf{0000}} \\ x_{\mathsf{0001}} \\ x_{\mathsf{0010}} \\ x_{\mathsf{0011}} \\ x_{\mathsf{0100}} \\ x_{\mathsf{0101}} \\ x_{\mathsf{0110}} \\ x_{\mathsf{0111}} \\ x_{\mathsf{1000}} \\ x_{\mathsf{1001}} \\ x_{\mathsf{1010}} \\ x_{\mathsf{1011}} \\ x_{\mathsf{1100}} \\ x_{\mathsf{1101}} \\ x_{\mathsf{1110}} \\ x_{\mathsf{1111}} \end{pmatrix} \quad (2)
$$

where x_{0000}, x_{0001}, etc. denote the individual isotopomers of malic acid. The entire vector will be denoted $\mathbf{x}_{\mathsf{MAL}}$. Analogously for the m/z 89 fragment, whose carbon atoms stem from

position C1, C2 and C3, the following measurement matrix can be derived:

$$\begin{pmatrix} M0 > m0 \\ M1 > m0 \\ M1 > m1 \\ M2 > m1 \\ M2 > m2 \\ M3 > m2 \\ M3 > m3 \\ M4 > m3 \end{pmatrix} = \begin{pmatrix} 1 & 0 & 0 & 0 & 0 & 0 & 0 & 0 & 0 & 0 & 0 & 0 & 0 & 0 & 0 & 0 \\ 0 & 1 & 0 & 0 & 0 & 0 & 0 & 0 & 0 & 0 & 0 & 0 & 0 & 0 & 0 & 0 \\ 0 & 0 & 1 & 0 & 1 & 0 & 0 & 0 & 1 & 0 & 0 & 0 & 0 & 0 & 0 & 0 \\ 0 & 0 & 0 & 1 & 0 & 1 & 0 & 0 & 0 & 1 & 0 & 0 & 0 & 0 & 0 & 0 \\ 0 & 0 & 0 & 0 & 0 & 0 & 1 & 0 & 0 & 0 & 1 & 0 & 1 & 0 & 0 & 0 \\ 0 & 0 & 0 & 0 & 0 & 0 & 0 & 1 & 0 & 0 & 0 & 1 & 0 & 1 & 0 & 0 \\ 0 & 0 & 0 & 0 & 0 & 0 & 0 & 0 & 0 & 0 & 0 & 0 & 0 & 0 & 1 & 0 \\ 0 & 0 & 0 & 0 & 0 & 0 & 0 & 0 & 0 & 0 & 0 & 0 & 0 & 0 & 0 & 1 \end{pmatrix} \cdot \begin{pmatrix} x_{0000} \\ x_{0001} \\ x_{0010} \\ x_{0011} \\ x_{0100} \\ x_{0101} \\ x_{0110} \\ x_{0111} \\ x_{1000} \\ x_{1001} \\ x_{1010} \\ x_{1011} \\ x_{1100} \\ x_{1101} \\ x_{1110} \\ x_{1111} \end{pmatrix} \tag{3}$$

Following the terminology in Tepper and Shlomi (2013), we denote the vector on the left hand side in equation (2) $\text{TMID}_{\mathsf{MAL},87}$ and in equation (3) $\text{TMID}_{\mathsf{MAL},89}$. The binary matrices in both equations are correspondingly denoted $\text{C}^{87}_{\mathsf{MAL}}$ and $\text{C}^{89}_{\mathsf{MAL}}$. Using LC-MS/MS technology both vectors $\text{TMID}_{\mathsf{MAL},87}$ and $\text{TMID}_{\mathsf{MAL},89}$ are experimentally accessible and are given by numbers. To compute the isotopomer vector $\mathbf{x}_{\mathsf{MAL}}$ from $\text{TMID}_{\mathsf{MAL},87}$ or $\text{TMID}_{\mathsf{MAL},89}$ the matrices $\text{C}^{87}_{\mathsf{MAL}}$ and $\text{C}^{89}_{\mathsf{MAL}}$ need to have full rank (i.e. rank=16). It turns out that both $\text{C}^{87}_{\mathsf{MAL}}$ and $\text{C}^{89}_{\mathsf{MAL}}$ have a rank of 8. Thus, neither equation (2) nor equation (3) can be inverted for the isotopomer

vector $\mathbf{x}_{\mathsf{MAL}}$. However, the TMID of both fragments can be combined in the following way:

$$\begin{pmatrix} \mathsf{TMID}_{\mathsf{MAL},87} \\ \mathsf{TMID}_{\mathsf{MAL},89} \end{pmatrix} = \begin{pmatrix} \mathsf{C}^{87}_{\mathsf{MAL}} \\ \mathsf{C}^{89}_{\mathsf{MAL}} \end{pmatrix} \cdot \begin{pmatrix} x_{0000} \\ x_{0001} \\ x_{0010} \\ x_{0011} \\ x_{0100} \\ x_{0101} \\ x_{0110} \\ x_{0111} \\ x_{1000} \\ x_{1001} \\ x_{1010} \\ x_{1011} \\ x_{1100} \\ x_{1101} \\ x_{1110} \\ x_{1111} \end{pmatrix} = \mathsf{C}^{87,89}_{\mathsf{MAL}} \cdot \mathbf{x}_{\mathsf{MAL}} \tag{4}$$

The matrix arising from concatenating $\mathrm{C}^{87}_{\mathsf{MAL}}$ and $\mathrm{C}^{89}_{\mathsf{MAL}}$ in equation (4) has rank of 11. Thus, both TMID in conjunction are more informative with respect to $\mathbf{x}_{\mathsf{MAL}}$ than each single TMID, but still do not allow to infer $\mathbf{x}_{\mathsf{MAL}}$ outright. The process of concatenating individual measurement matrices of type $\mathrm{C}^{87}_{\mathsf{MAL}}$ can be continued to include the TMID of further fragments with unique carbon origin. Combining all product ions with unique carbon origin from the product ion spectrum of Mal yields a matrix $\mathsf{C}^{\mathsf{all}}_{\mathsf{MAL}}$ with a rank of 16, i.e. full rank. Therefore, it can be inverted for $\mathbf{x}_{\mathsf{MAL}}$. Alternatively, the individual matrices $\mathbf{C}$ for all measurable fragments of a particular metabolite can be concatenated, yielding $\mathsf{C}^{\mathsf{all}}_{\mathsf{i}}$. We computed matrix $\mathsf{C}^{\mathsf{all}}_{\mathsf{i}}$ for all considered metabolites and determined its rank, which is given in Supplementary Information S2. A product ion spectrum with various positional carbon atom breaks yields a high rank for matrix $\mathsf{C}^{\mathsf{all}}_{\mathsf{i}}$ and optimally a full rank, i.e. $\mathrm{rank}(\mathsf{C}^{\mathsf{all}}_{\mathsf{i}})=\dim(\mathbf{x}_{\mathsf{i}})$.

Now, for alternative carbon origins (C1 vs C4 for Asp or C1 vs C5 for Glu) the tandem mass isotopomer measurement matrix of the type in equation (2) looks different. However, since C1 or C4 refer to a particular ordering of isotopomers, the measurement matrix for one of two possible carbon origins can also describe the alternative carbon origin, if the ordering of the isotopomers is correspondingly changed. Analogously, the tandem mass isotopomer measurement matrix describing one of two alternative carbon origins can be transformed to one describing the other carbon origin by means of a permutation operation according to the

following equation which treats the m/z 71 ion of Mal exemplarily:

$$\mathbf{C}_{\mathsf{MAL}}^{71,Orig1} = \mathbf{C}_{\mathsf{MAL}}^{71,Orig2} \cdot \mathbf{P} \tag{5}$$

As a basic fact of linear algebra, a permutation matrix has always full rank (i.e. 16 for the case of Mal). For matrix multiplication it holds: $rank(\mathbf{A} \cdot \mathbf{B}) = rank(\mathbf{A})$, when $\mathbf{B}$ has full row rank. Therefore, the mapping matrices for different carbon origins have always the same rank. When a measured tandem MS distribution arises from a mixed carbon ancestry, the problem can be formulated following the framework proposed by Tepper and Shlomi (2013):

$$\mathbf{TMID}_{\mathsf{MAL,71}} = (\lambda \mathbf{C}_{\mathsf{MAL}}^{71,Orig1} + (1-\lambda)\mathbf{C}_{\mathsf{MAL}}^{71,Orig2}) \cdot \mathbf{x}_{\mathsf{MAL}} \tag{6}$$

For the symmetric case, i.e. the mixture coefficient λ is known to be 0.5, equation (7) arises after inserting equation (5) into equation (6):

$$\mathbf{TMID}_{\mathsf{MAL,71}} = (0.5 \cdot (\mathbf{C}_{\mathsf{MAL}}^{71,Orig2} \cdot \mathbf{P} + \mathbf{C}_{\mathsf{MAL}}^{71,Orig2})) \cdot \mathbf{x}_{\mathsf{MAL}} \tag{7}$$

In equation (7) we are left with a matrix addition. To determine the positional information content of a TMID which arises from a mixture of carbon origins, we have to analyze the rank of the matrix sum on the right hand side of equation (7). Factoring out $\mathbf{C}_{\mathsf{MAL}}^{71,Orig2}$ yields:

$$\mathbf{TMID}_{\mathsf{MAL,71}} = (0.5 \cdot \mathbf{C}_{\mathsf{MAL}}^{71,Orig2} \cdot (\mathbf{P} + \mathbb{1})) \cdot \mathbf{x}_{\mathsf{MAL}} \tag{8}$$

It turns out that permutation matrix $\mathbf{P}$ is symmetric since independent pairwise column swaps are performed to map $\mathbf{C}_{\mathsf{MAL}}^{71,Orig2}$ onto $\mathbf{C}_{\mathsf{MAL}}^{71,Orig1}$. $\mathbb{1}$ is symmetric by definition. Since symmetry is preserved under matrix addition, the matrix sum $\mathbf{P} + \mathbb{1}$ is also symmetric. Any symmetric real matrix is diagonalizable and has therefore full rank. Thus, it again holds $rank(\mathbf{A} \cdot \mathbf{B})$=$rank(\mathbf{A})$ with respect to equation (8). Therefore, the resulting rank of the expression $0.5 \cdot \mathbf{C}_{\mathsf{MAL}}^{71,Orig2} \cdot (\mathbf{P} + \mathbb{1})$ in equation (8), representing mixed carbon ancestry, has the same rank as $\mathsf{C}_{\mathsf{MAL}}^{71,Orig2}$, representing unique carbon ancestry. The argument also holds for arbitrary λ since a symmetric matrix is still symmetric after scalar multiplication. Therefore, the information content of a mixed carbon ancestry is not reduced in comparison to a unique carbon ancestry as long as the mixture coefficient is known. For product ions derived from symmetric intermediates it can be assumed to be 0.5.

S 13 Total Ion Chromatogram and Product Ion Spectrum of the M1 Mass Isotopomer of Malate

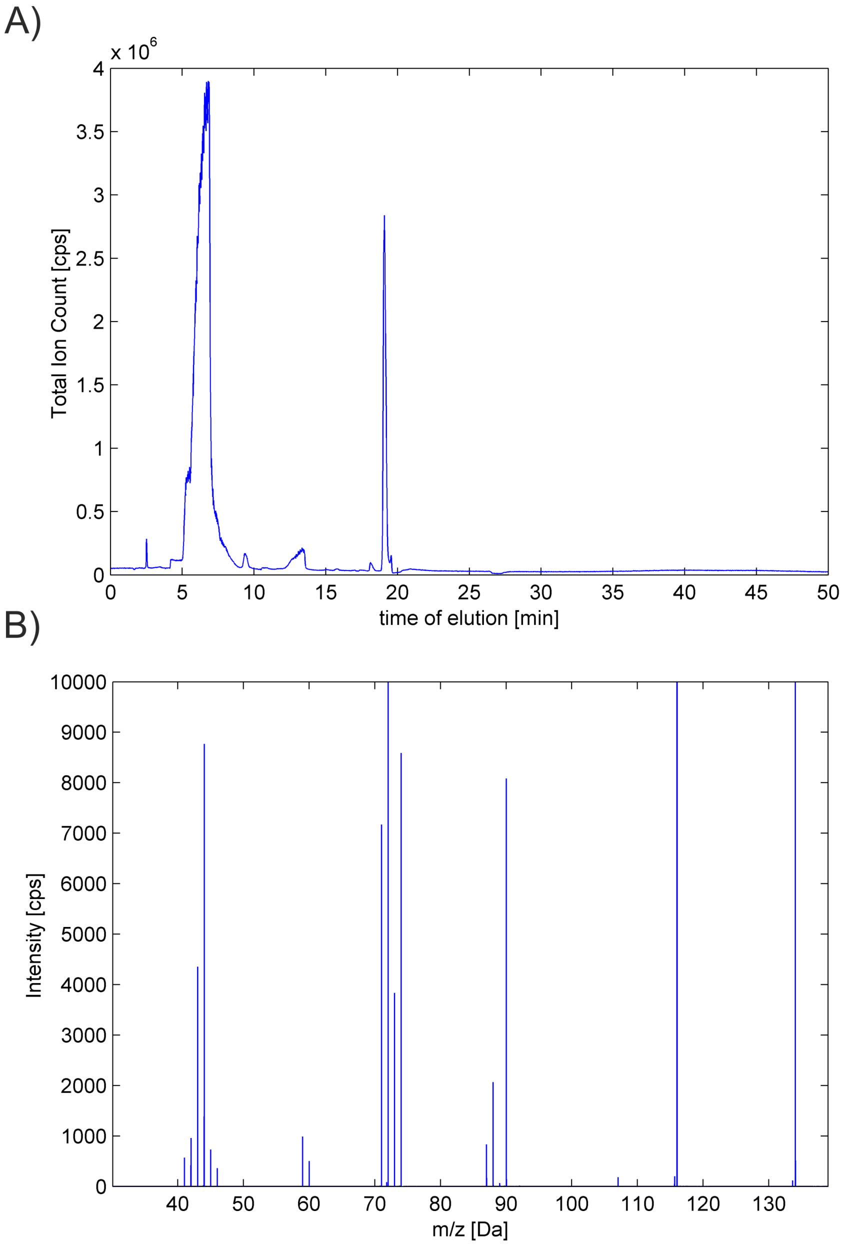

Figure S-21: A) Total Ion Chromatogram during the chromatographic elution described in the Methods section of the main text. The peak centered around 6.76 min represents pyruvate whereas the peak at 19.09 min corresponds to malic acid. B) The product ion spectrum of Q1=134 (mass isotopomer M1 of Malate) in the range of 18.89 min to 19.47 min. The y-range of plot B) was limited to 1e4 to make low intensity peaks visible. The peaks at 72.0182, 116.0091 and 134.0201 exceed the axis limit.

Bibliography

Eckersley, M., Bowie, J. H., Hayes, R. N., 1989. Collision-induced dissociations of deprotonated α-amino acids. The occurrence of specific proton transfers preceding fragmentation. International Journal of Mass Spectrometry and Ion Processes 93 (2), 199–213.

Eichinger, P. C. H., Hayes, R. N., Bowie, J. H., 1990. Anionic rearrangement in the gas phase. the collision-induced loss of carbon monoxide from deprotonated pyruvates and hydroxyacetates. Journal of the Chemical Society, Perkin Transactions 2 (11), 1815–1819.

Horai, H., Arita, M., Kanaya, S., Nihei, Y., Ikeda, T., Suwa, K., Ojima, Y., Tanaka, K., Tanaka, S., Aoshima, K., Oda, Y., Kakazu, Y., Kusano, M., Tohge, T., Matsuda, F., Sawada, Y., Hirai, M. Y., Nakanishi, H., Ikeda, K., Akimoto, N., Maoka, T., Takahashi, H., Ara, T., Sakurai, N., Suzuki, H., Shibata, D., Neumann, S., Iida, T., Tanaka, K., Funatsu, K., Matsuura, F., Soga, T., Taguchi, R., Saito, K., Nishioka, T., 2010. MassBank: a public repository for sharing mass spectral data for life sciences. J Mass Spectrom 45 (7), 703–14.

Rühl, M., Rupp, B., Nöh, K., Wiechert, W., Sauer, U., Zamboni, N., 2012. Collisional fragmentation of central carbon metabolites in LC-MS/MS increases precision of 13C metabolic flux analysis. Biotechnol Bioeng 109 (3), 763–71.

Shukla, A. K., Futrell, J. H., 2000. Tandem mass spectrometry: dissociation of ions by collisional activation. J Mass Spectrom 35 (9), 1069–90.

Tepper, N., Shlomi, T., 2013. An integrated computational approach for metabolic flux analysis coupled with inference of tandem-MS collisional fragments. Bioinformatics 29 (23), 3045–52.

Wang, M., Audi, G., Wapstra, A., Kondev, F., MacCormick, M., Xu, X., Pfeiffer, B., 2012. The AME2012 atomic mass evaluation. Chinese Physics C 36 (12), 1603.

Wolfram-Research, I., 2014. Mathematica edition: Version 9.0.
