# Supplementary material for: Comprehensive and accurate tracking of carbon origin of LC-tandem mass spectrometry collisional fragments for 13C-MFA
Source: Anal Bioanal Chem. 2017 Jan 23;409(9):2309–26. doi: 10.1007/s00216-016-0174-9 (PMC5477699; doi:10.1007/s00216-016-0174-9)
Supplement: Supplementary file 3 — (TAR 5.69 mb) [file 216_2016_174_MOESM3_ESM.zip › 216_2016_174_MOESM3_ESM/S7-MetaboliteMassSpectra.pdf]

S7 - Metabolite Product Ion Spectra with Structures and Supplementary Product Ion Spectra

Negative Ionization Mode

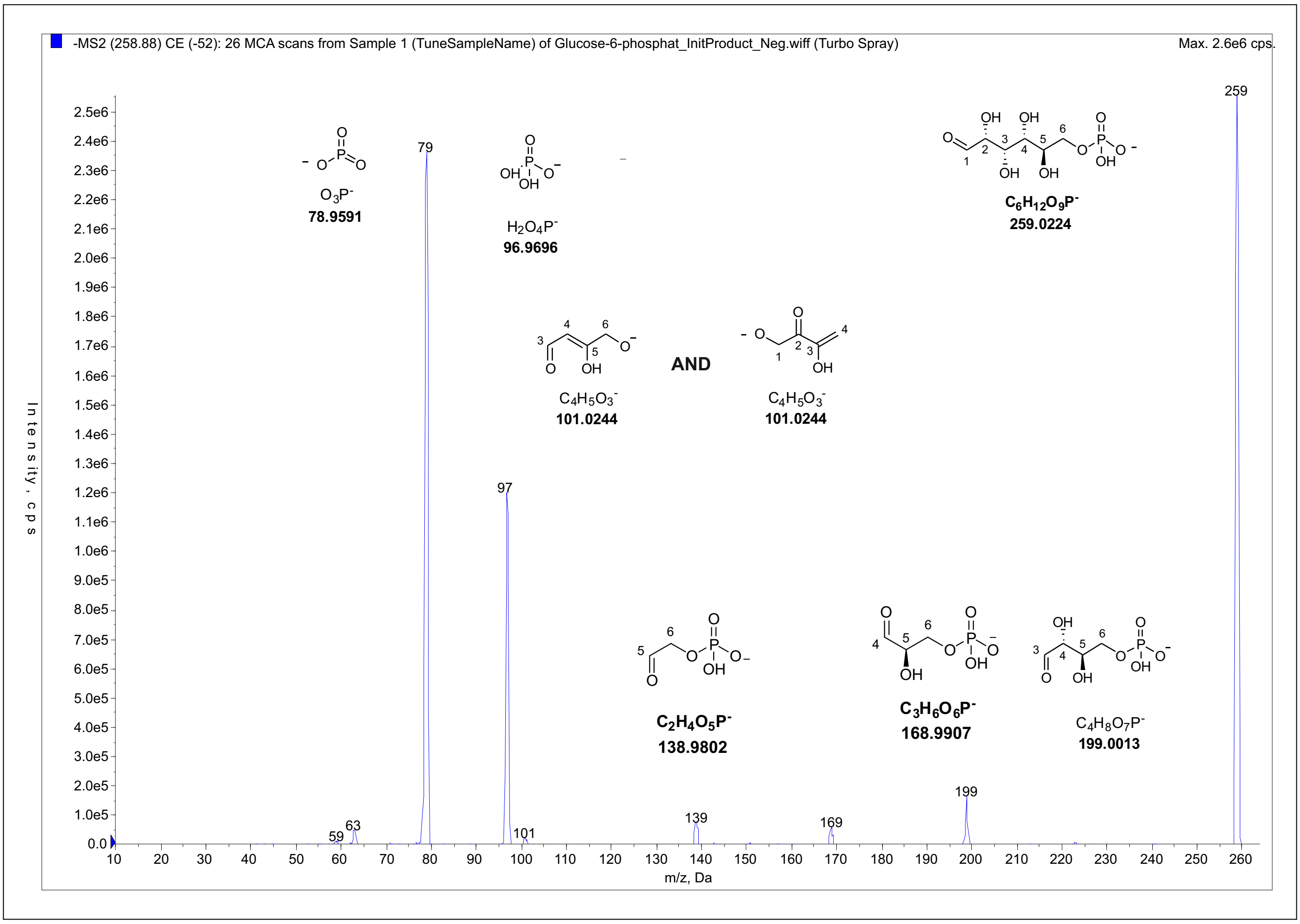

Chart S-1: The product ion spectrum of the [M-H]- ion of glucose 6-phosphate.

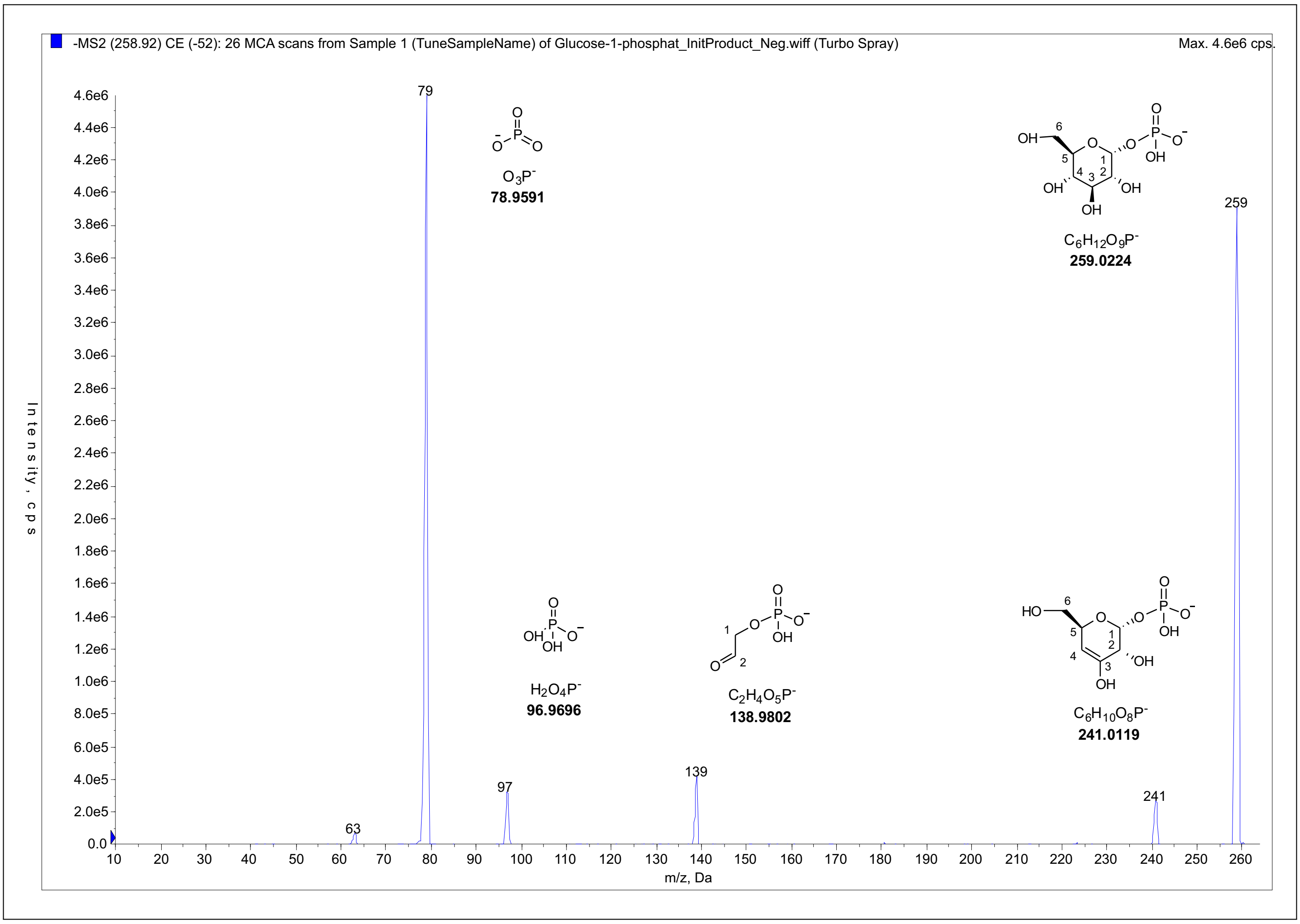

Chart S-2: The product ion spectrum of the [M-H]- ion of glucose 1-phosphate.

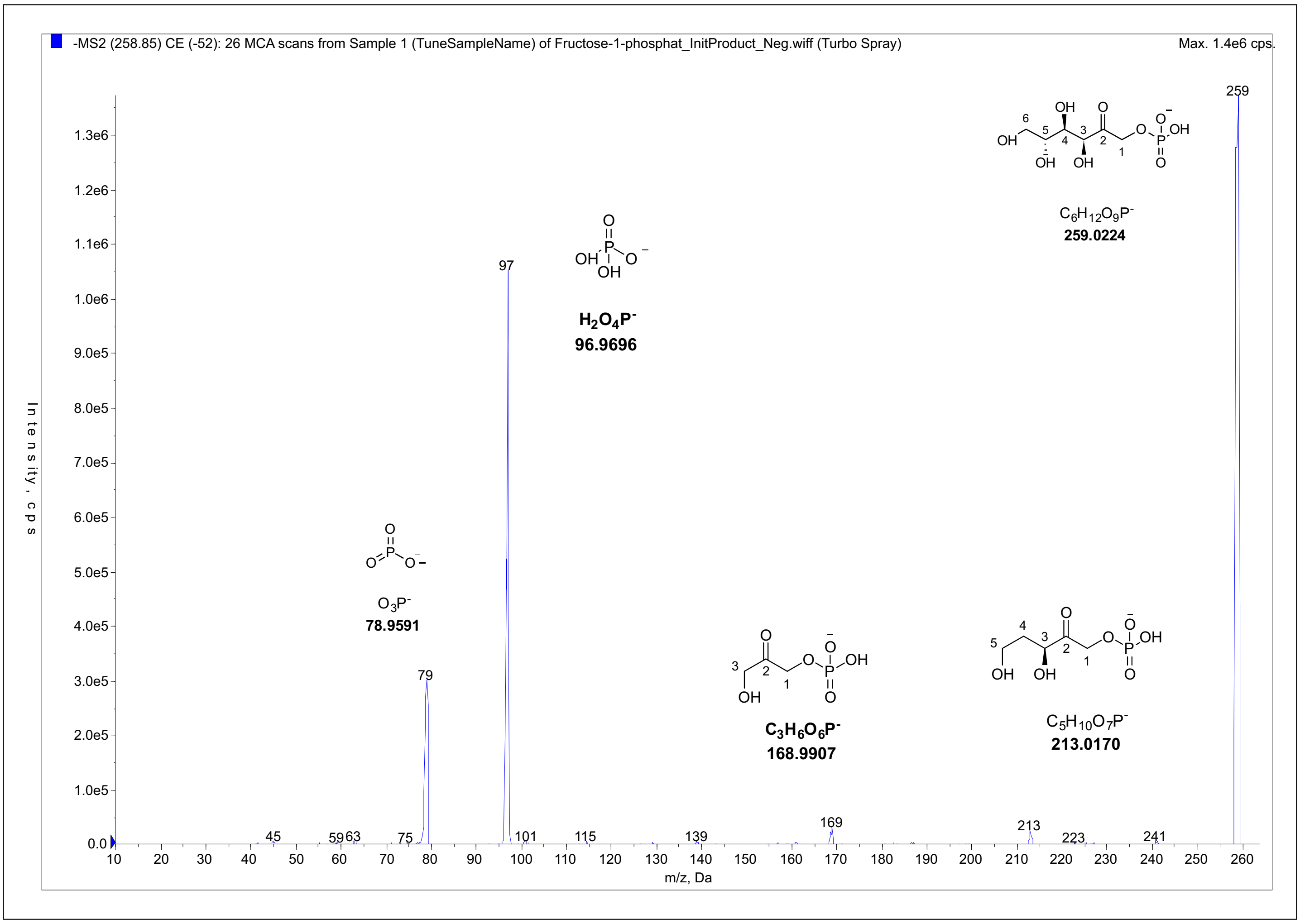

Chart S-3: The product ion spectrum of the [M-H]- ion of fructose 1-phosphate.

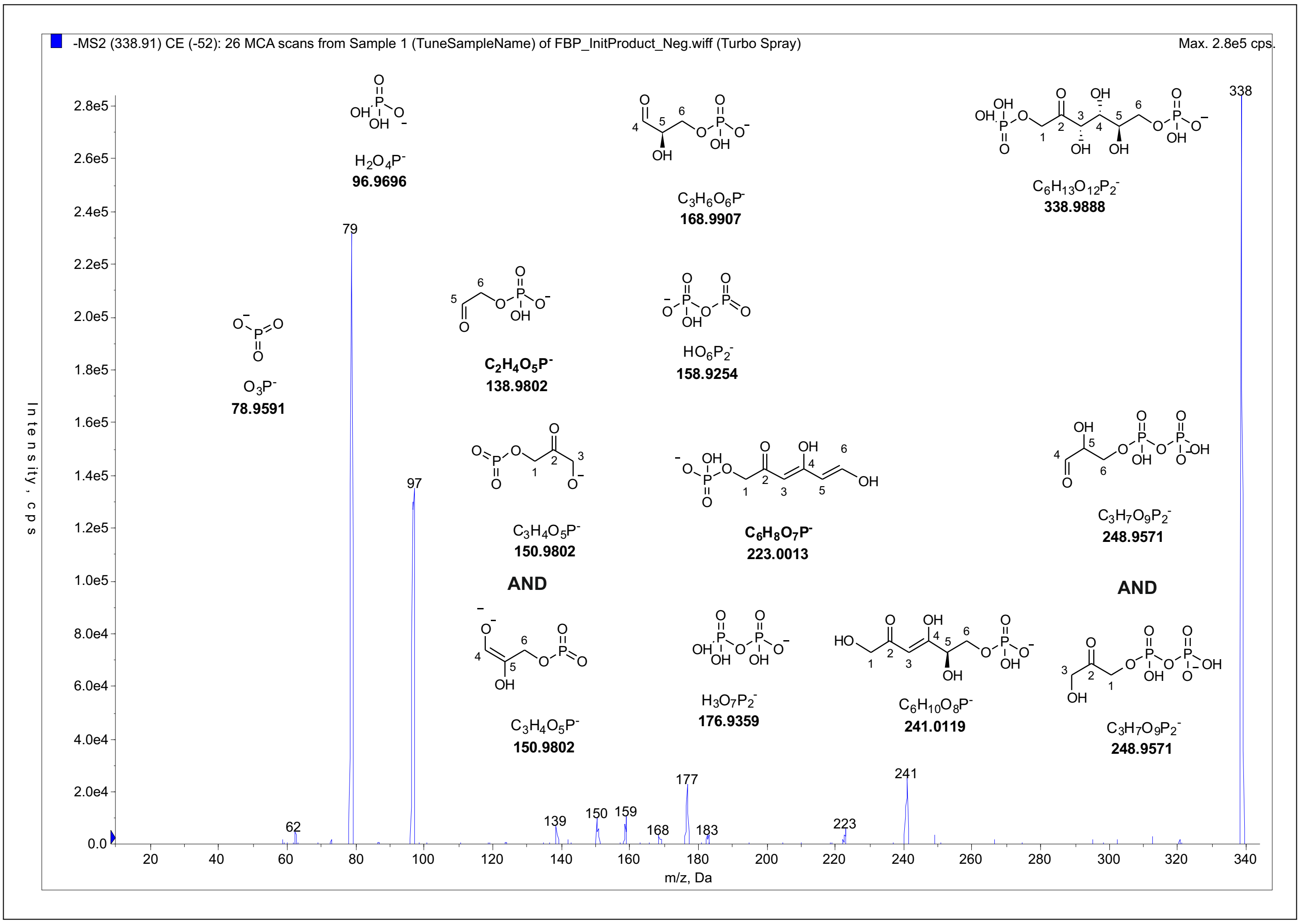

Chart S-4: The product ion spectrum of the [M-H]- ion of fructose 1,6-bisphosphate.

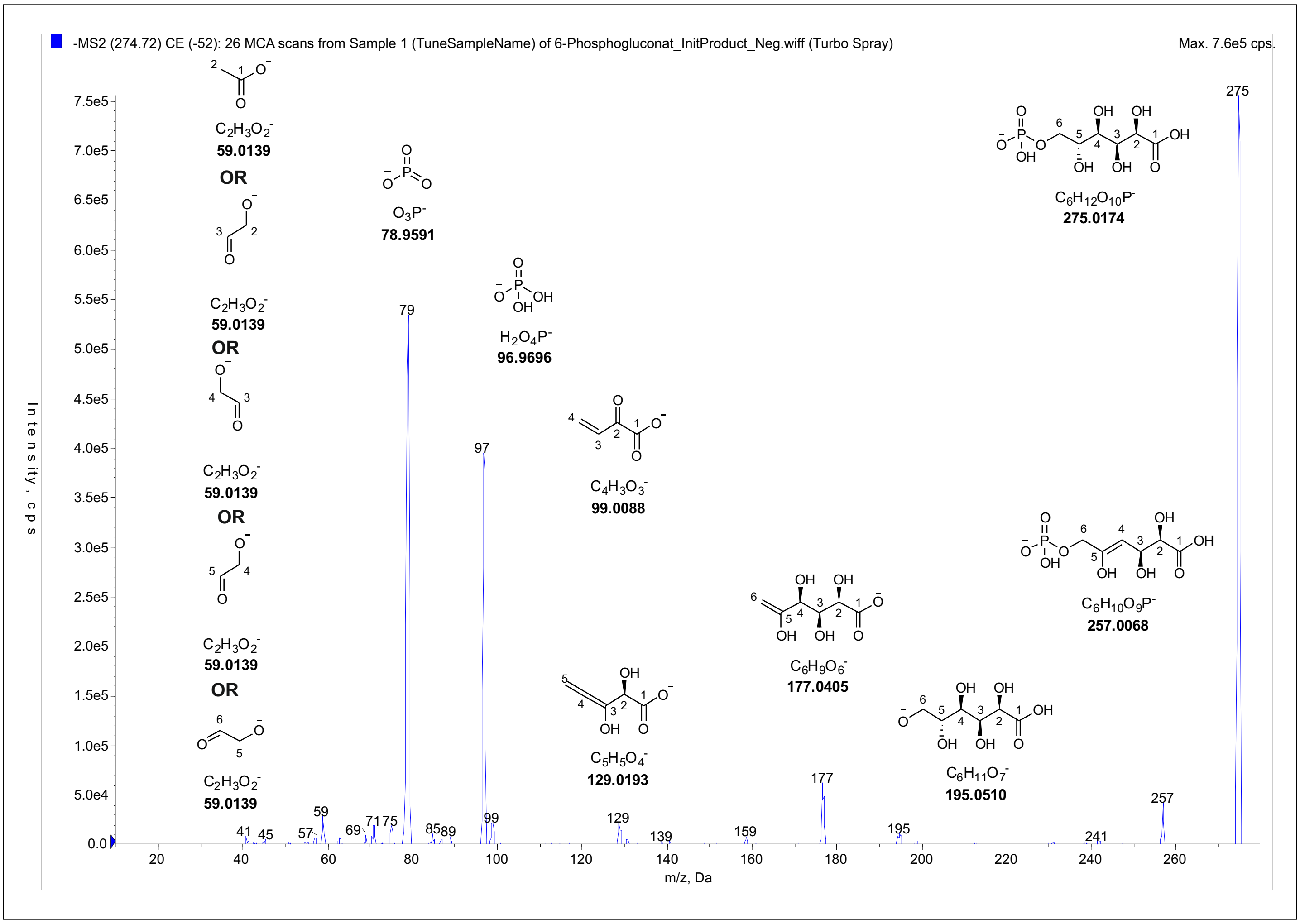

Chart S-5: TThe product ion spectrum of the [M-H]- ion of 6-phosphogluconate.

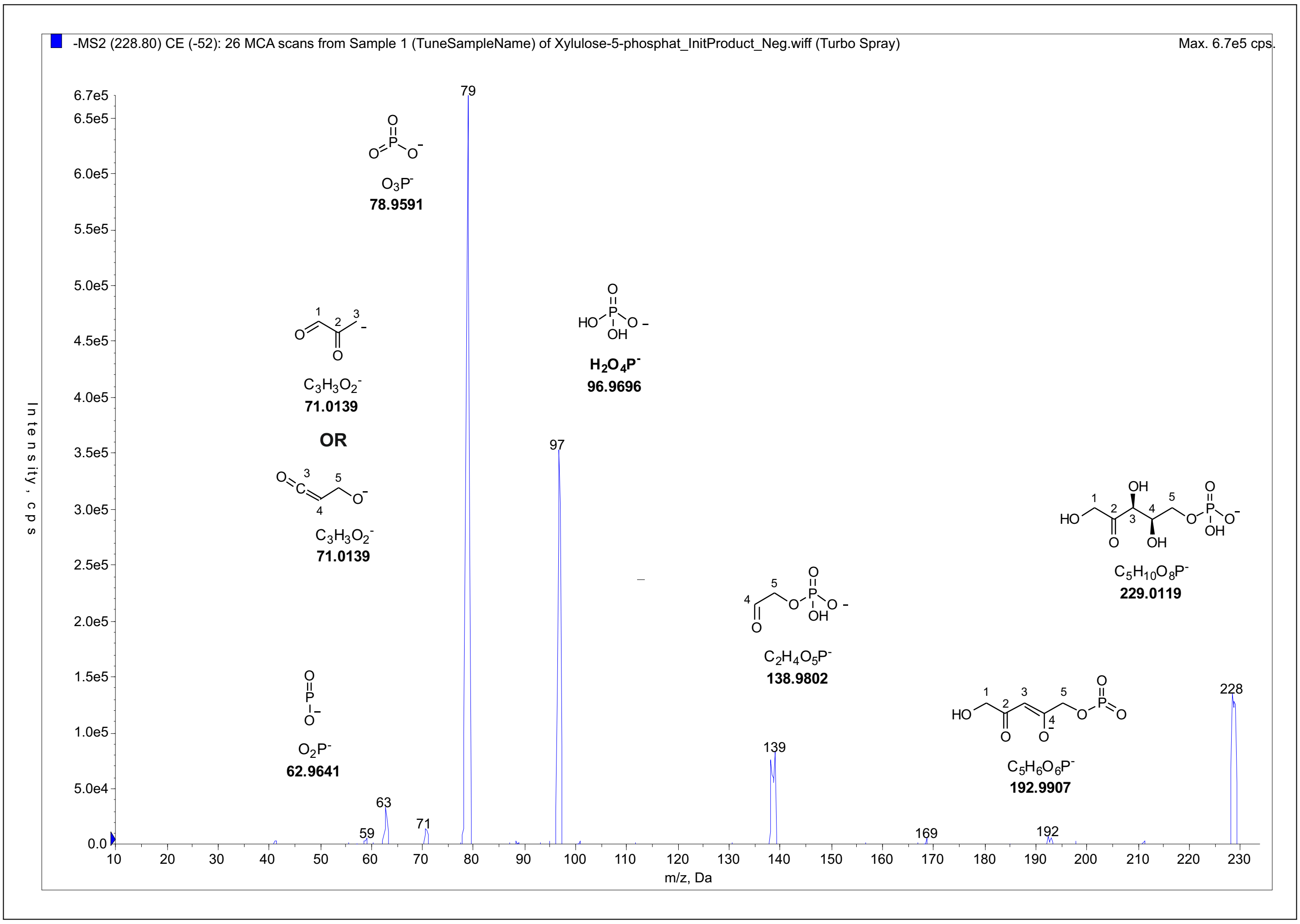

Chart S-6: The product ion spectrum of the [M-H]- ion of xylulose 5-phosphate.

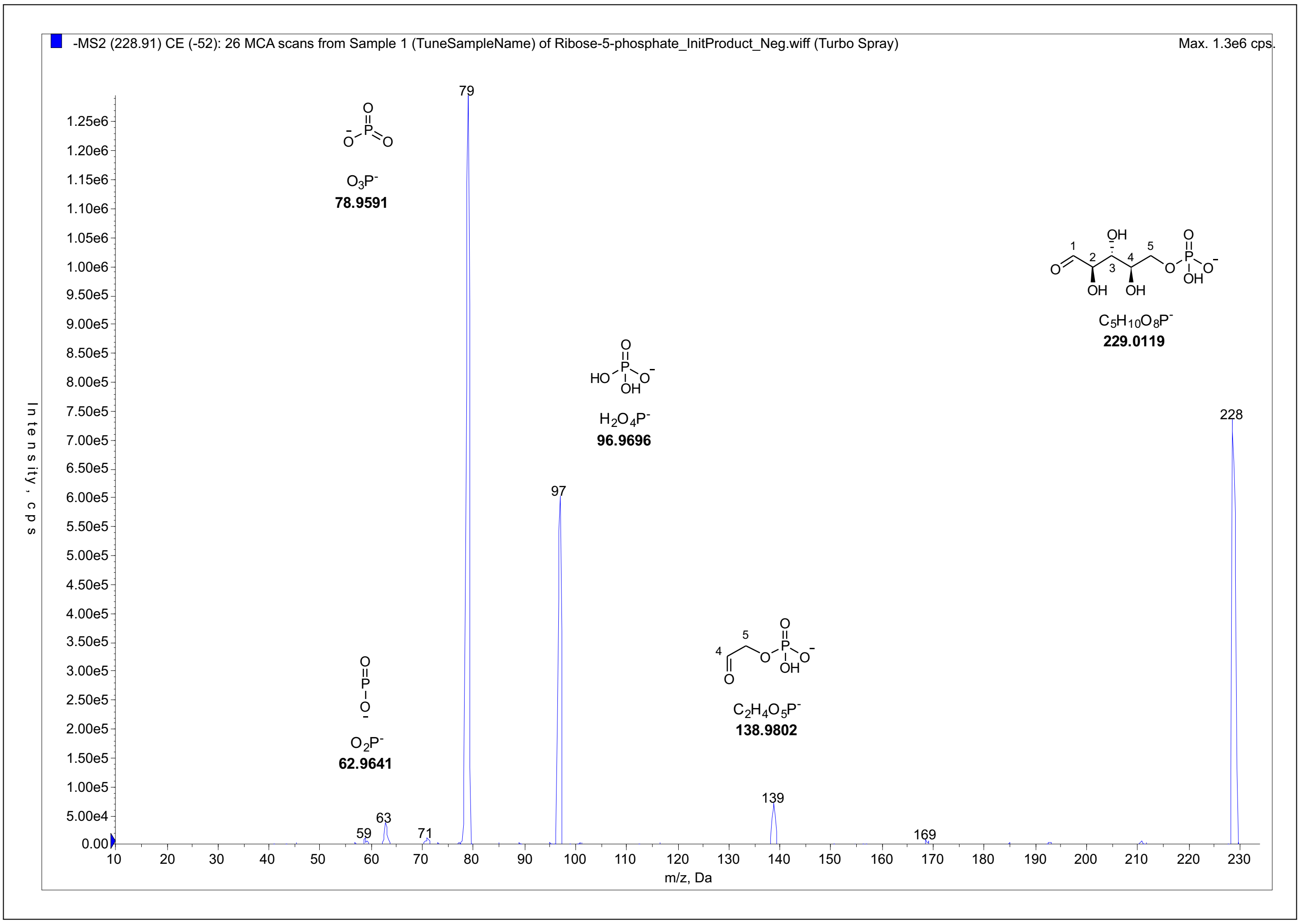

Chart S-7: The product ion spectrum of the [M-H]- ion of ribose 5-phosphate.

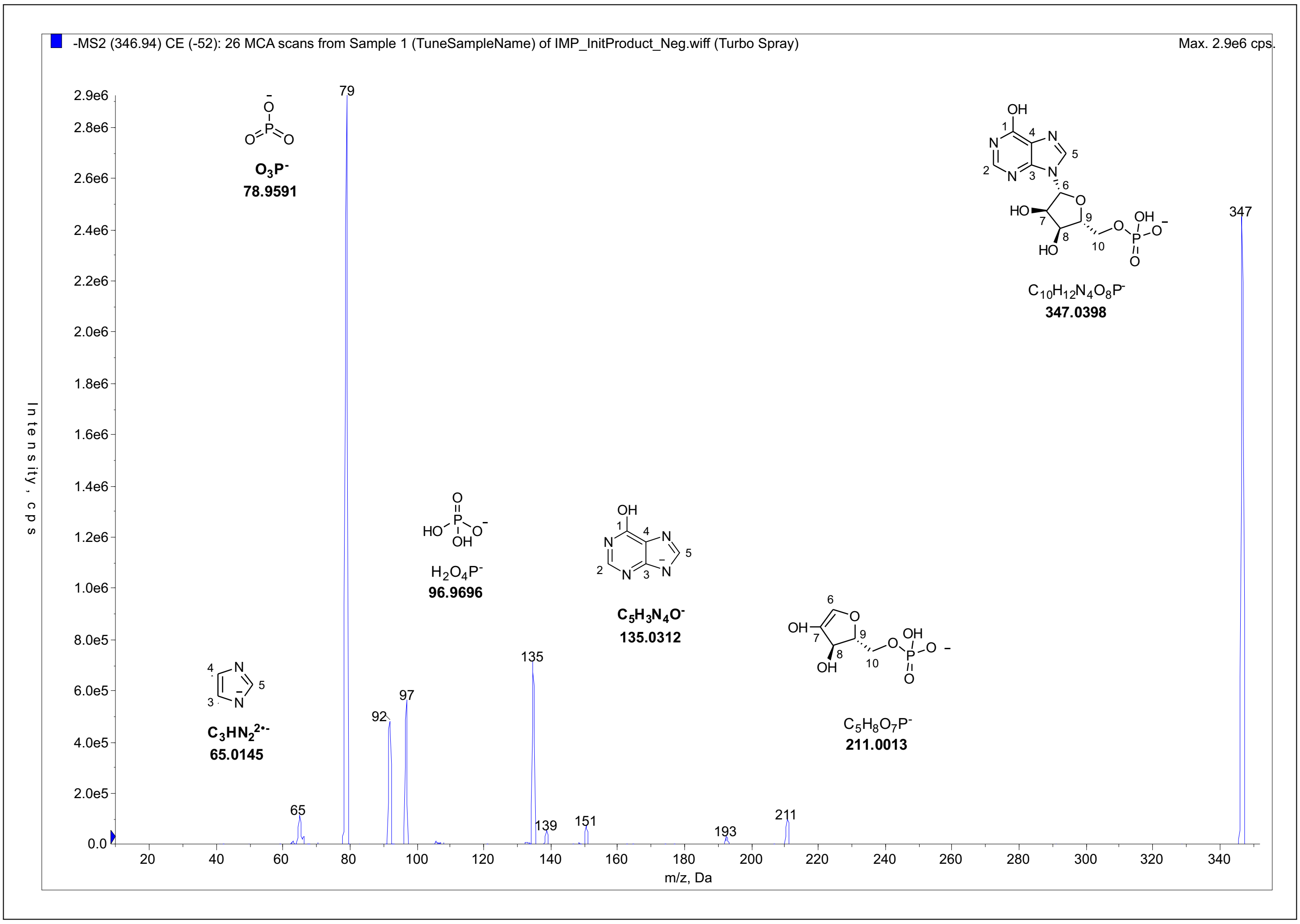

Chart S-8: The product ion spectrum of the [M-H]- ion of inosine 5-monophosphate.

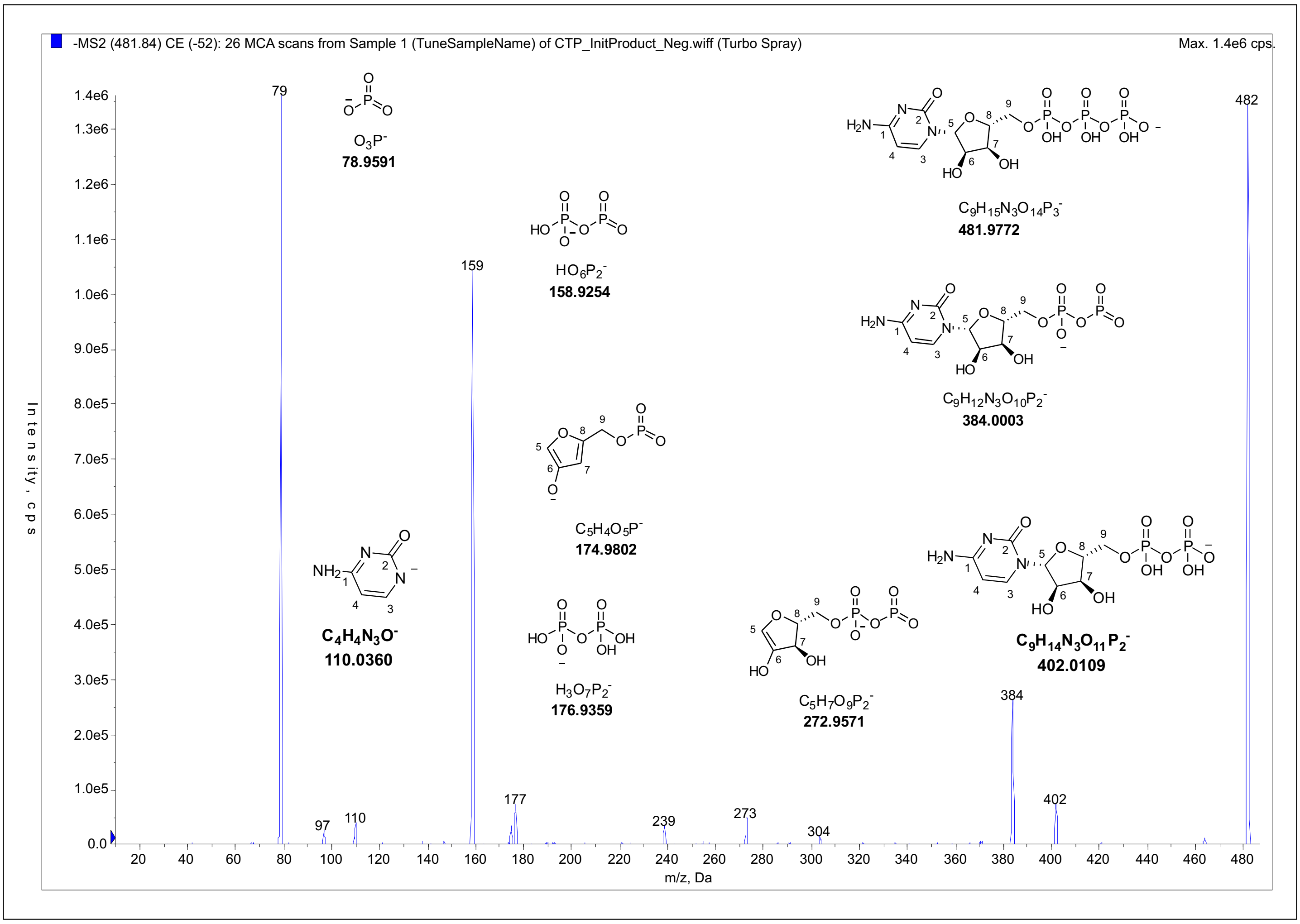

Chart S-9: The product ion spectrum of the [M-H]- ion of cytidine 5-triphosphate.

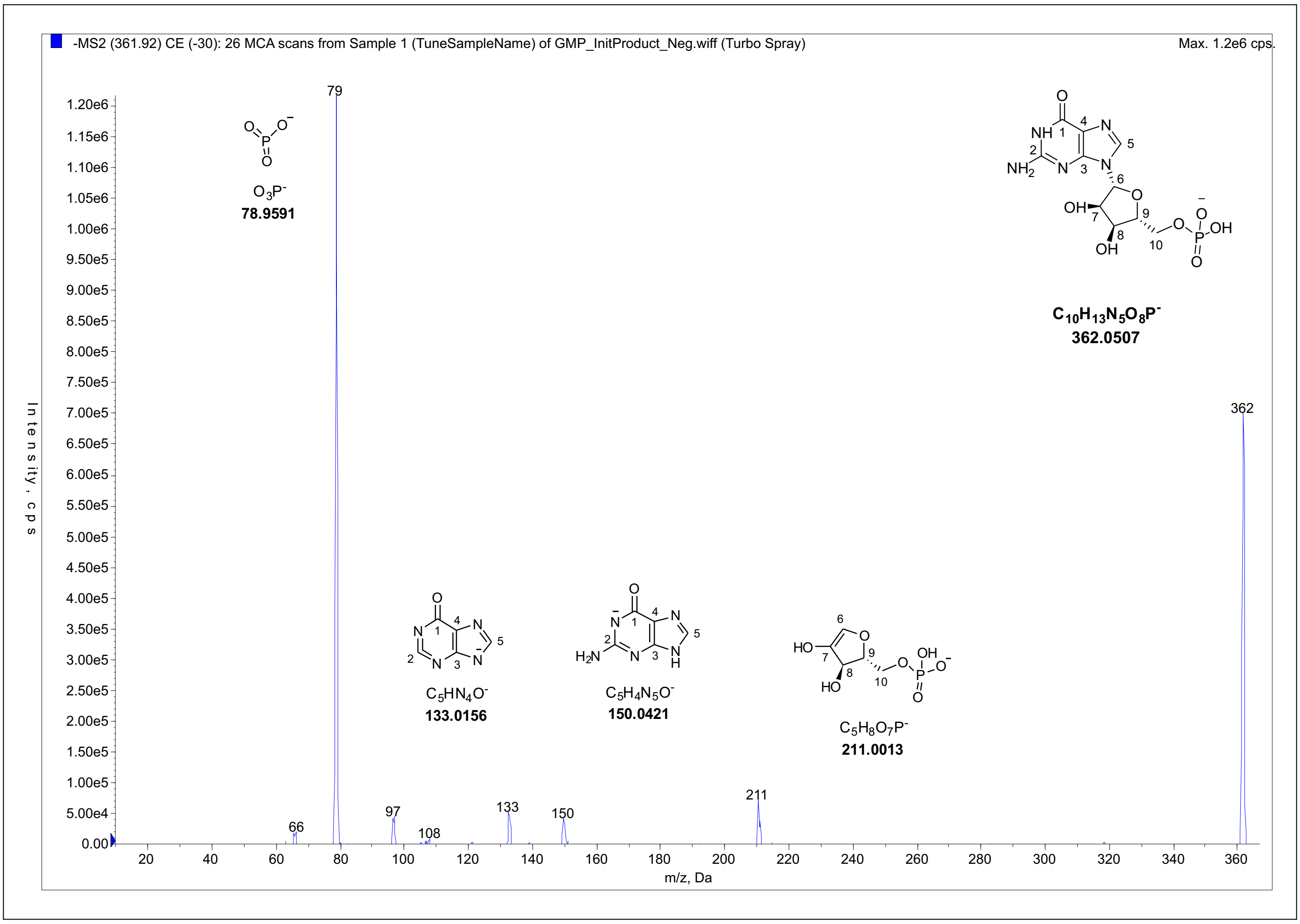

Chart S-10: TThe product ion spectrum of the [M-H]- ion of guanosine 5-monophosphate.

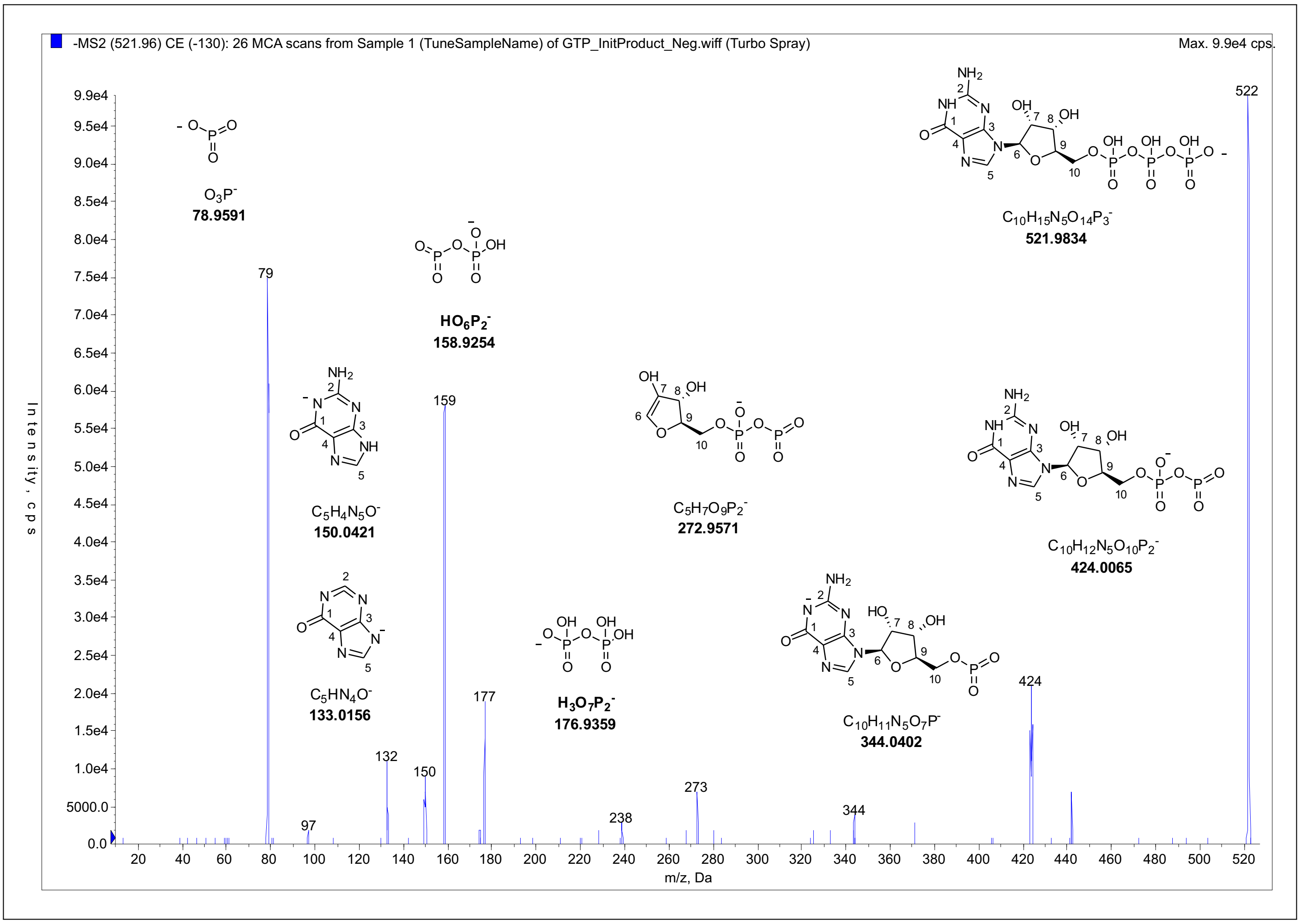

Chart S-11: The product ion spectrum of the [M-H]- ion of guanosine 5-triphosphate.

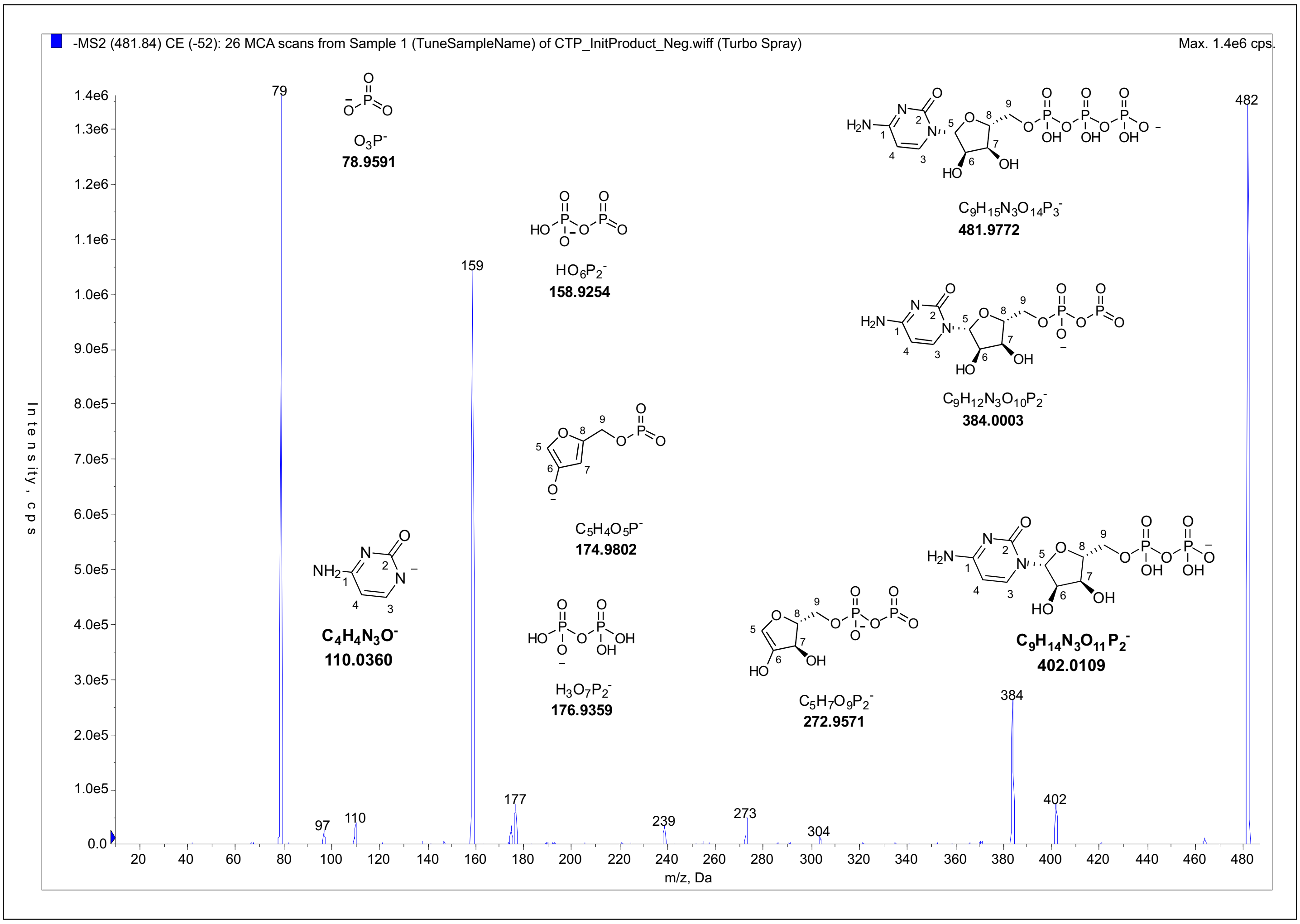

Chart S-12: The product ion spectrum of the [M-H]- ion of cytidine 5-triphosphate.

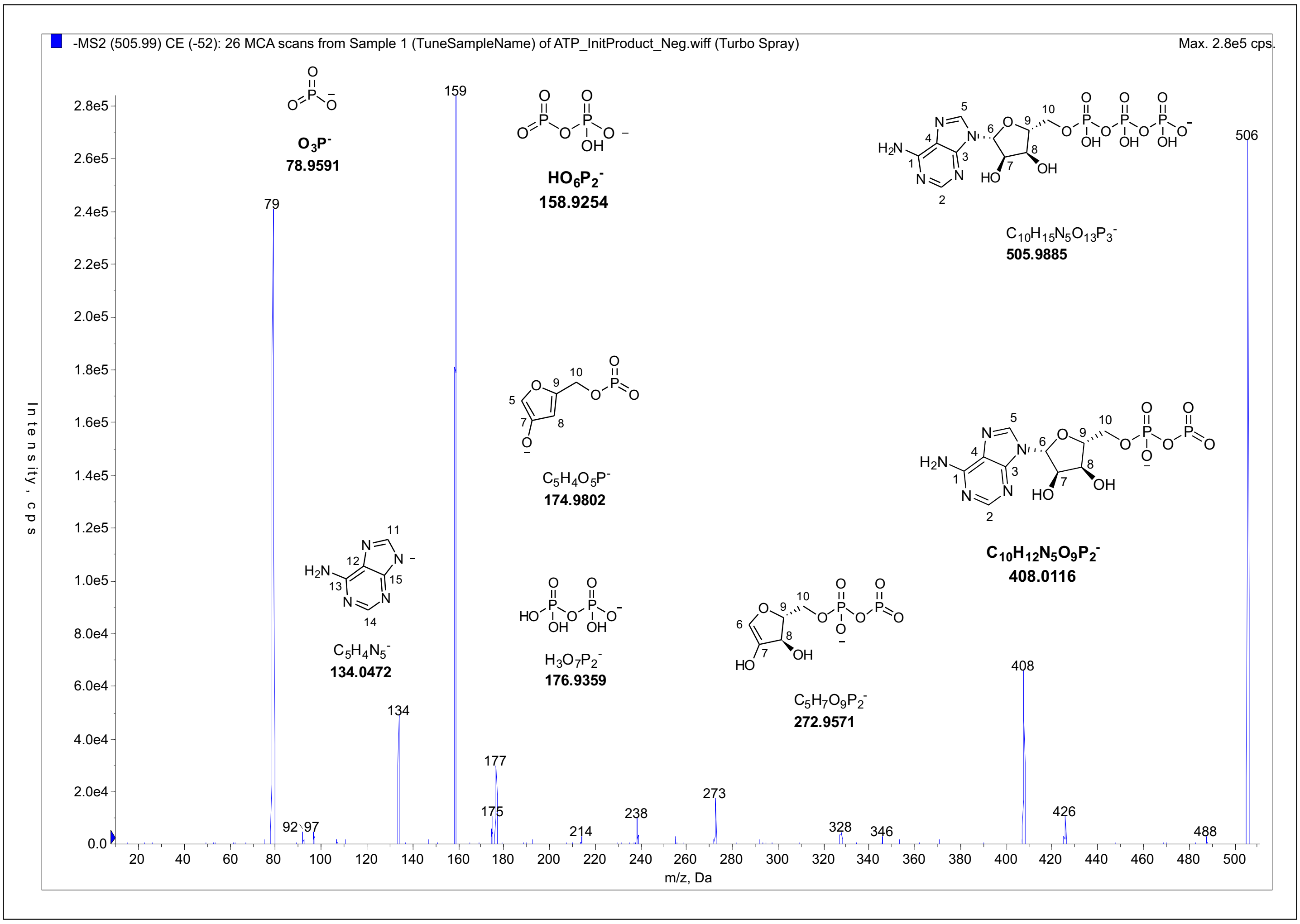

Chart S-13: The product ion spectrum of the [M-H]- ion of adenonosine 5-triphosphate.

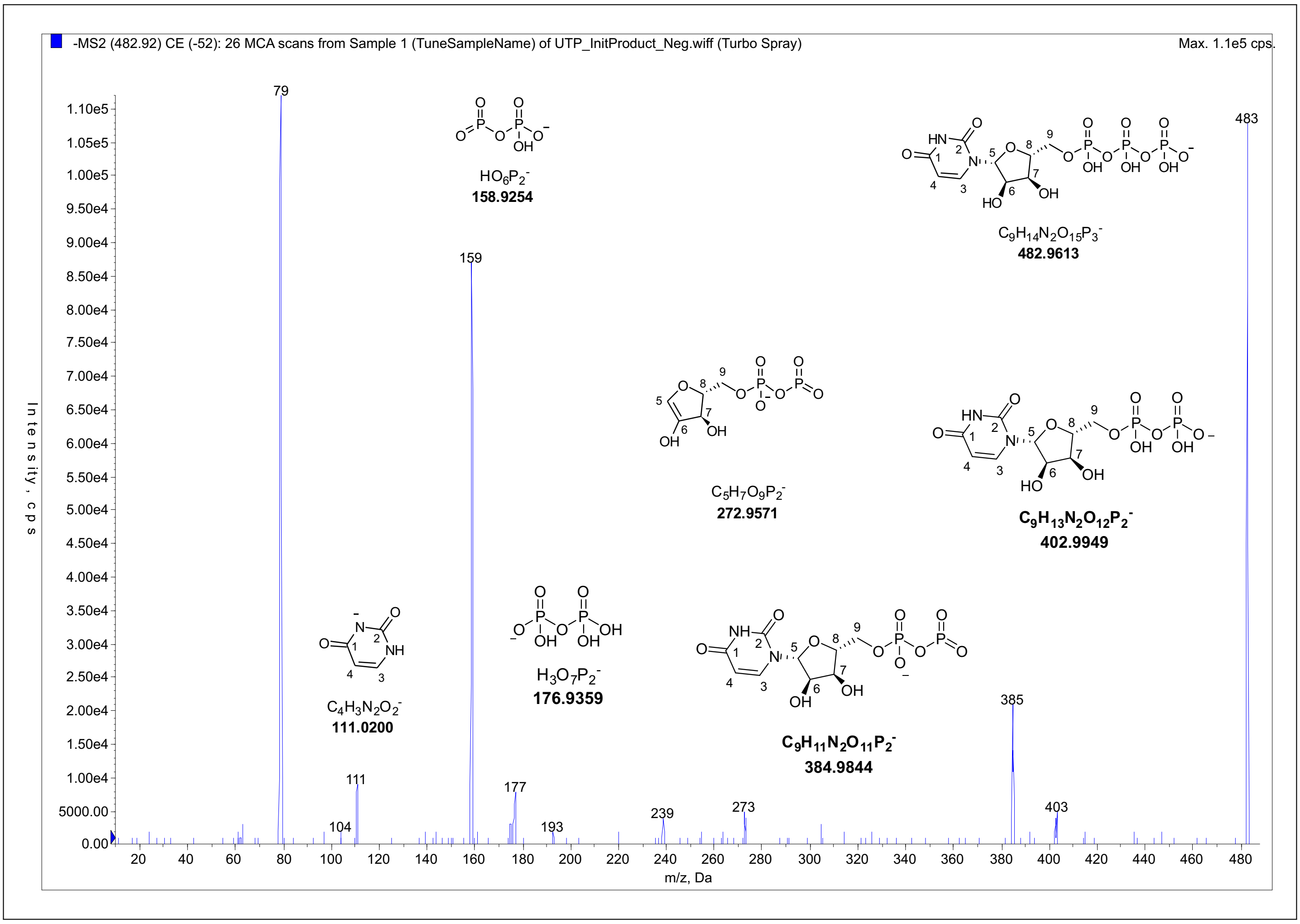

Chart S-14: The product ion spectrum of the [M-H]- ion of uridine 5-triphosphate.

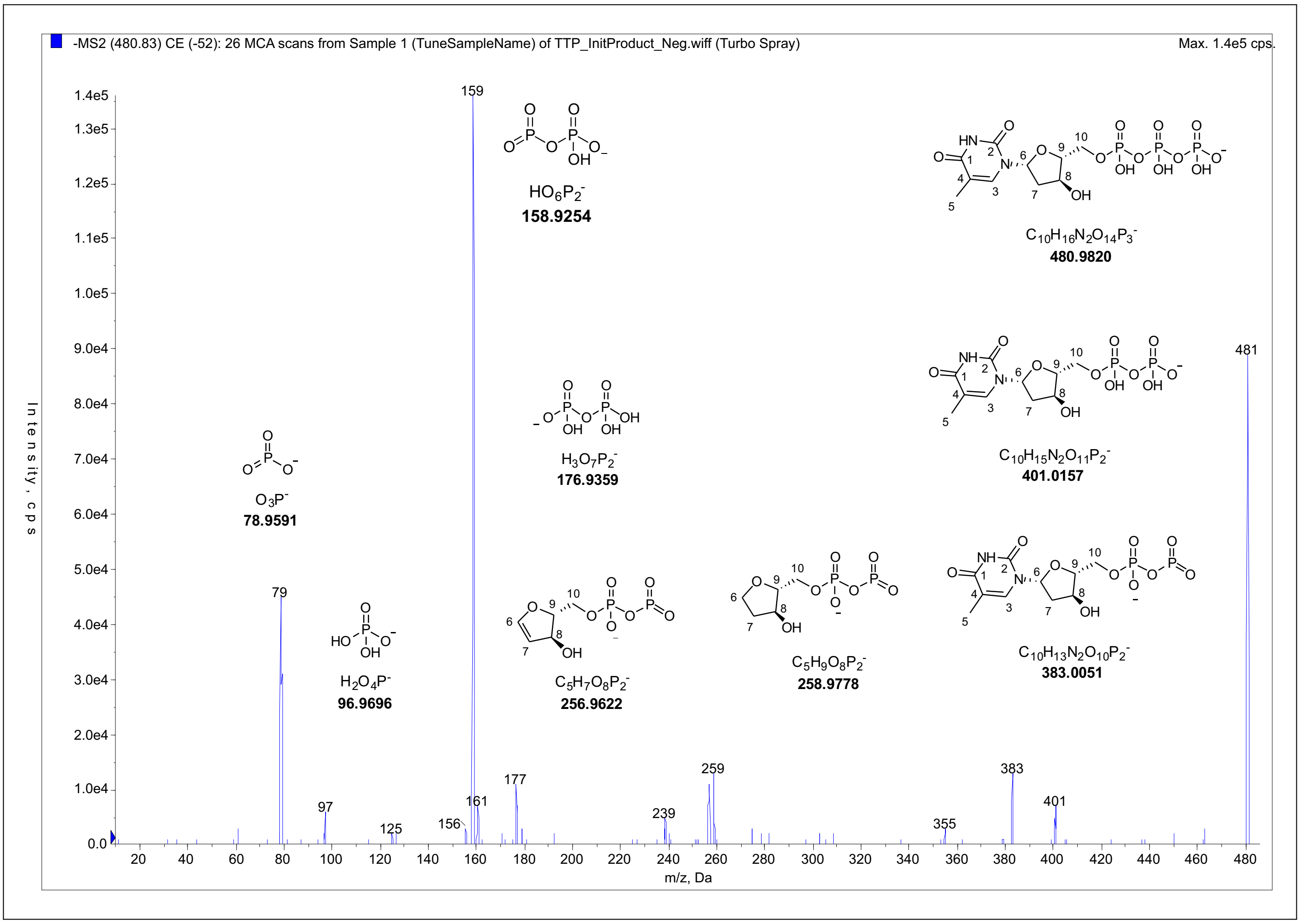

Chart S-15: The product ion spectrum of the [M-H]- ion of thymidine 5-triphosphate.

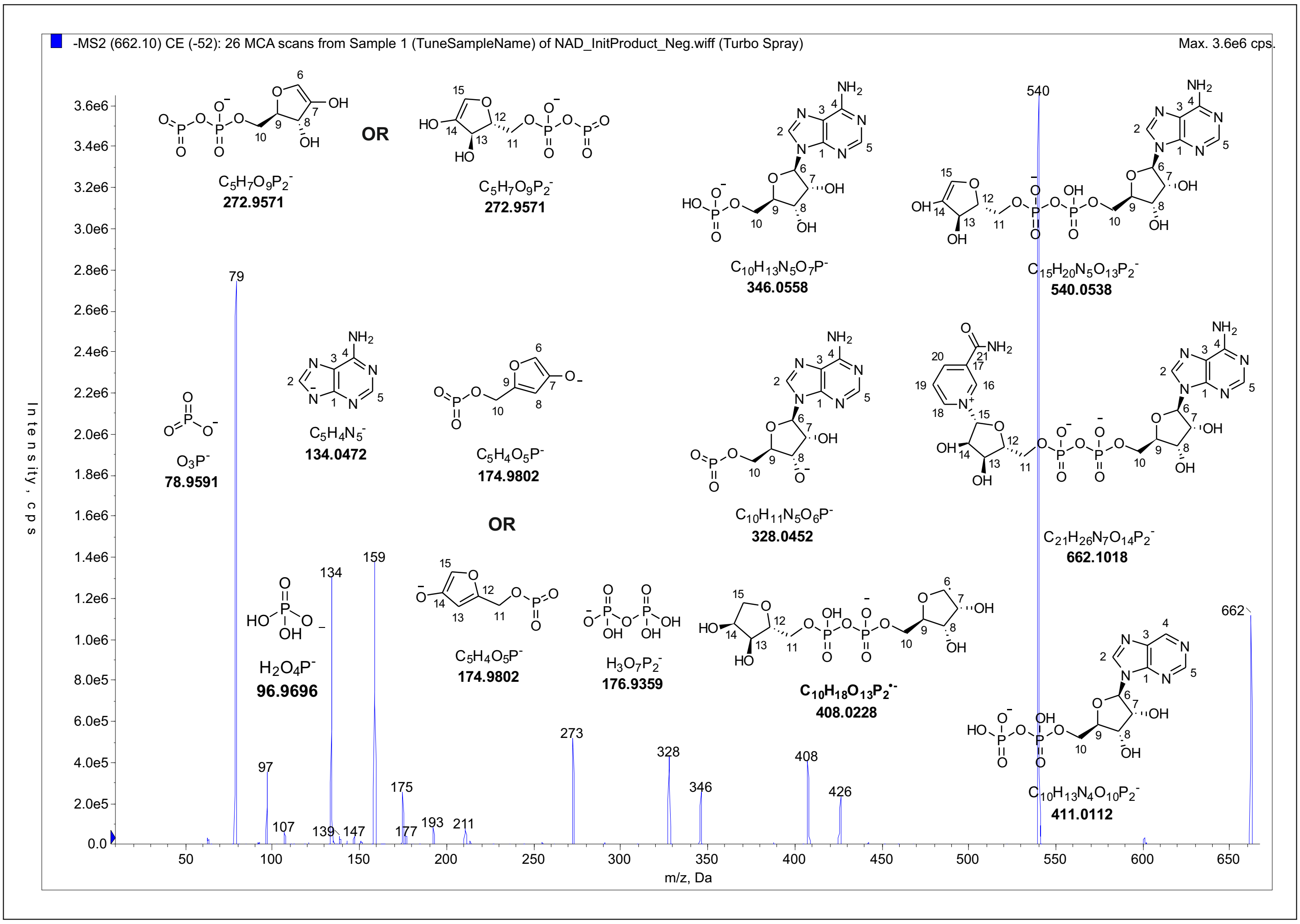

Chart S-16: The product ion spectrum of the [M-H]- ion of NAD.

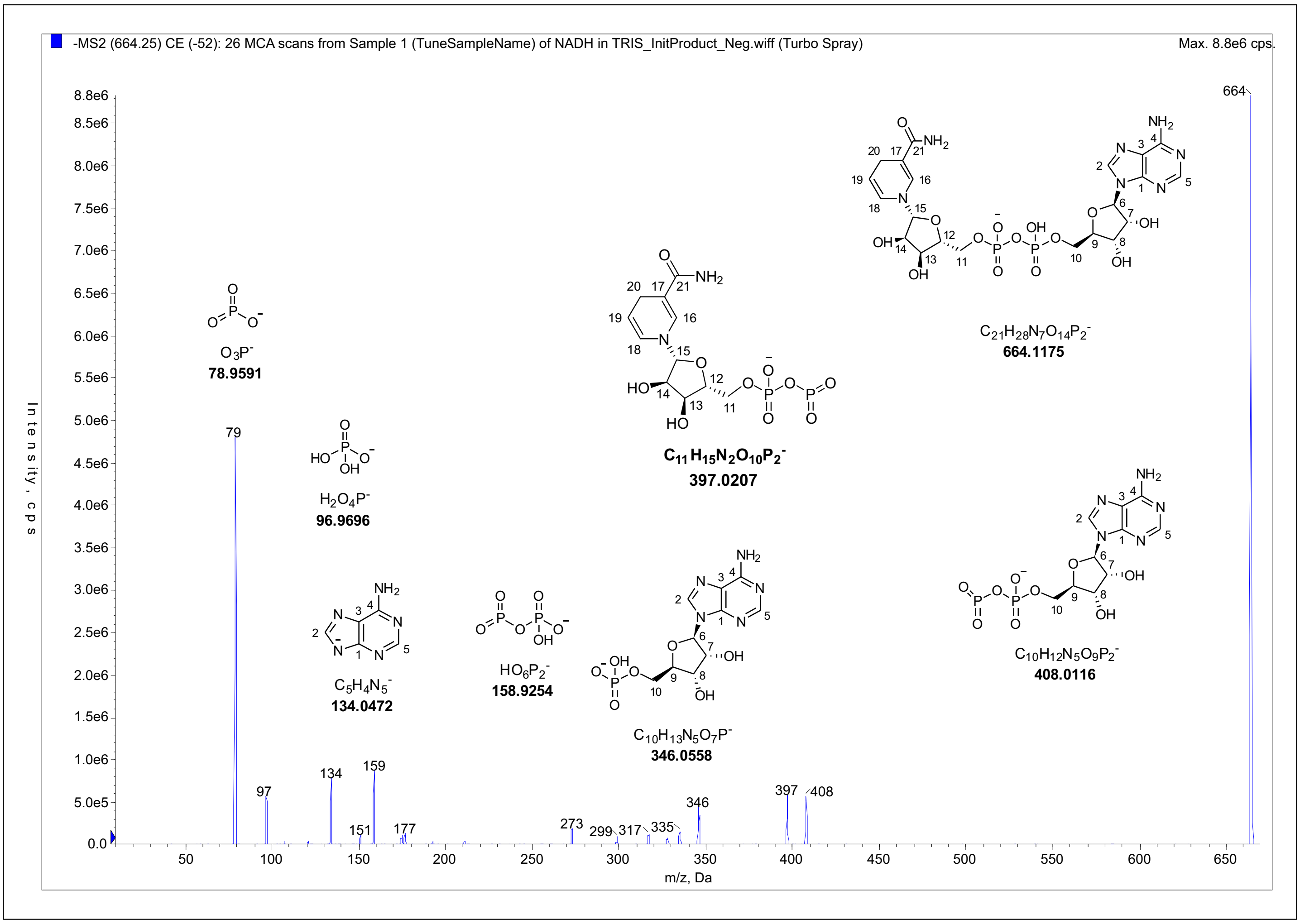

Chart S-17: The product ion spectrum of the [M-H]- ion of NADH.

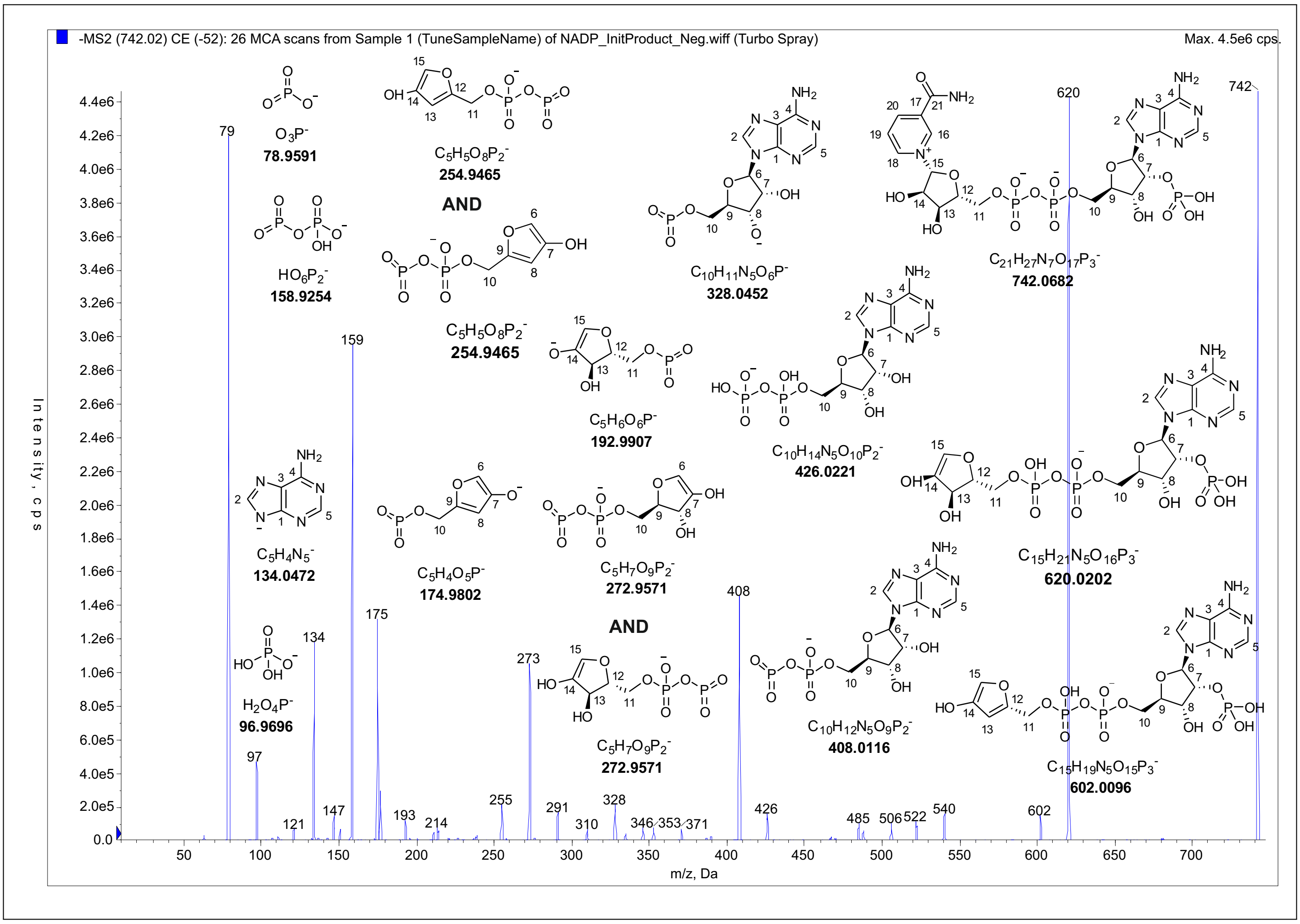

Chart S-18: The product ion spectrum of the [M-H]- ion of NADP.

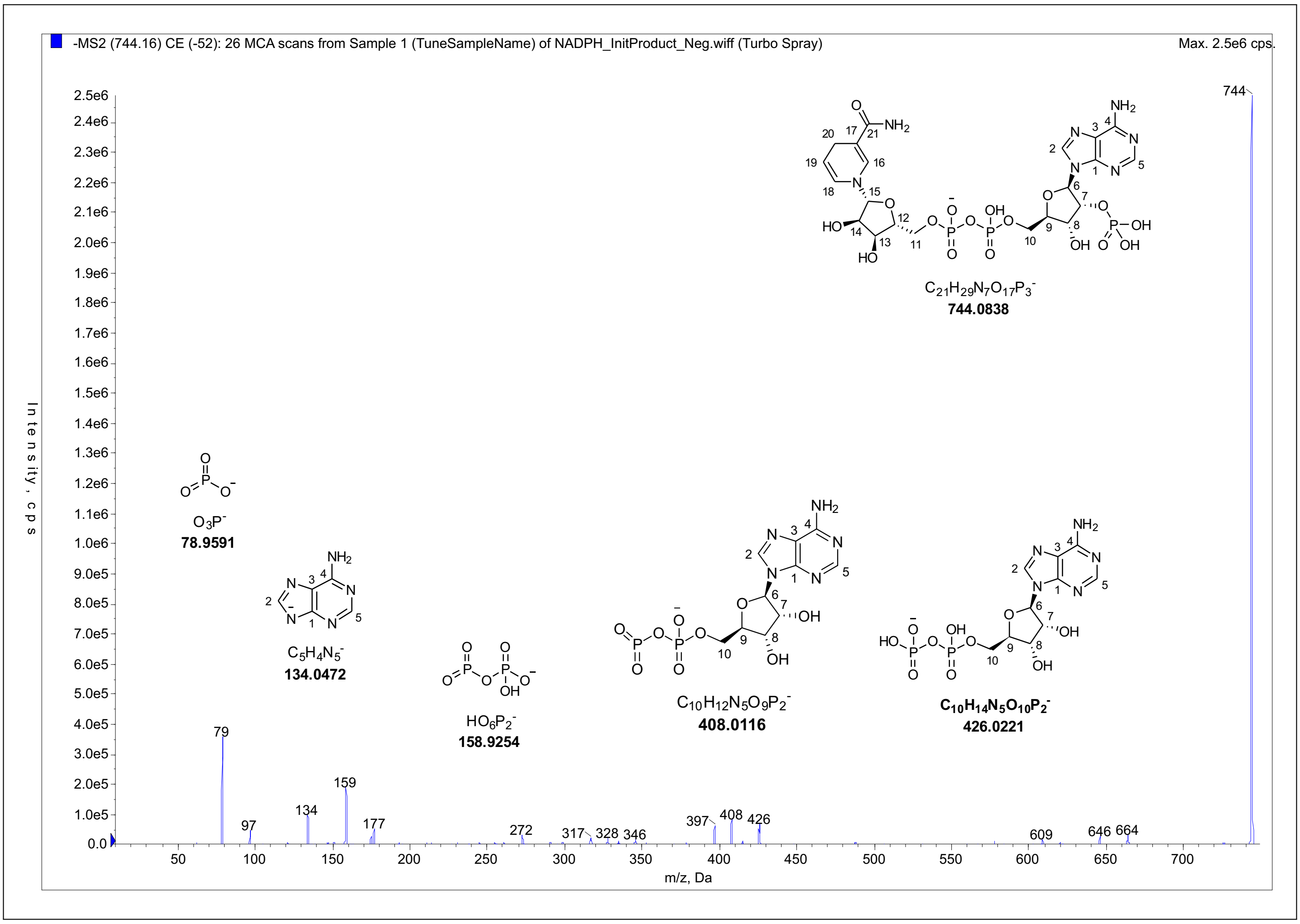

Chart S-19: The product ion spectrum of the [M-H]- ion of NADPH.

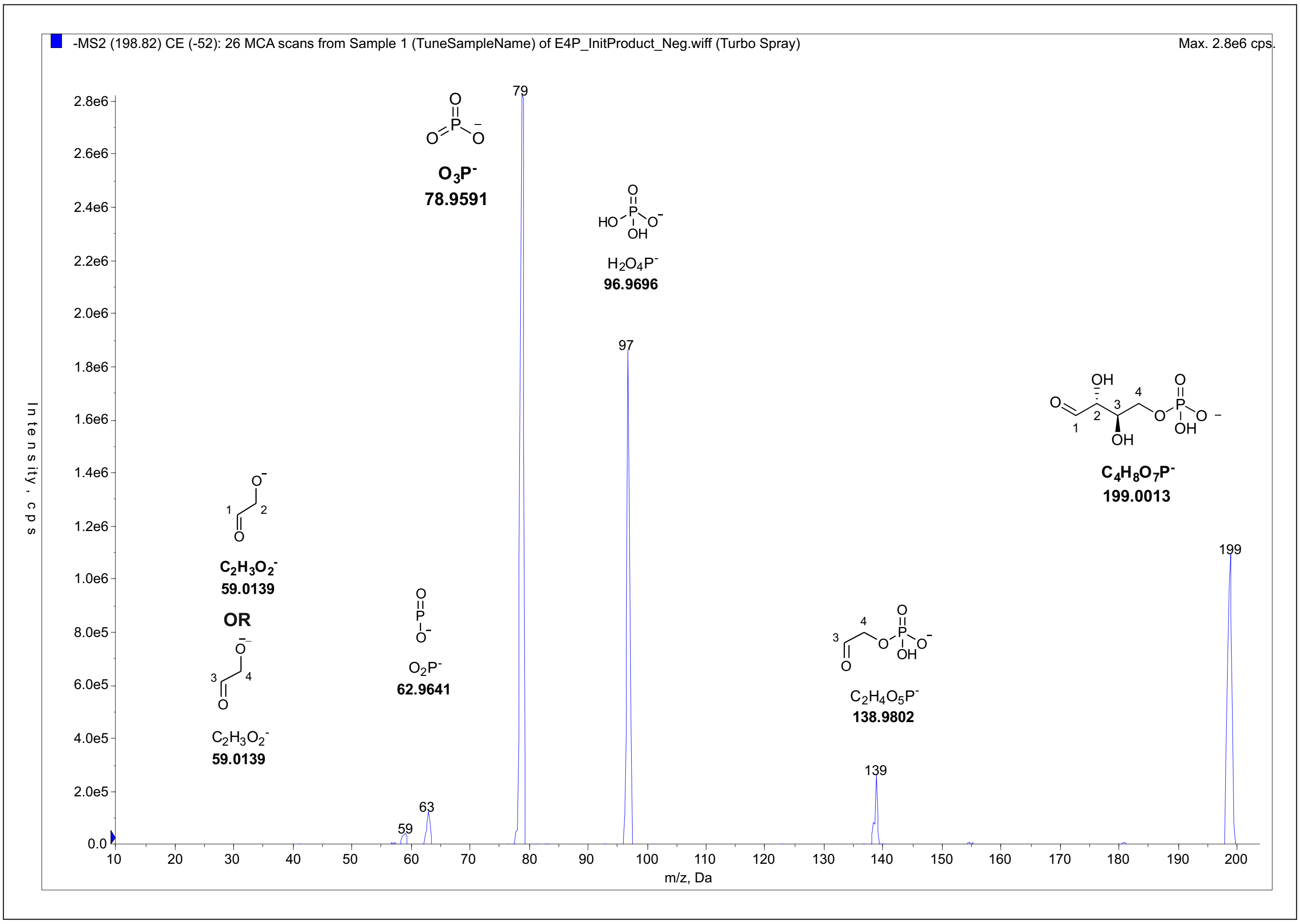

Chart S-20: The product ion spectrum of the [M-H]- ion of erythrose 4-phosphate.

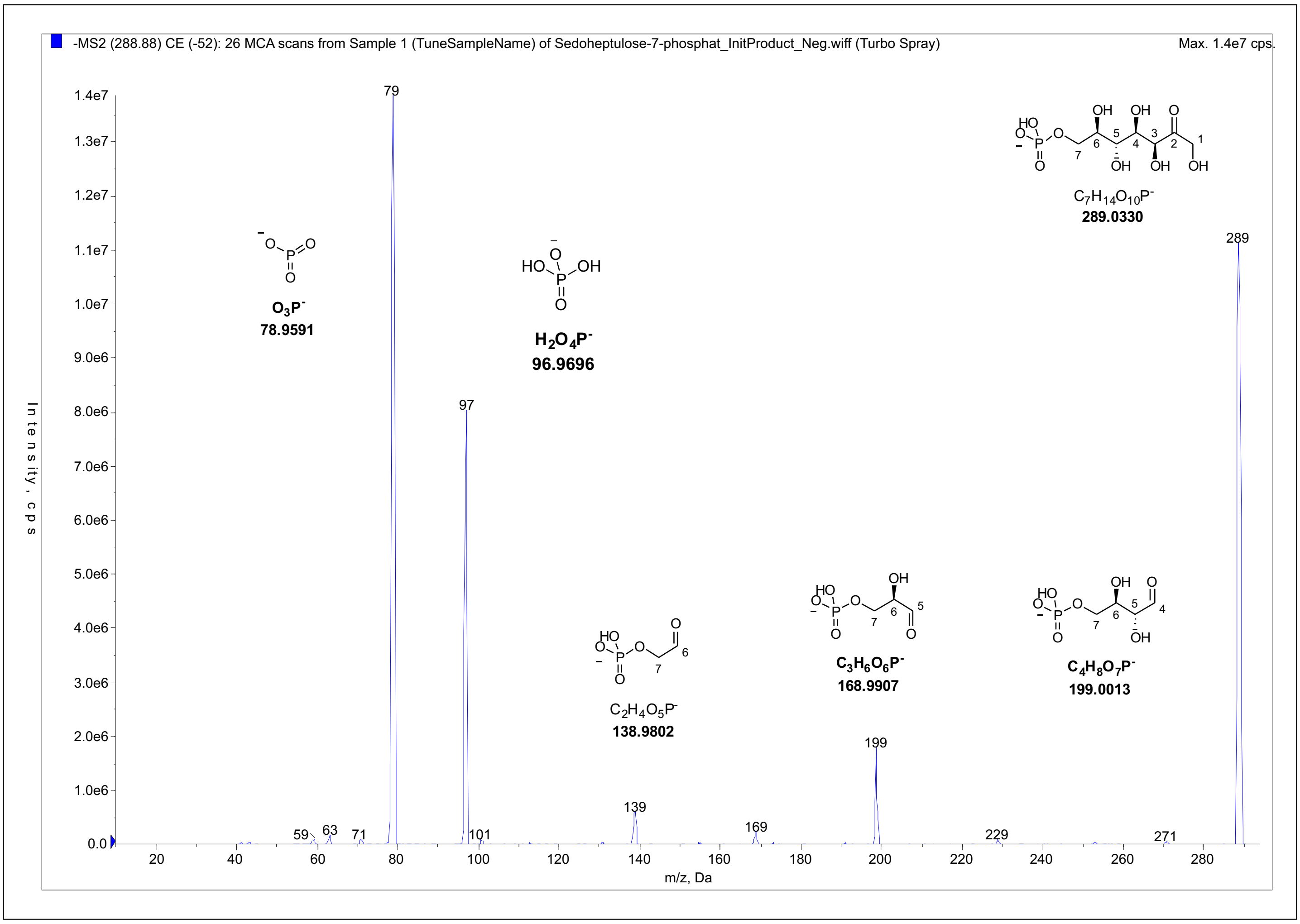

Chart S-21: The product ion spectrum of the [M-H]- ion of sedoheptulose 7-phosphate.

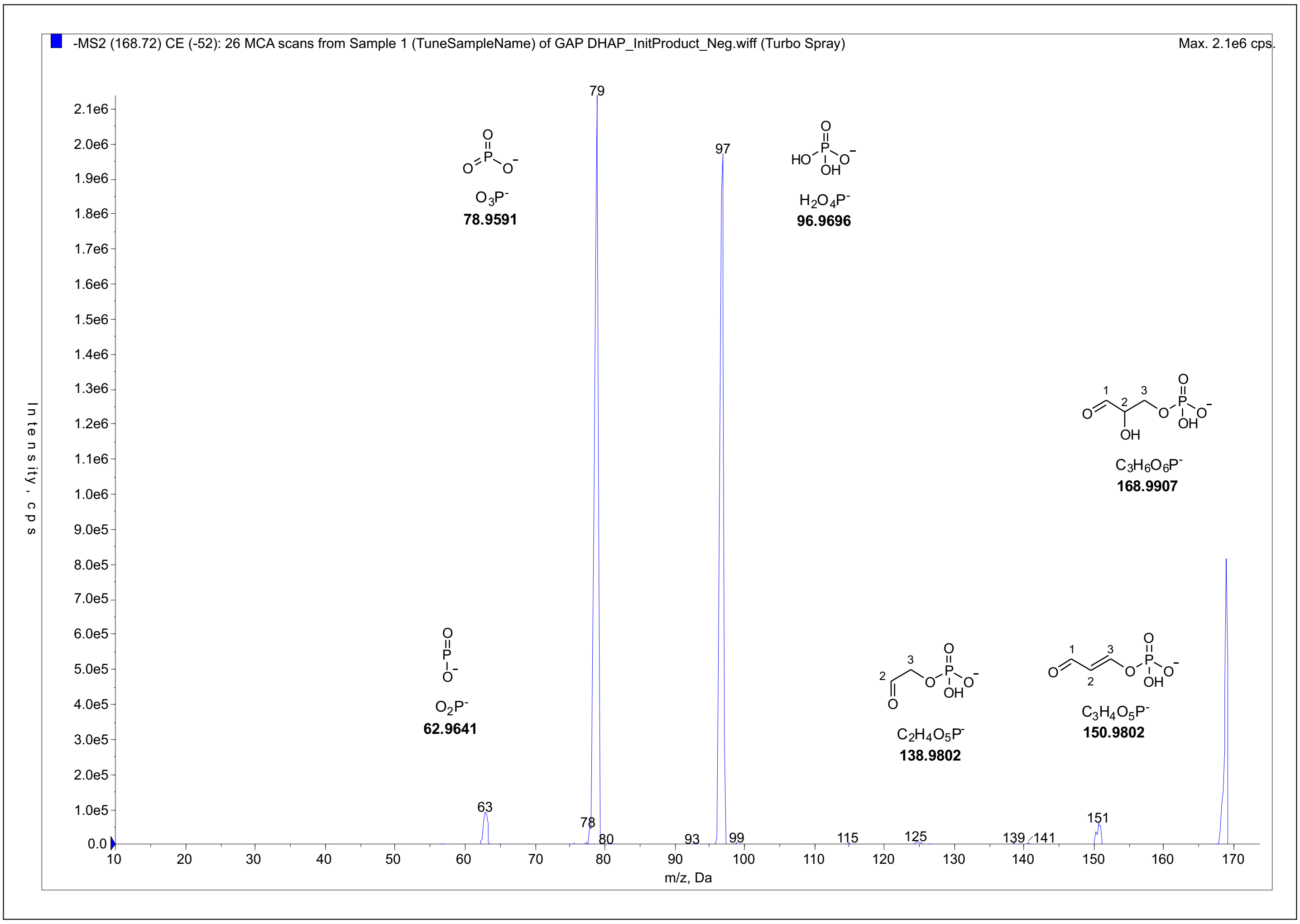

Chart S-22: The product ion spectrum of the [M-H]- ion of glyceraldehyde 3-phosphate.

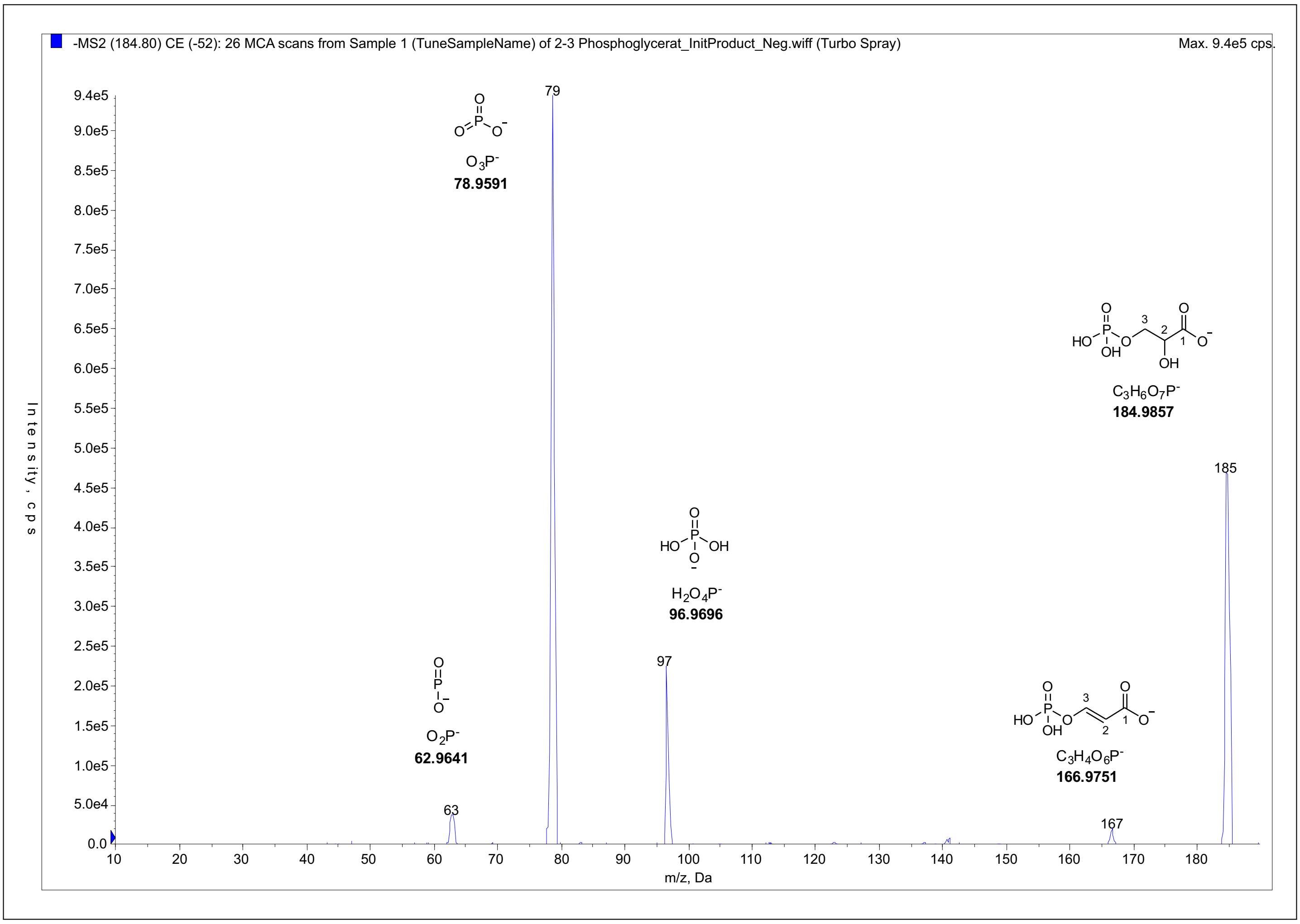

Chart S-23: The product ion spectrum of the [M-H]- ion of 3-phosphoglycerate.

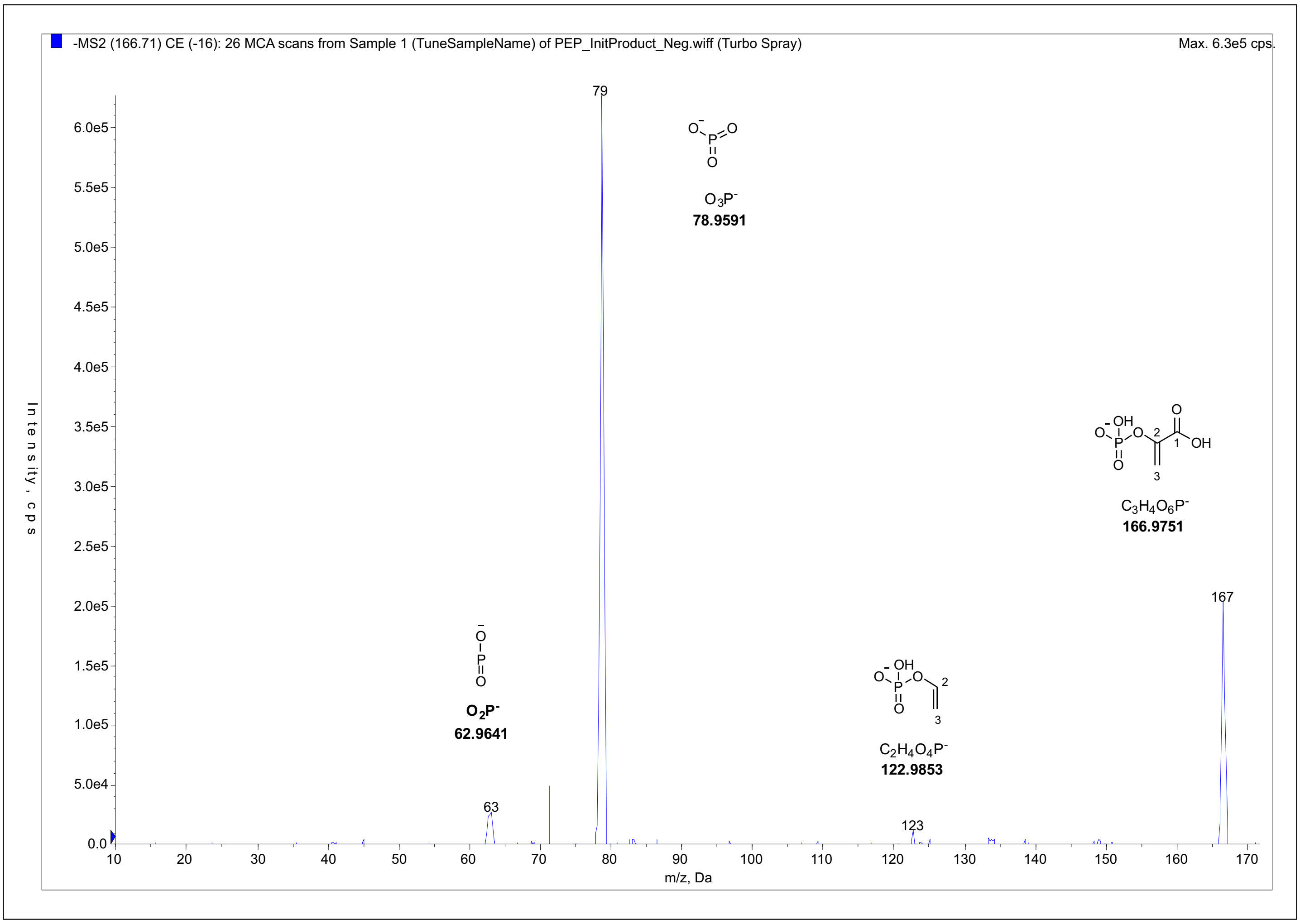

Chart S-24: The product ion spectrum of the [M-H]- ion of phosphoenolpyruvate.

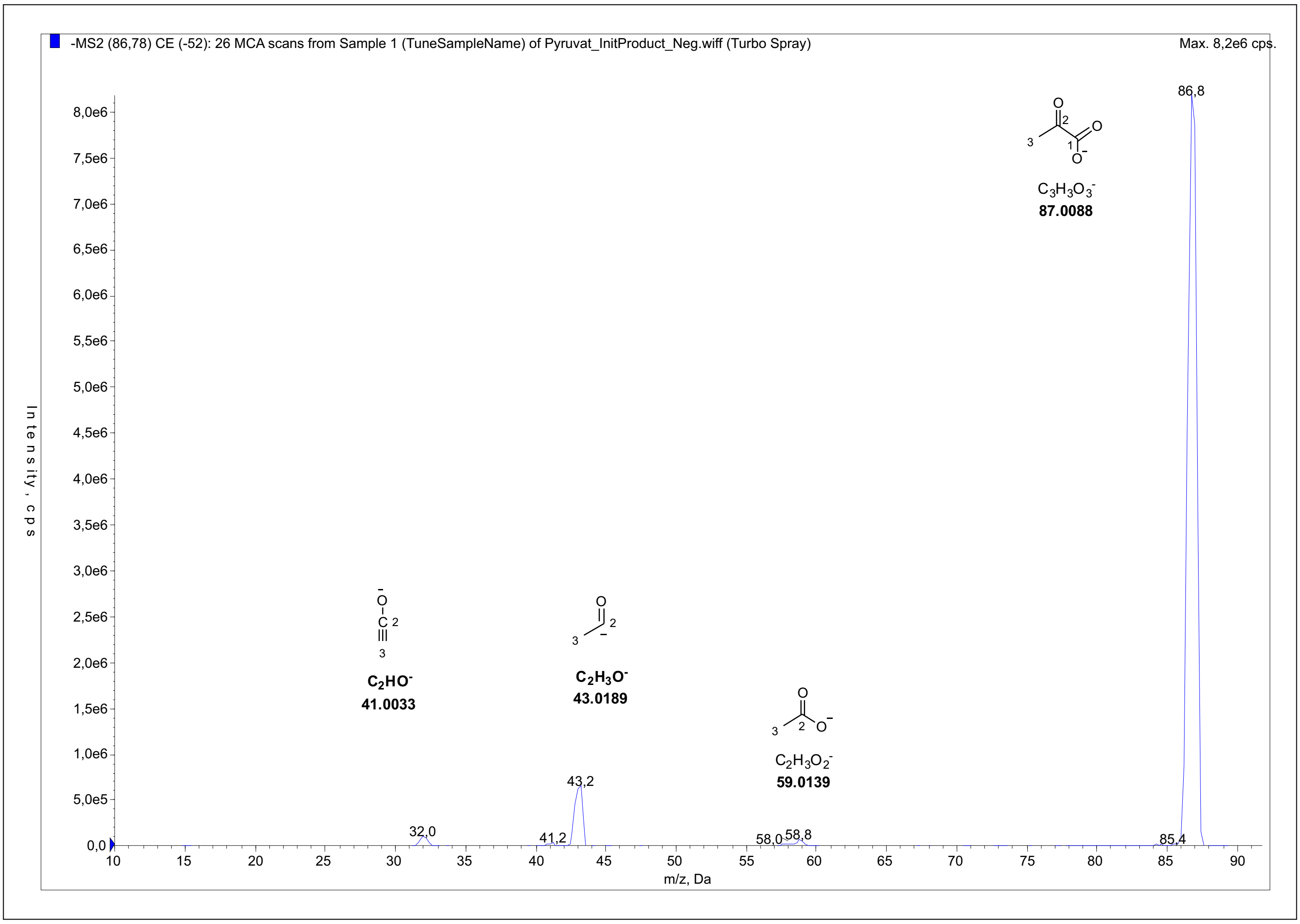

Chart S-25: The product ion spectrum of the [M-H]- ion of pyruvate.

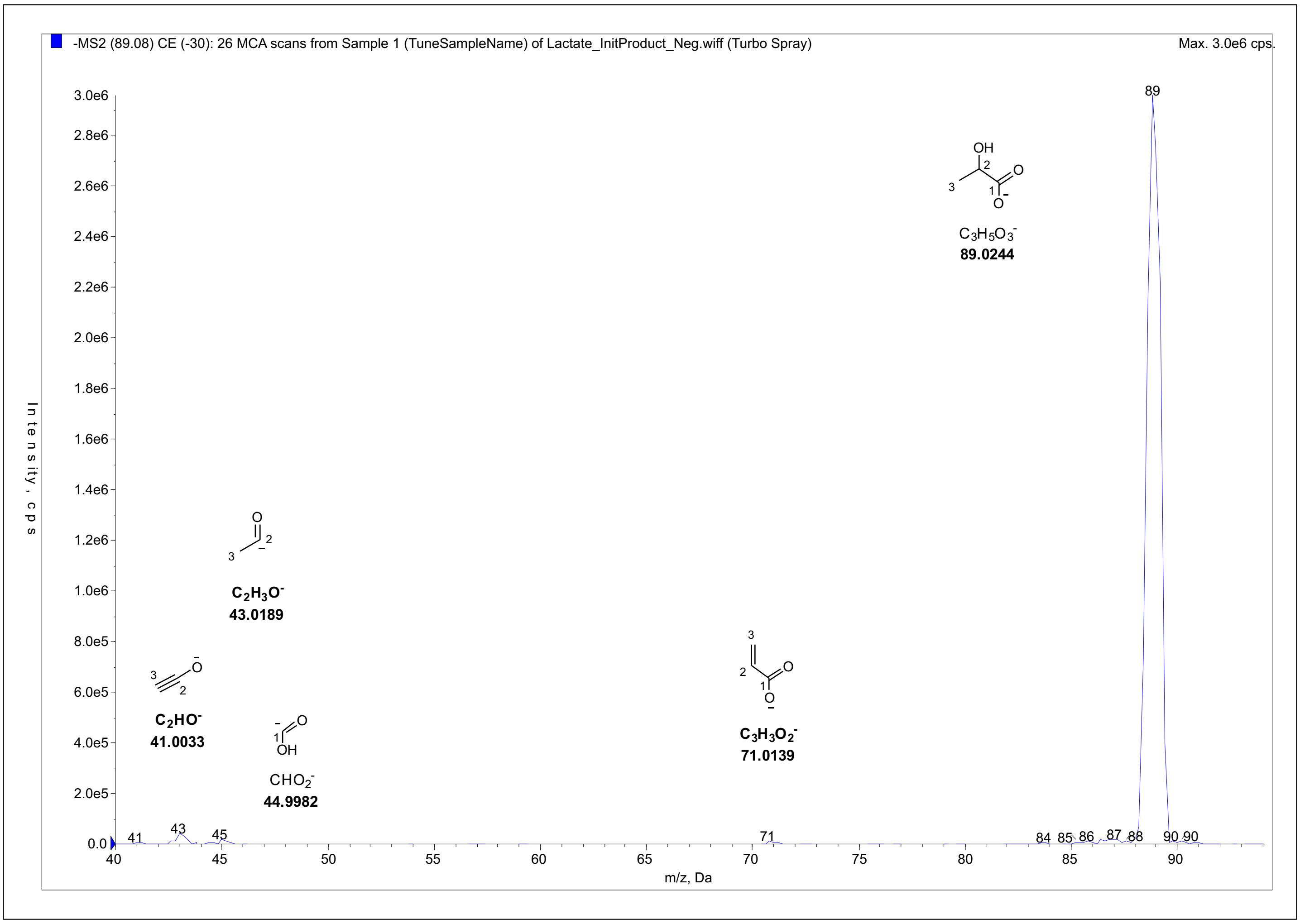

Chart S-26: The product ion spectrum of the [M-H]- ion of lactate.

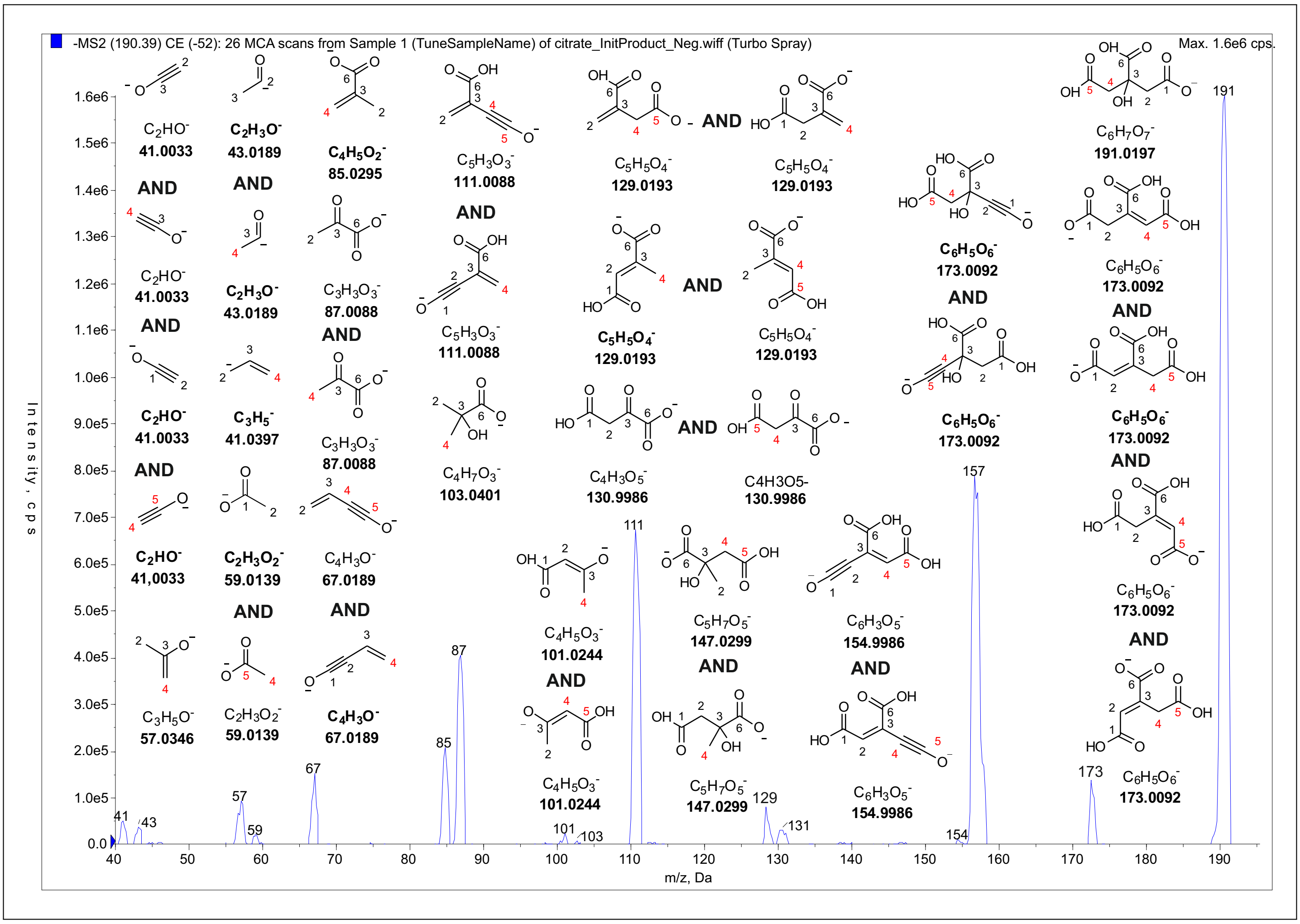

Chart S-27: The product ion spectrum of the [M-H]- ion of citrate.

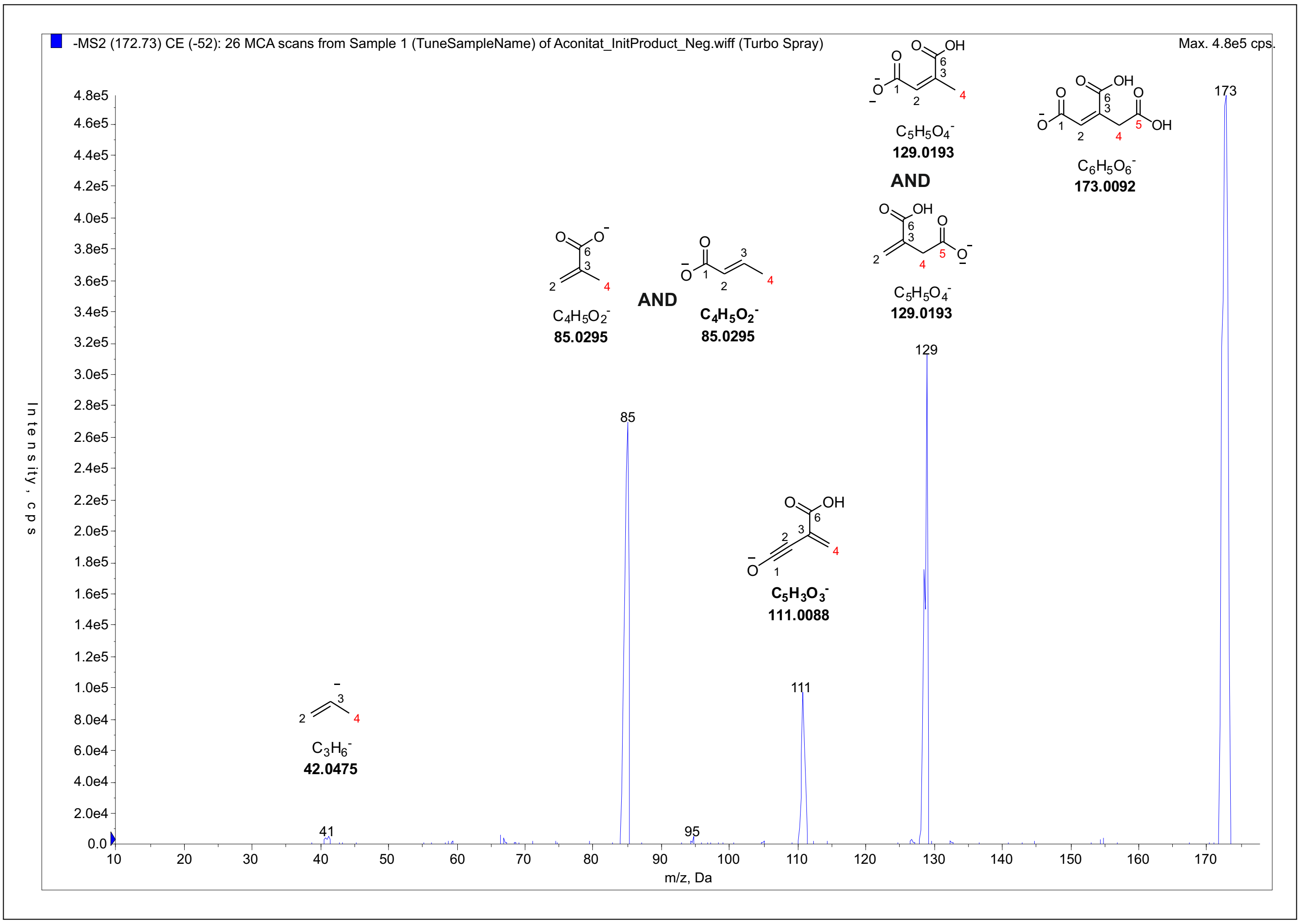

Chart S-28: The product ion spectrum of the [M-H]- ion of cis-aconitate.

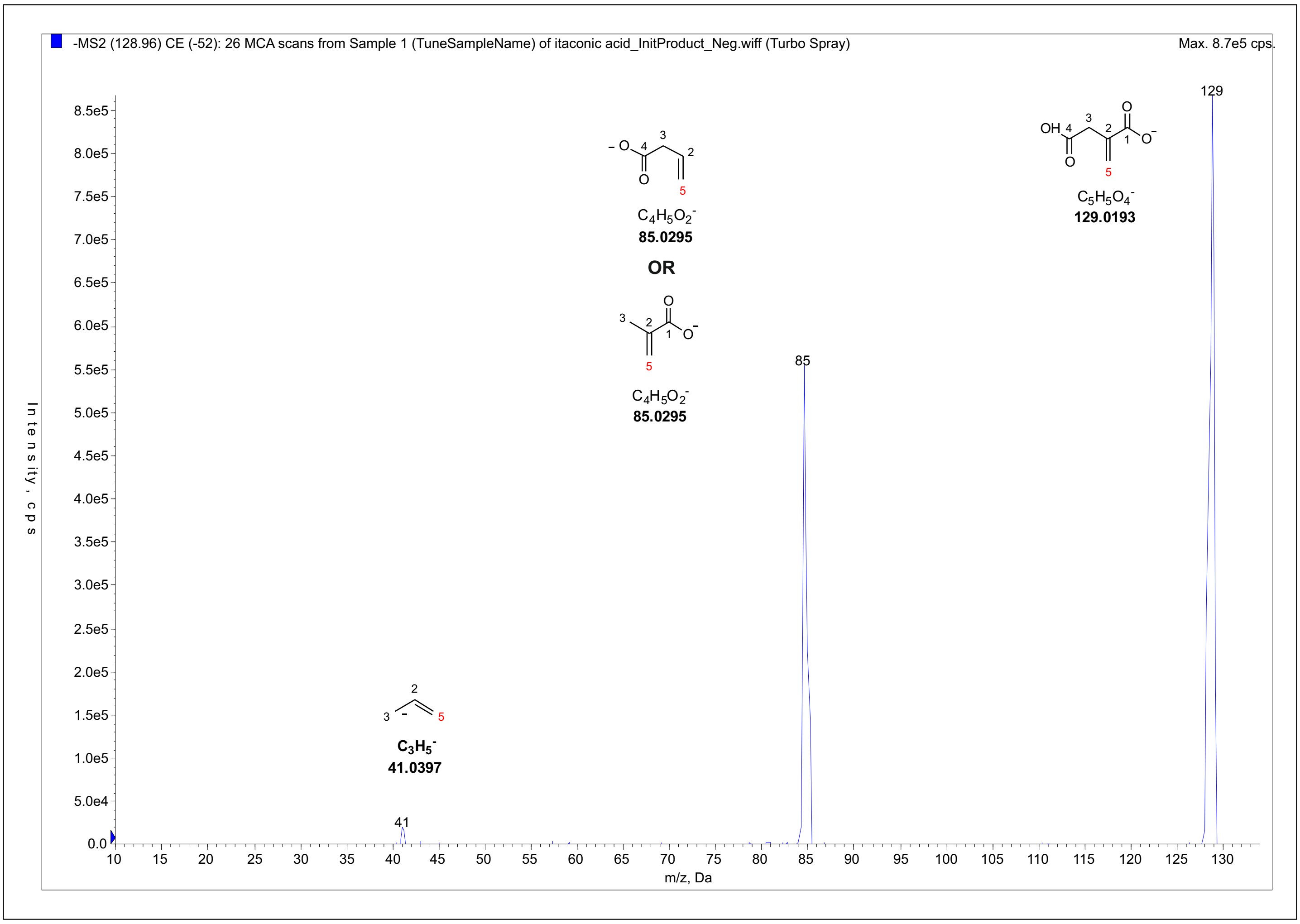

Chart S-29: The product ion spectrum of the [M-H]- ion of itaconate.

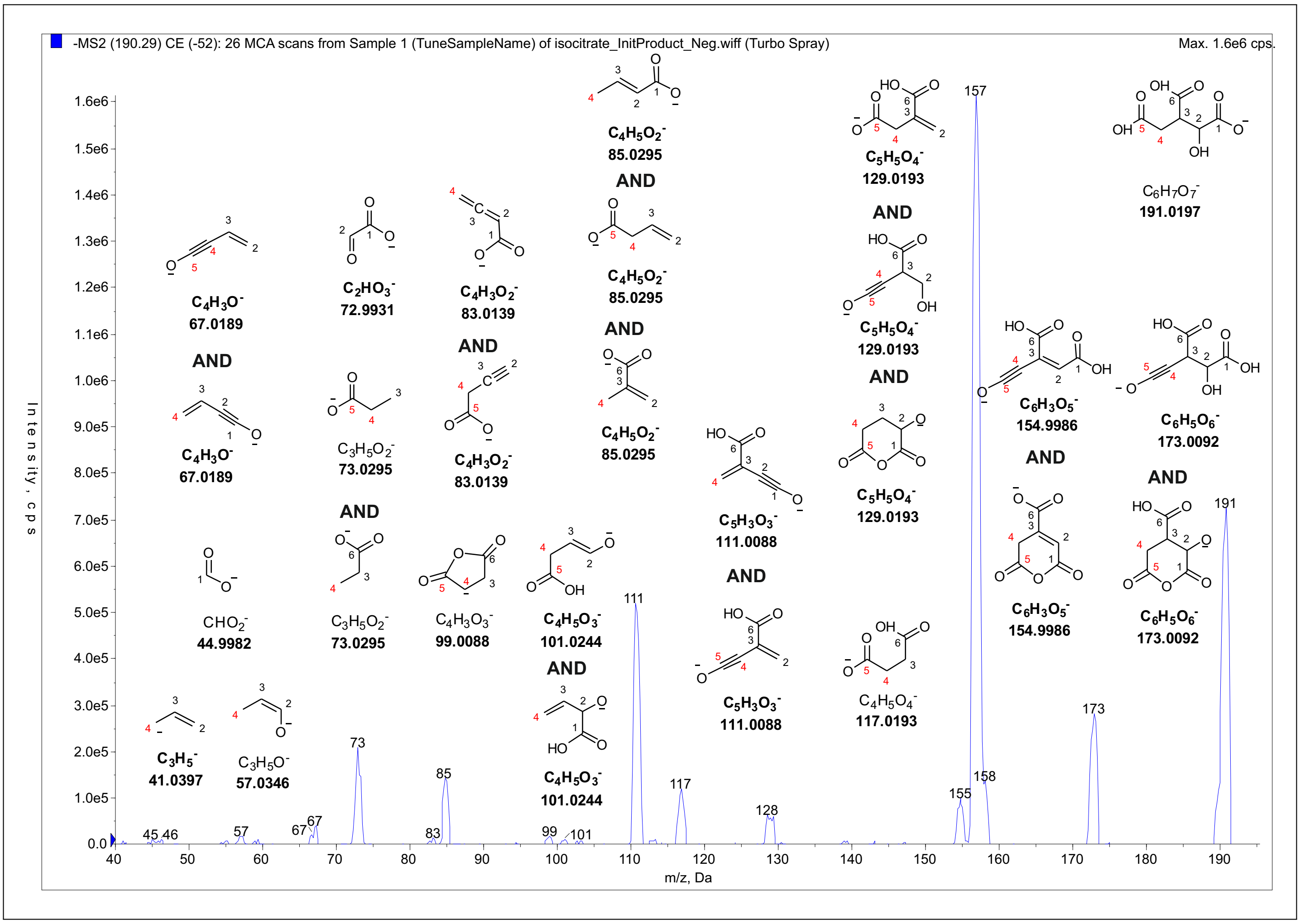

Chart S-30: The product ion spectrum of the [M-H]- ion of isocitrate. Contrary to citric acid no oxaloactetate ion at m/z=131 is formed through elimination of acetic acid. Therefore, the fragment ions of oxaloacetic acid are missing in the isocitrate mass spectrum, i.e. m/z=87 and 59. The missing pyruvate anion, being derived from the oxaloacetic acid fragmentation, results in the absence of the m/z=43 ion as the most intense ion of pyruvate.

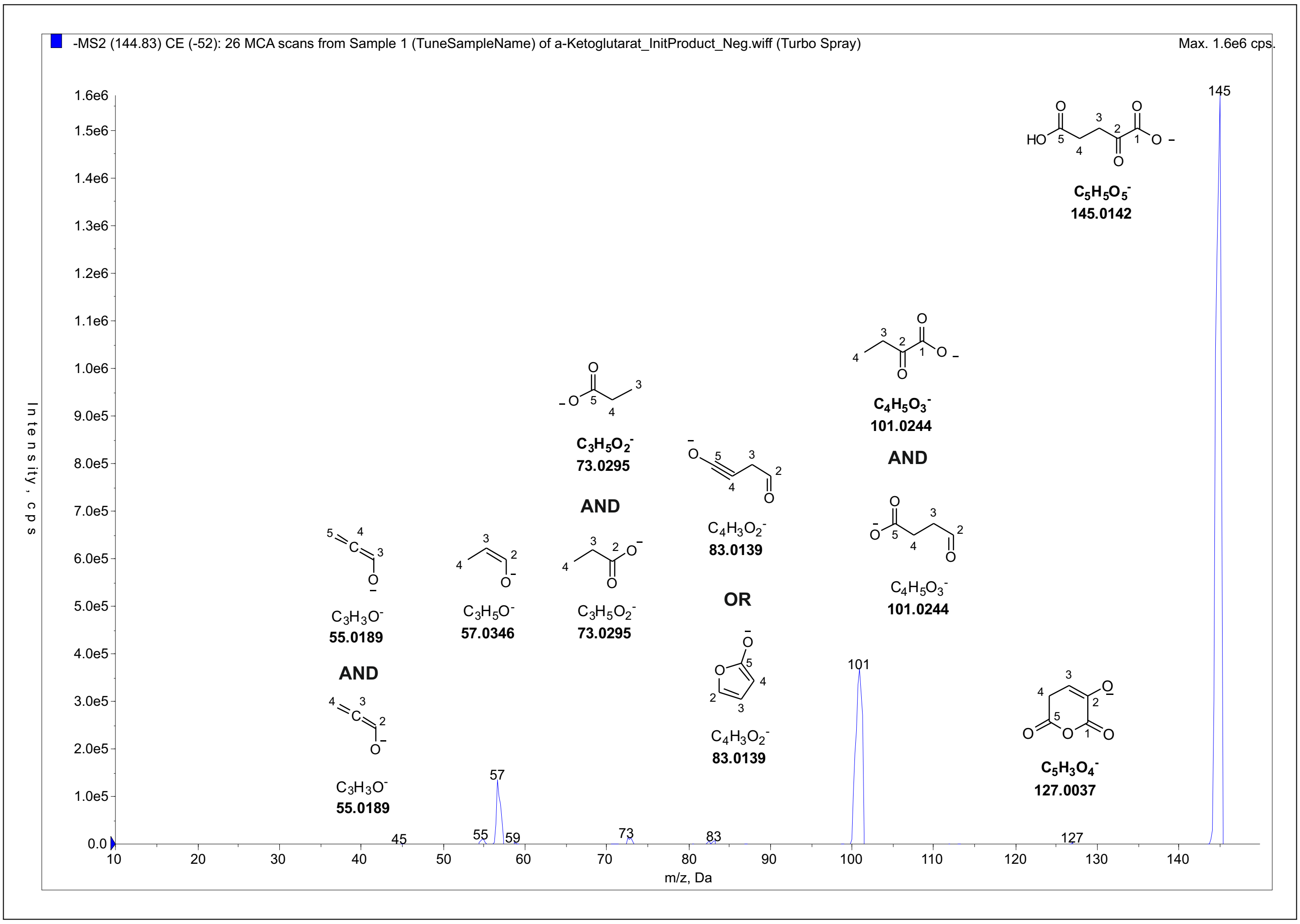

Chart S-31: The product ion spectrum of the [M-H]- ion of α-ketoglutarate.

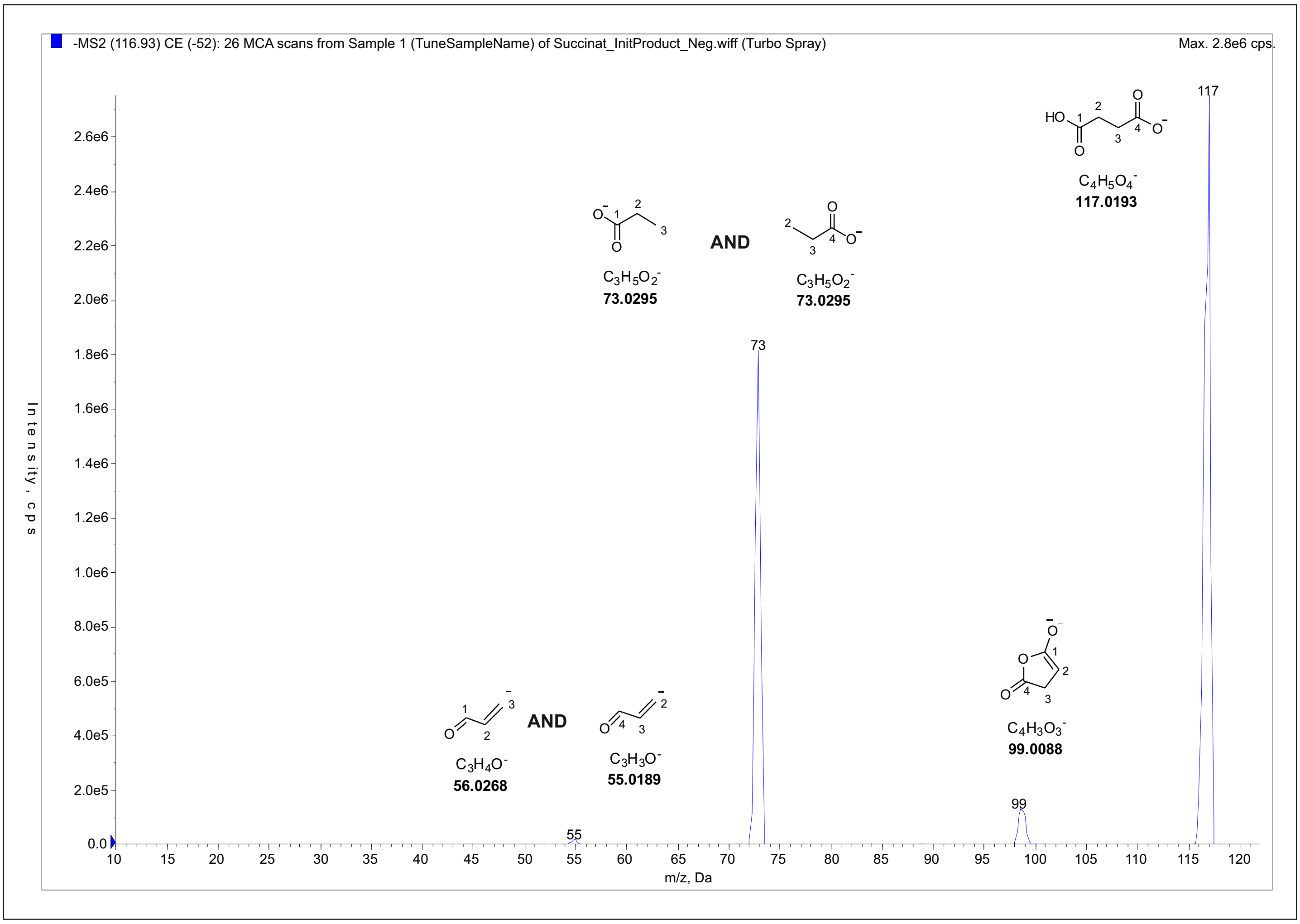

Chart S-32: The product ion spectrum of the [M-H]- ion of succinate.

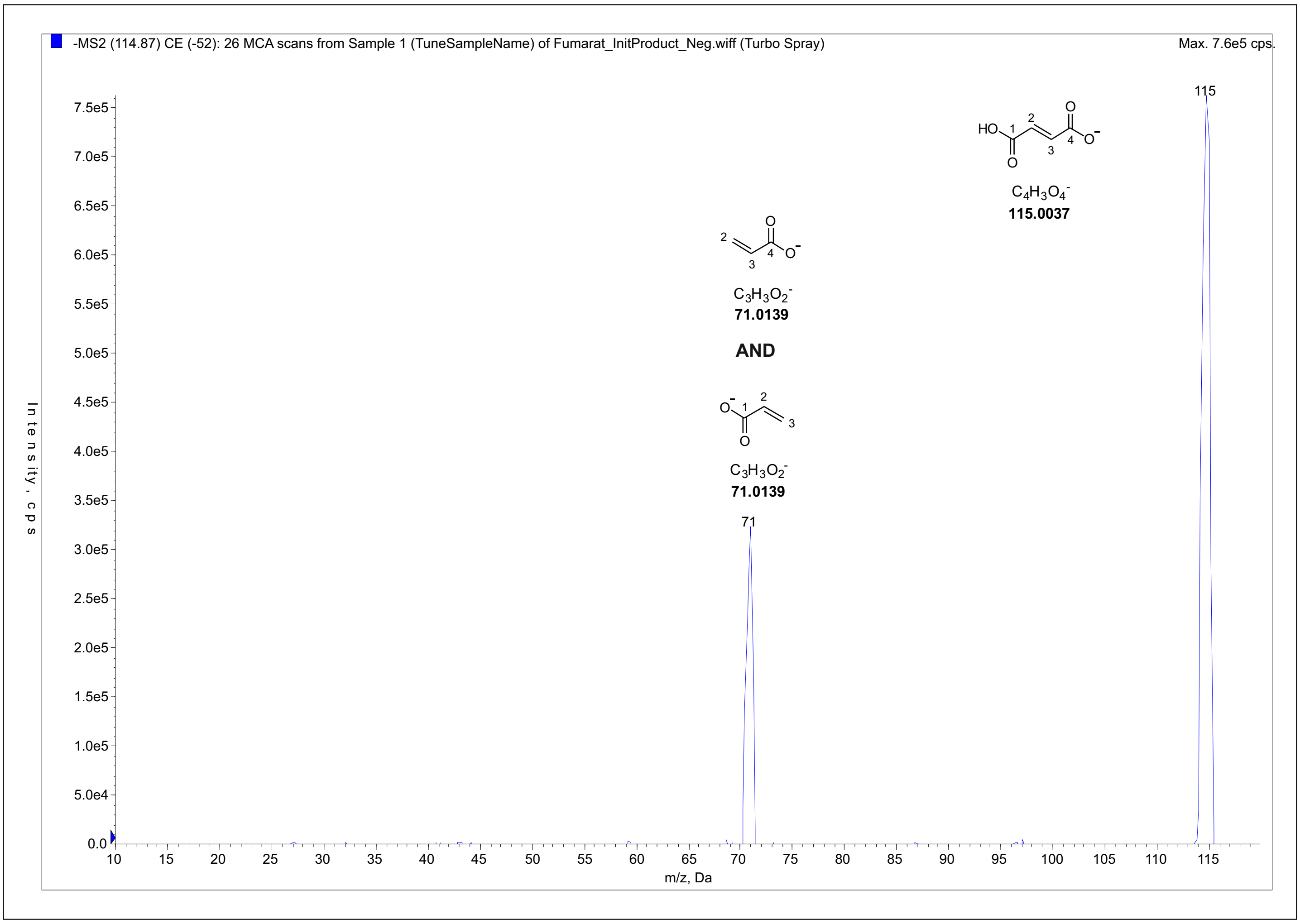

Chart S-33: The product ion spectrum of the [M-H]- ion of fumarate.

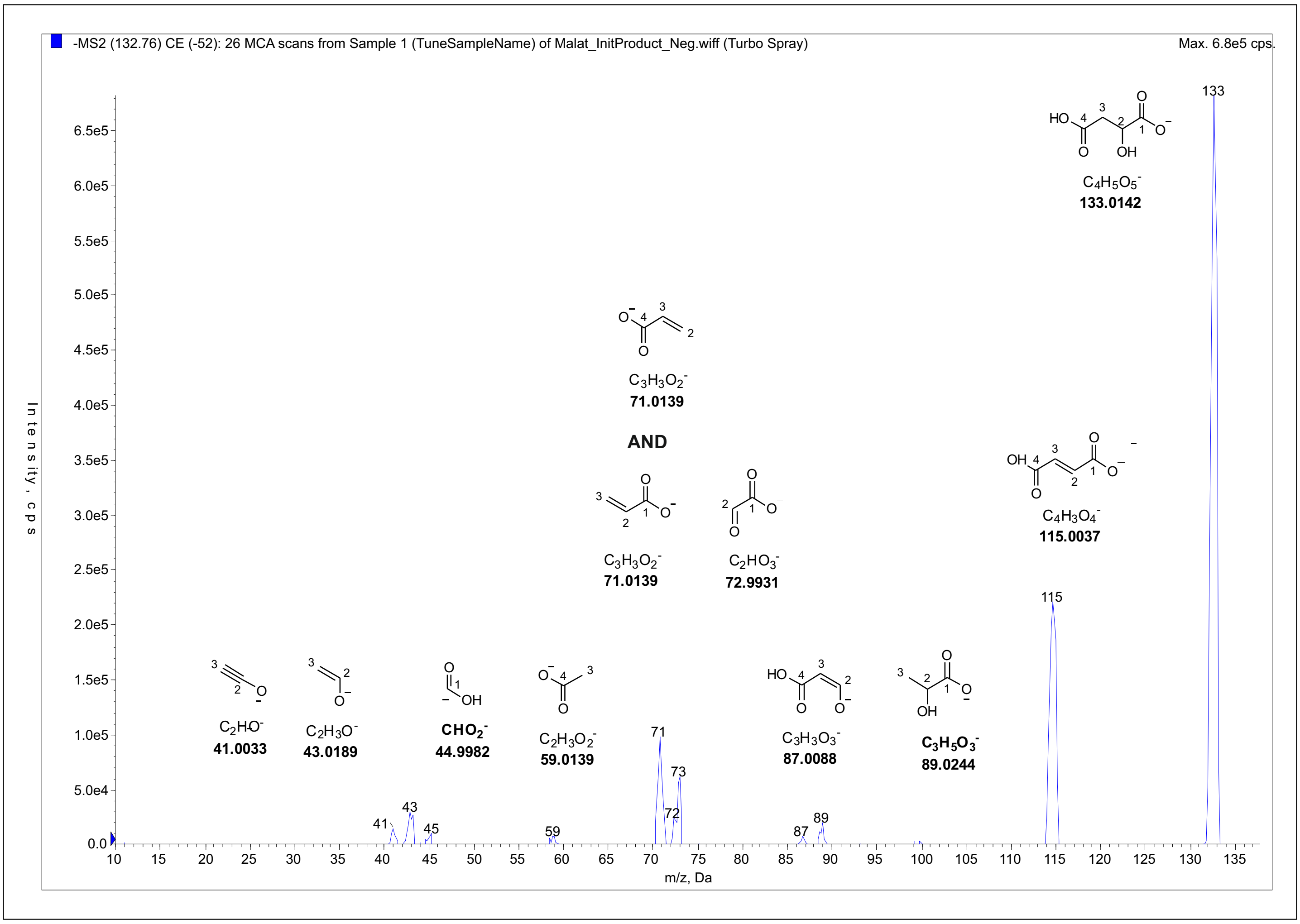

Chart S-34: The product ion spectrum of the [M-H]- ion of malate.

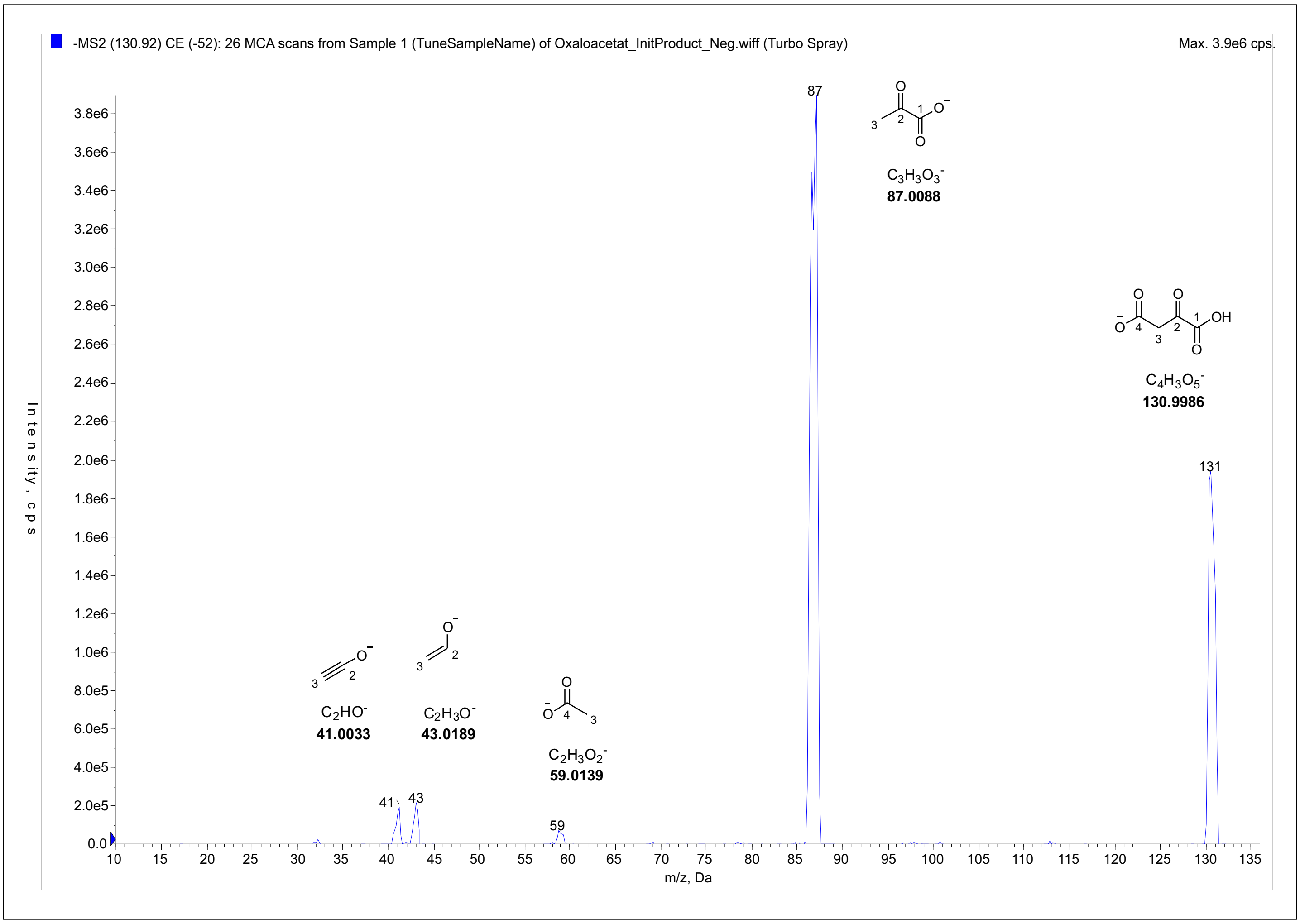

Chart S-35: The product ion spectrum of the [M-H]- ion of oxaloacetate.

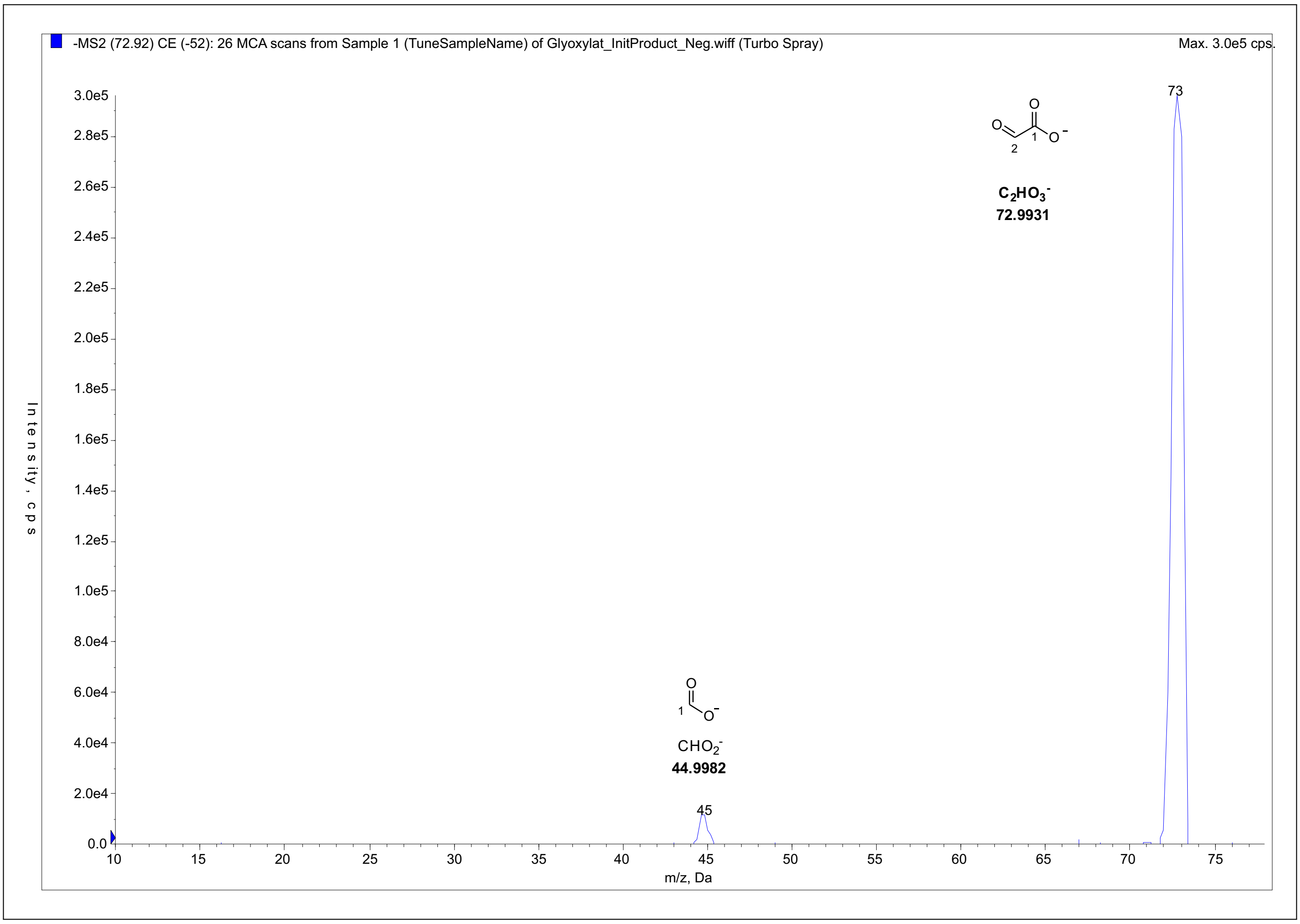

Chart S-36: The product ion spectrum of the [M-H]- ion of glyoxylate.

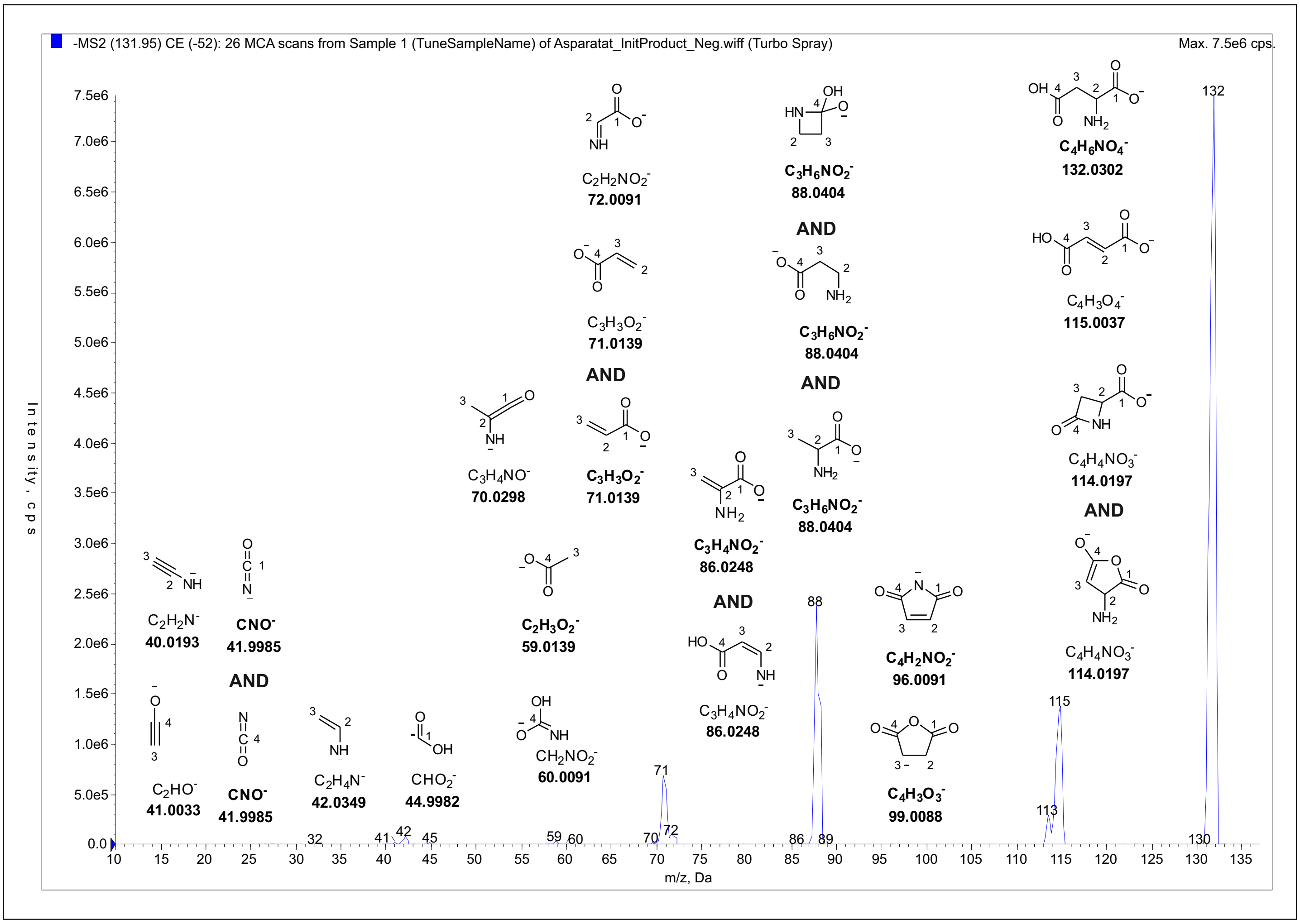

Chart S-37: The product ion spectrum of the [M-H]- ion of aspartate.

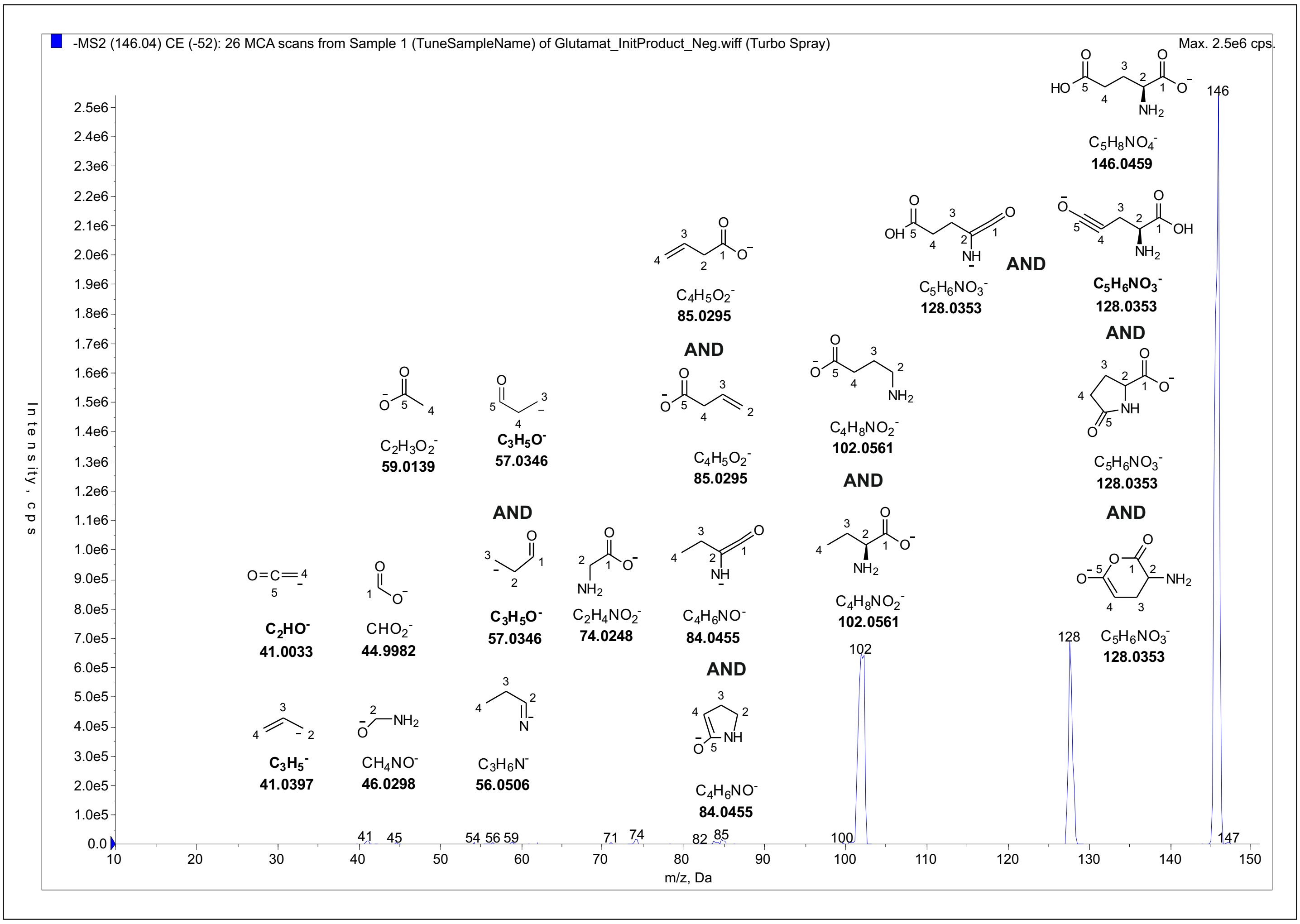

Chart S-38: The product ion spectrum of the [M-H]- ion of glutamate.

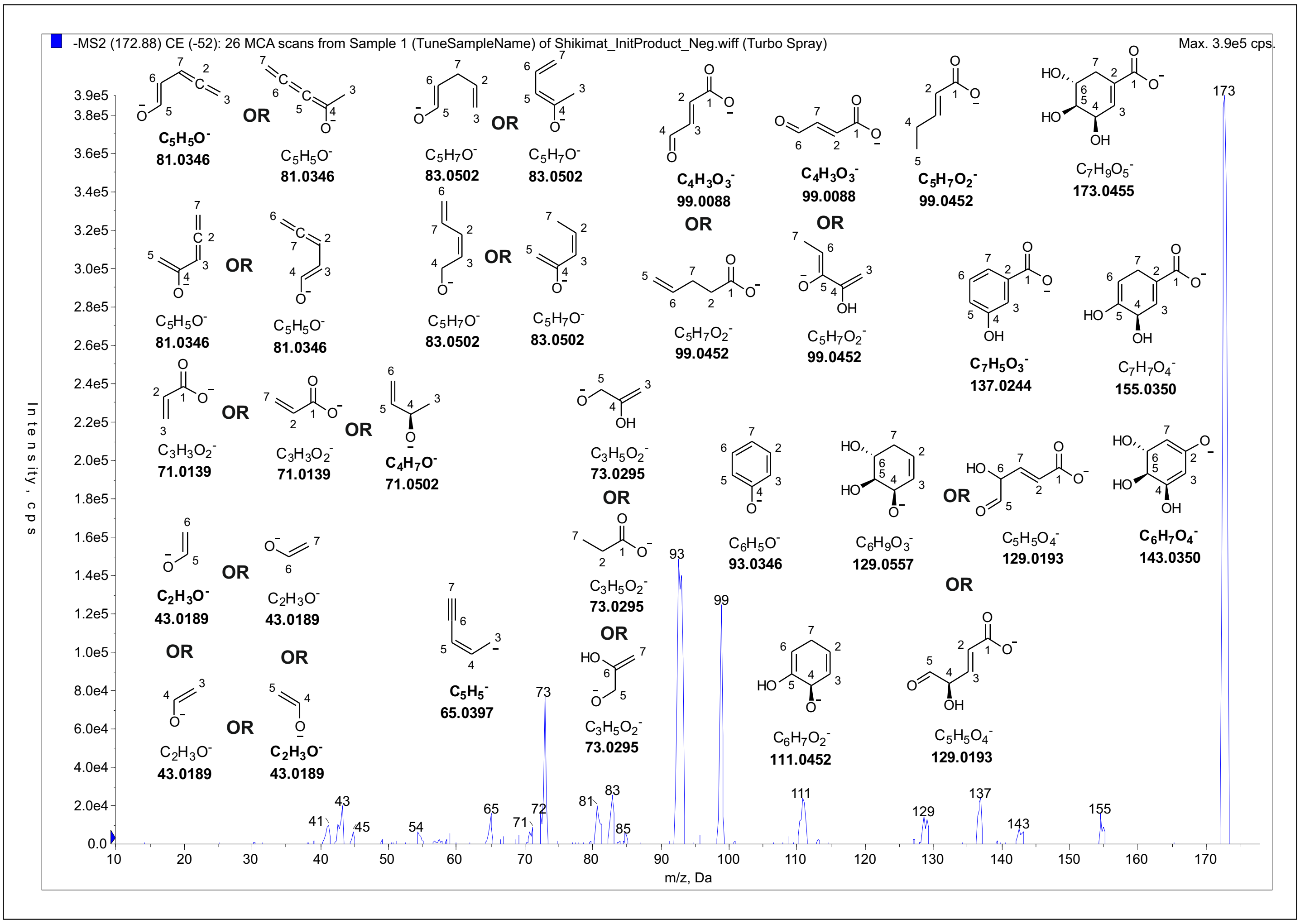

Chart S-39: The product ion spectrum of the [M-H]- ion of shikimate.

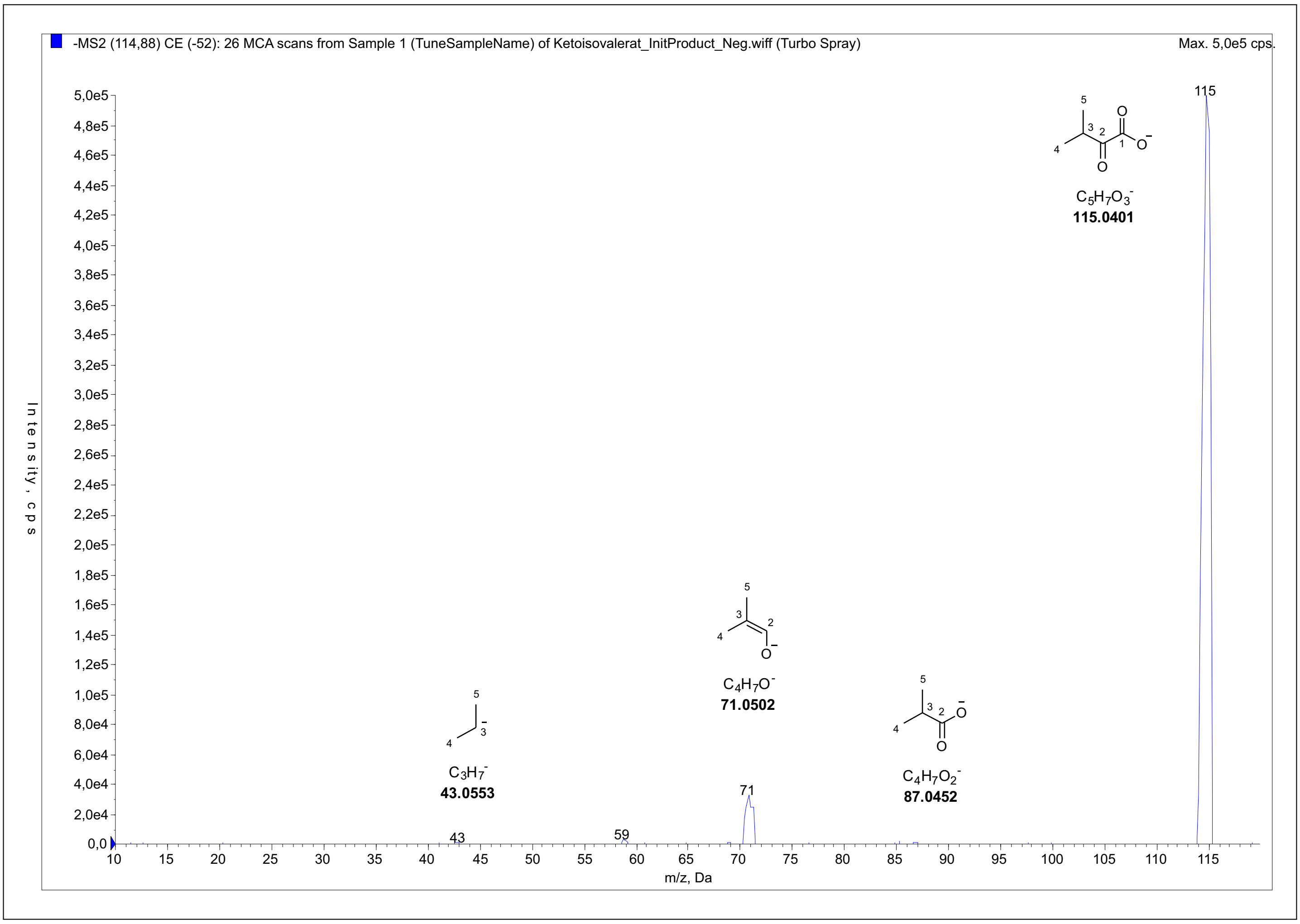

Chart S-40: The product ion spectrum of the [M-H]- ion of 2-ketoisovalerate.

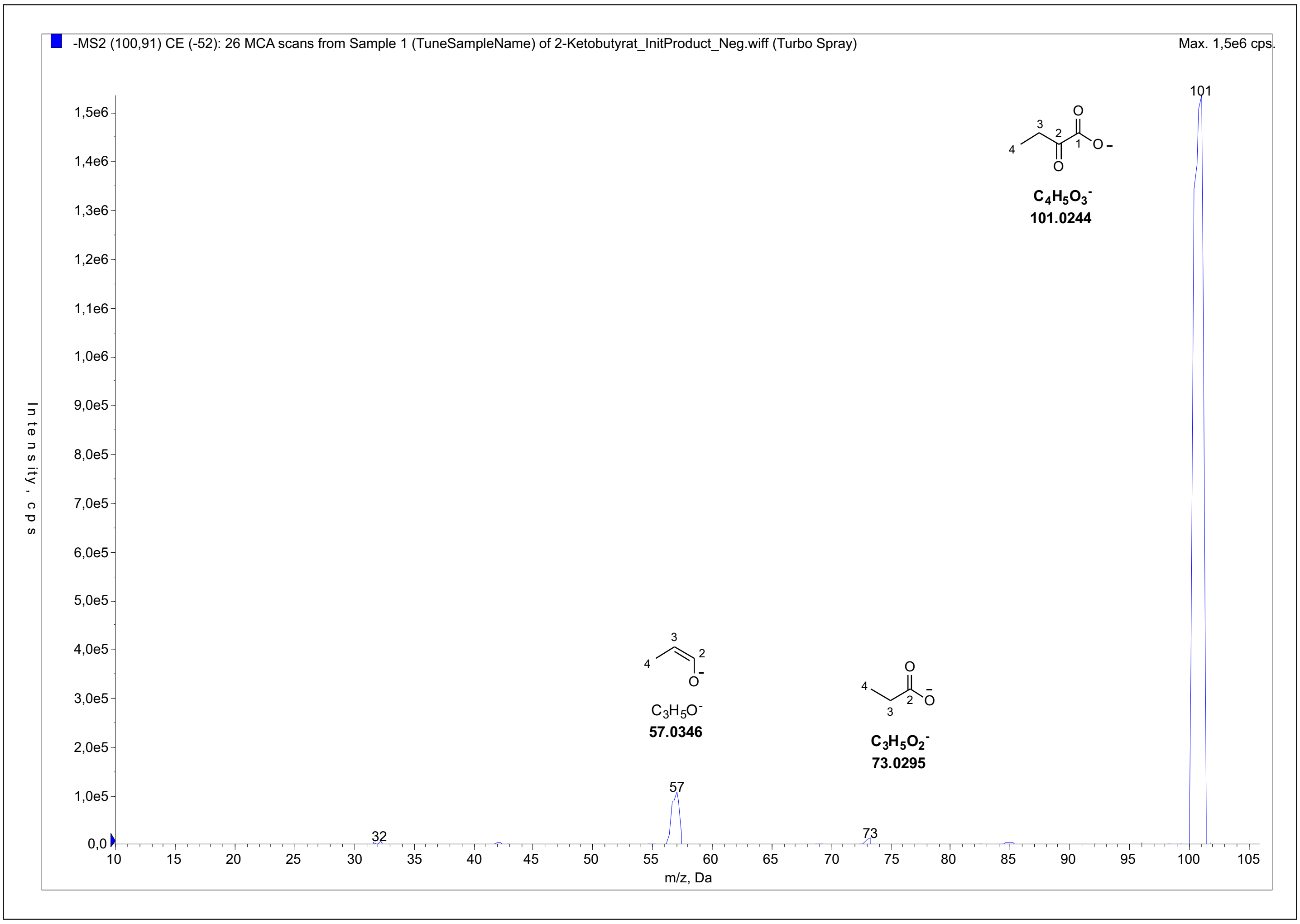

Chart S-41: The product ion spectrum of the [M-H]- ion of 2-ketobutyrate.

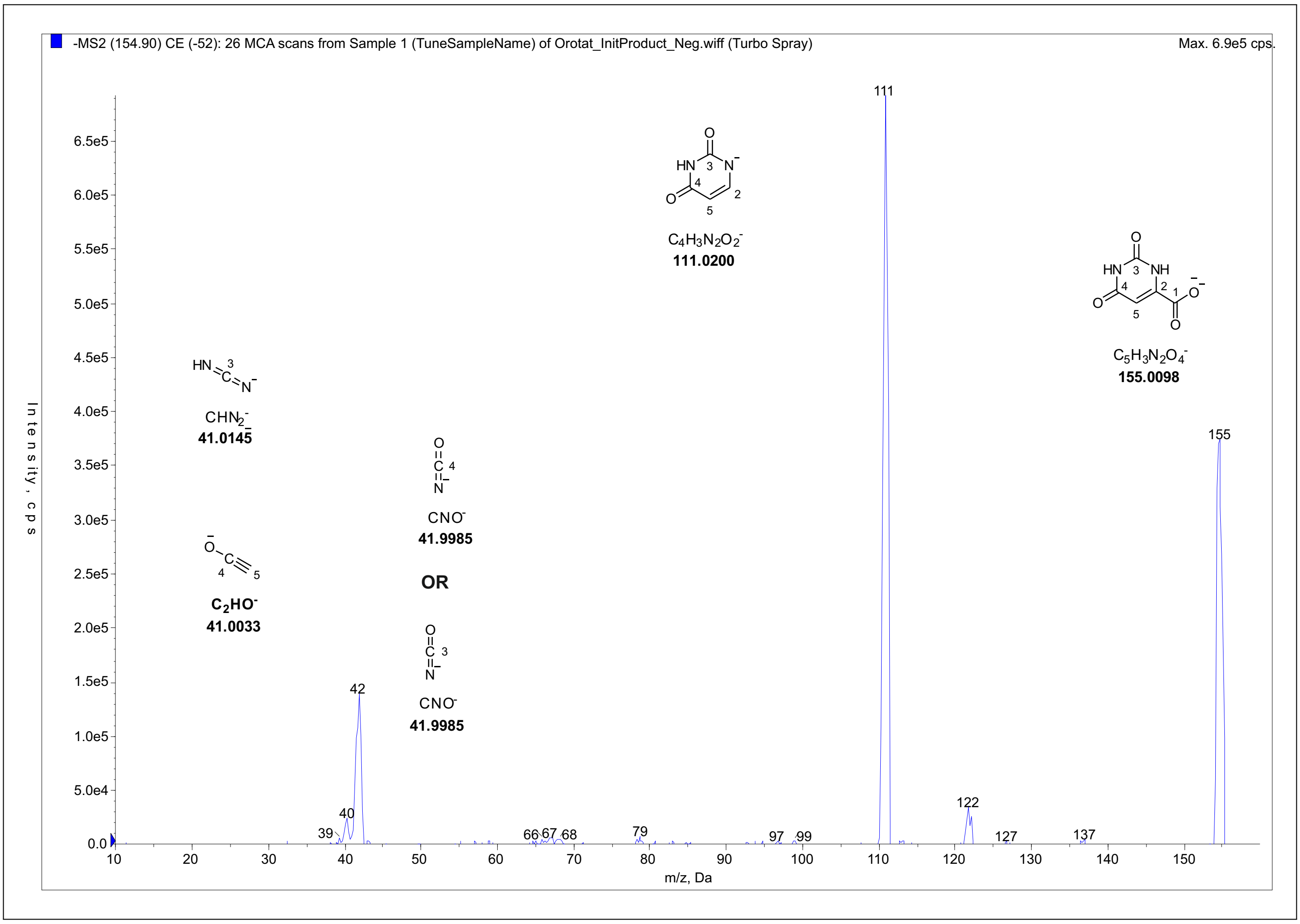

Chart S-42: The product ion spectrum of the [M-H]- ion of orotate.

Positive Ionization Mode

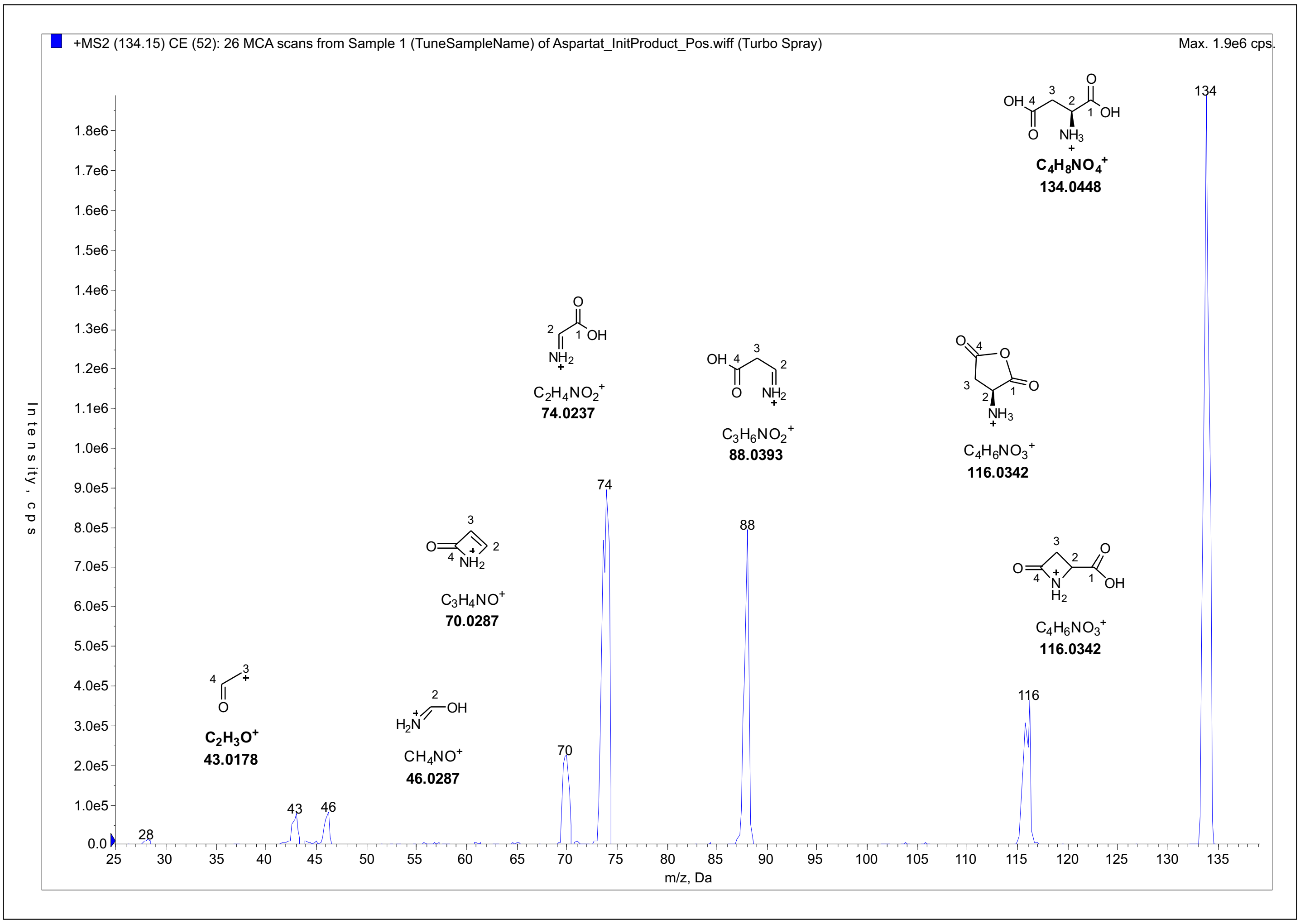

Chart S-43: The product ion spectrum of the [M+H]+ ion of aspartate.

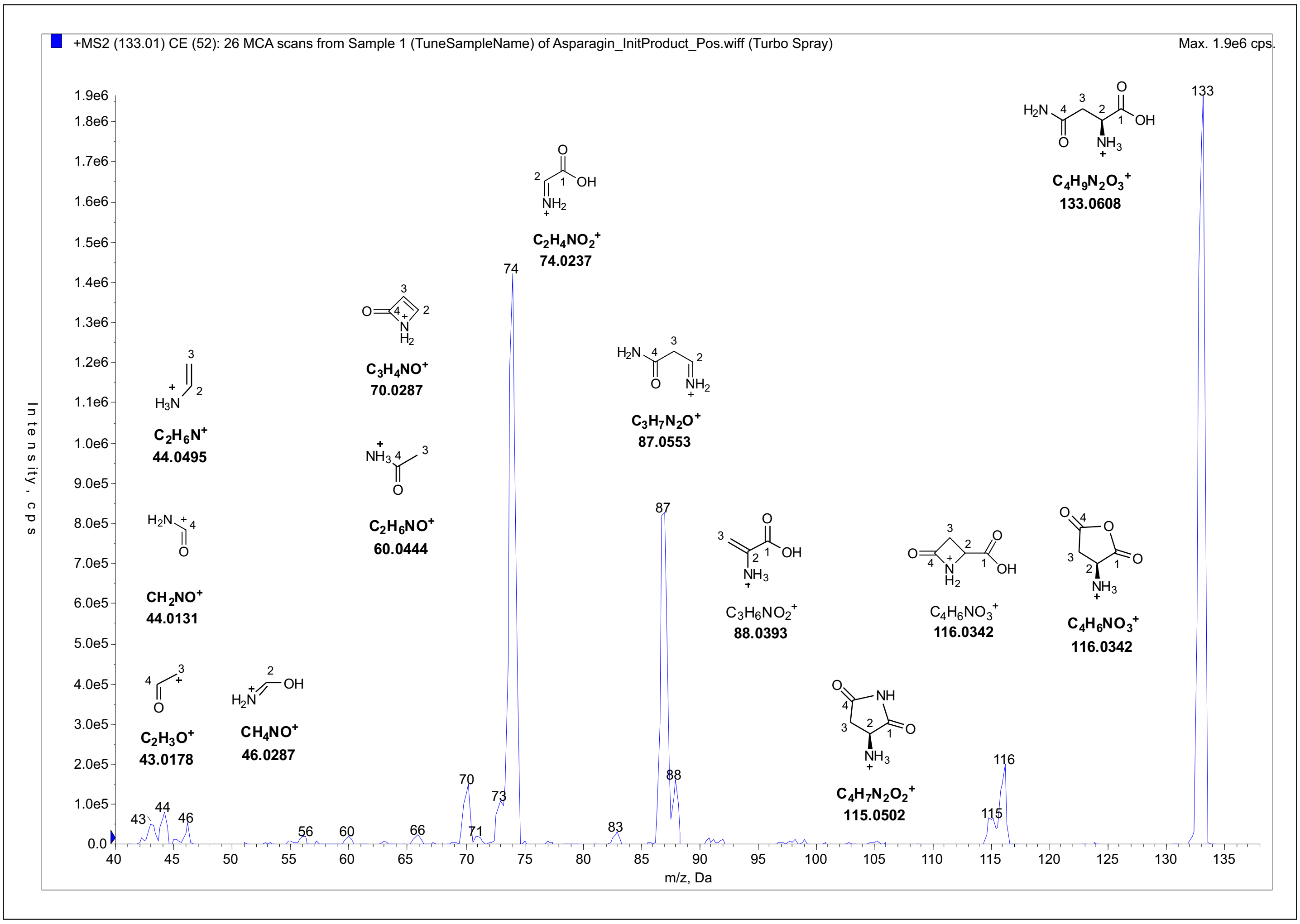

Chart S-44: The product ion spectrum of the [M+H]+ ion of asparagine.

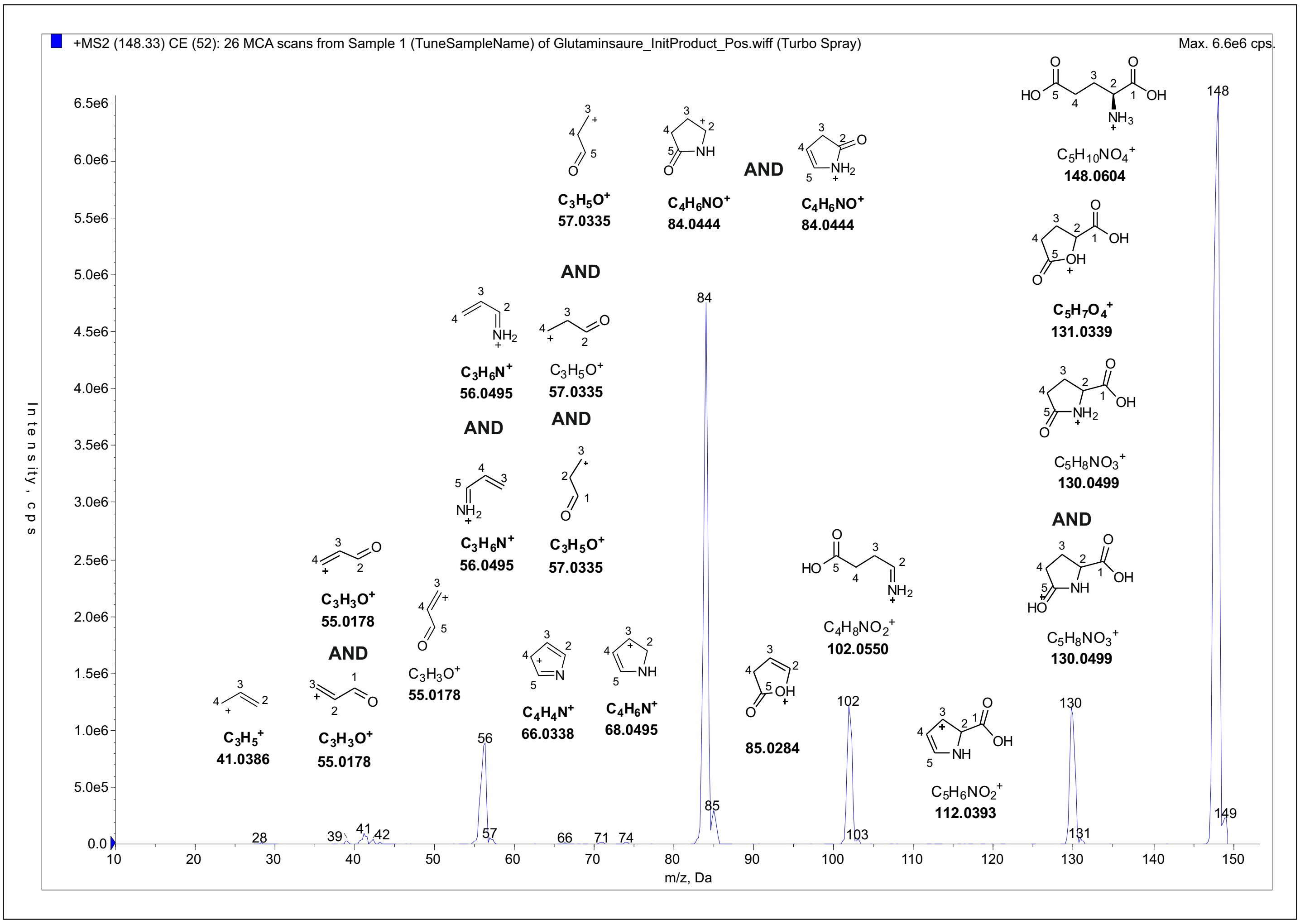

Chart S-45: The product ion spectrum of the [M+H]+ ion of glutamate.

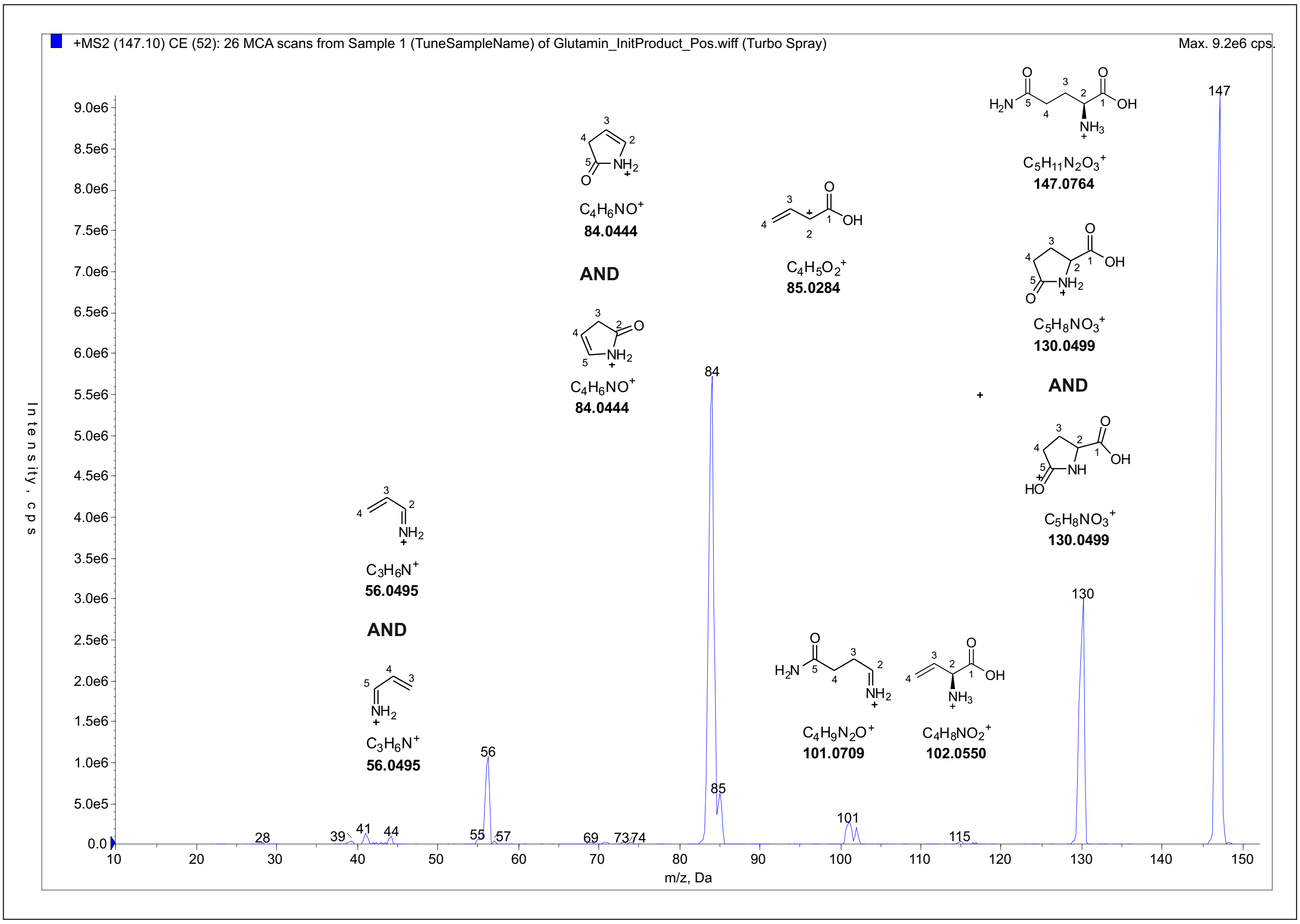

Chart S-46: The product ion spectrum of the [M+H]+ ion of glutamine.

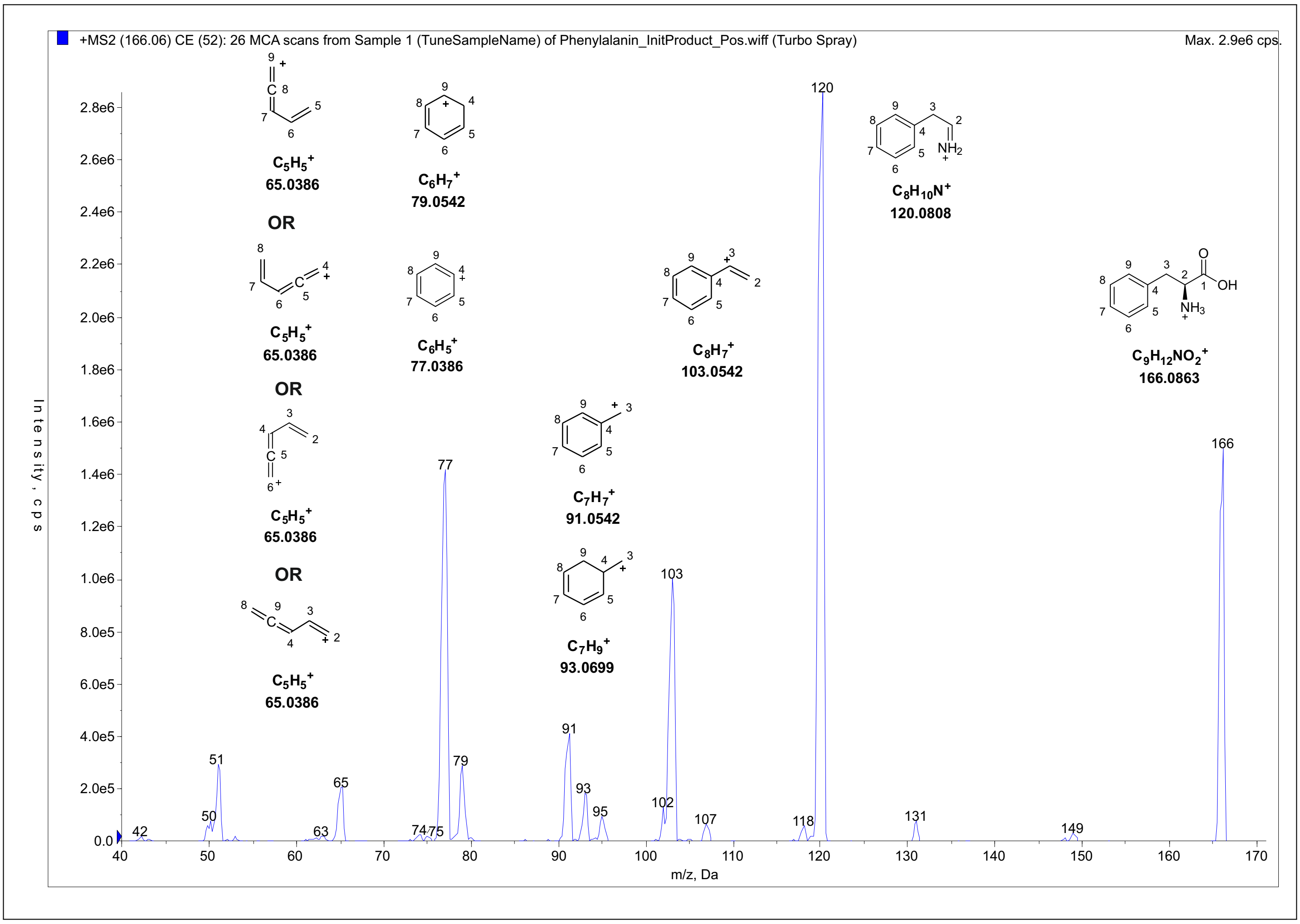

Chart S-47: The product ion spectrum of the [M+H]+ ion of phenylalanine.

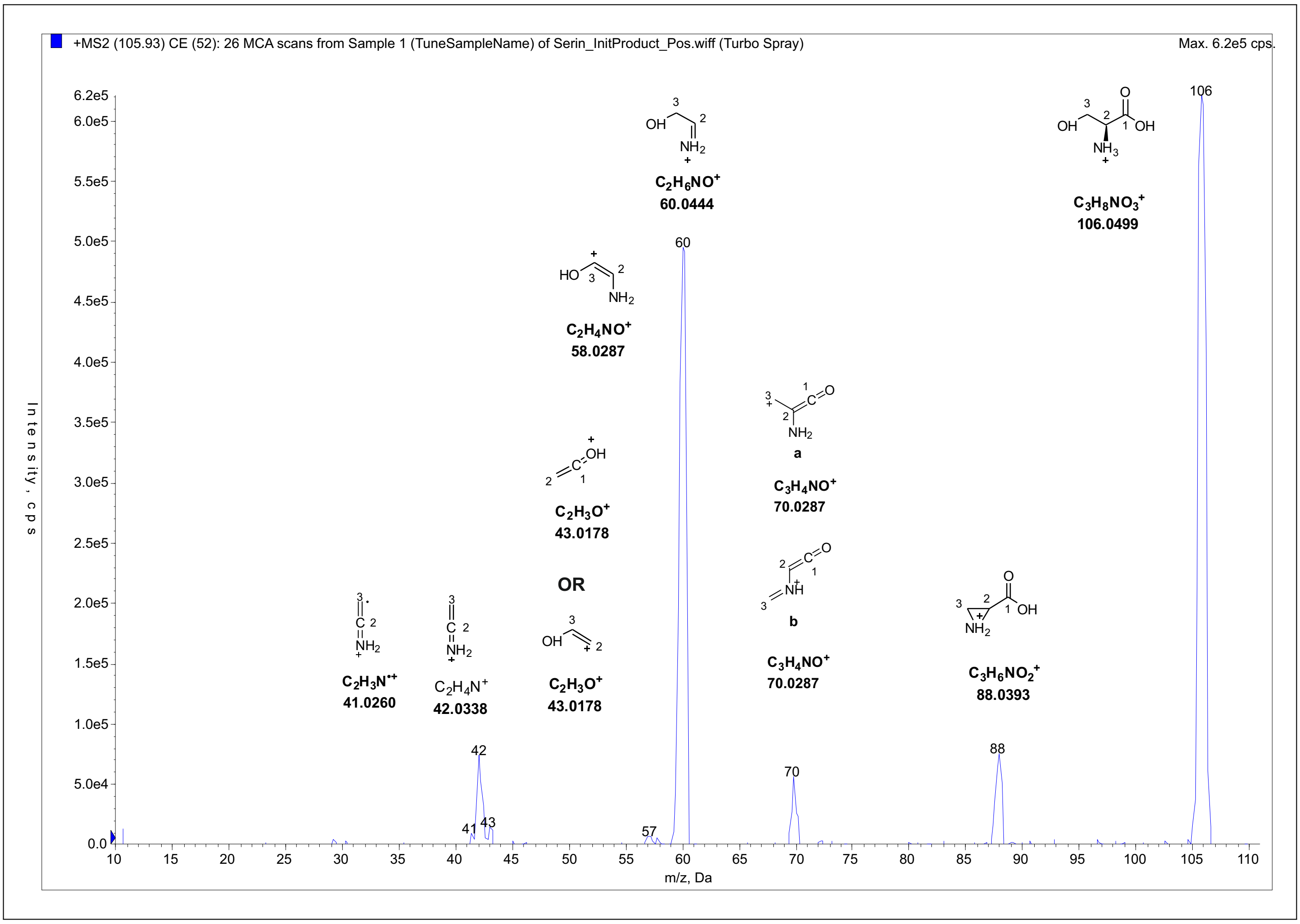

Chart S-48: The product ion spectrum of the [M+H]+ ion of serine.

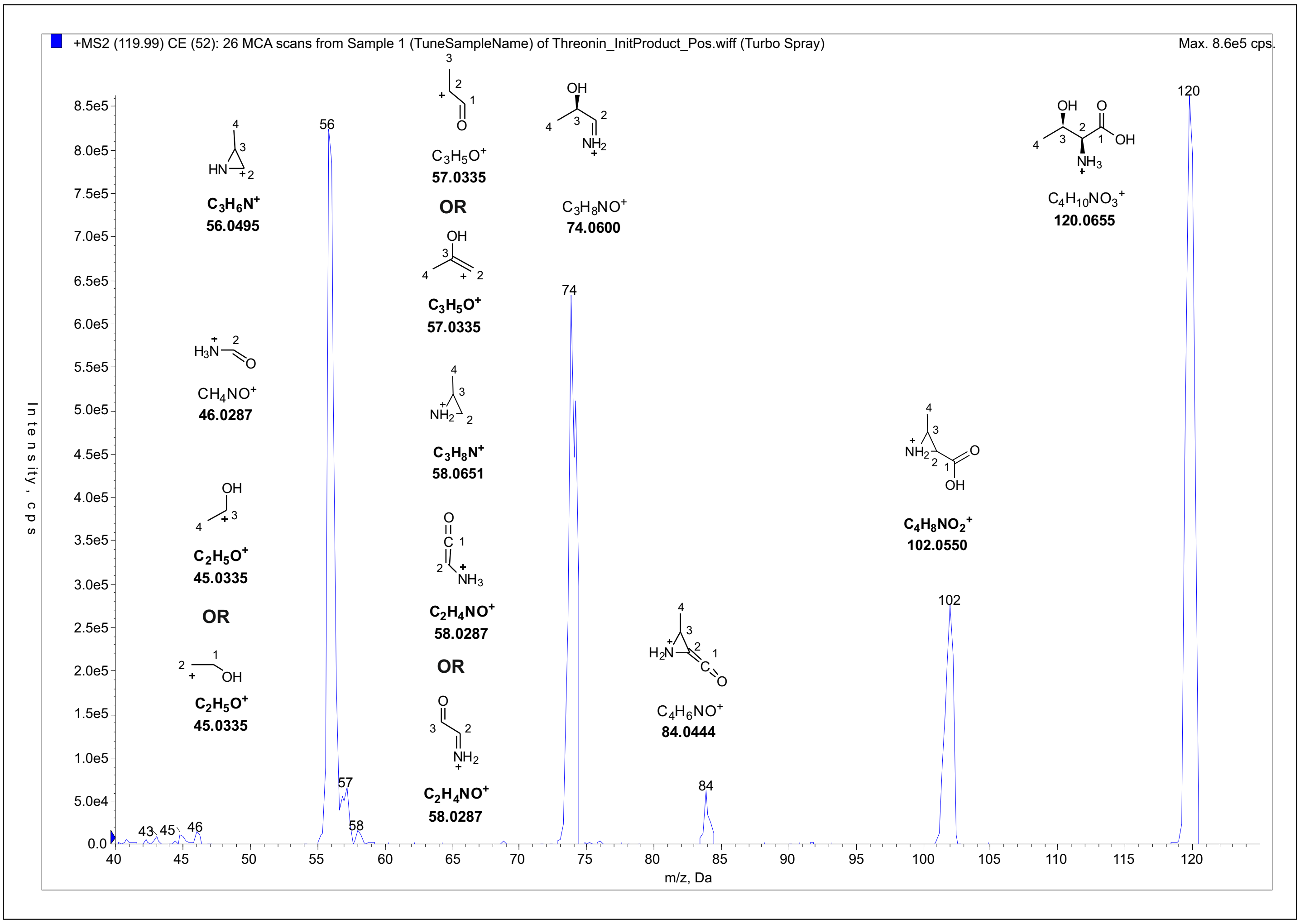

Chart S-49: The product ion spectrum of the [M+H]+ ion of threonine.

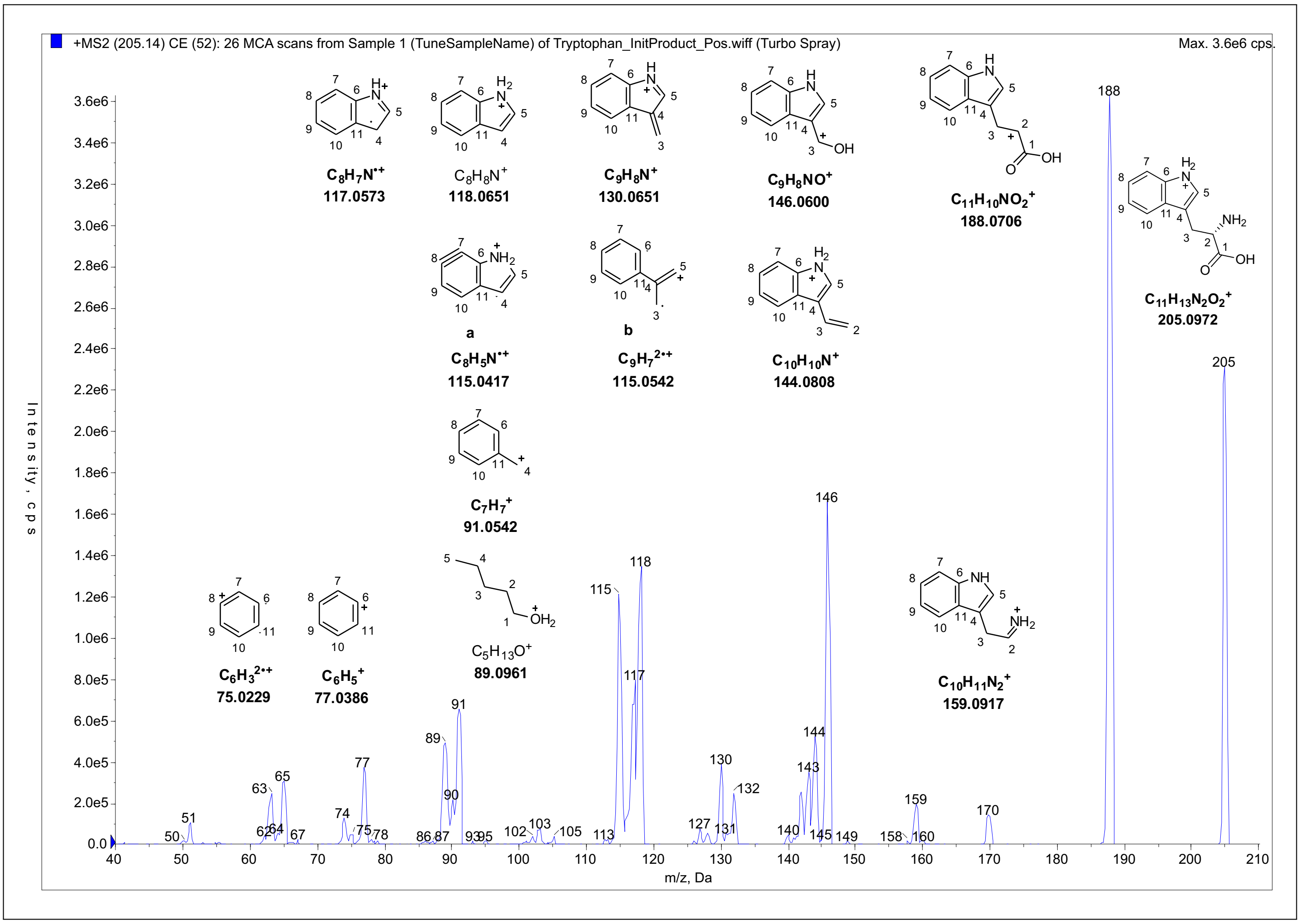

Chart S-50: The product ion spectrum of the [M+H]+ ion of tryptophane.

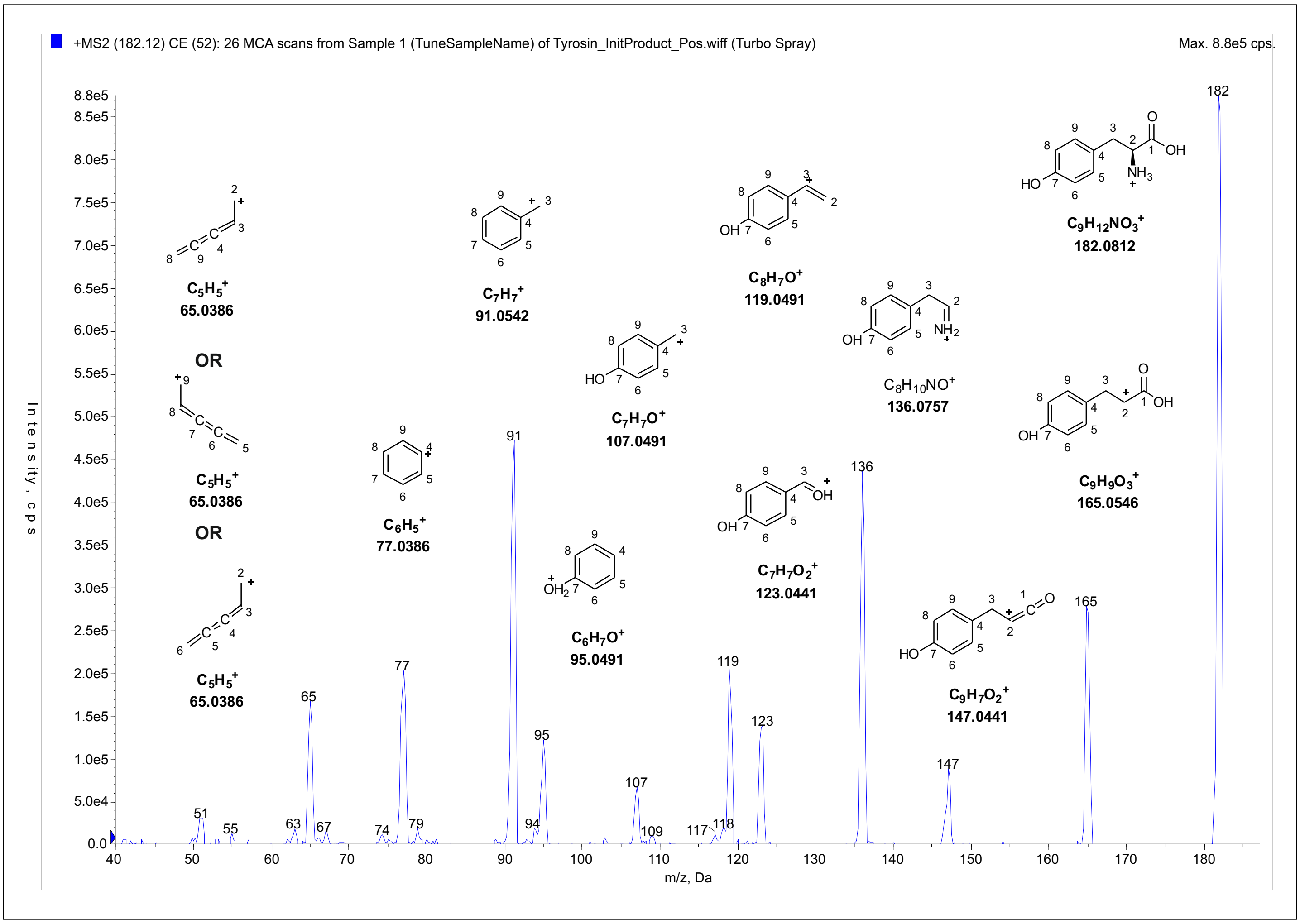

Chart S-51: The product ion spectrum of the [M+H]+ ion of tyrosine.

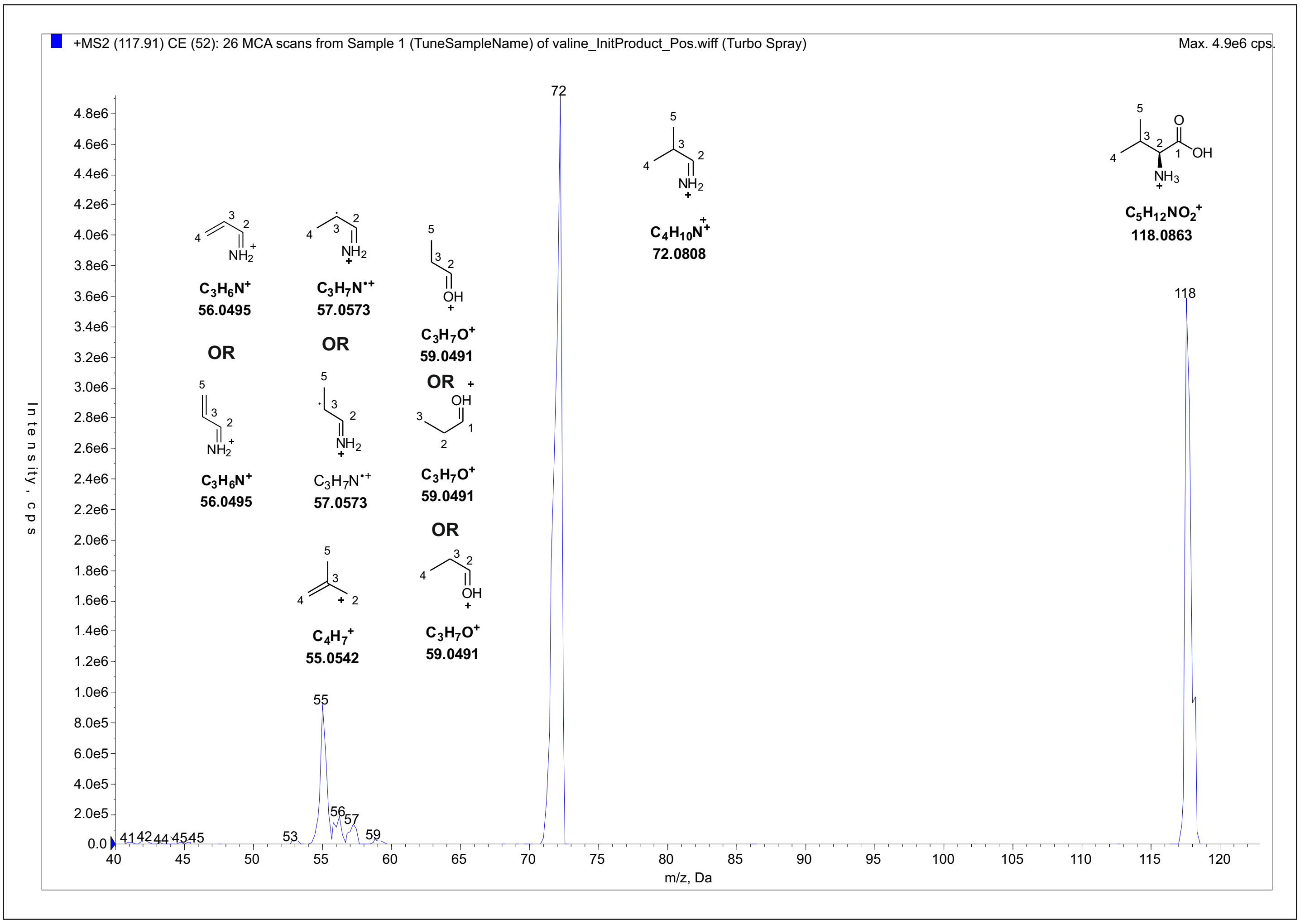

Chart S-52: The product ion spectrum of the [M+H]+ ion of valine.

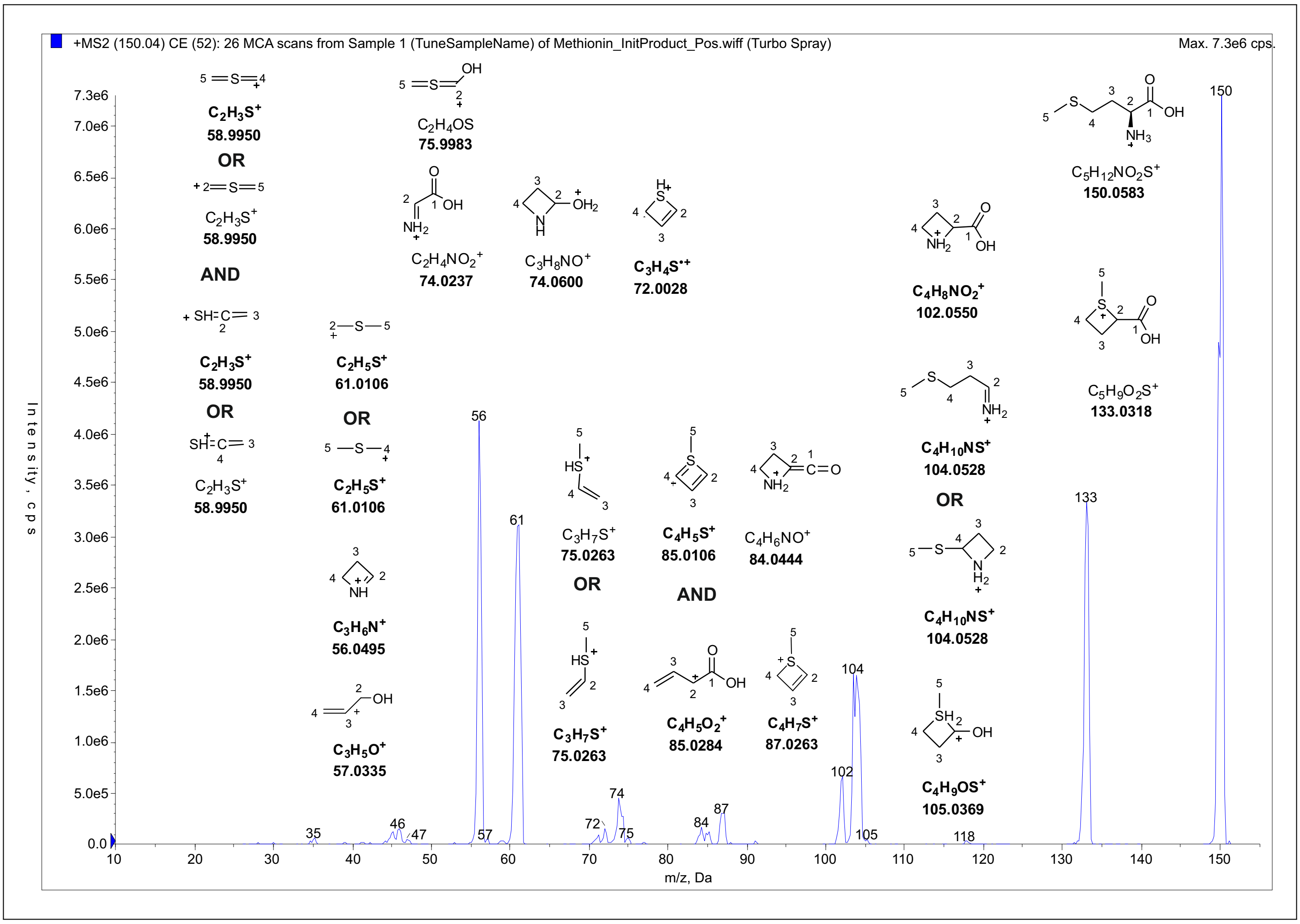

Chart S-53: The product ion spectrum of the [M+H]+ ion of methionine.

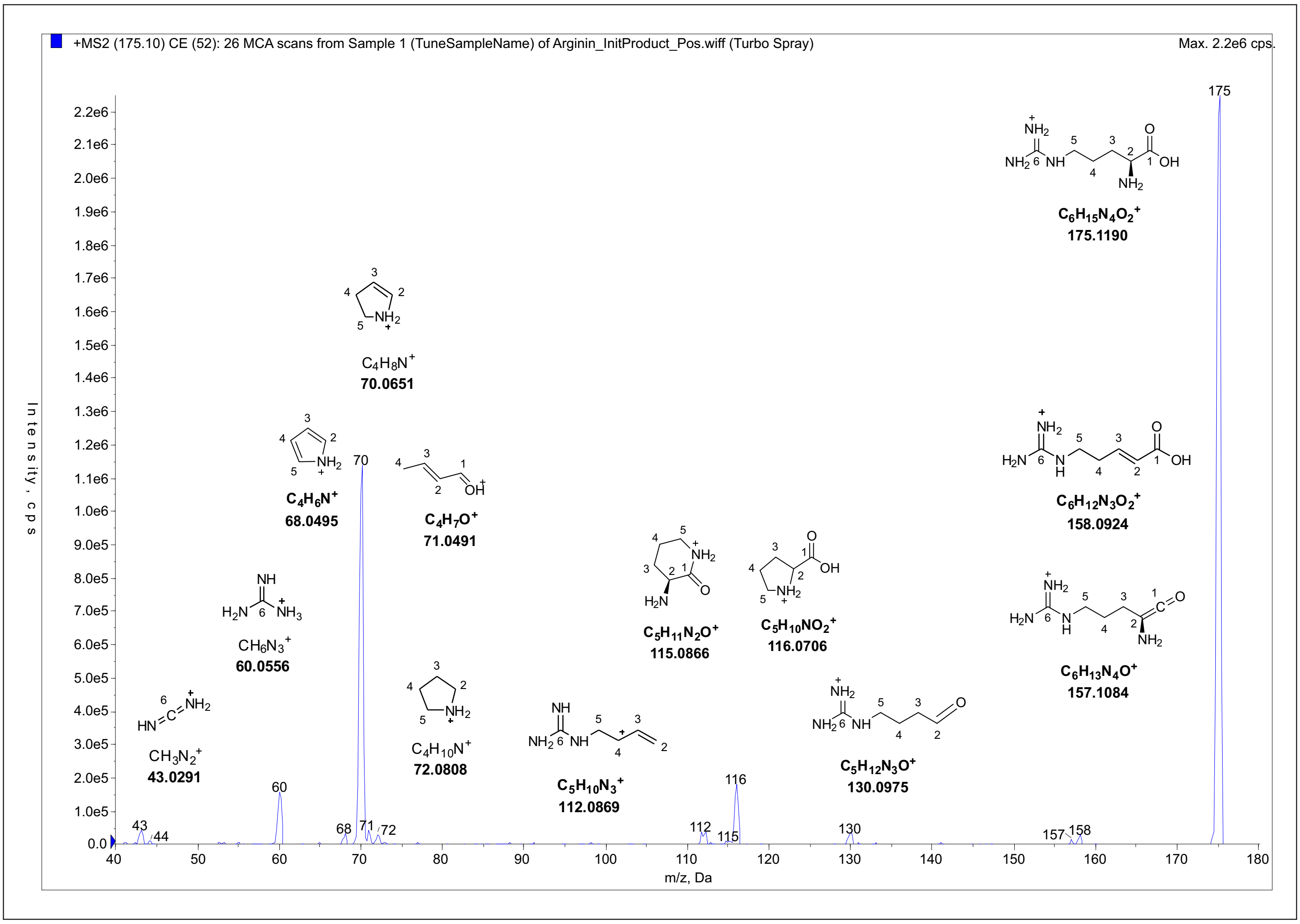

Chart S-54: The product ion spectrum of the [M+H]+ ion of arginine.

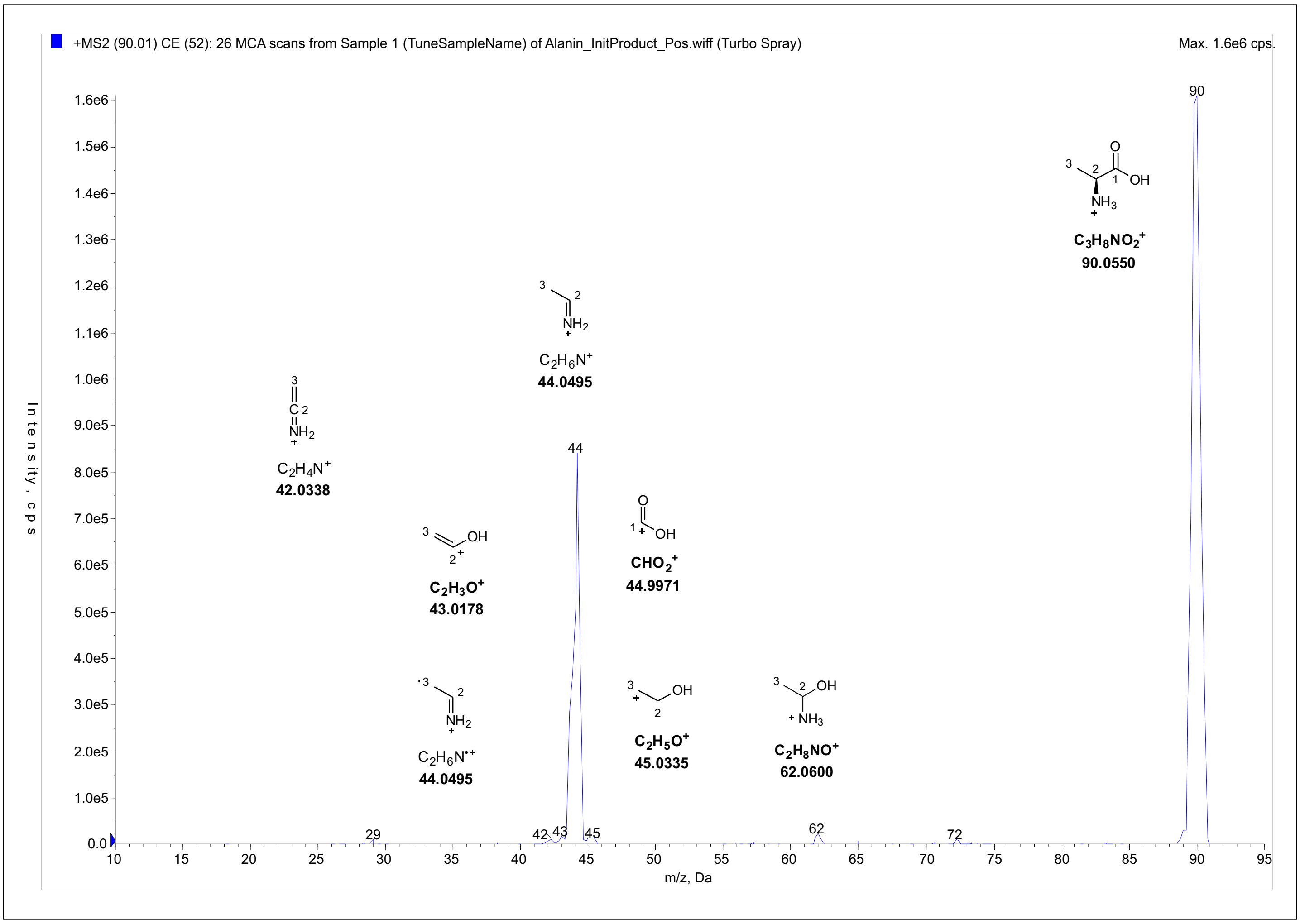

Chart S-55: The product ion spectrum of the [M+H]+ ion of alanine.

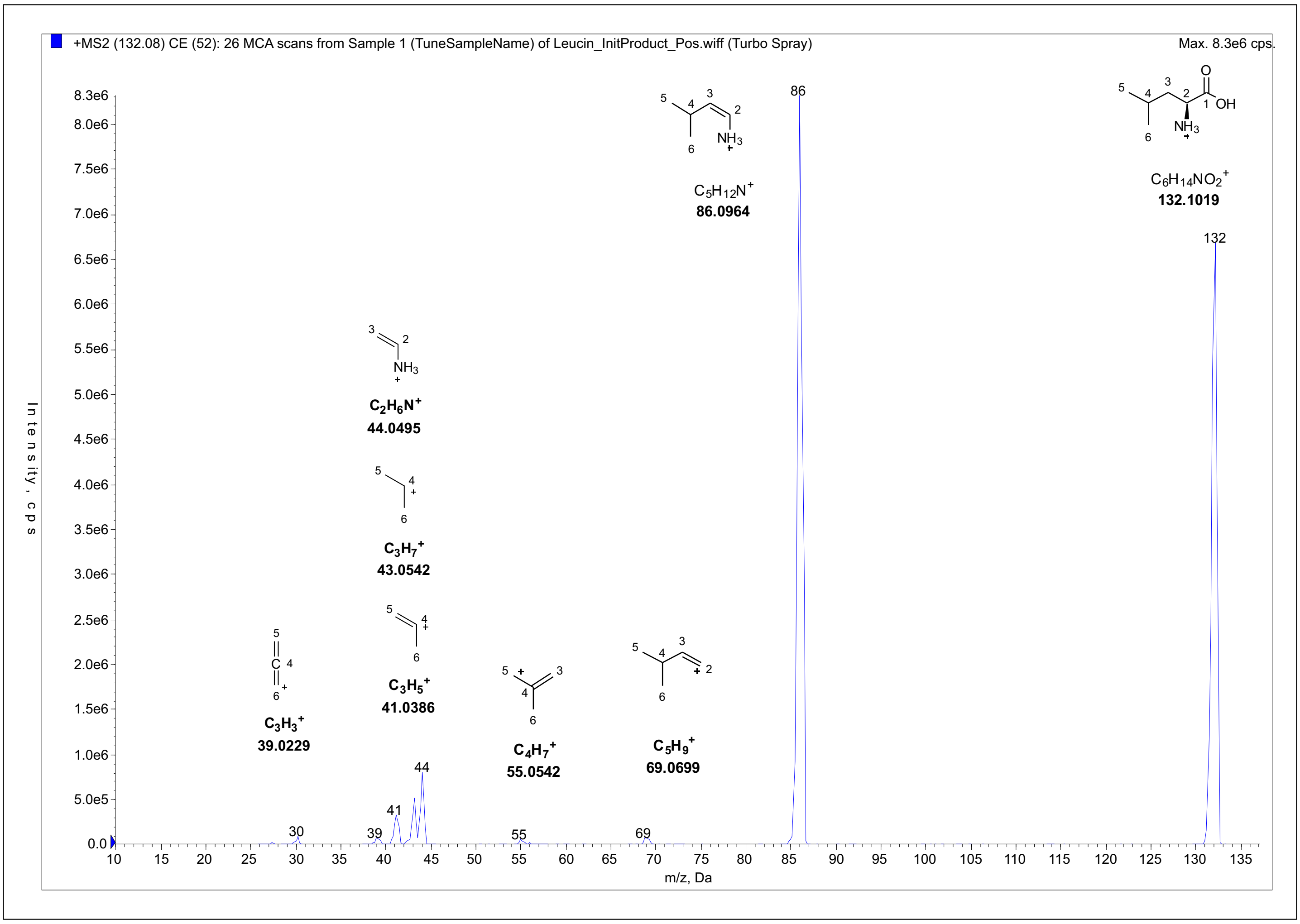

Chart S-56: The product ion spectrum of the [M+H]+ ion of leucine.

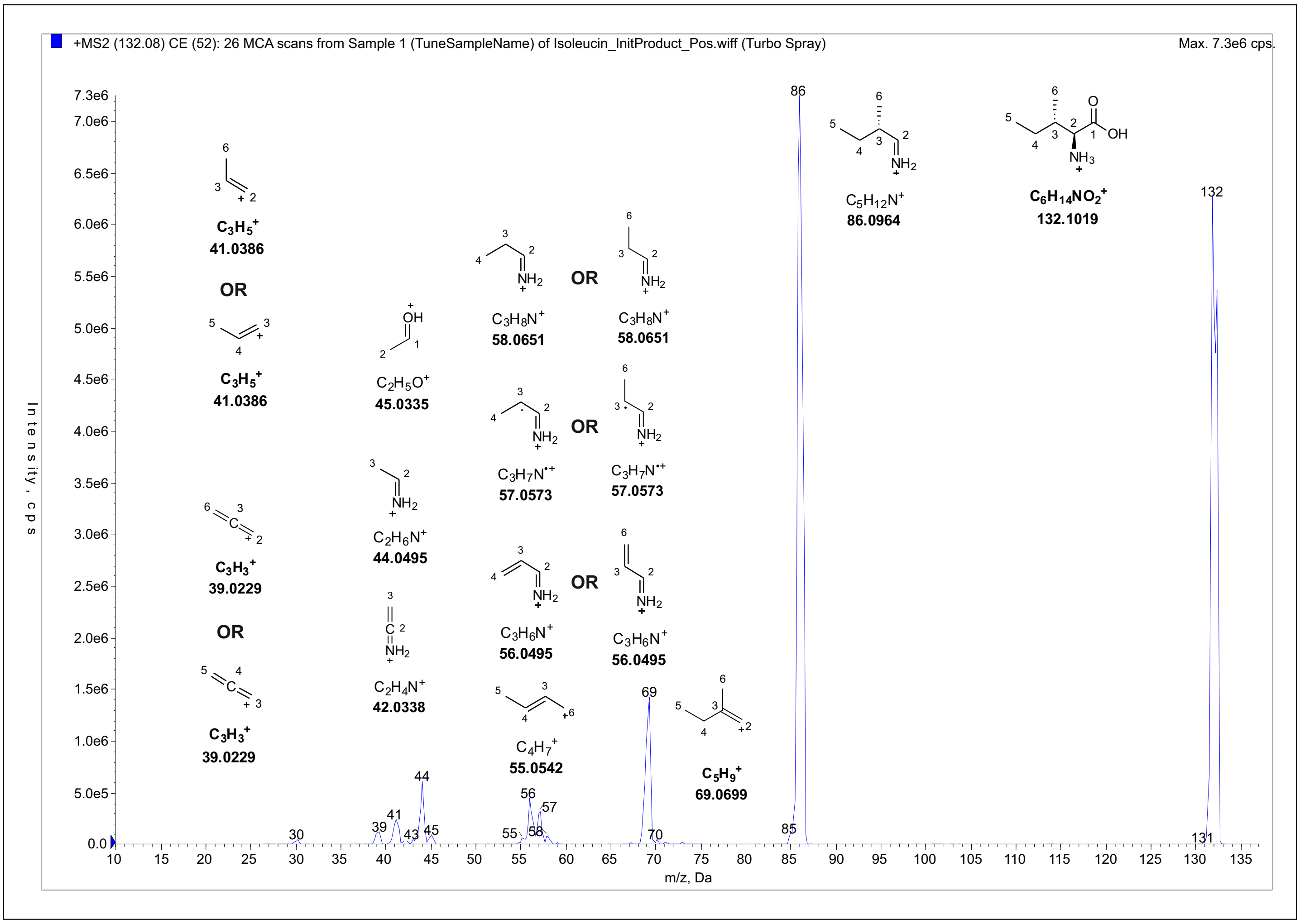

Chart S-57: The product ion spectrum of the [M+H]+ ion of isoleucine.

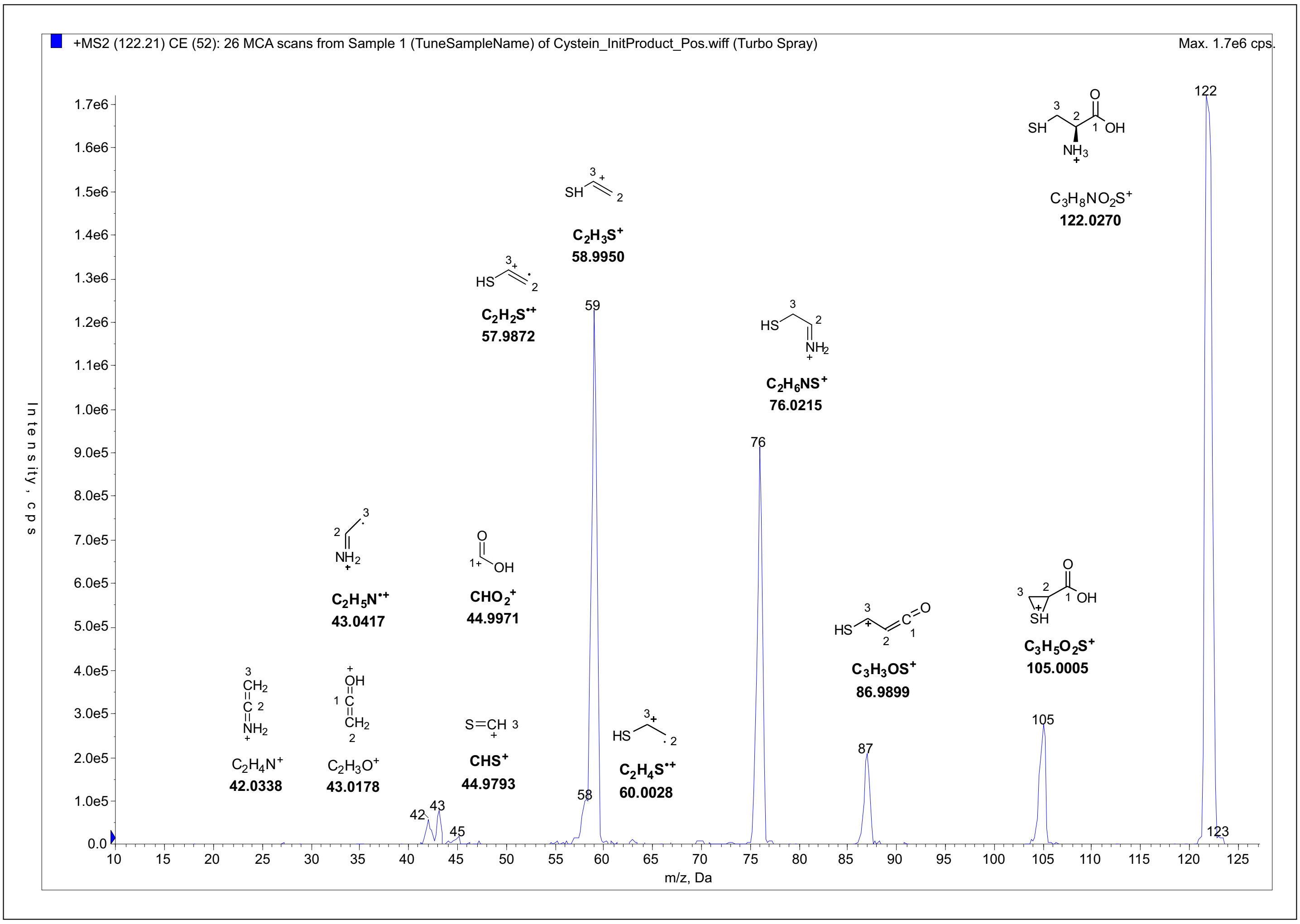

Chart S-58: The product ion spectrum of the [M+H]+ ion of cysteine.

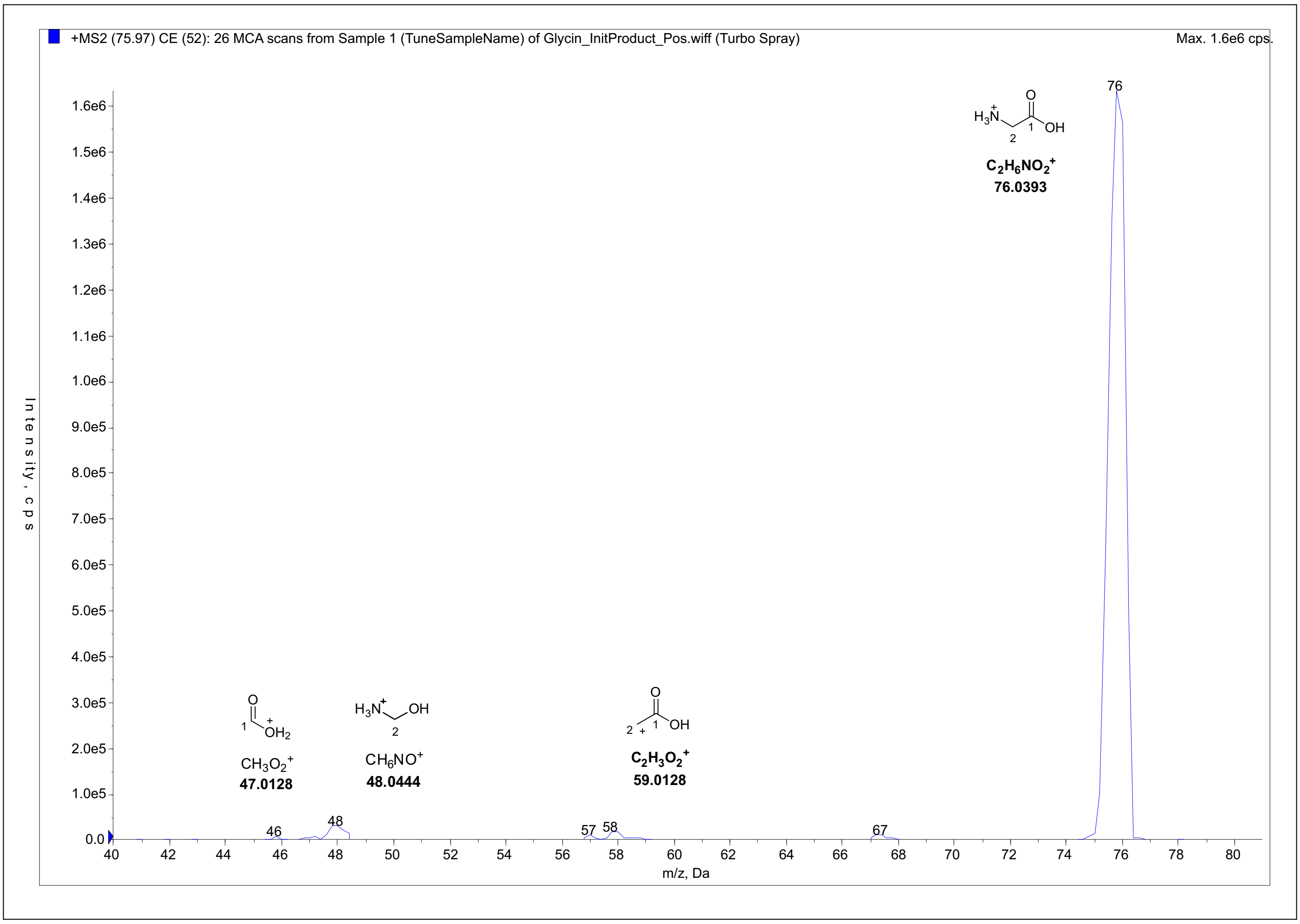

Chart S-59: The product ion spectrum of the [M+H]+ ion of glycine.

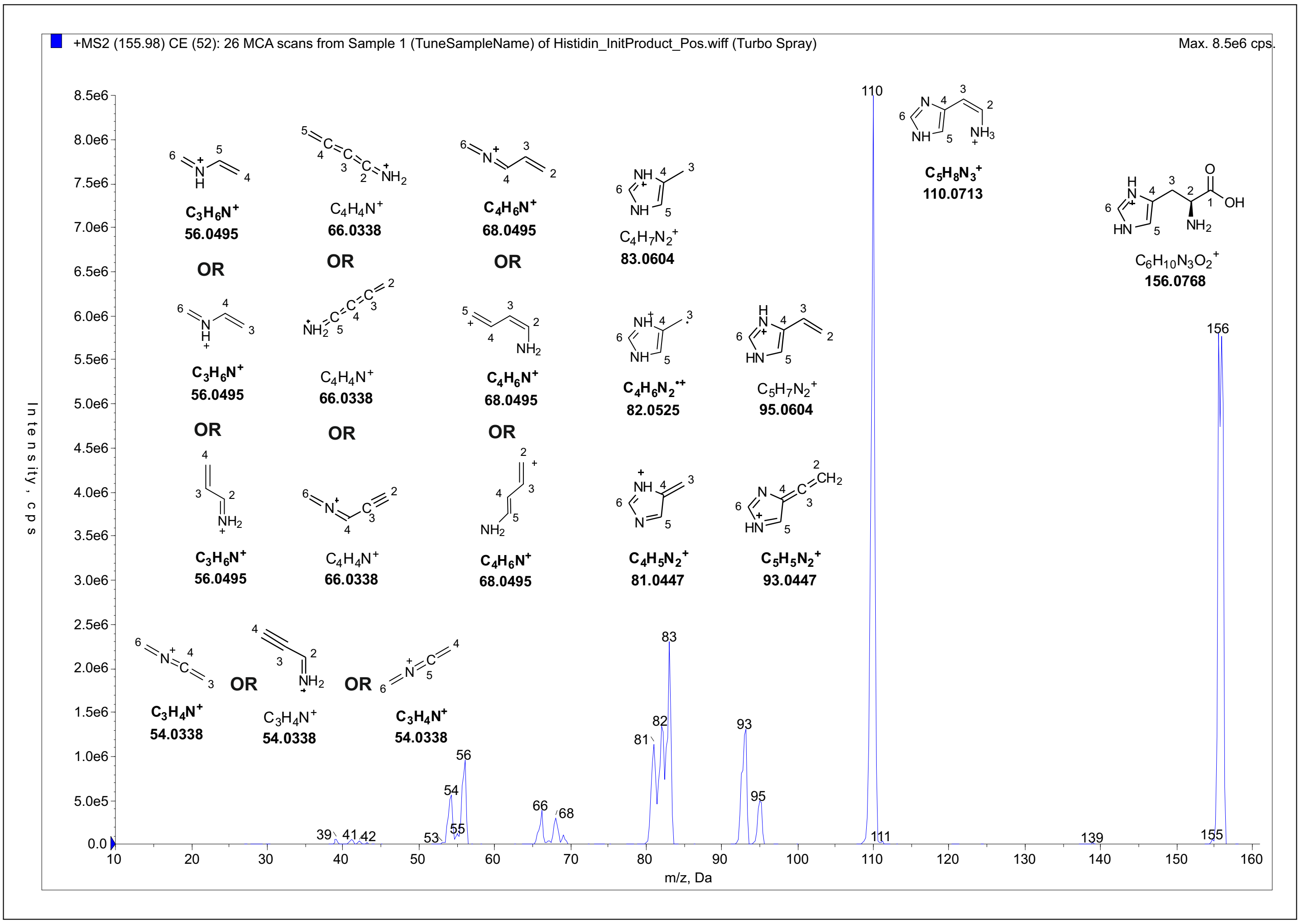

Chart S-60: The product ion spectrum of the [M+H]+ ion of histidine.

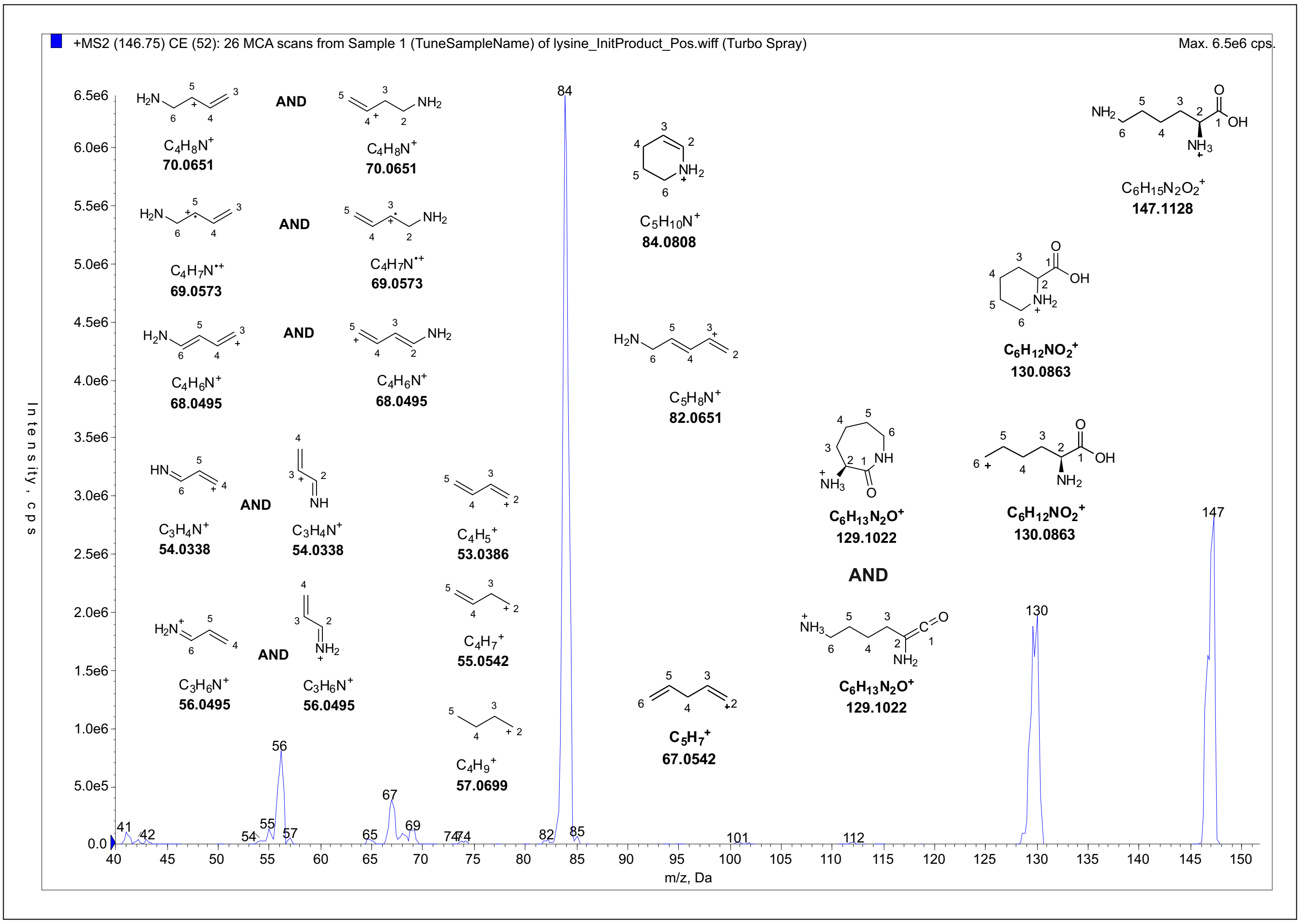

Chart S-61: The product ion spectrum of the [M+H]+ ion of lysine.

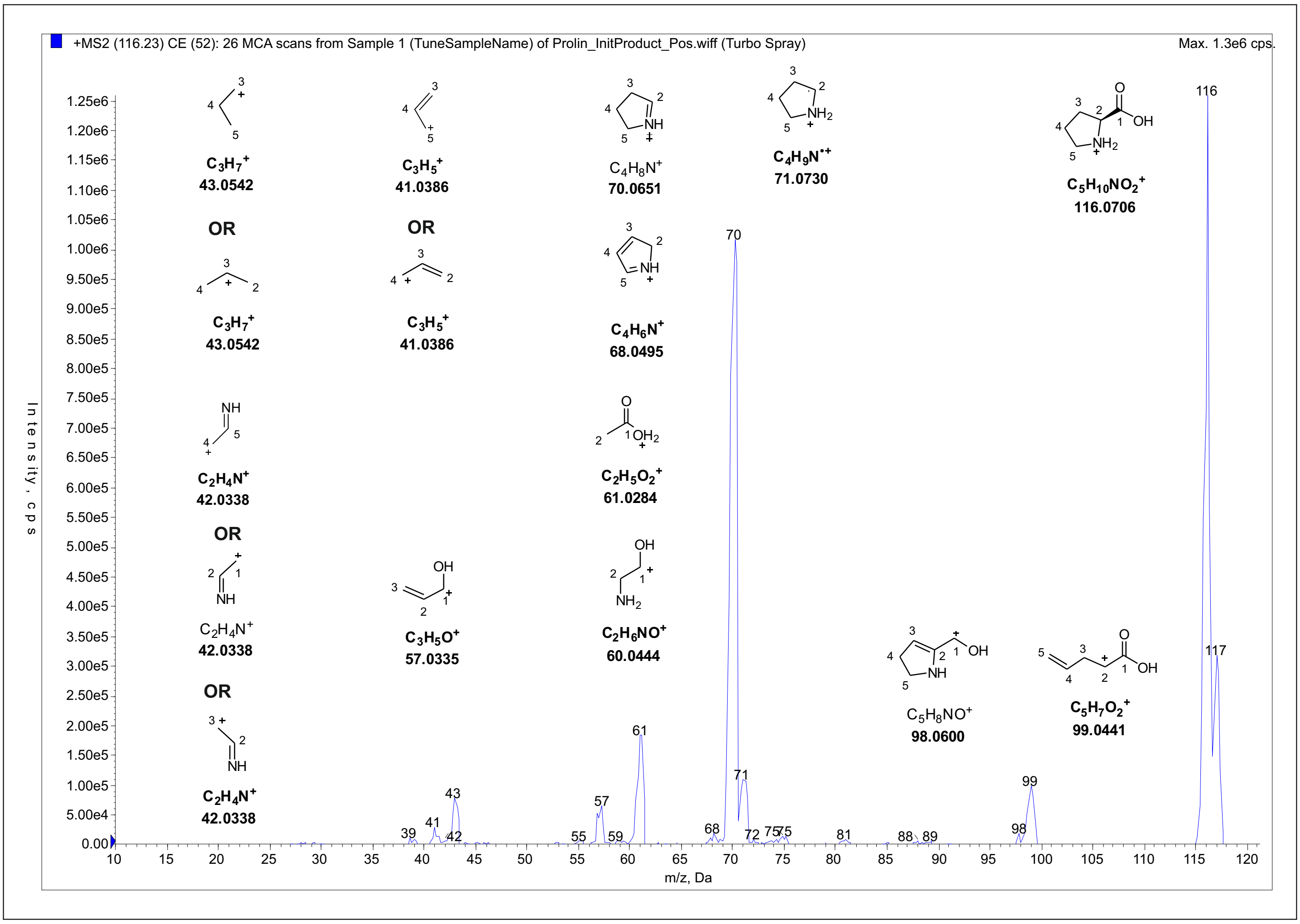

Chart S-62: The product ion spectrum of the [M+H]+ ion of proline.

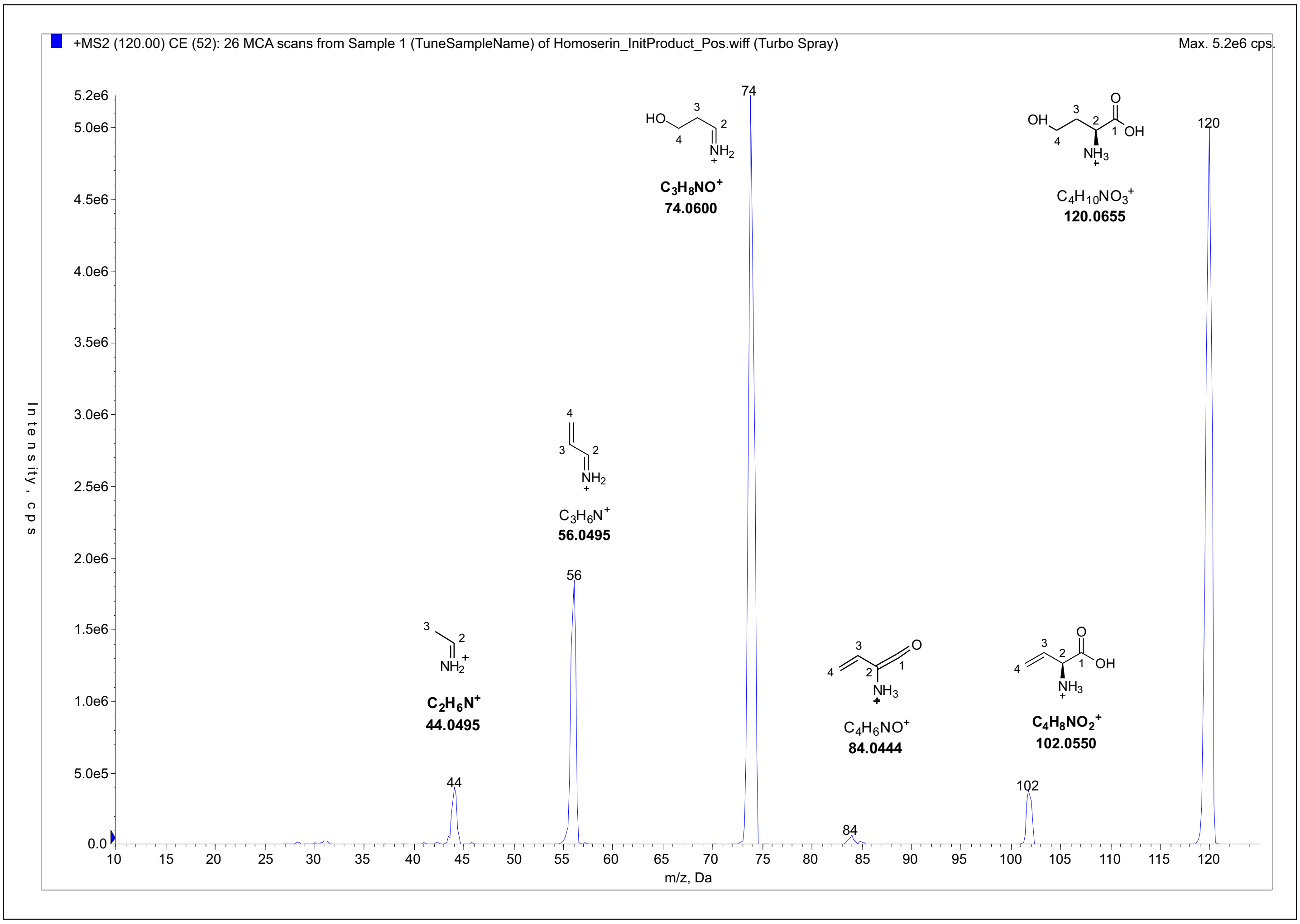

Chart S-63: The product ion spectrum of the [M+H]+ ion of homoserine.

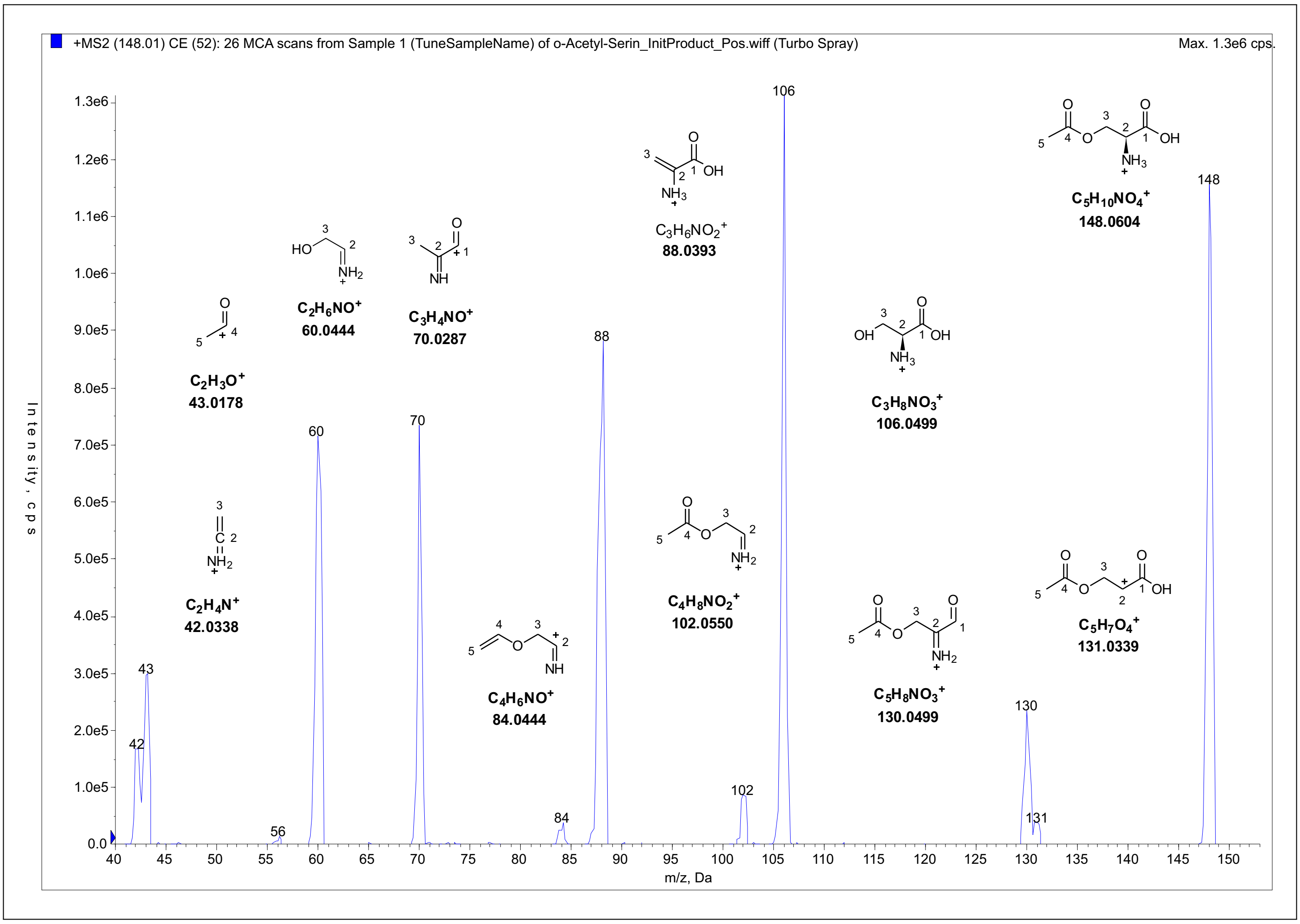

Chart S-64: The product ion spectrum of the [M+H]+ ion of O-acetyl-serine.

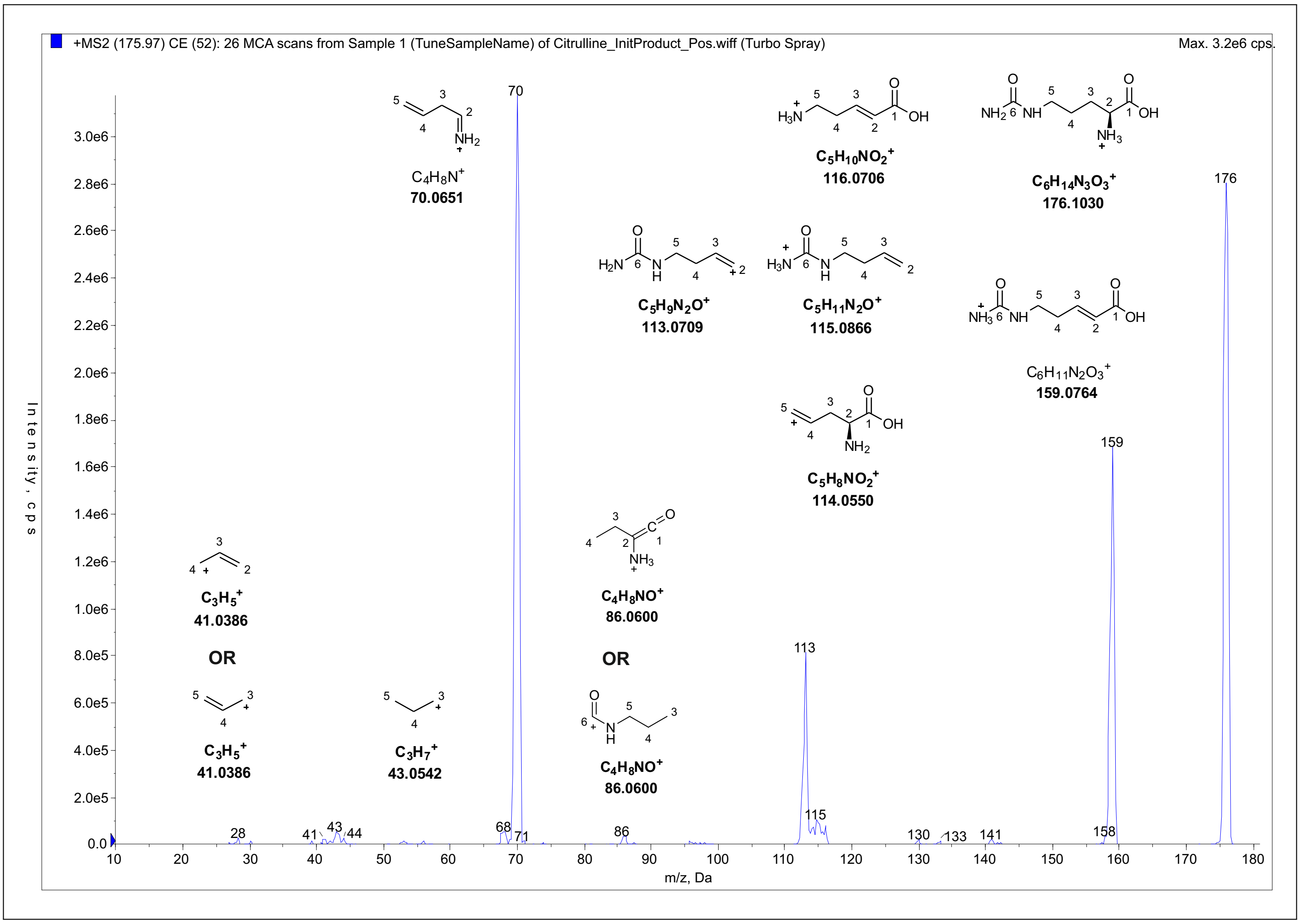

Chart S-65: The product ion spectrum of the [M+H]+ ion of citrulline.

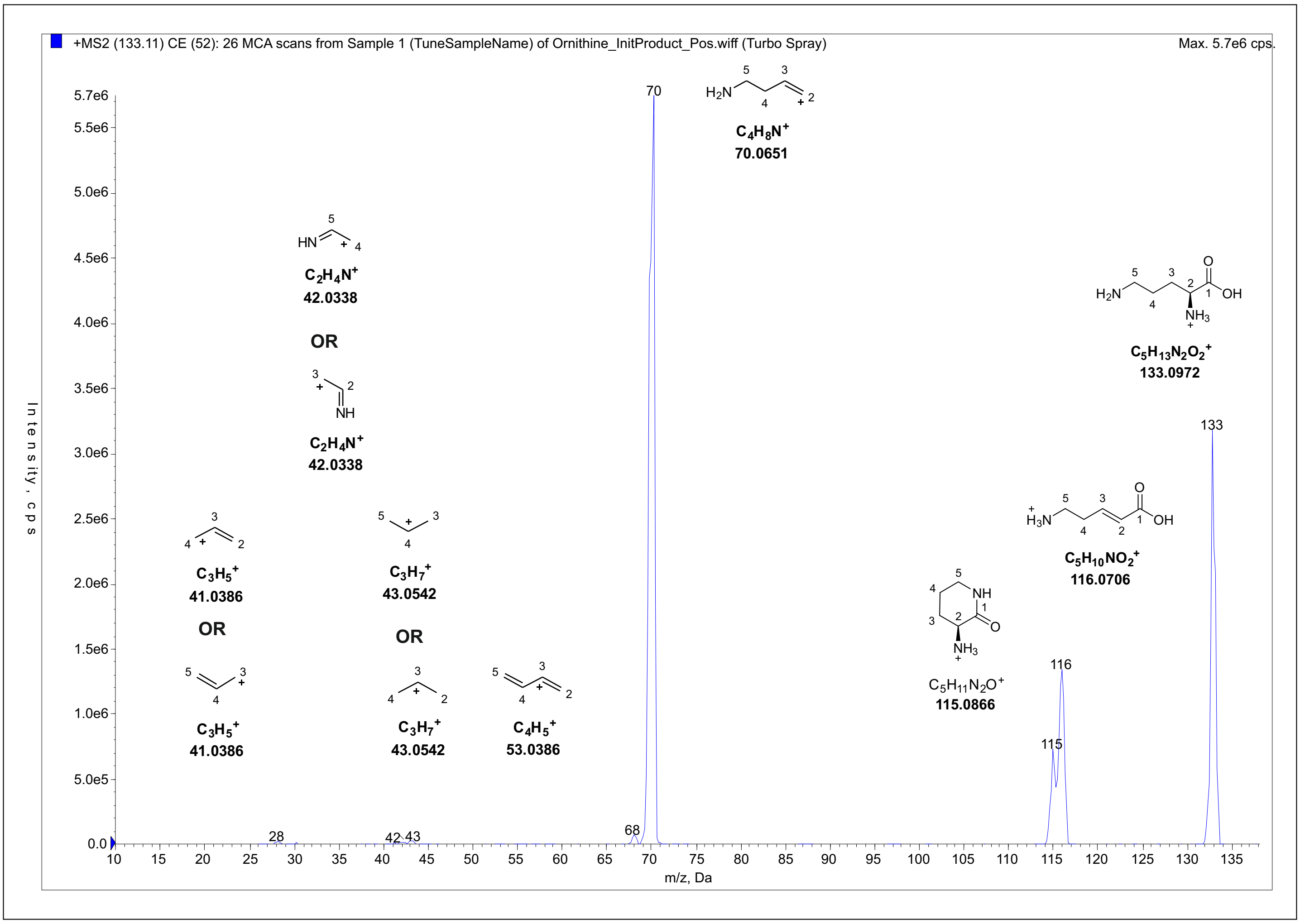

Chart S-66: The product ion spectrum of the [M+H]+ ion of ornithine.

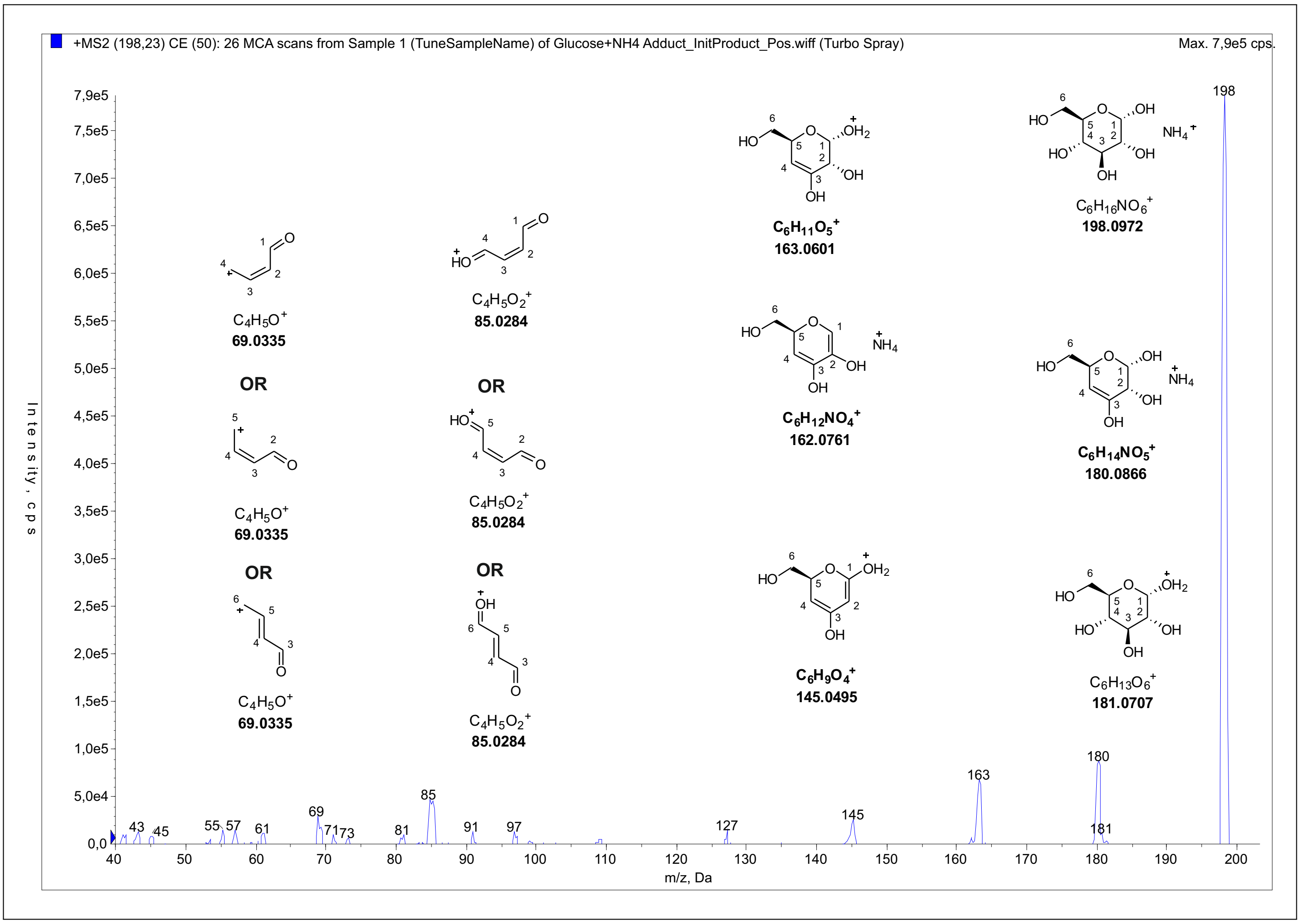

Chart S-67: The product ion spectrum of the [M+NH4]+ ion of glucose.

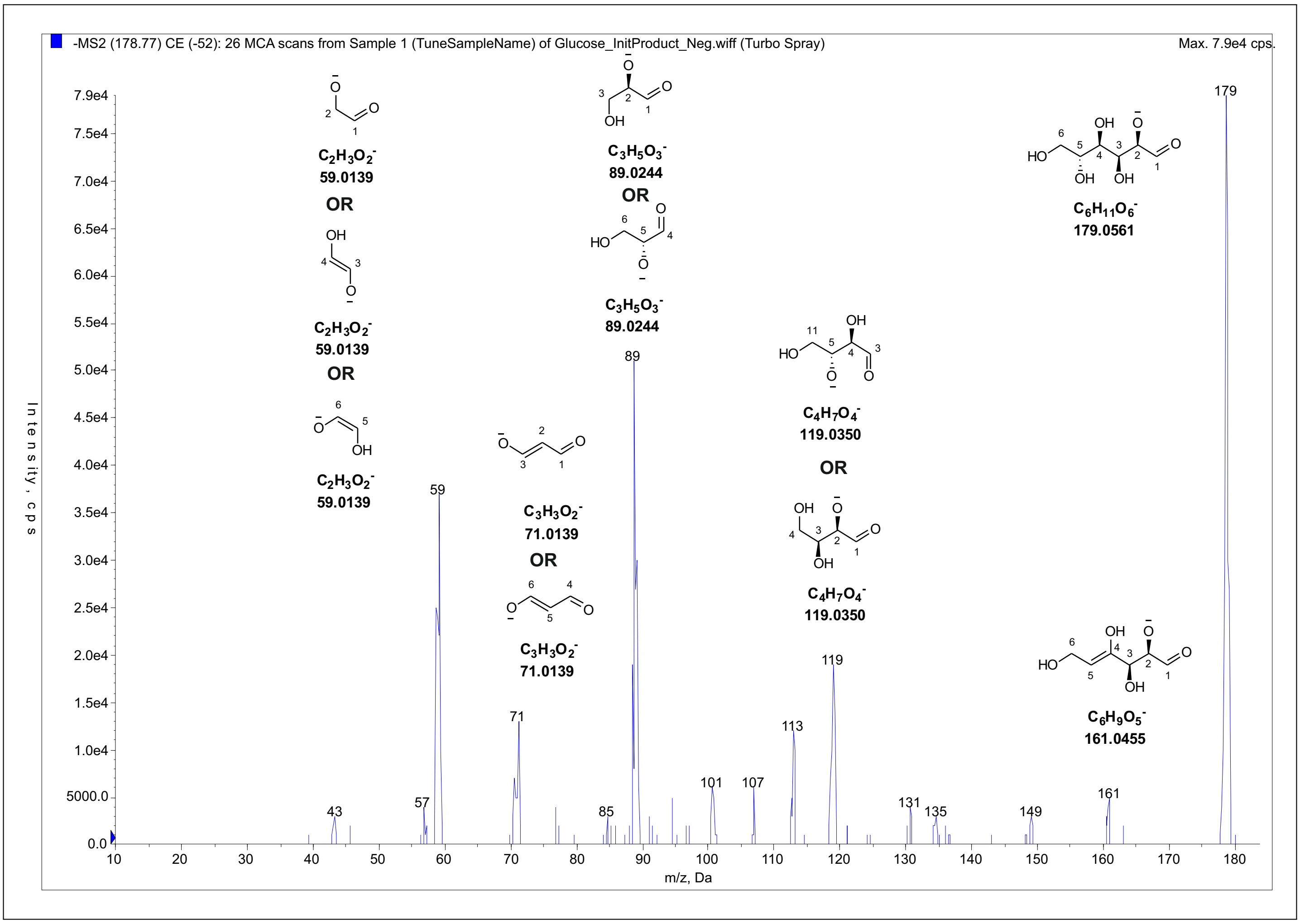

Chart S-68: The product ion spectrum of the [M-H]+ ion of glucose.

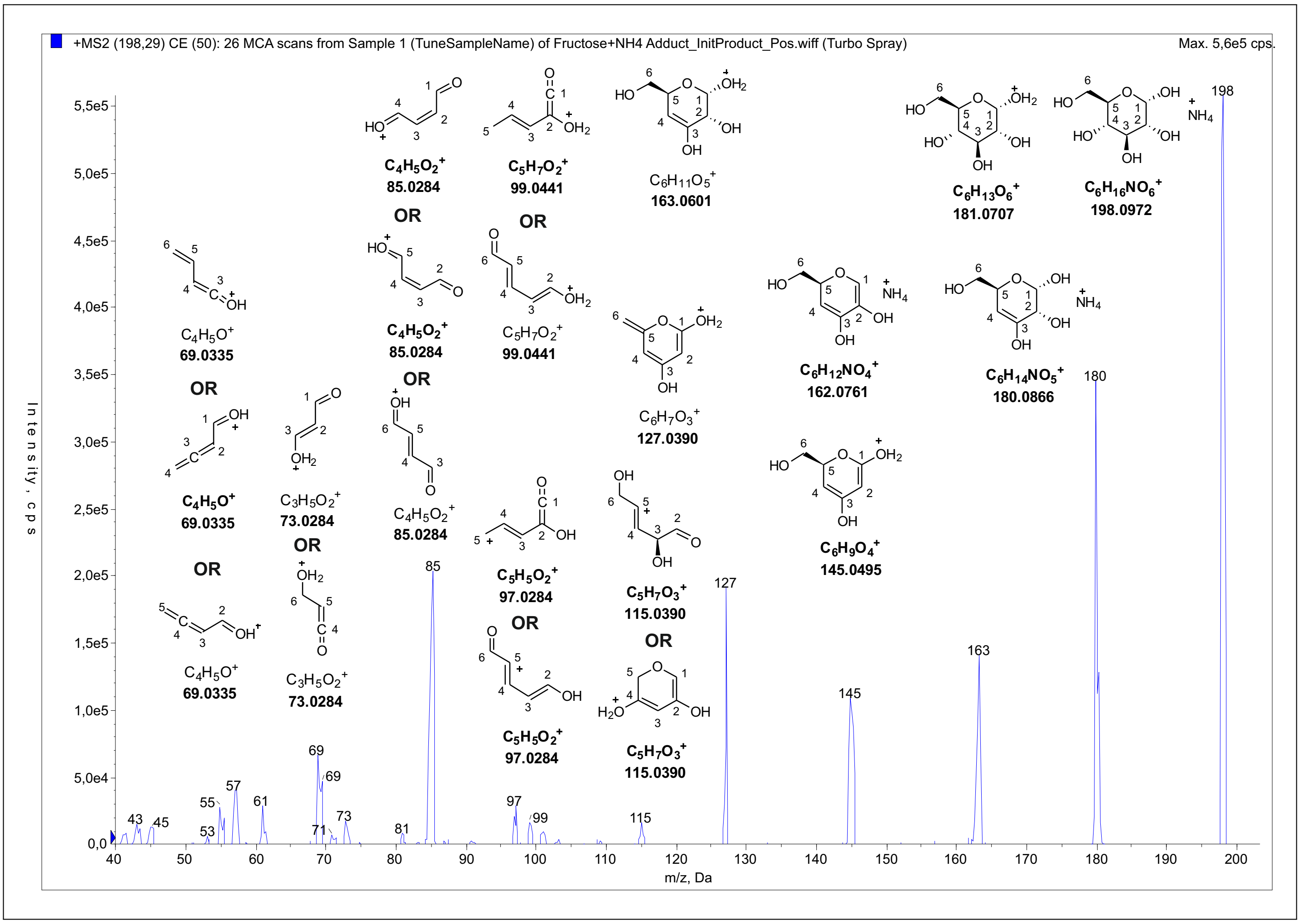

Chart S-69: The product ion spectrum of the [M+NH4]+ ion of fructose.

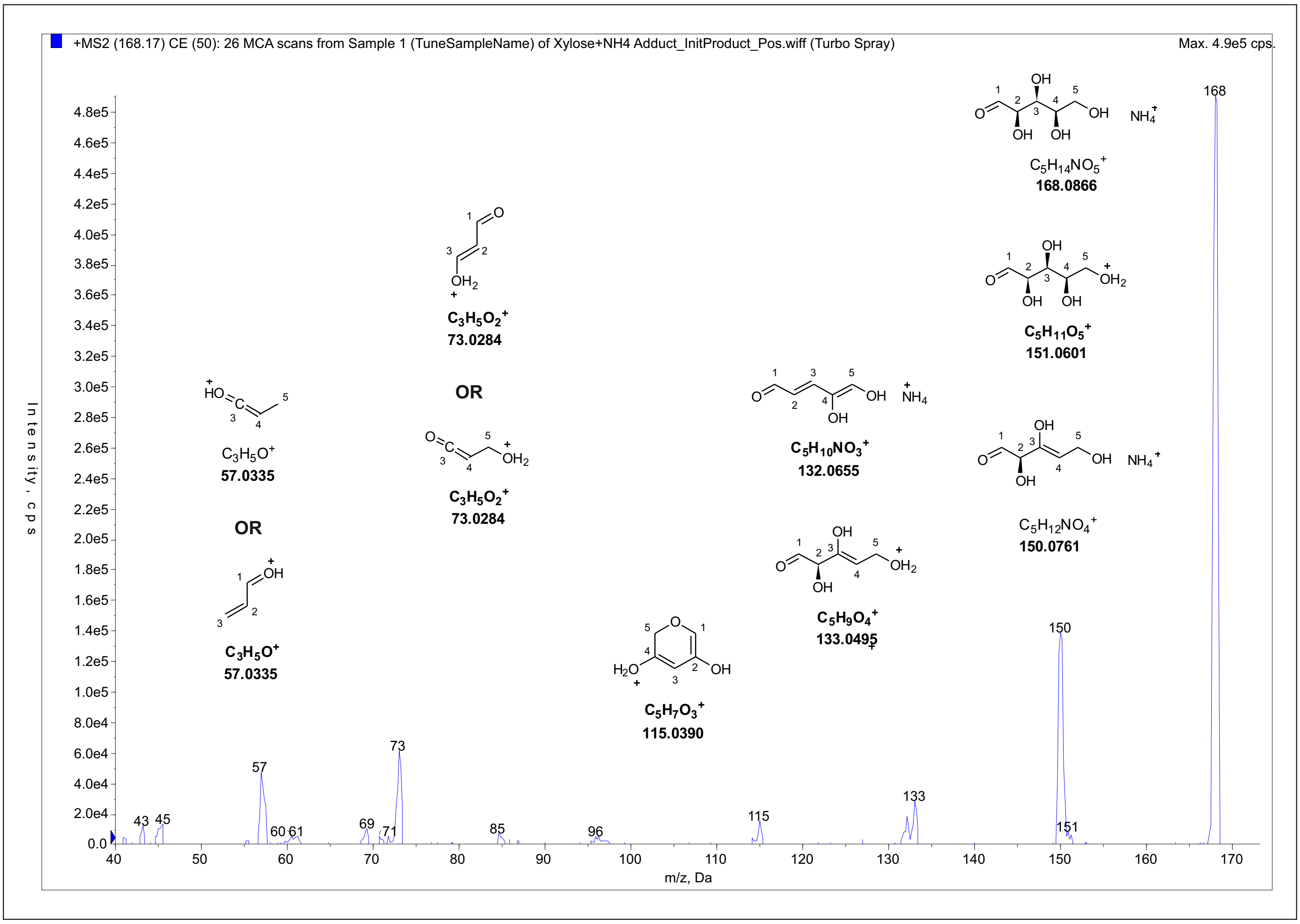

Chart S-70: The product ion spectrum of the [M+NH4]+ ion of xylose.

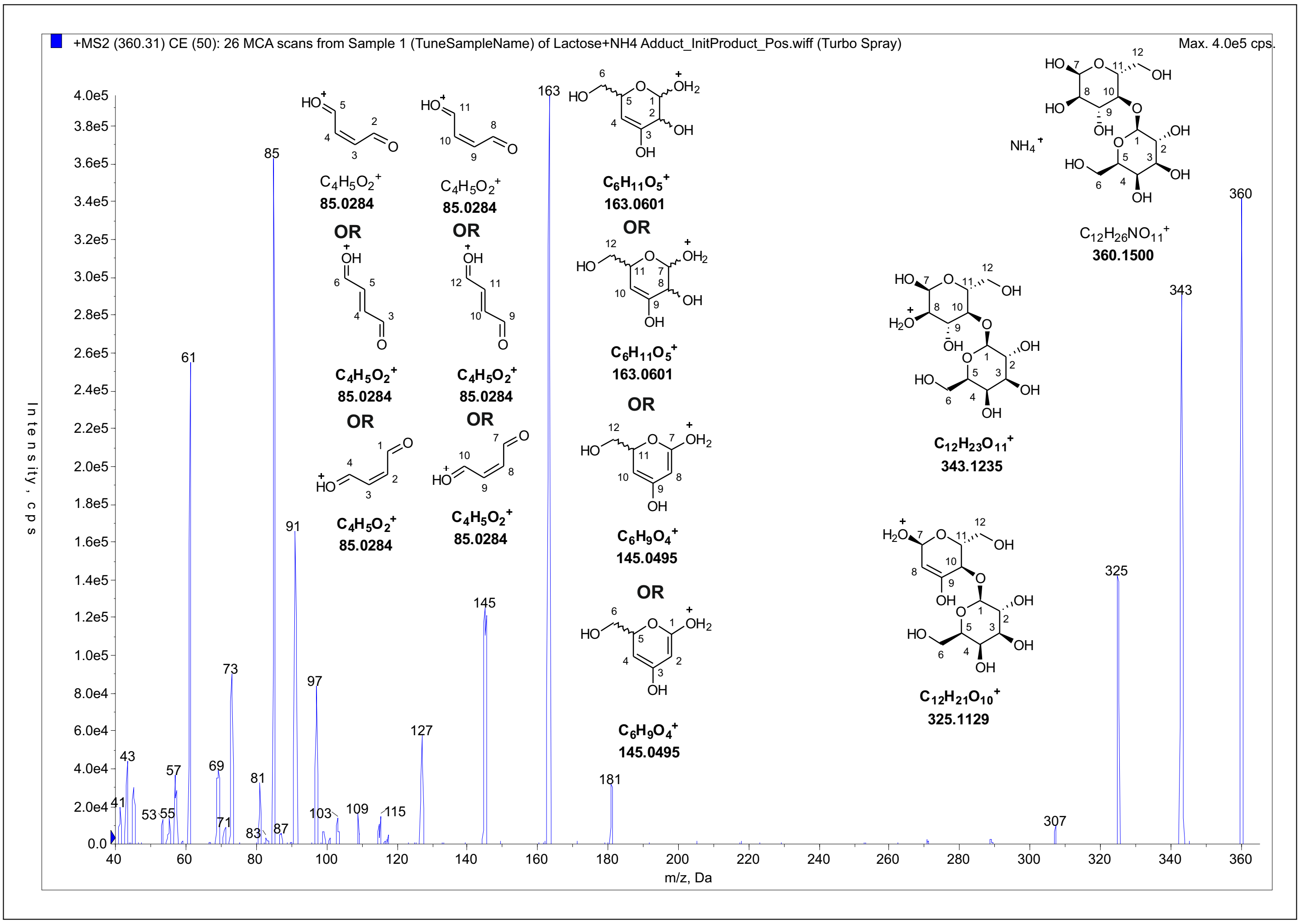

Chart S-71: The product ion spectrum of the [M+NH4]+ ion of lactose.

Supplementary Spectra

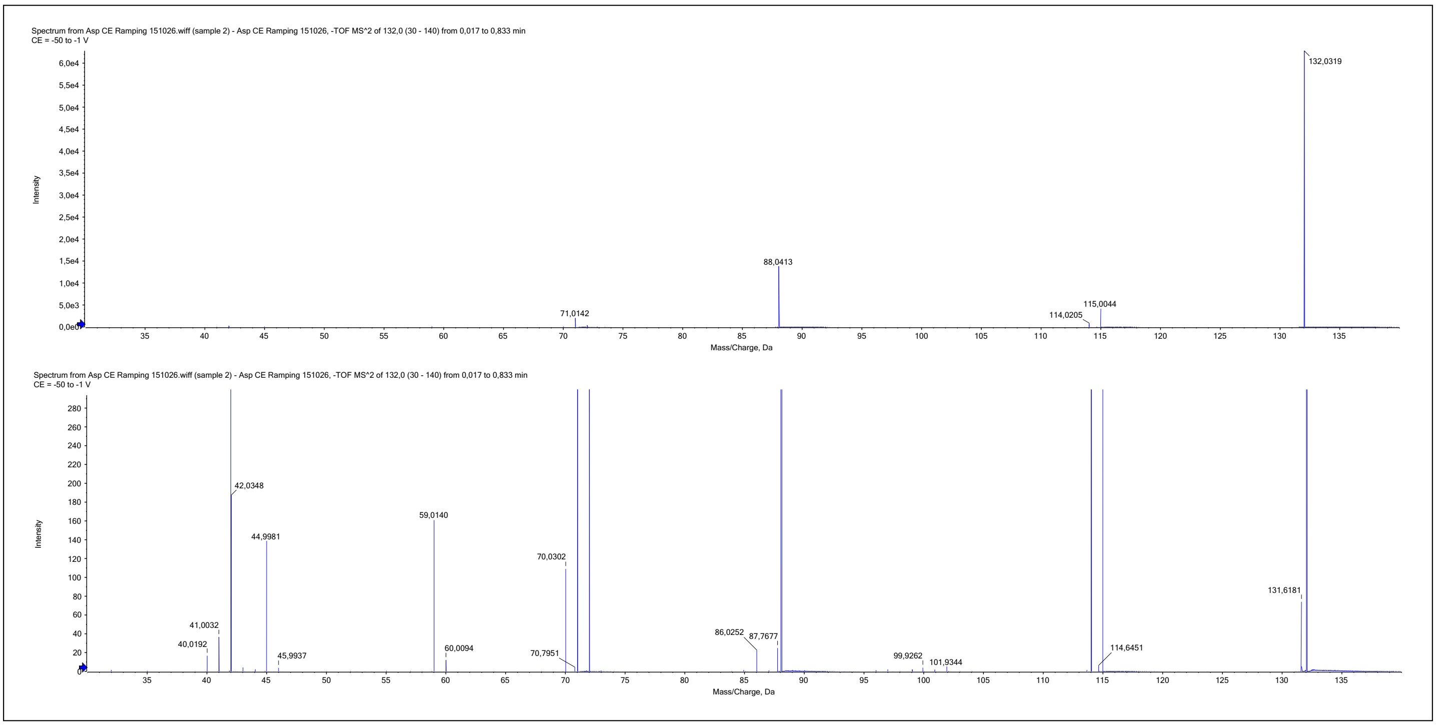

Chart S-72: The QTOF-product ion spectrum of the [M-H]-ion of aspartate.

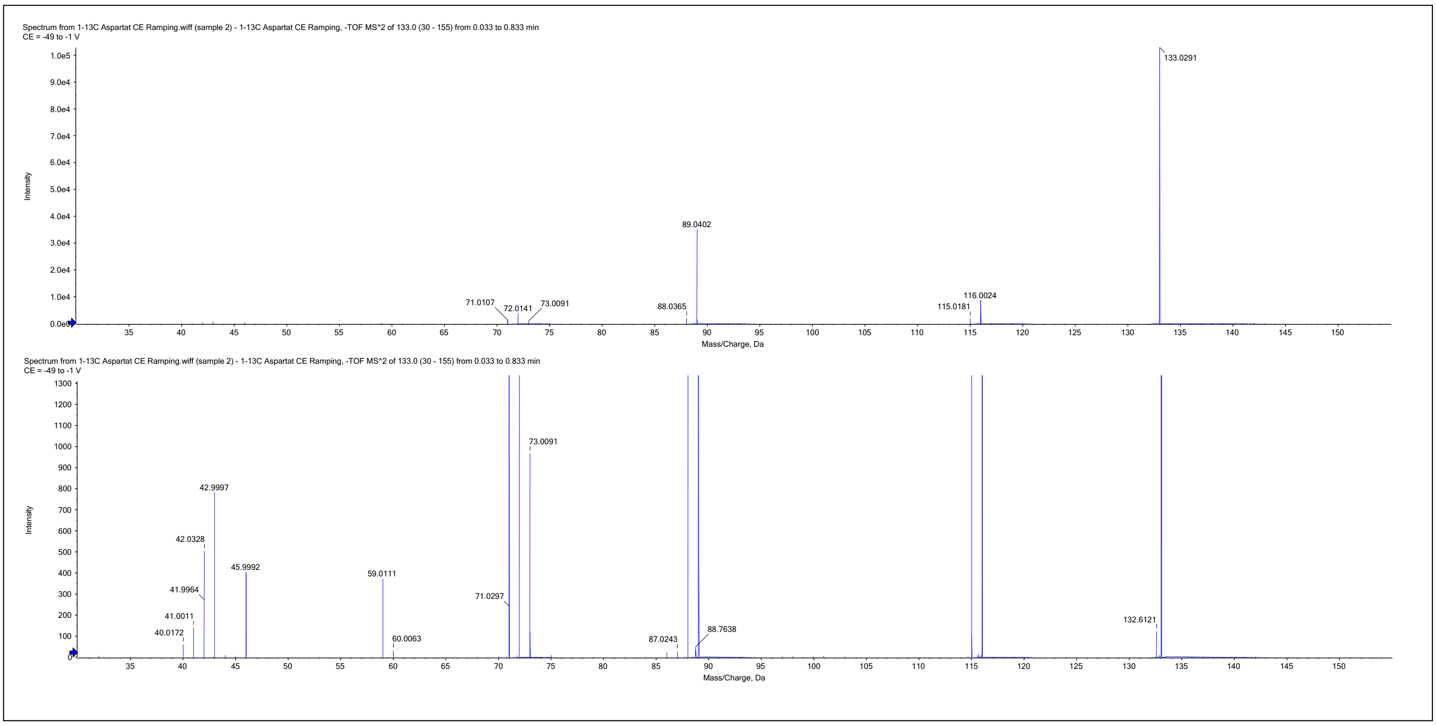

Chart S-73: The QTOF-product ion spectrum of the [M-H]-ion of [1-^{13}C]aspartate.

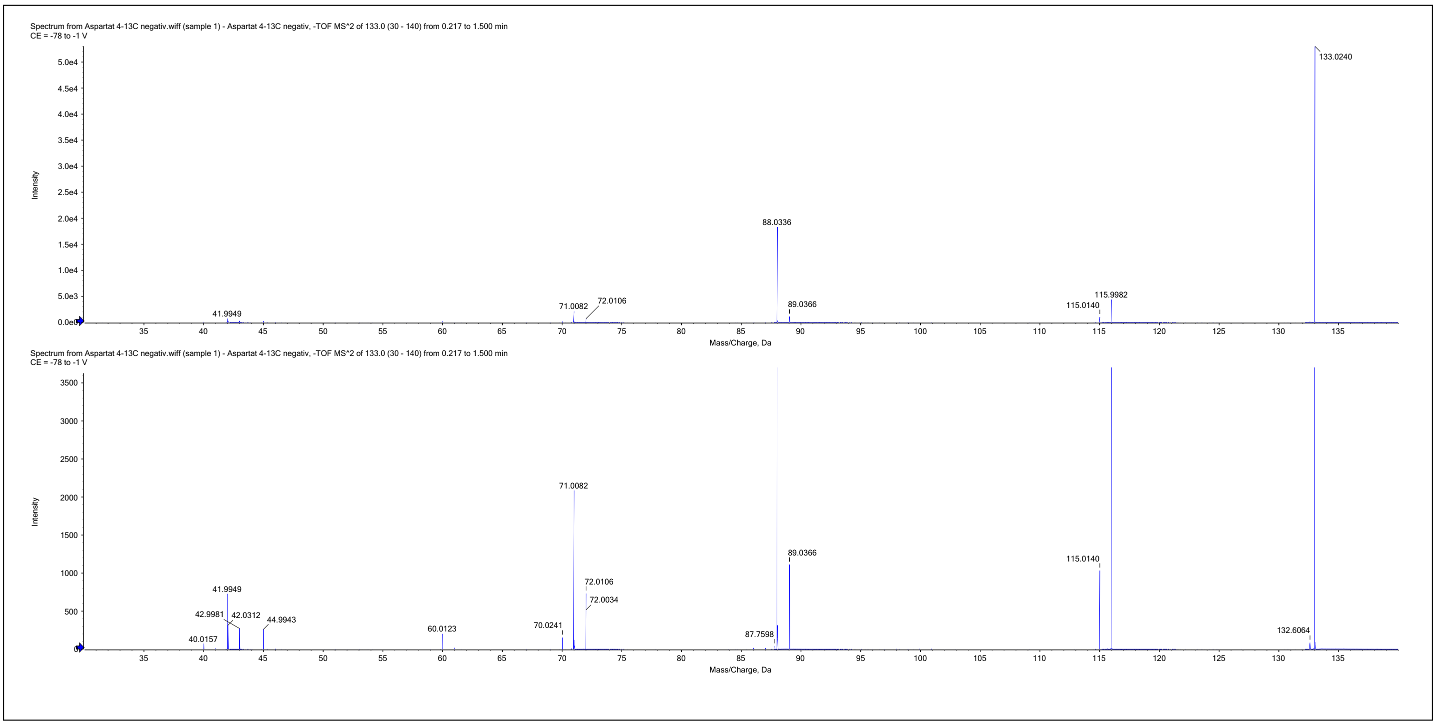

Chart S-74: The QTOF product ion spectrum of the [M-H]-ion of [4-^{13}C]aspartate.

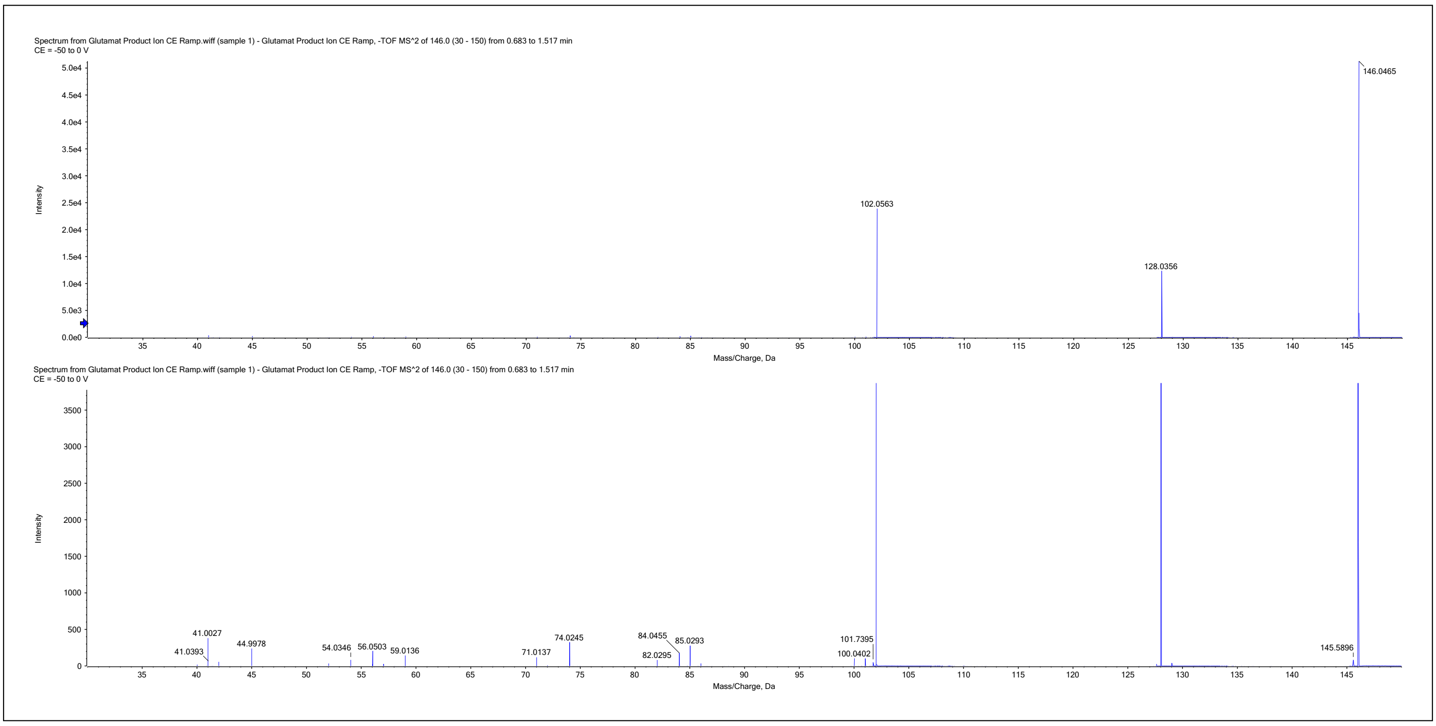

Chart S-75: The QTOF product ion spectrum of the [M-H]-ion of glutamate.

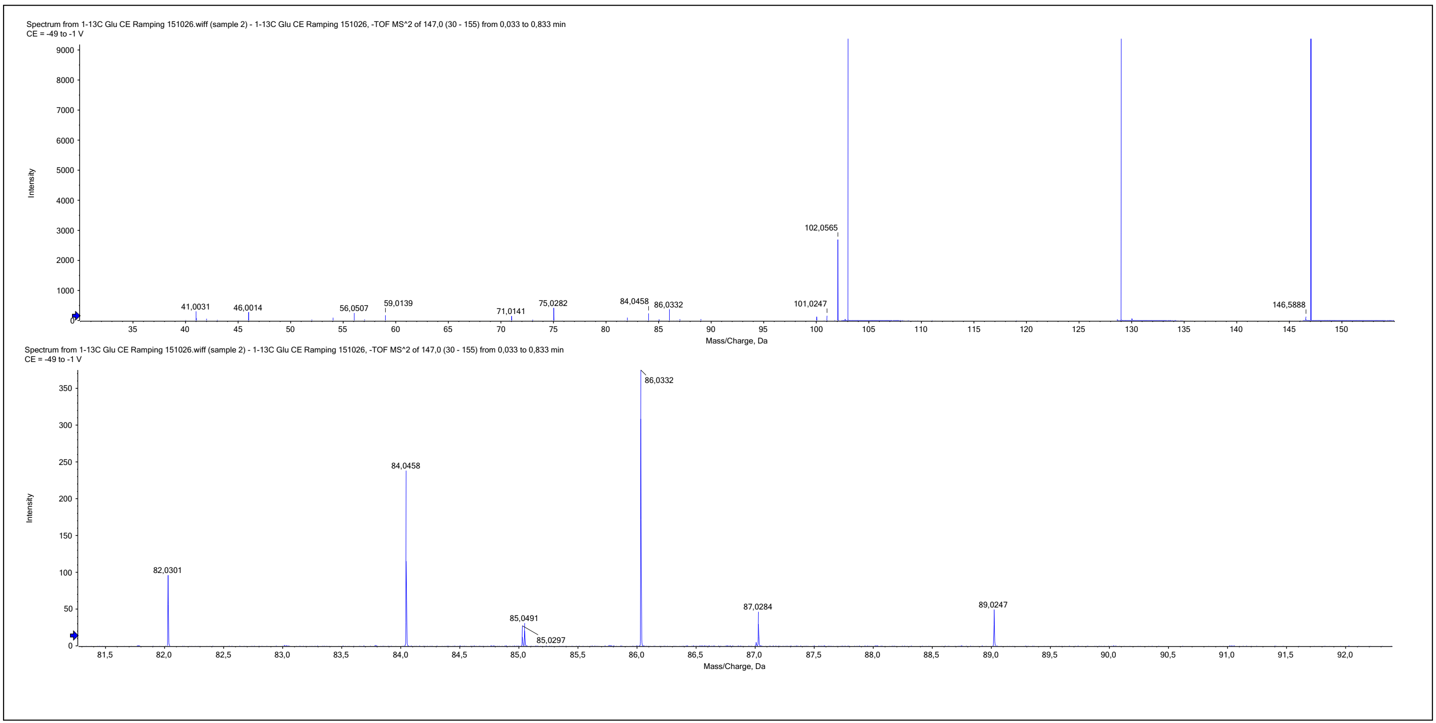

Chart S-76: The QTOF product ion spectrum of the [M-H]-ion of [1-^{13}C]glutamate.

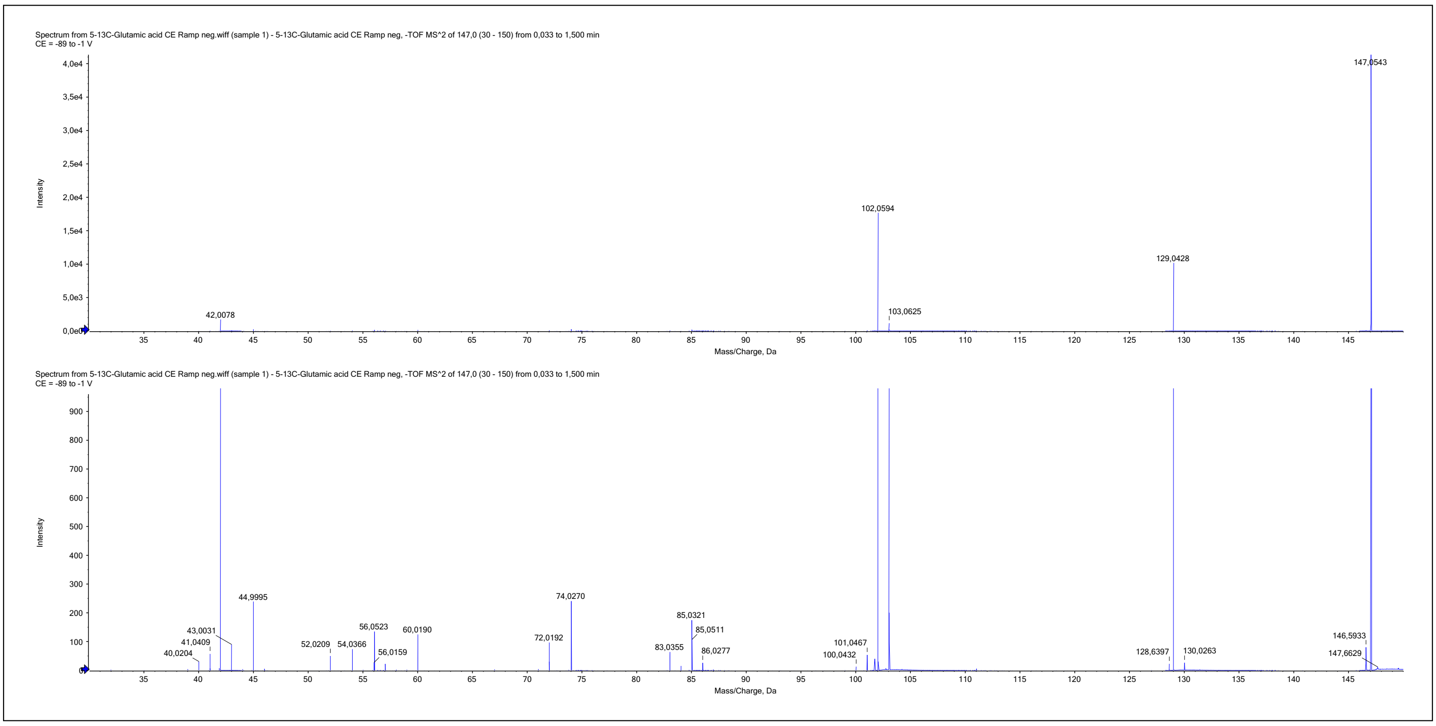

Chart S-77: The QTOF product ion spectrum of the [M-H]-ion of [5-^{13}C]glutamate.

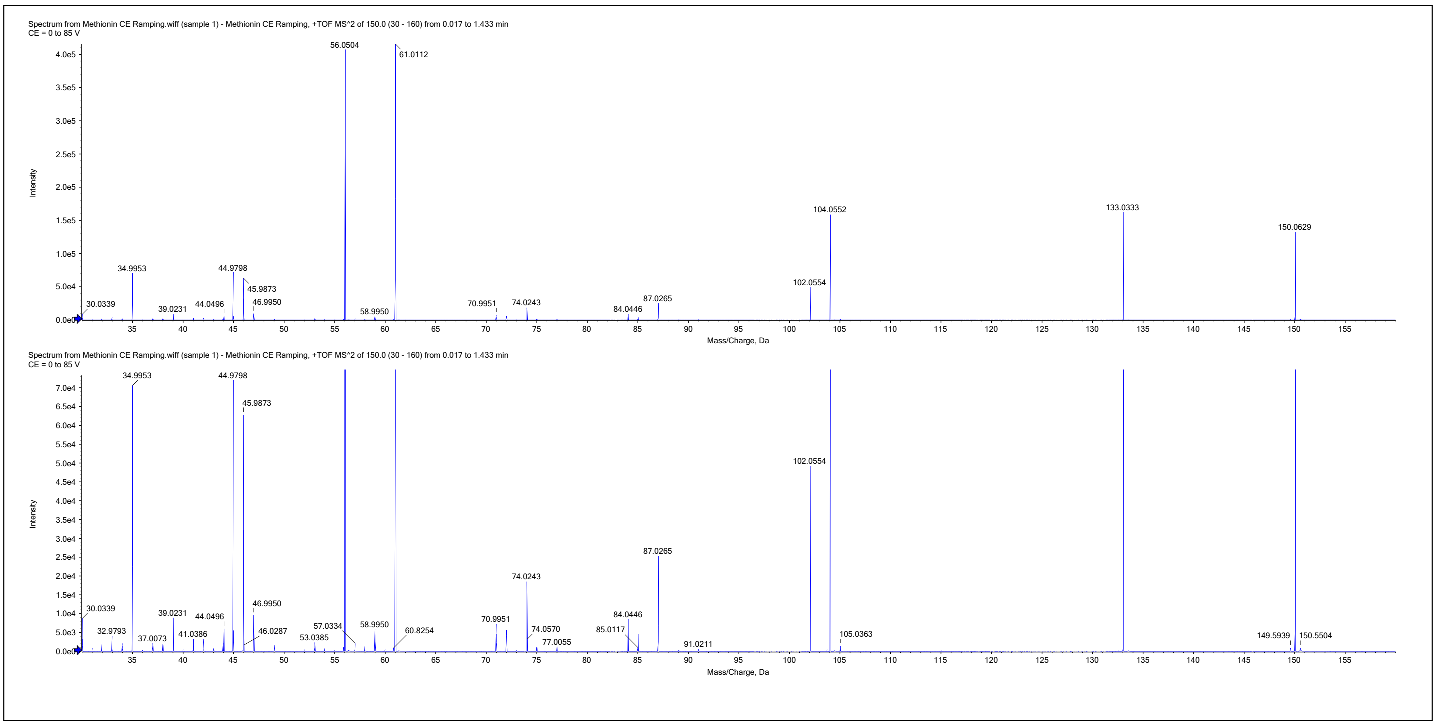

Chart S-78: The QTOF product ion spectrum of the [M+H]+ion of methionine.

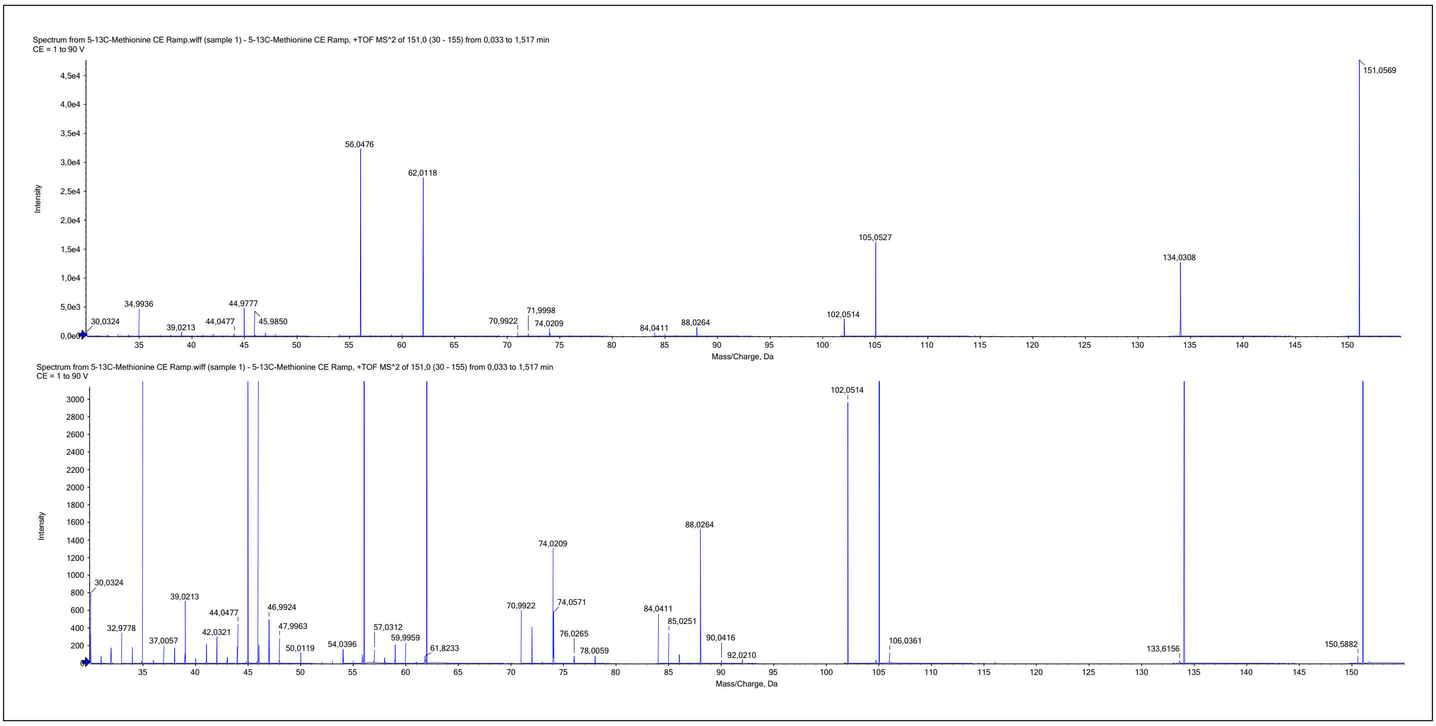

Chart S-79: The QTOF product ion spectrum of the [M-H]-ion of [5-^{13}C]methionine.

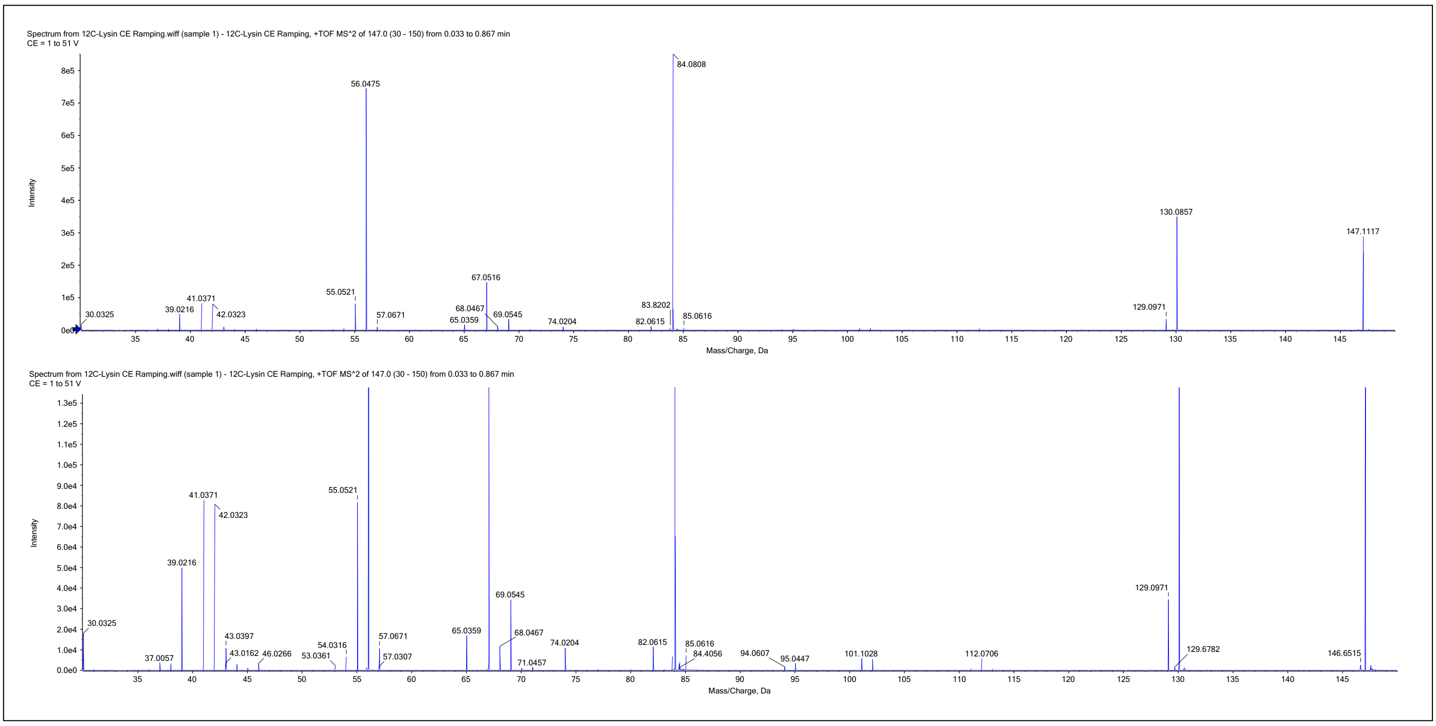

Chart S-80: The QTOF product ion spectrum of the [M+H]+ion of lysine.

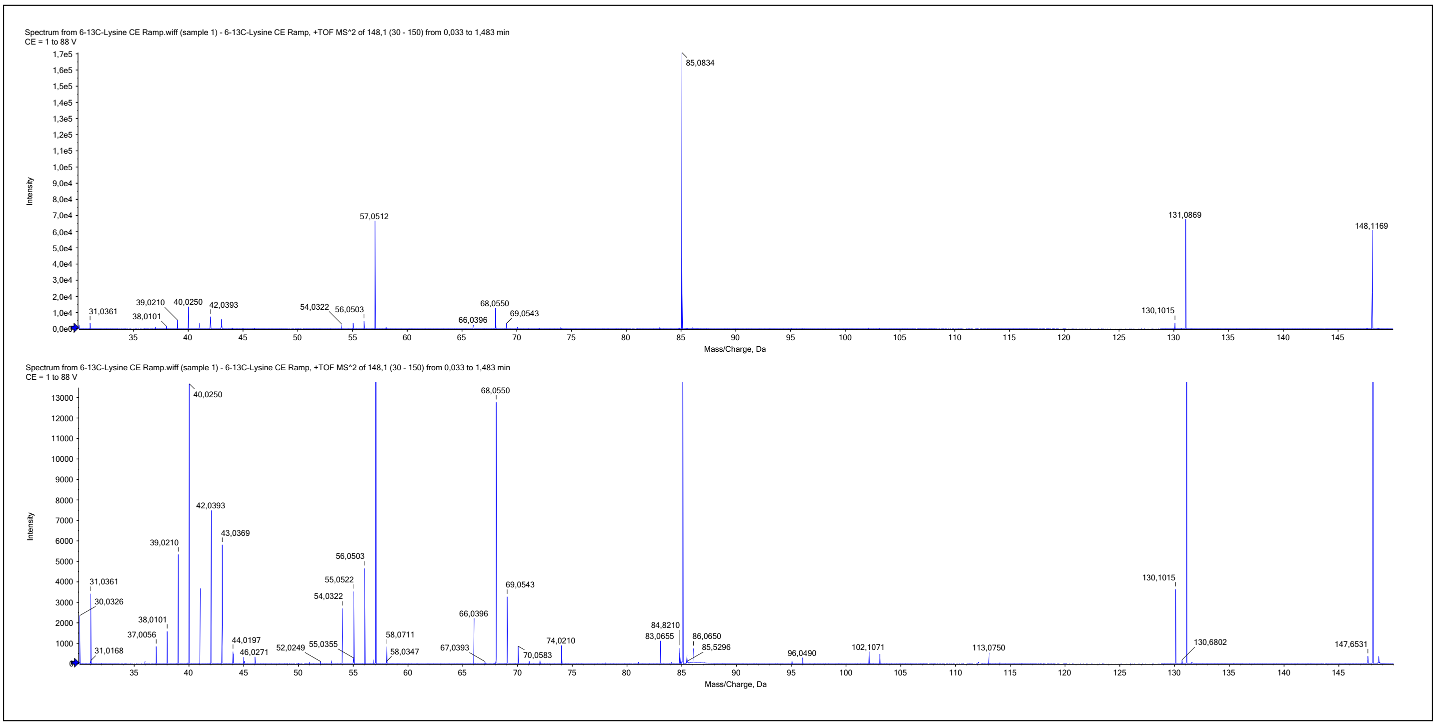

Chart S-81: The QTOF product ion spectrum of the [M-H]-ion of [6-^{13}C]lysine.

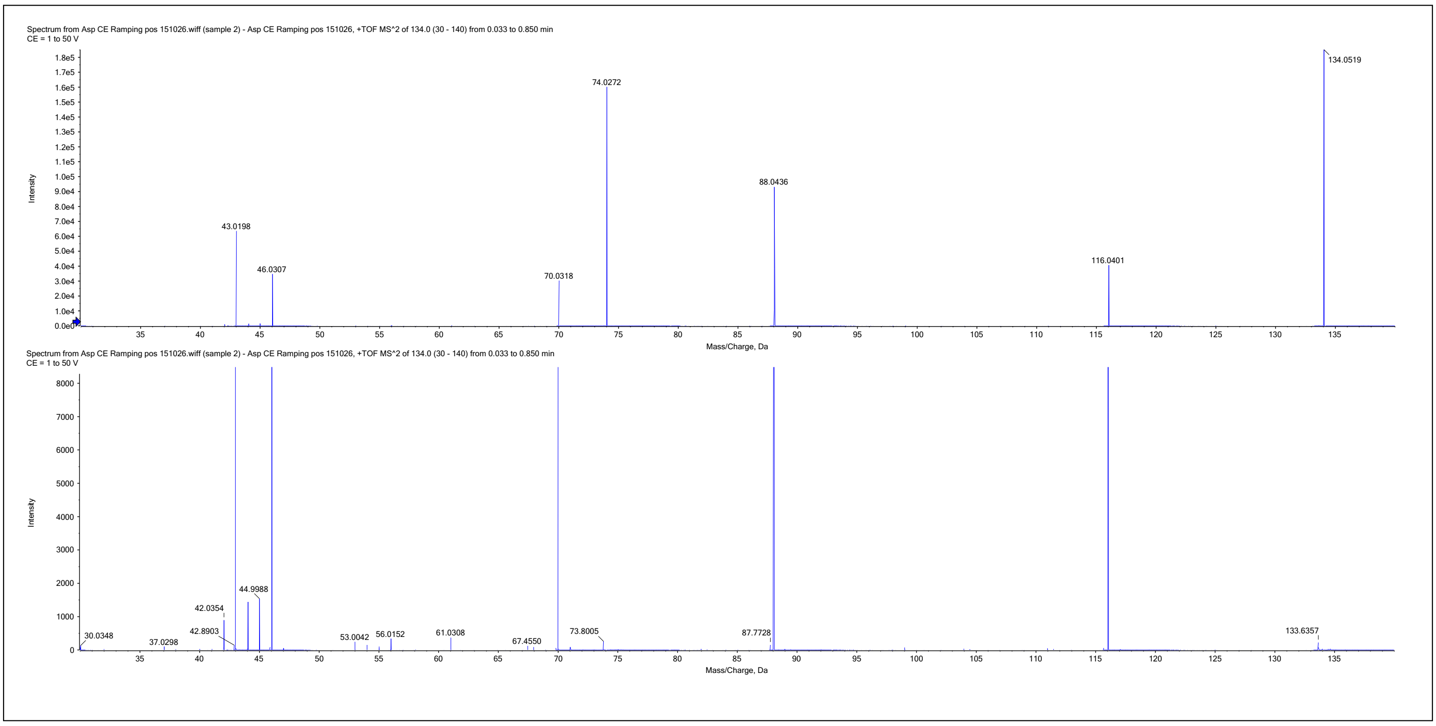

Chart S-82: The QTOF product ion spectrum of the [M-H]-ion of [6-^{13}C]lysine.

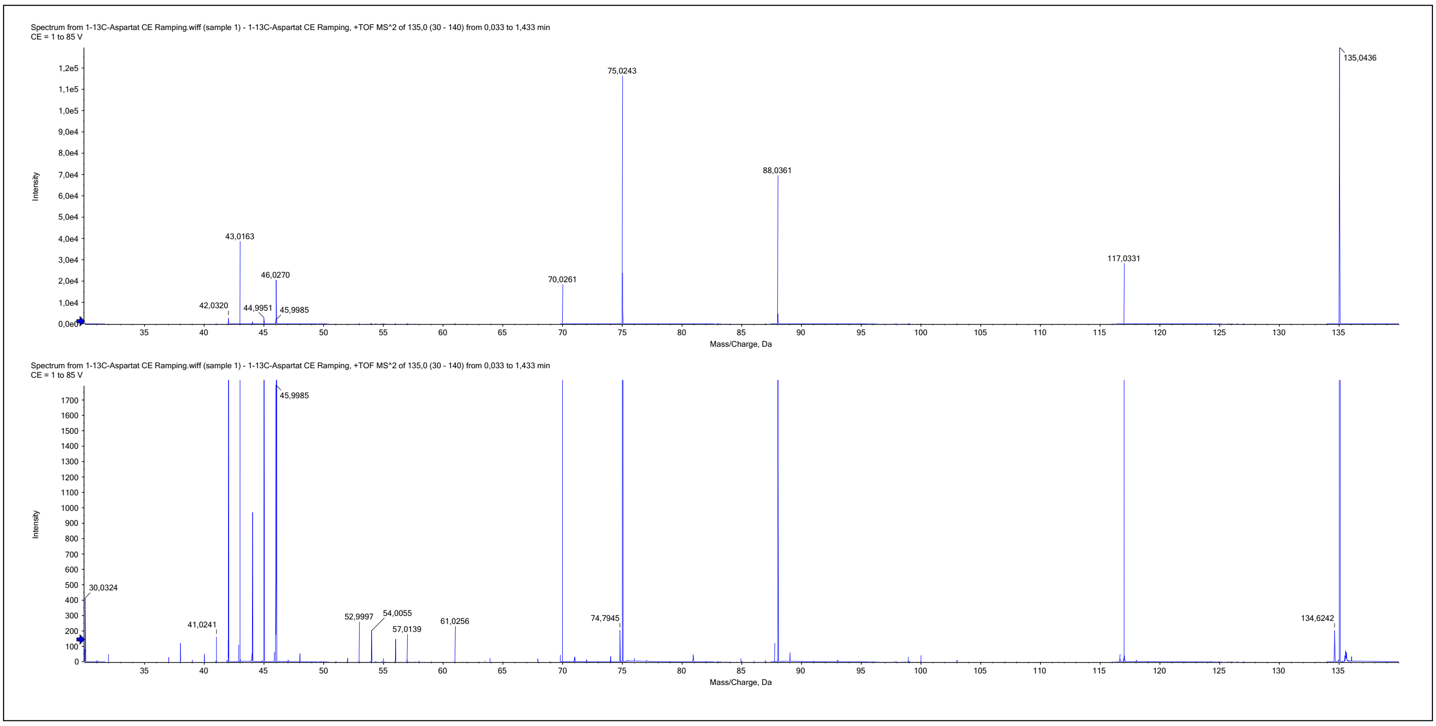

Chart S-83: The QTOF product ion spectrum of the [M-H]-ion of [6-^{13}C]lysine.

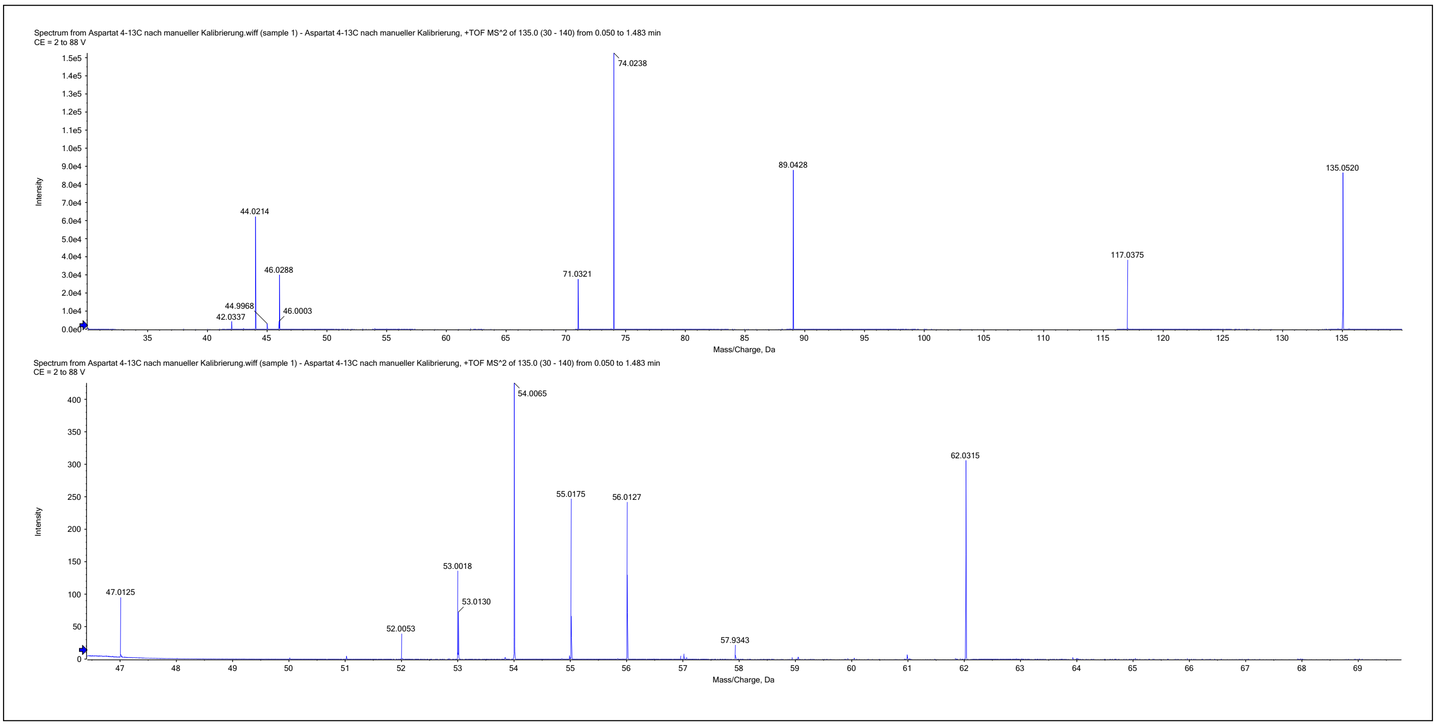

Chart S-84: The QTOF product ion spectrum of the [M-H]-ion of [6-^{13}C]lysine.

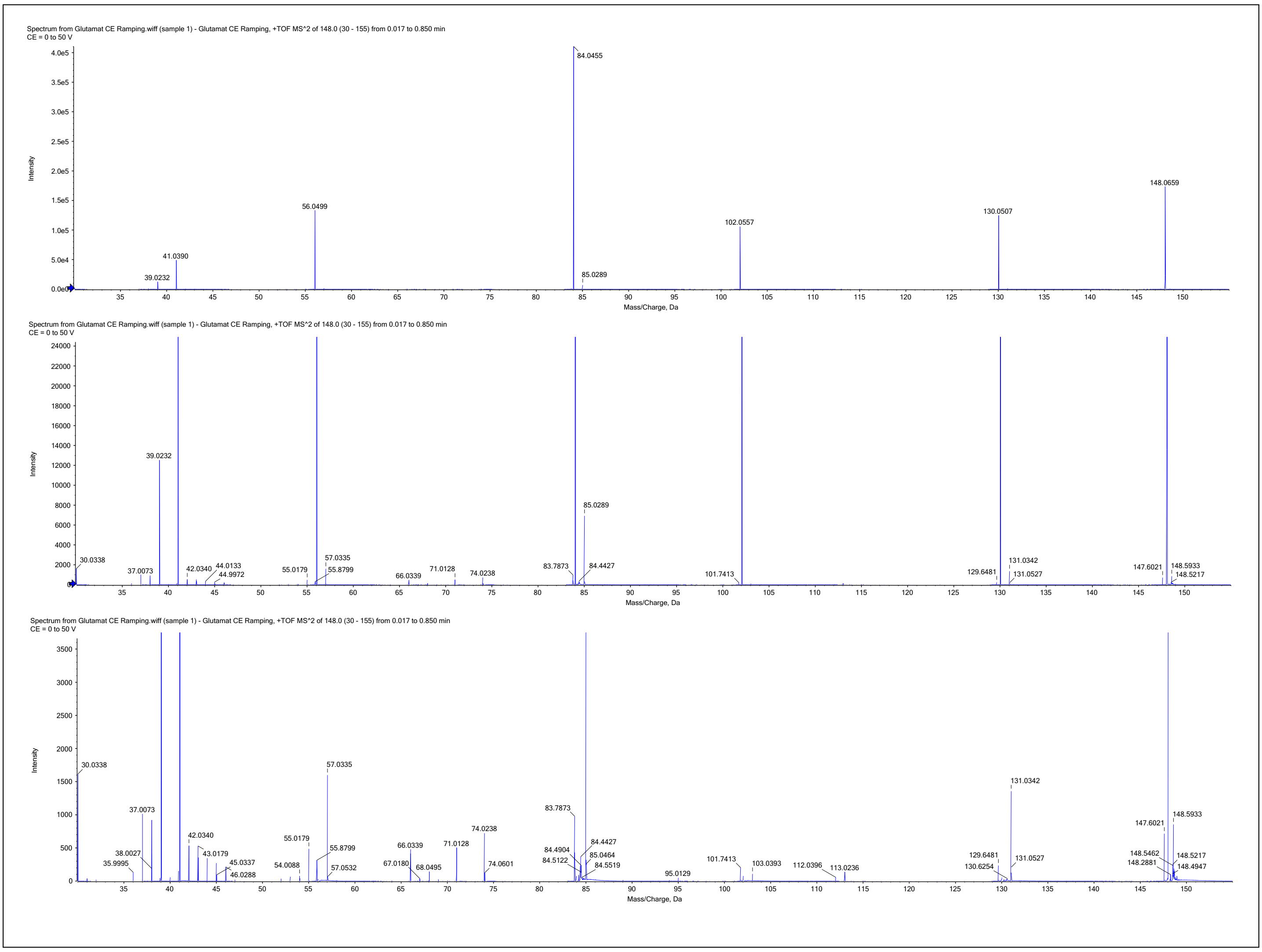

Chart S-85: The QTOF product ion spectrum of the [M-H]-ion of glutamate.

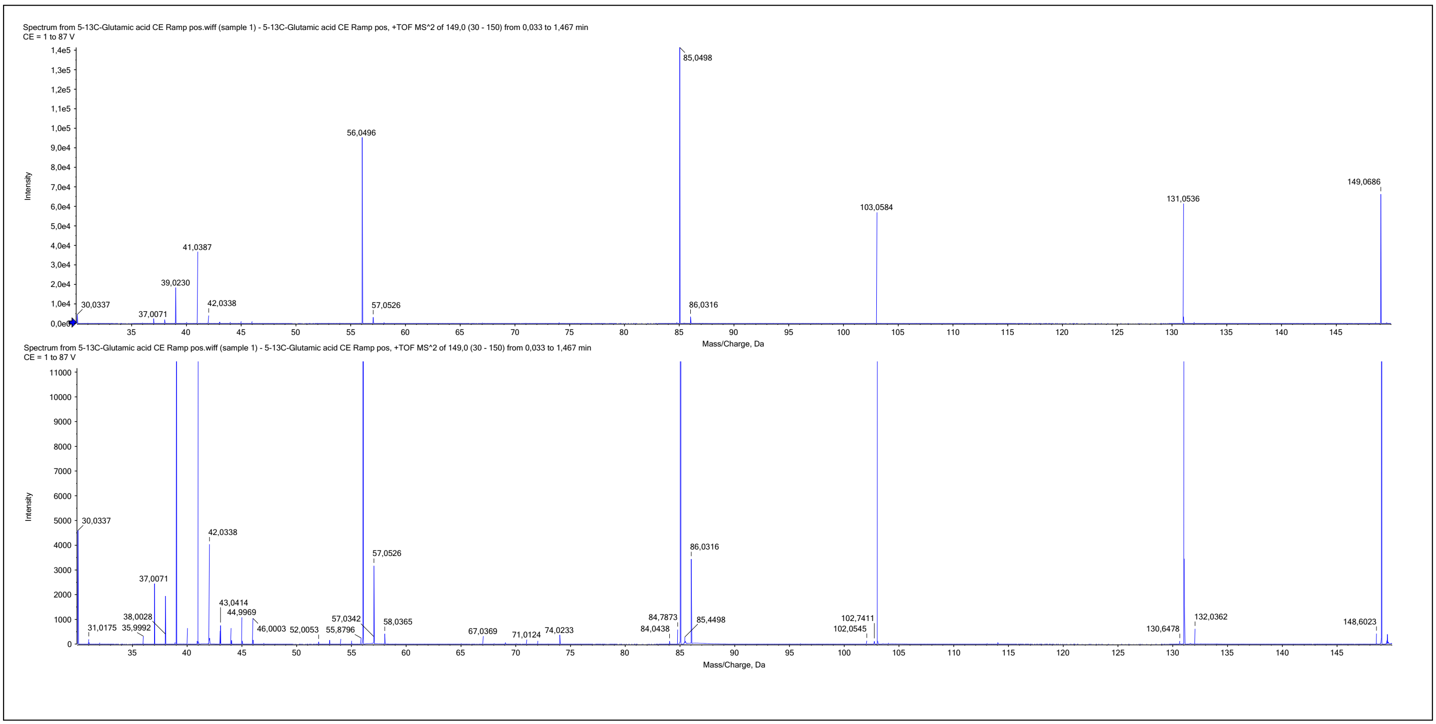

Chart S-86: The QTOF product ion spectrum of the [M-H]-ion of [5-^{13}C]glutamate.

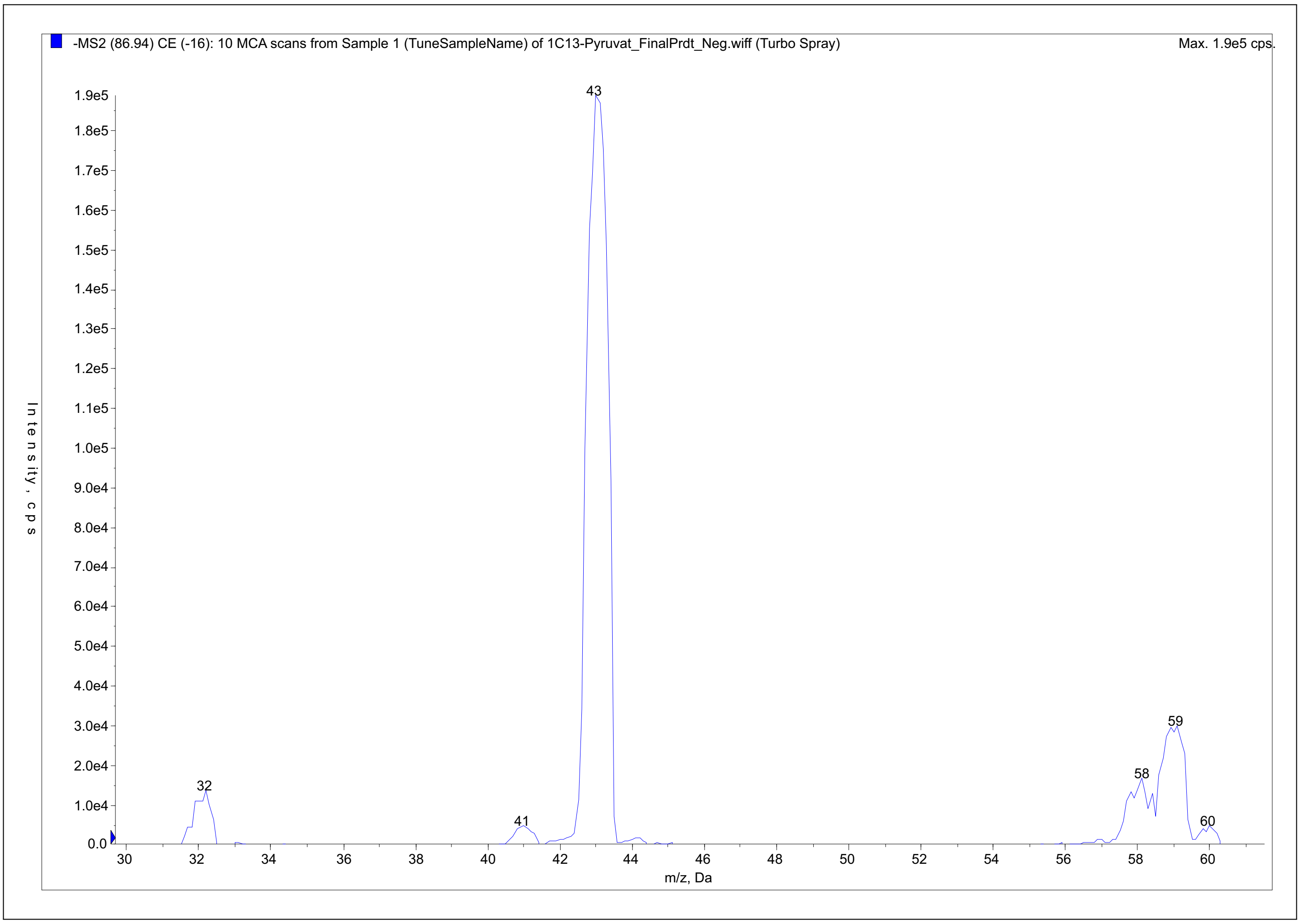

Chart S-87: The QqQ product ion spectrum of the [M-H]-ion of [1-^{13}C]pyruvate.

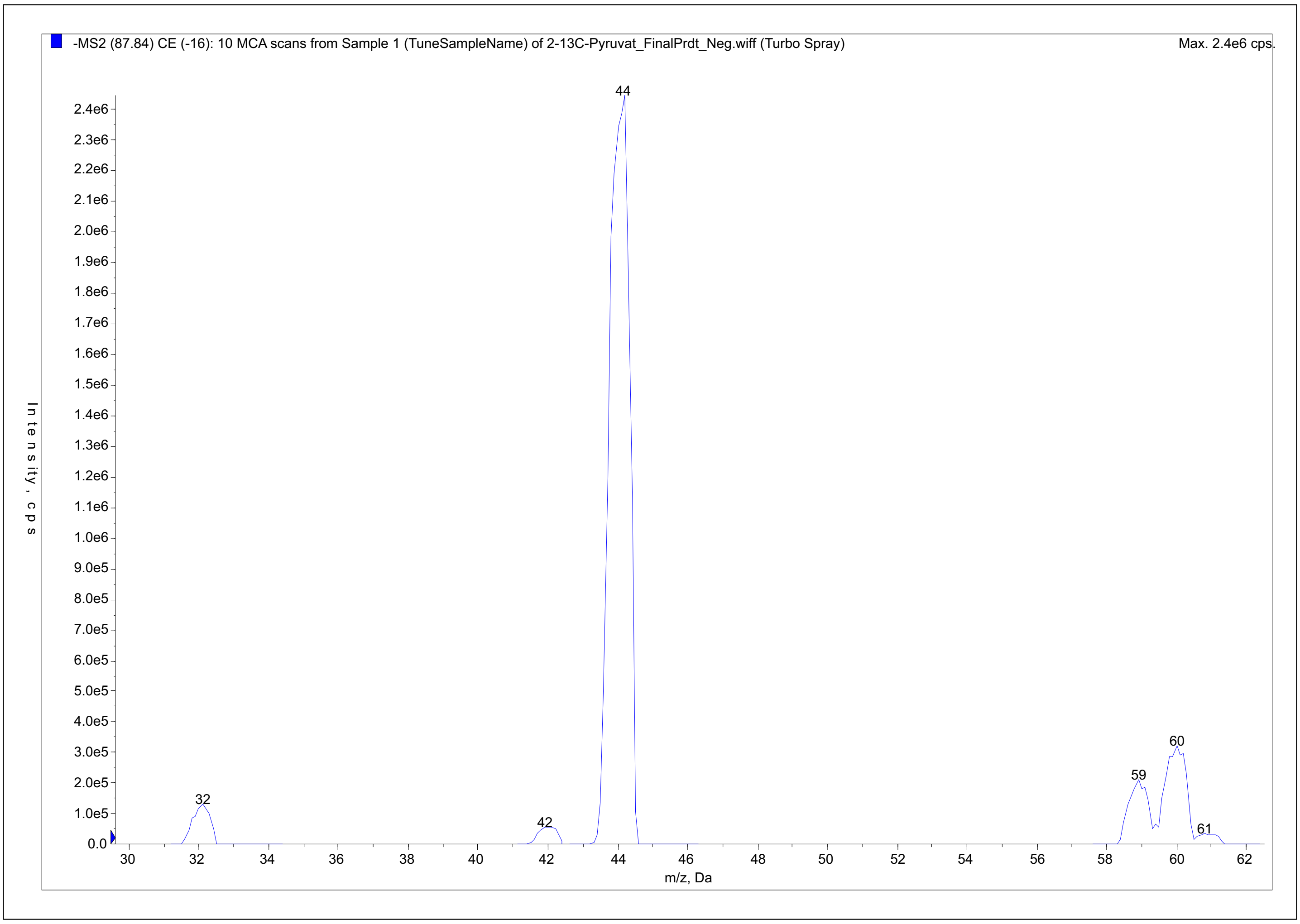

Chart S-88: The QqQ product ion spectrum of the [M-H]-ion of [2-^{13}C]pyruvate.

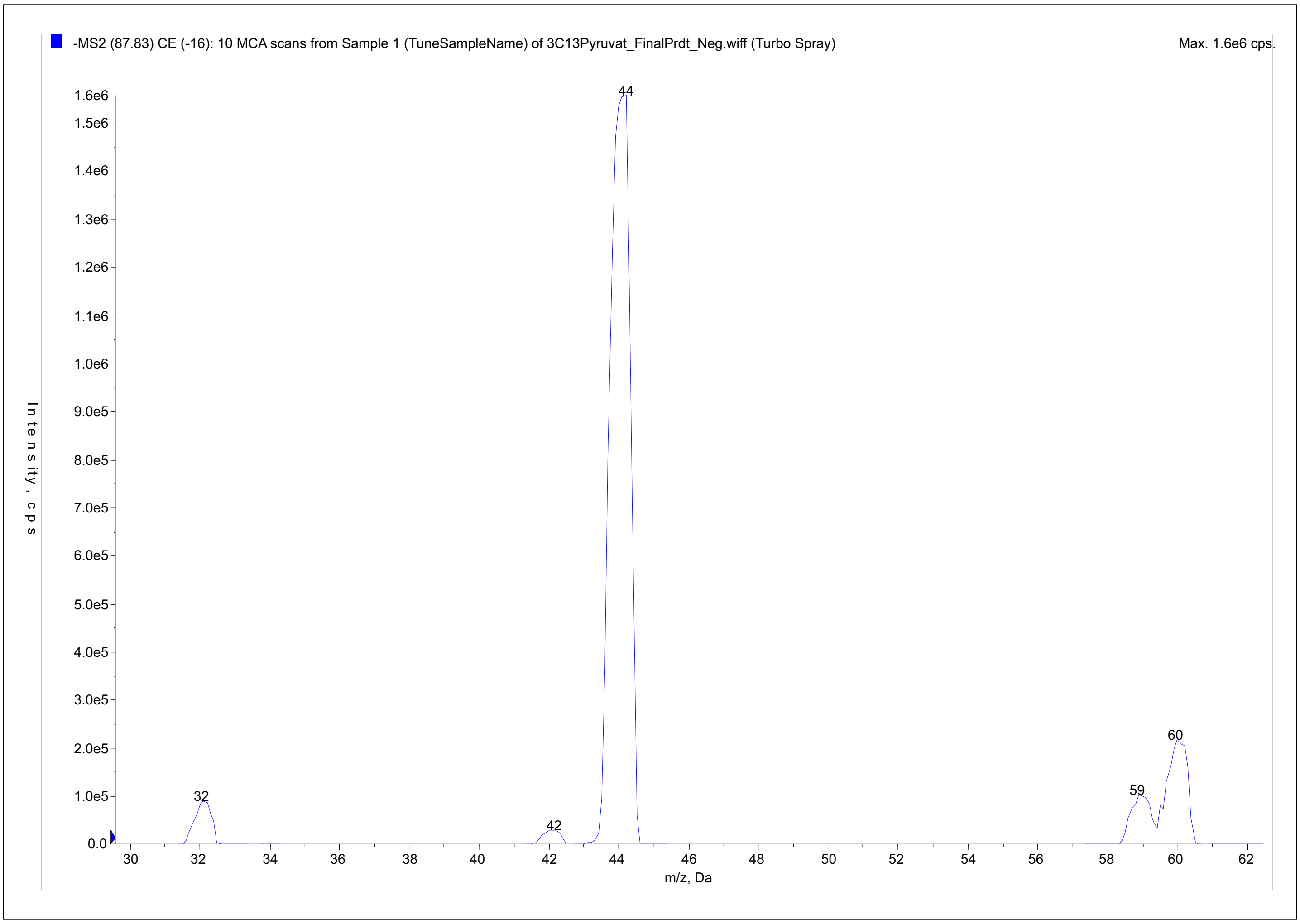

Chart S-89: The QqQ product ion spectrum of the [M-H]-ion of [3-^{13}C]pyruvate.

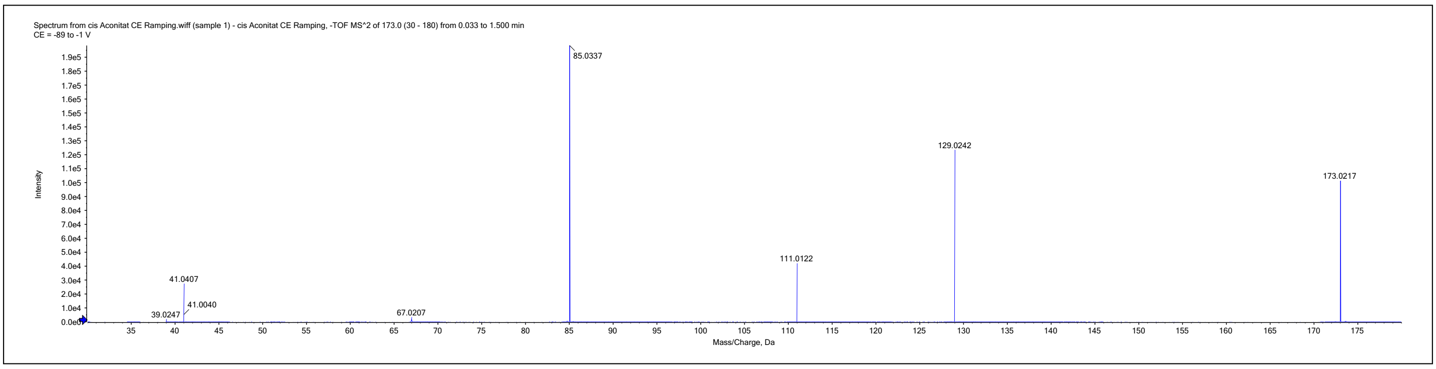

Chart S-90: The QTOF product ion spectrum of the [M-H]-ion of cis-aconitate.

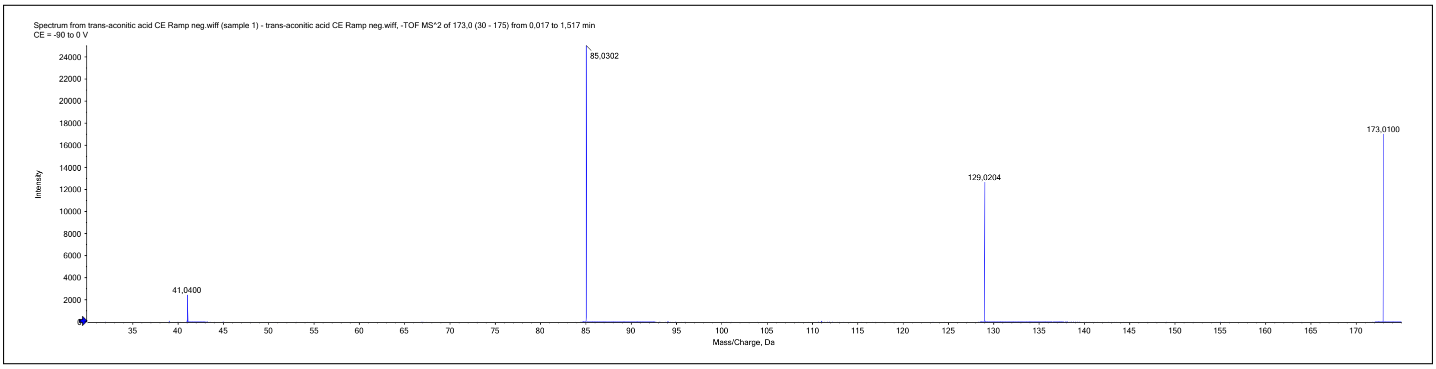

Chart S-91: The QTOF product ion spectrum of the [M-H]-ion of trans-aconitate.

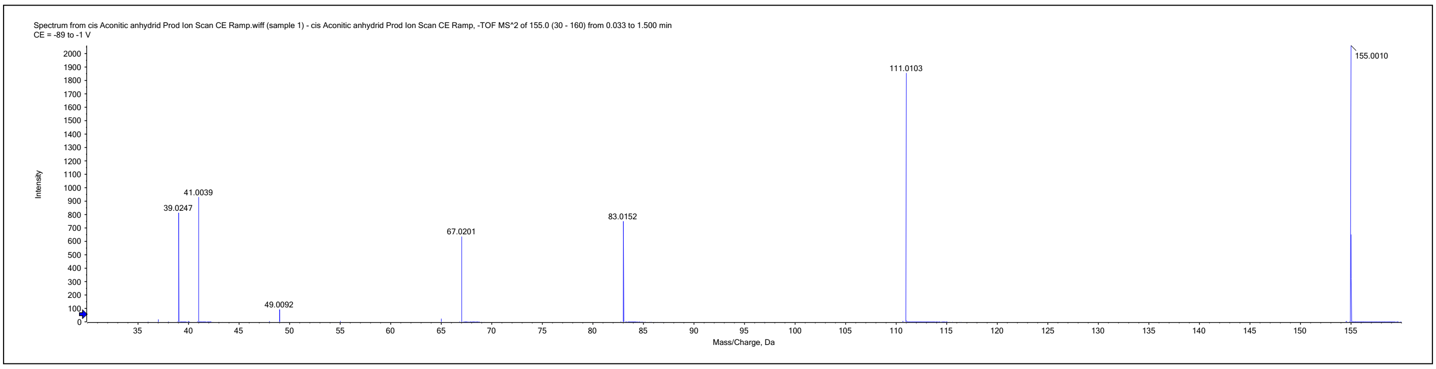

Chart S-92: The QTOF product ion spectrum of the [M-H]-ion of cis-aconitic anhydride.

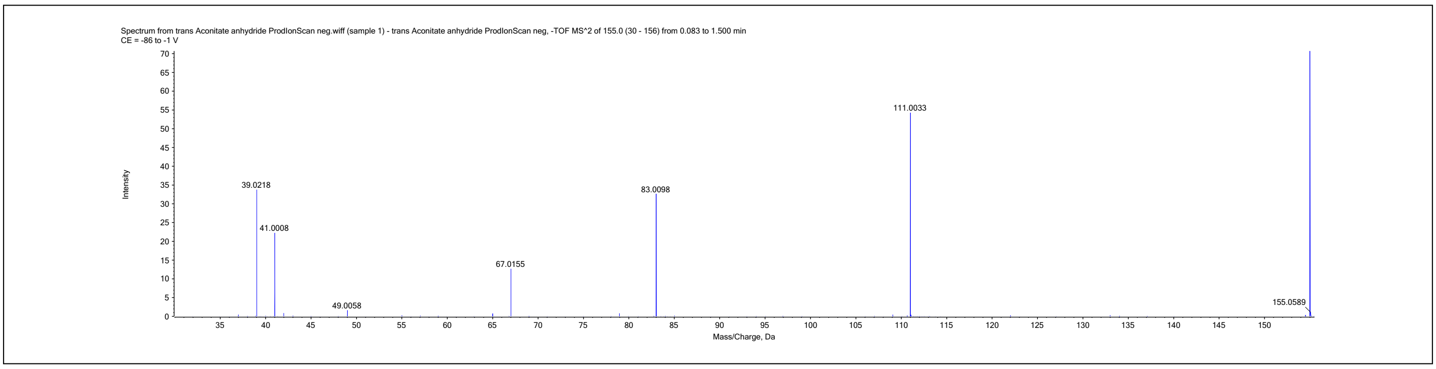

Chart S-93: The QTOF product ion spectrum of the [M-H]-ion of trans-aconitic anhydride.

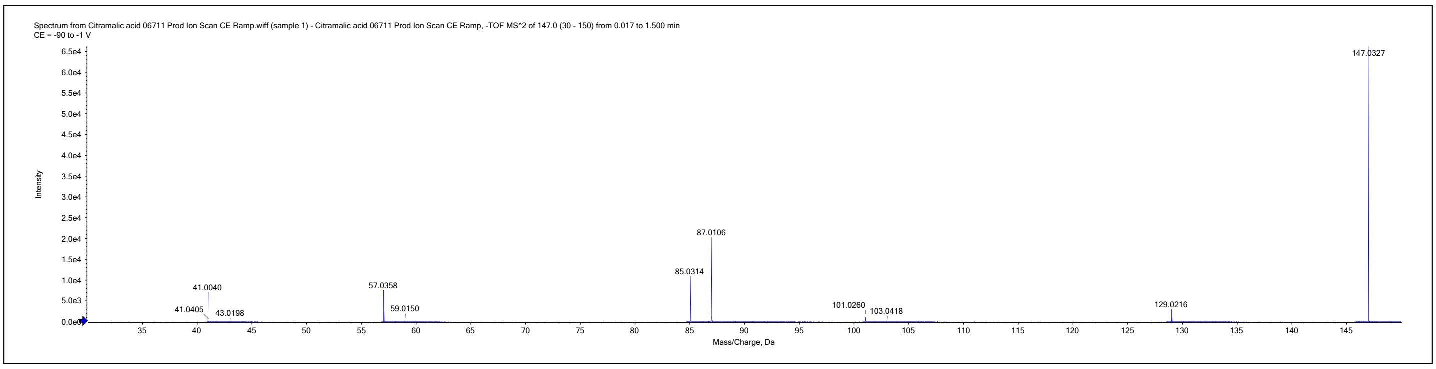

Chart S-94: The QTOF product ion spectrum of the [M-H]-ion of R-citramalate.

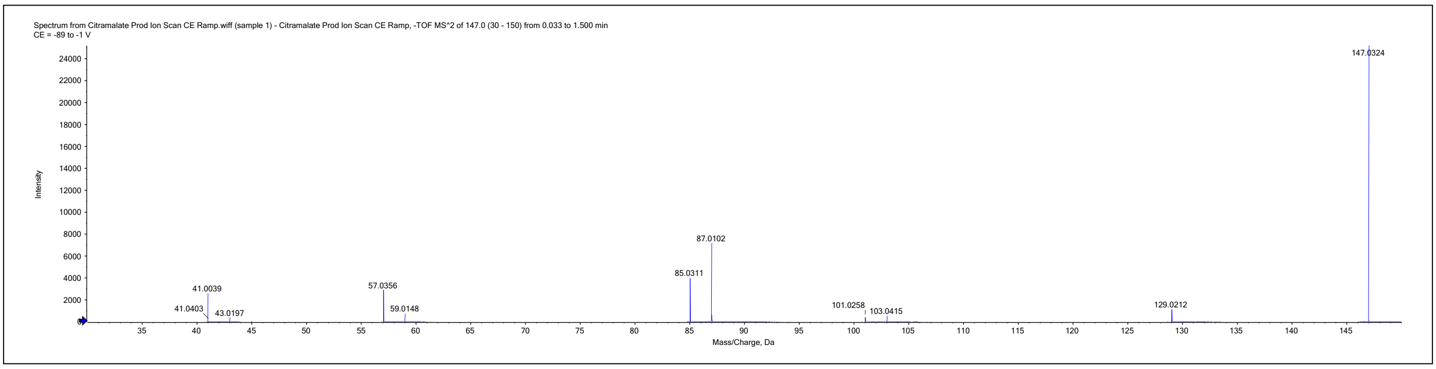

Chart S-95: The QTOF product ion spectrum of the [M-H]-ion of RS-citramalate.

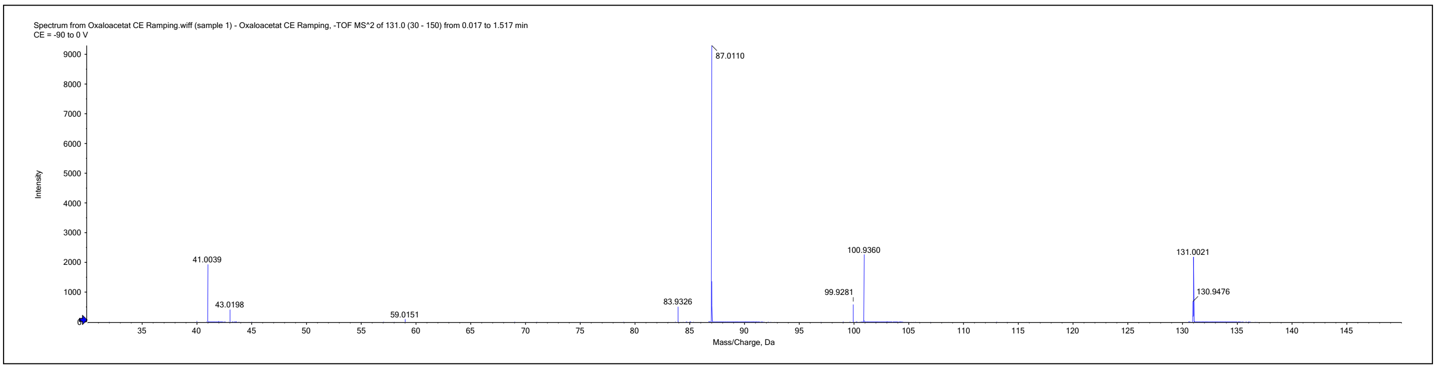

Chart S-96: The QTOF product ion spectrum of the [M-H]-ion of oxaloacetate.

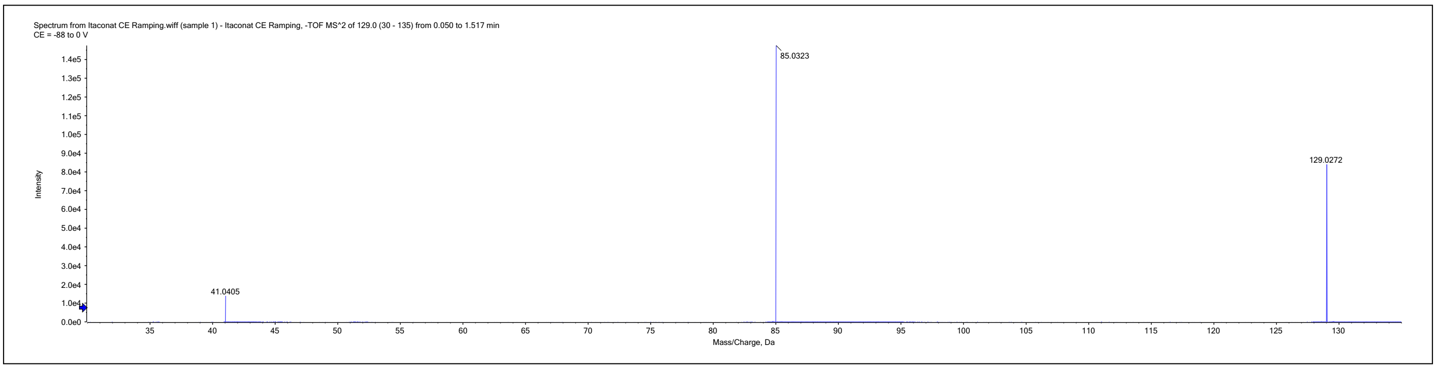

Chart S-97: The QTOF product ion spectrum of the [M-H]-ion of itaconate.

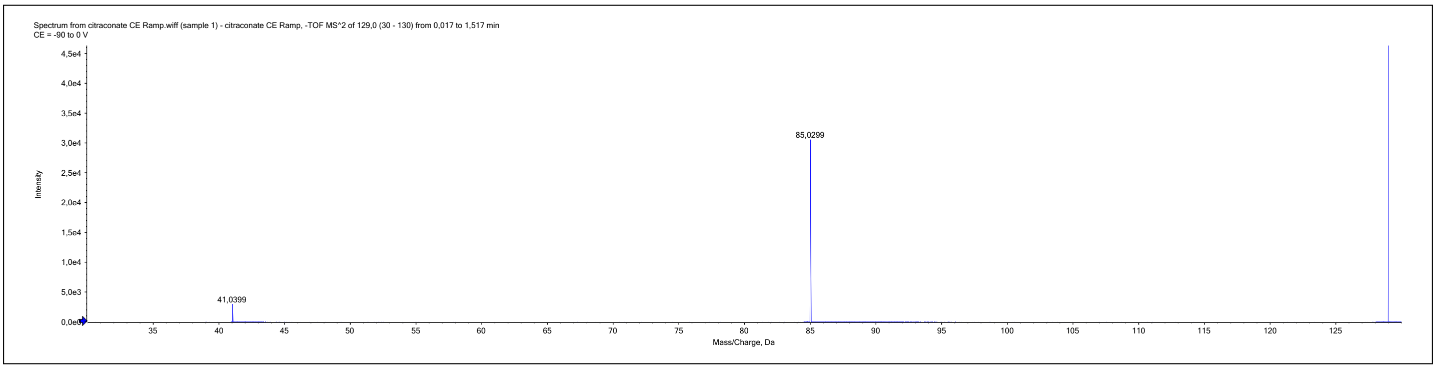

Chart S-98: The QTOF product ion spectrum of the [M-H]-ion of citraconate.

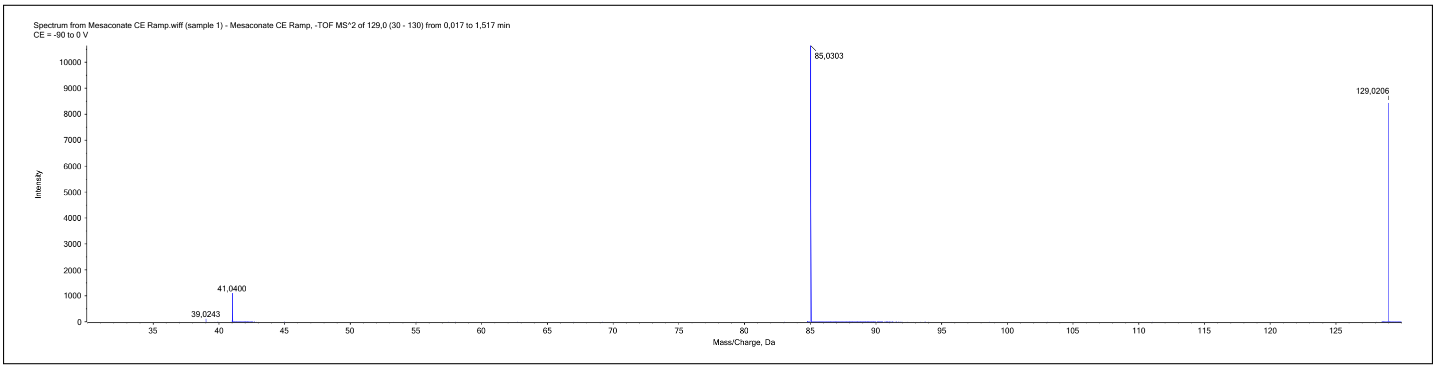

Chart S-99: The QTOF product ion spectrum of the [M-H]-ion of mesaconate.

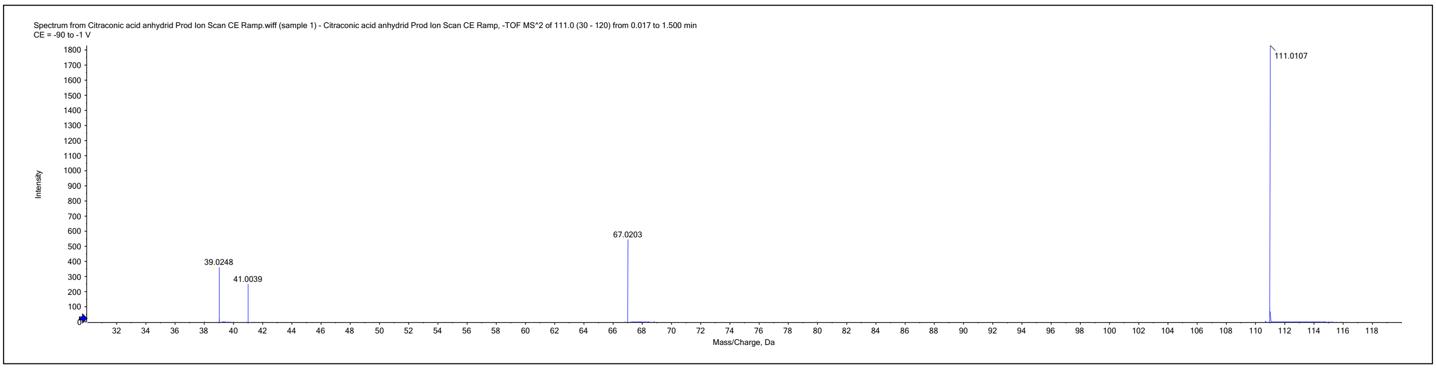

Chart S-100: The QTOF product ion spectrum of the [M-H]-ion of citraconic anhydride.

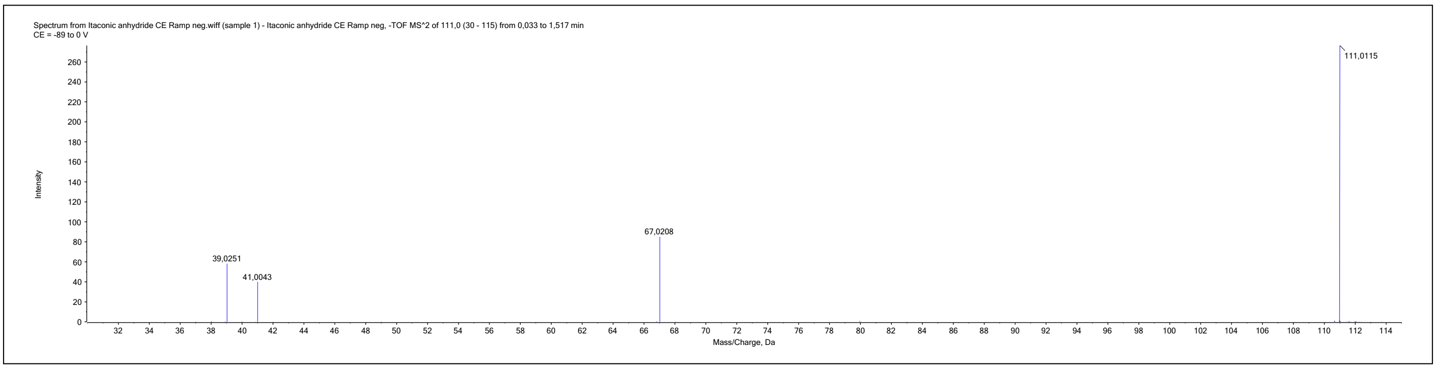

Chart S-101: The QTOF product ion spectrum of the [M-H]-ion of itaconic anhydride.

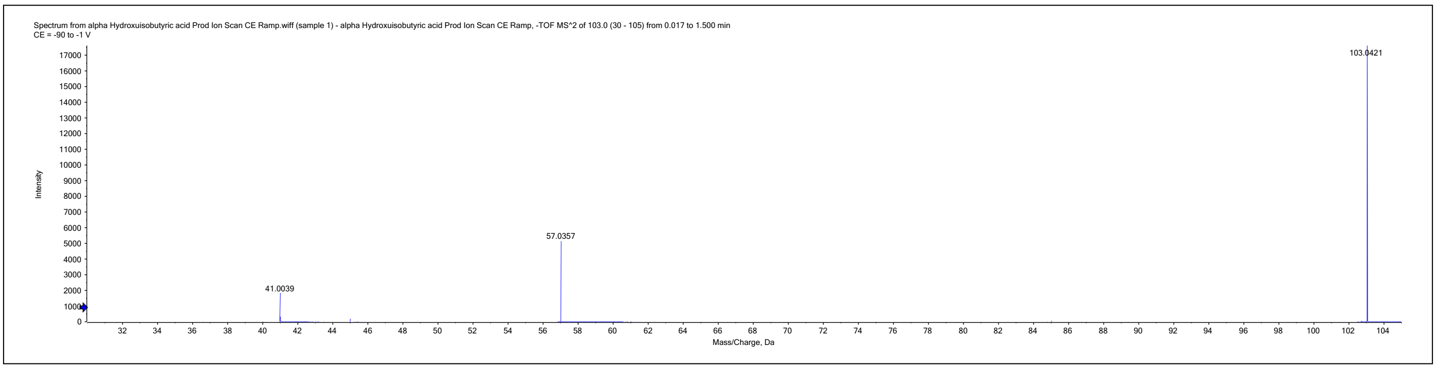

Chart S-102: The QTOF product ion spectrum of the [M-H]- ion of α-hydroxyisobutanoate.

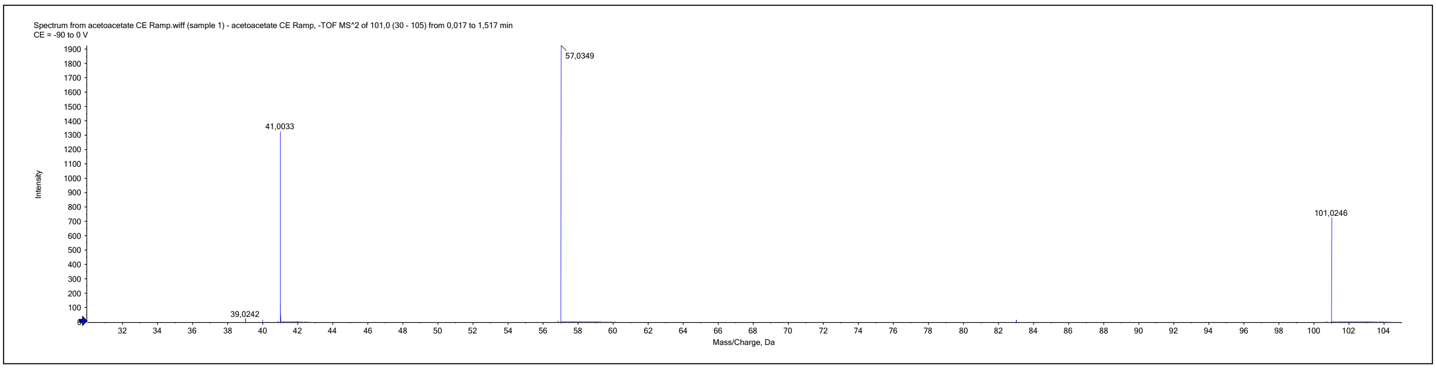

Chart S-103: The QTOF product ion spectrum of the [M-H]- ion of acetoacetate.

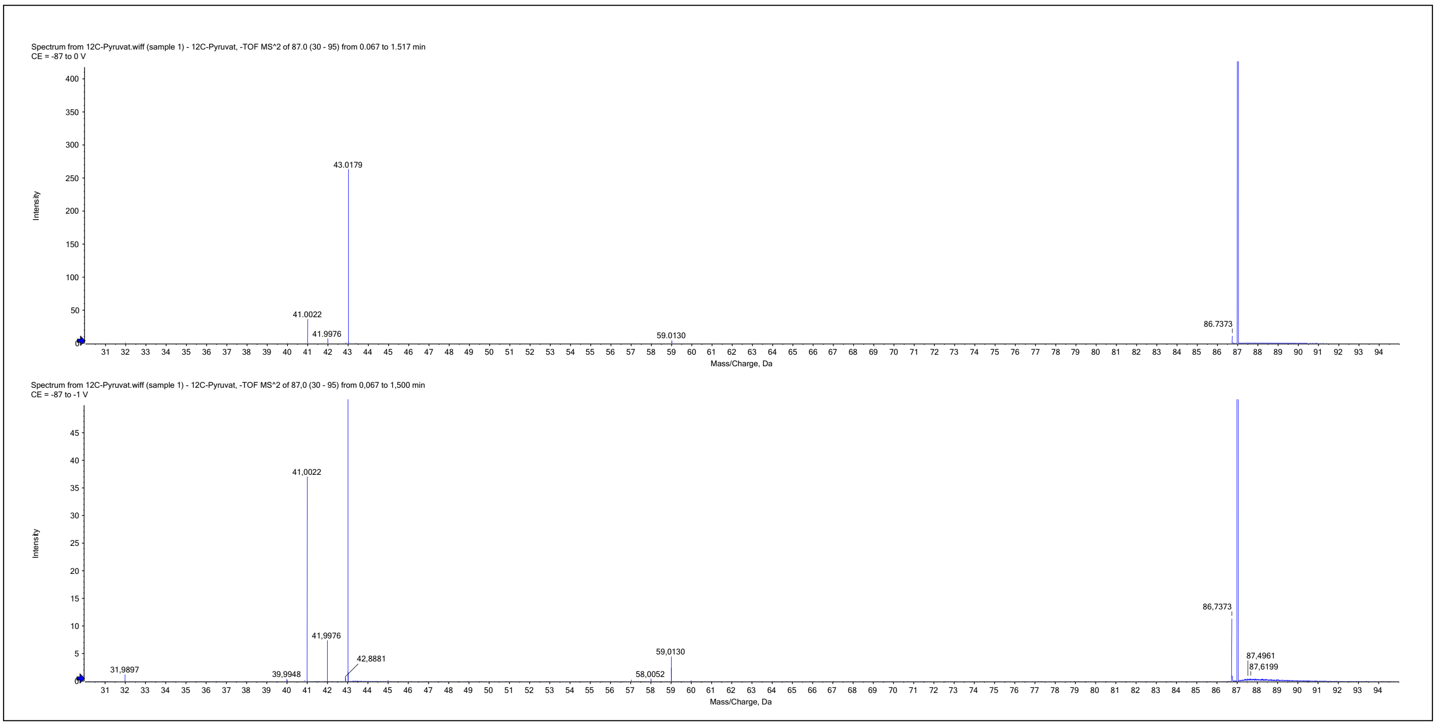

Chart S-104: The QTOF product ion spectrum of the [M-H]- ion of pyruvate.

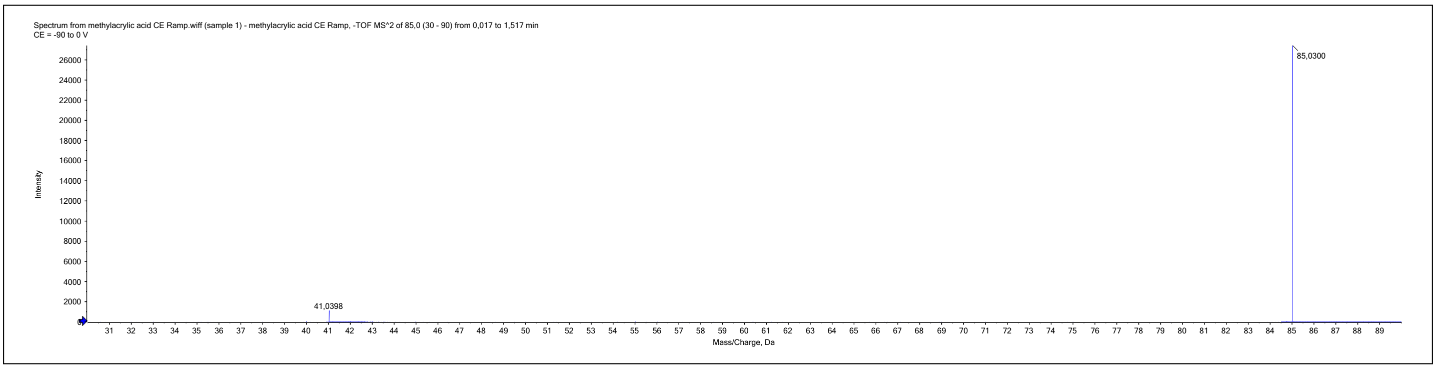

Chart S-105: The QTOF product ion spectrum of the [M-H]- ion of methylacrylate.

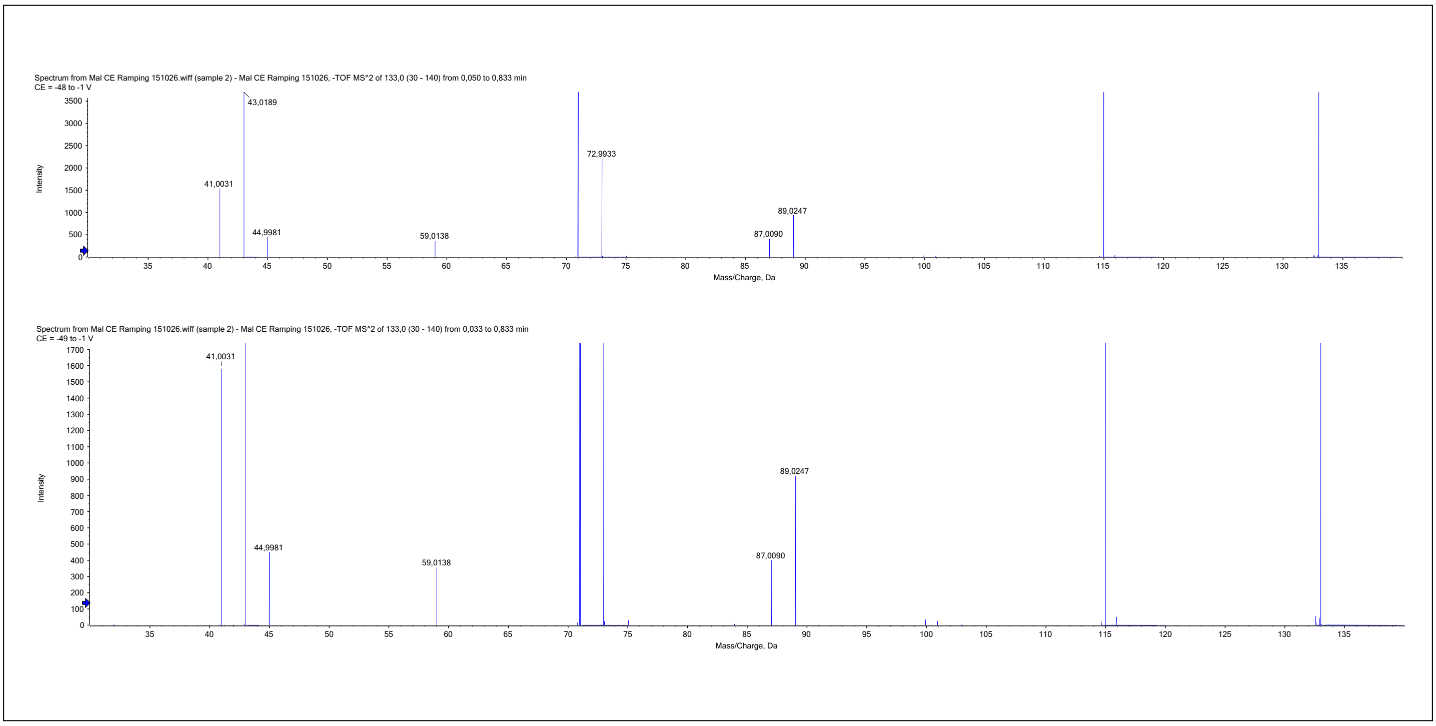

Chart S-106: The QTOF product ion spectrum of the [M-H]-ion of malate.

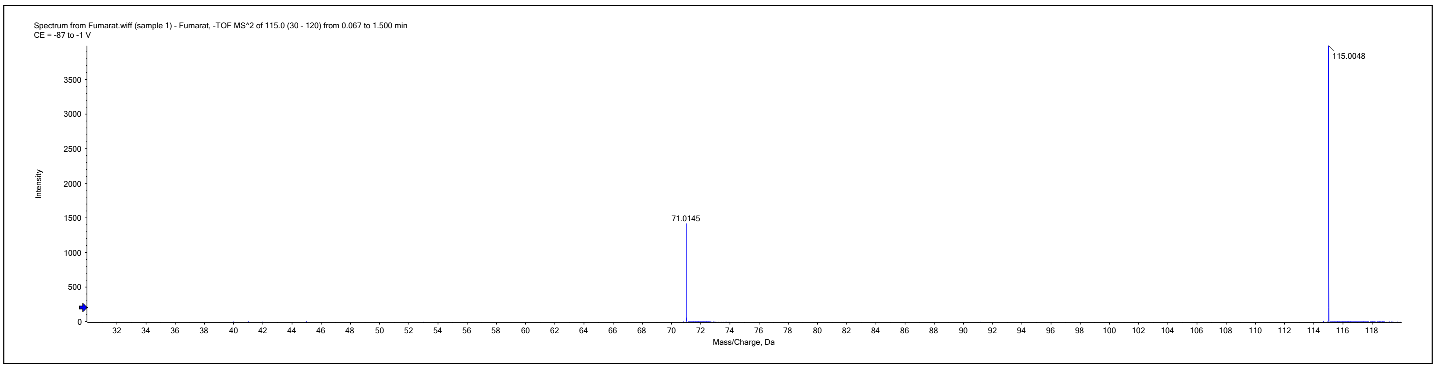

Chart S-107: The QTOF product ion spectrum of the [M-H]-ion of fumarate.

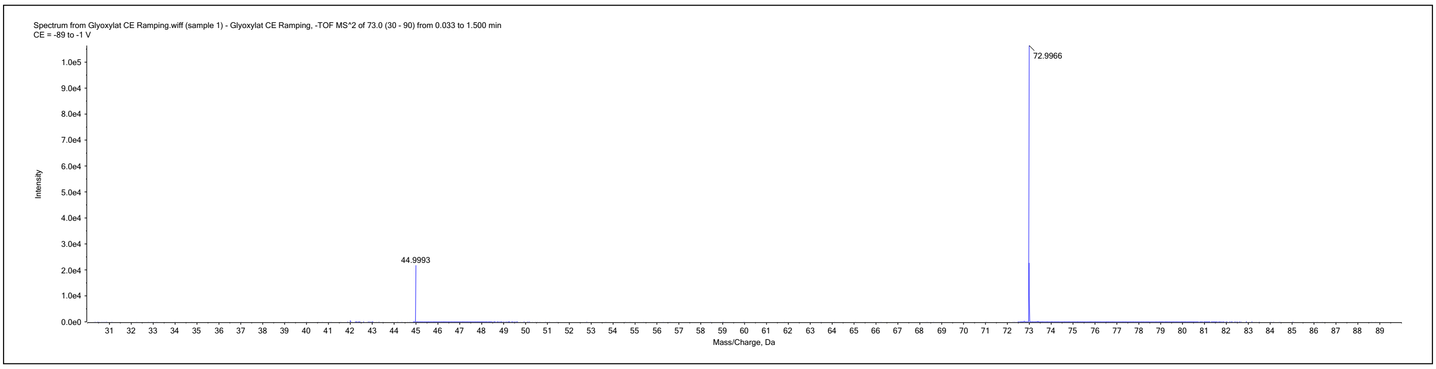

Chart S-108: The QTOF product ion spectrum of the [M-H]-ion of glyoxylate.

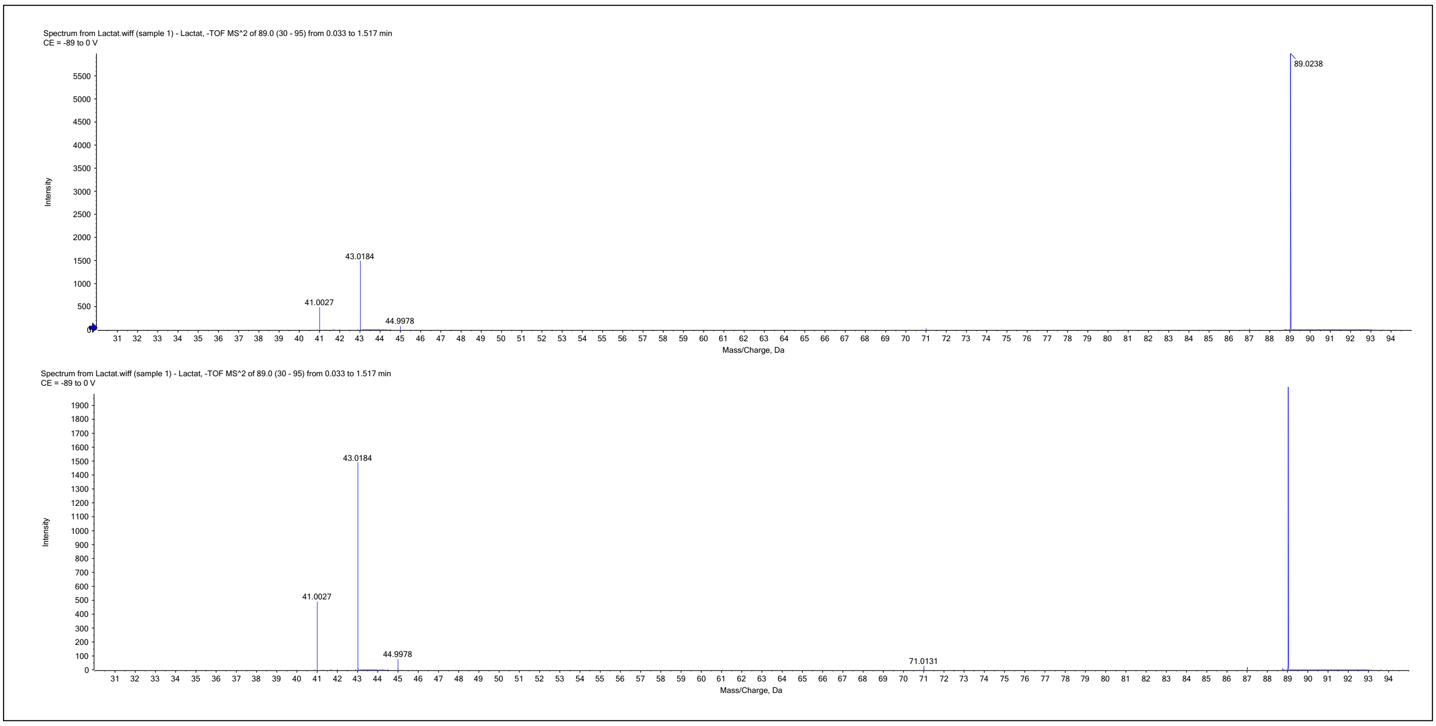

Chart S-109: The QTOF product ion spectrum of the [M-H]-ion of lactate.

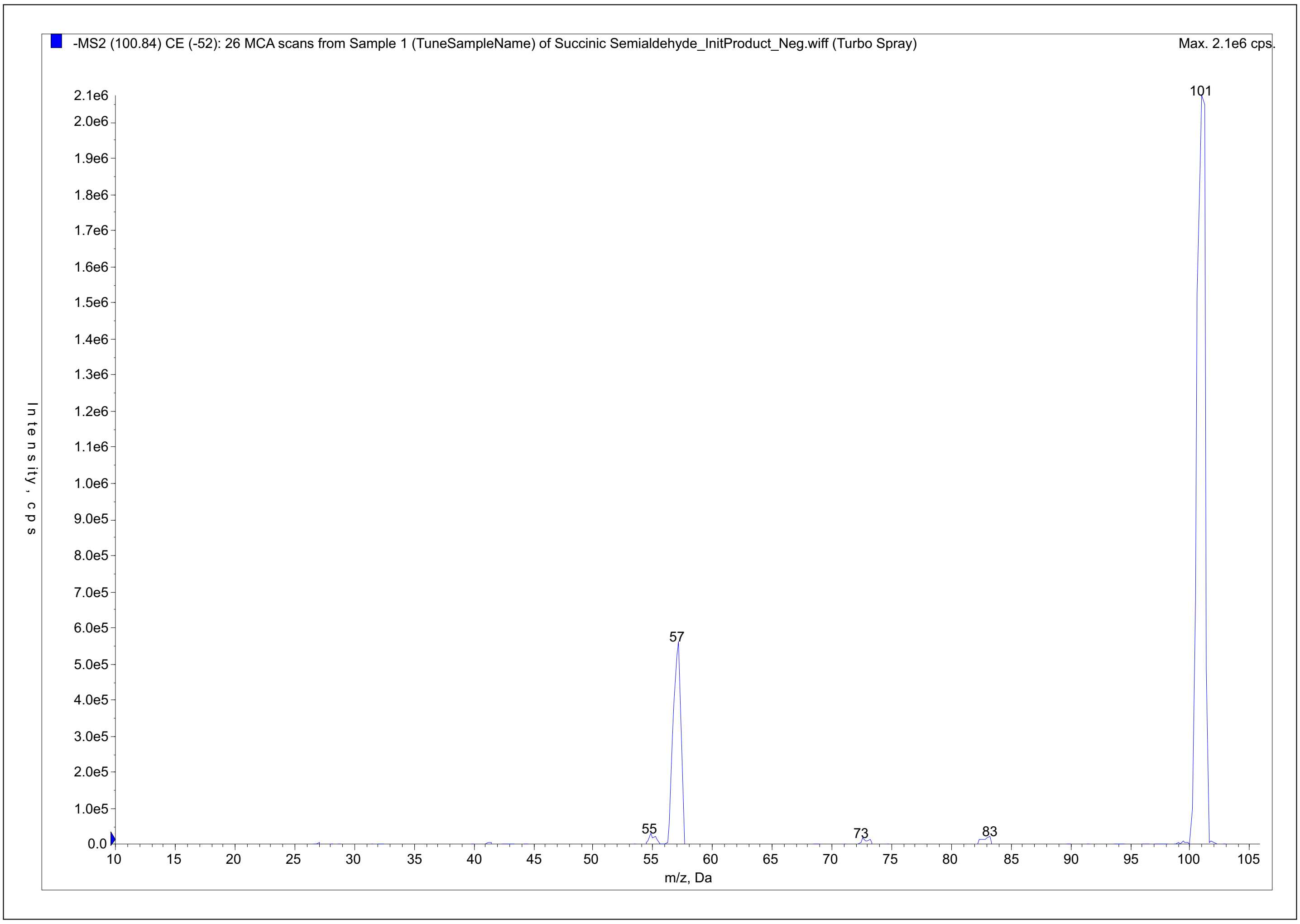

Chart S-110: The QqQ product ion spectrum of the [M-H]-ion of succinic semialdehyde.
